# An Eco-Friendly Ultrasound-Assisted Synthesis of Novel Fluorinated Pyridinium Salts-Based Hydrazones and Antimicrobial and Antitumor Screening

**DOI:** 10.3390/ijms17050766

**Published:** 2016-05-21

**Authors:** Nadjet Rezki, Salsabeel A. Al-Sodies, Mohamed R. Aouad, Sanaa Bardaweel, Mouslim Messali, El Sayed H. El Ashry

**Affiliations:** 1 Department of Chemistry, Faculty of Science, Taibah University, Al-Madinah Al-Munawarah 30002, Saudi Arabia; s7l_88@hotmail.com (S.A.A.-S.); aouadmohamedreda@yahoo.fr (M.R.A.); aboutasnim@yahoo.fr (M.M.); 2 Department of Chemistry, Faculty of Sciences, University of Sciences and Technology Mohamed Boudiaf, Laboratoire de Chimie and Electrochimie des Complexes Metalliques (LCECM) USTO-MB, P.O. Box 1505, El M`nouar, Oran 31000, Algeria; 3 Department of Pharmaceutical Sciences, Faculty of Pharmacy, University of Jordan, Amman 11942, Jordan; S.Bardaweel@ju.edu.jo; 4 Chemistry Department, Faculty of Science, Alexandria University, Alexandria 21500, Egypt; eelashry60@hotmail.com

**Keywords:** ultrasound irradiation, hydrazones, metathesis, antimicrobial activity, anticancer activity

## Abstract

The present work reports an efficient synthesis of fluorinated pyridinium salts-based hydrazones under both conventional and eco-friendly ultrasound procedures. The synthetic approach first involves the preparation of halogenated pyridinium salts through the condensation of isonicotinic acid hydrazide (**1**) with *p*-fluorobenzaldehyde (**2**) followed by the nucleophilic alkylation of the resulting *N*-(4-fluorobenzylidene)isonicotinohydrazide (**3**) with a different alkyl iodide. The iodide counteranion of **5**–**10** was subjected to an anion exchange metathesis reaction in the presence of an excess of the appropriate metal salts to afford a new series of fluorinated pyridinium salts tethering a hydrazone linkage **11**–**40**. Ultrasound irradiation led to higher yields in considerably less time than the conventional methods. The newly synthesized ILs were well-characterized with FT-IR, ^1^H NMR, ^13^C NMR, ^11^B, ^19^F, ^31^P and mass spectral analyses. The ILs were also screened for their antimicrobial and antitumor activities. Within the series, the salts tethering fluorinated counter anions **11**–**13**, **21**–**23**, **31**–**33** and **36**–**38** were found to be more potent against all bacterial and fungal strains at MIC 4–8 µg/mL. The *in vitro* antiproliferative activity was also investigated against four tumor cell lines (human ductal breast epithelial tumor **T47D**, human breast adenocarcinoma **MCF-7**, human epithelial carcinoma **HeLa** and human epithelial colorectal adenocarcinoma **Caco-2**) using the MTT assay, which revealed that promising antitumor activity was exhibited by compounds **5**, **12** and **14**.

## 1. Introduction

In recent years, the development of clean, safe and efficient eco-friendly protocols has become a major challenge in green chemistry. Ultrasound (US) has been extensively adopted as a promising green pathway [1] in several organic transformations. US was reported to drastically increase reaction rates, improve yields and provide high purity of products with an easy work-up [2,3,4]. An enhanced selectivity and reduction of chemical hazards using this safe ultrasound method have been well documented [2,3,4].

Ionic liquids (ILs) have emerged as fascinating new green solvents as alternatives to volatile organic solvents due to their sought-after properties such as negligible vapor pressure, high thermal stability, high ionic conductivity and high ability to solvate both polar and non-polar compounds [2,5]. Their most attractive features are their very low volatility, nonflammability and stability, which make them suitable for applications in diverse fields, such as organic synthesis, catalysis and biocatalysis, analytical chemistry, nanotechnology, food science and as function fluids (e.g., lubricants, heat transfer fluids and corrosion inhibitors) [4,5]. Recently, some acidic task-specific ILs were used as solvents and catalysts for the hydrolysis/conversion of cellulose and lignocellulosic biomass [6].

ILs have also demonstrated promising applications for medicinal chemistry, including antimicrobial, antiseptic, anticancer and anti-inflammatory activities [7].

Because the synthesis of ionic liquids [5,8] is not easy, chemists have developed several green protocols for the clean and safe synthesis of IL liquids including microwave (MW), ultrasound (US) and solvent-free reactions.

Hydrazones are an important class of Schiff bases and are widely used as antimalarial [9], anticancer [10], antibacterial [11], antifungal [12], antitubercular [13], antimicrobial [14] and antiviral [15] agents. The azomethine linkage on the Schiff base structures are responsible for their bioactivity, enabling them to serve as models for biologically important scaffolds [16]. In addition, there are many commonly used drugs incorporating hydrazone groups in their structures.

In view of the emerging importance of ILs and hydrazones as antimicrobial and anticancer agents and our general interest in ultrasound-assisted organic synthesis, we focus on developing a straightforward, safe and ecofriendly method for the synthesis of fluorinated pyridinium ILs tethering a hydrazone linkage under ultrasound irradiation and conventional heating. To the best of our knowledge, the synthesis of ionic liquids carrying a hydrazone functionality has not been previously reported in the literature. However, the synthesized compounds were found to be salts rather than ILs and were screened for their antimicrobial and antitumor activities in order to evaluate the synergistic effect resulting from the clubbing of these salts with azomethine hydrazone functionality in a single molecular frame work.

## 2. Results and Discussion

### 2.1. Chemistry

**Scheme 1:**
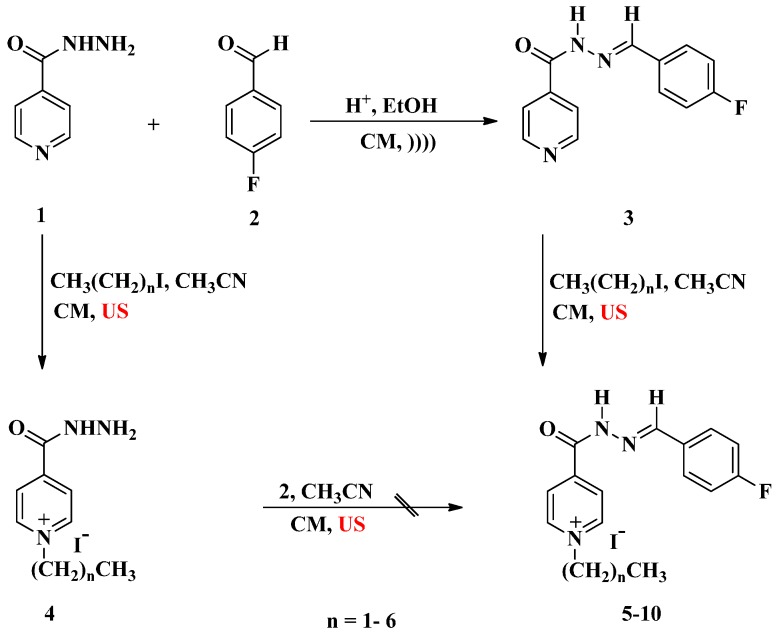
Synthesis of halogenated pyridinium salts tagged with hydrazone **5**–**10** under conventional method (CM) and ultrasound irradiation (US).

In the present work, several attempts to find the optimum conditions for the synthesis of new classes of fluorinated pyridinium ionic liquid-based hydrazone have been investigated under both conventional and ultrasound methods. These attempts led to the finding that the alkylation of isonicotinic acid hydrazide with several alkyl iodides in different solvents such as acetonitrile, toluene and methanol afforded very poor yields (24%–28%) of compound **4**, under either conventional heating or US. In addition, no reaction was observed under several attempts to condensate the resulting **4** with *p*-fluorobenzaldehyde (**2**) in the presence of a catalytic amount of HCl in boiling ethanol and/or under ultrasonic conditions (Scheme 1).

Conversely, the successful strategy for synthesizing the target **5**–**10** was based on the alkylation of *N*-(4-fluorobenzylidene)isonicotinohydrazide (**3**) with the appropriate alkyl halides under both conventional and US conditions. Thus, the condensation of acid hydrazide **1** with *p*-fluorobenzaldehyde (**2**) afforded the corresponding hydrazone **3** in excellent yield (90%) in refluxing ethanol for 1 h; a comparable yield has been obtained within 30 min under ultrasound irradiation. The resulting hydrazone **3** has been alkylated with different alkyl iodides, furnishing the target halogenated pyridinium salts **5**–**10** in 83%–91% yields, as shown in Scheme 1.

When the alkylation was carried out under ultrasound irradiation, great reductions in reaction time (12–14 h) were observed with higher yields (90%–94%) compared to those obtained under the classical method (72 h) (Table 1).

The structures of the hydrazones **5**–**10** have been established based on their mass and spectroscopic data (^1^H NMR, ^13^C NMR, ^19^F NMR). The NMR spectra of the synthesized compounds **5**–**10** measured in DMSO-*d*_6_ revealed the presence of a diastereomeric mixture (*i.e.*, *E*/*cis* and *E*/*trans*) for each imino-amide moiety.

The *N*-hexyl derivative **9** was selected to discuss the NMR data used to confirm the success of the quaternization reaction. From its ^1^H NMR spectrum, the appearance of the diagnostic C**H_3_** and NC**H_2_** as a triplet at δ_H_ 0.88 ppm and a doublet of doublets at δ_H_ 4.70 ppm, respectively, are clear evidence for the success of the alkylation reaction. The remaining methylene groups were also observed.

**Figure 1:**
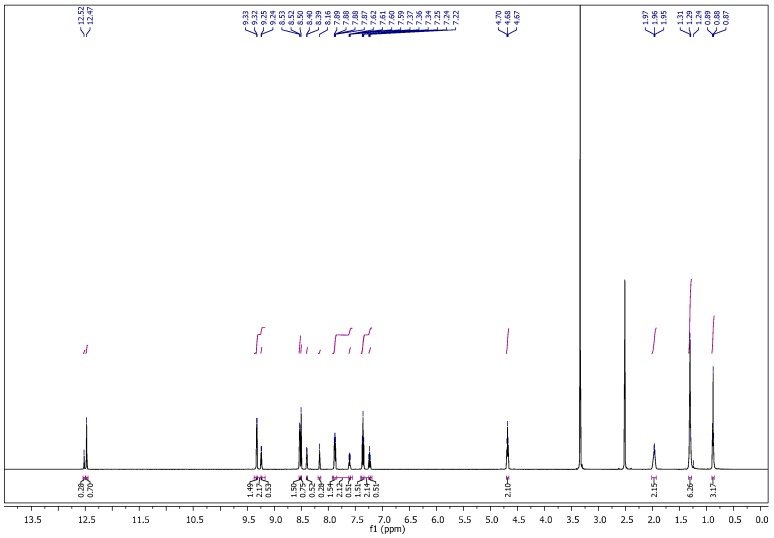
^1^H NMR spectrum of compound **9** in DMSO-*d*_6_.

**Figure 2:**
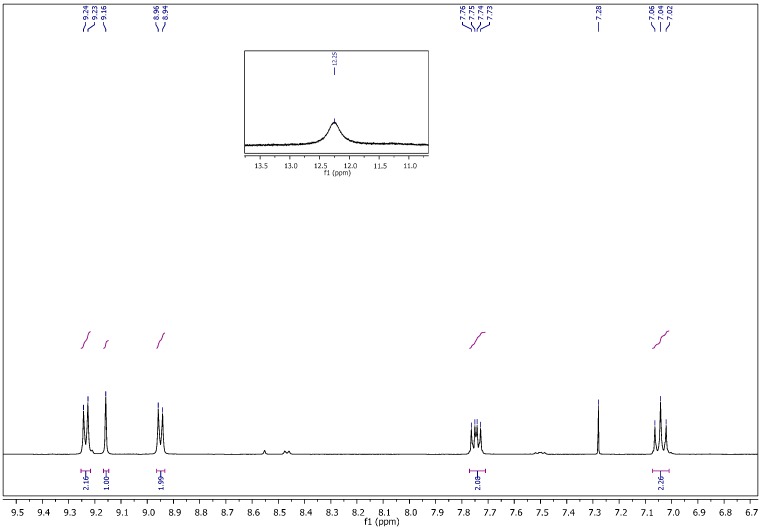
^1^H NMR spectrum of compound **9** in CDCl_3_.

The spectrum also revealed the presence of two singlets at δ_H_ 8.16 and 8.50 ppm, with a ratio of 1:3, which have a total integration of one proton characteristic of the imine proton (**H**C=N). In addition, the N**H** group split into two singlets at δ_H_ 12.47 and 12.52 ppm that have a total integration of one proton with the same ratio (Figure 1). Moreover, eight aromatic protons resonated in their appropriate chemical shifts with a similar isomeric pattern to that observed for the NH and H–C=N groups. To confirm the solvent effect for the isomerism of hydrazones, the ^1^H NMR spectrum of compound **9** was recorded in a less polar solvent (CDCl_3_). Consequently, one singlet signal was observed at δ_H_ 12.25 ppm for the N**H** proton and at δ_H_ 9.13 ppm for the **H**C=N proton, corresponding to the *cis* and *trans* conformers of the *E* isomer (Figure 2). These results agree with those previously reported in our work, where the hydrazone functionality was proven to exhibit *E*/*cis* and *E*/*trans* geometrical isomerism in polar solvents such as DMSO-*d*_6_, while only the *E*/*cis* or *E*/*trans* isomer was recorded in a less polar solvent (CDCl_3_) [17,18].

Further assignment of the diastereomer formation for compound **9** was supported by ^13^C NMR and dept-135 experiments. In the ^13^C NMR spectrum, each peak of compound **9** appeared as two sets of signals due to the presence of the diastereomeric mixture. In the aliphatic region, the methyl and N**C**H_2_ carbons resonated as two sets of signals at 61.43 and 61.51, respectively. The **C**=N and **C**=O groups of the *E*/*cis* and *E*/*trans* diastereomers also resonated as double peaks at δ_C_ 159.27–165.72 ppm.

**Figure 3:**
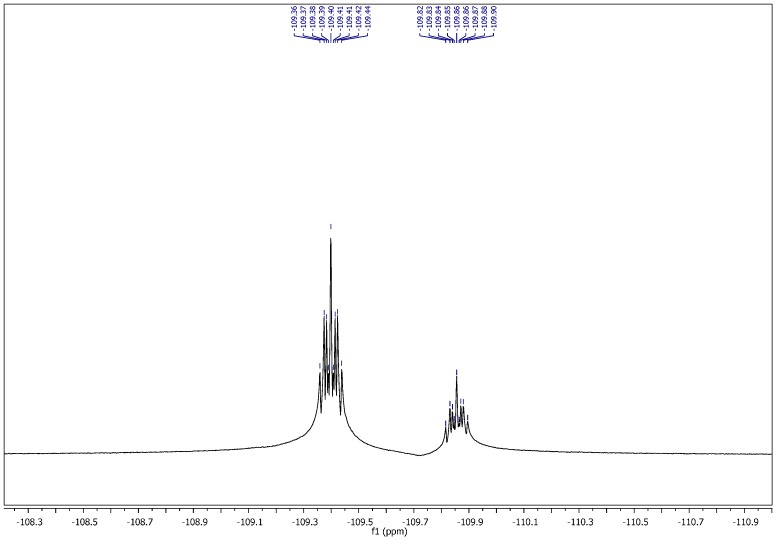
^19^F NMR spectrum of compound **9** in DMSO-*d*_6_.

The ^19^F NMR spectrum (Figure 3) also proved the formation of a diastereomeric mixture (*E*/*cis* and *E*/*trans*) through the appearance of two characteristic multiplets at δ_F_ −109.90 to −109.82 and −109.44 to −109.36 ppm, attributed to the aromatic fluorine atom.

The structure of compound **9** was also confirmed by the electron impact mass spectrum, which showed a molecular ion peak at 459.38 [M^+^].

**Scheme 2:**
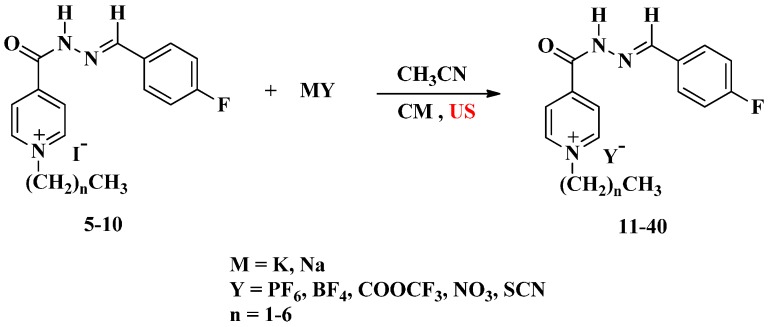
Synthesis of specific-based hydrazones **11**–**40**.

All the newly synthesized iodonated pyridinium salts **5**–**10** have been subjected to a metathesis reaction in which the iodide anion has been displaced by different anions. The anion exchange reactions were carried out by their refluxing with different metal salts such as NaBF_4_, KPF_6_, NaOCOCF_3_, NaSCN, or NaNO_3_, in acetonitrile as solvent for 16 h to give new specific-based hydrazones **11**–**40** in 80%–98% yields (Scheme 2).

When these reactions were assisted by ultrasound irradiation, 6 h were required to afford comparable yields of the same ILs (Table 2).

The analysis of the NMR spectra of compounds **11**–**40** revealed that their ^1^H and ^13^C NMR are practically the same as those recorded for their precursors **5**–**10**, with the isomeric splitting pattern. Accordingly, the ^31^P NMR, ^19^F NMR and mass spectra analyses have supported the success of the metathesis reaction. In the ^31^P NMR spectrum of compound **31**, the appearance of a characteristic multiplet signal at δ_P_ −157.37 to −131.02 ppm confirms the presence of the **P**F_6_^−^ anion. In addition, its ^19^F NMR spectrum displays two characteristic singlets at δ_F_ −71.10 and −69.22 ppm, confirming the presence of a fluorine atom in its P**F**_6_^−^ form, while the aromatic fluorine atom was assigned as two multiplets at δ_F_ (−109.90 to −109.82) ppm and (−109.44 to −109.36) ppm. In addition, the presence of a molecular ion peak at 473.39 [M^+^] in its mass spectrum supports the structure of compound **31**. The iodide anion exchange of **9** using NaBF_4_ as a metal salt led to the formation of compound **32**, with its structure supported by its ^11^B NMR and ^19^F NMR spectra. The appearance of a characteristic doublet at δ_B_ −1.28 ppm in the ^11^B NMR spectrum confirmed the incorporation of the boron anion in its structure.

The ^19^F NMR spectrum displays two doublets at δ_F_ −148.30 and −145.25 ppm, attributed to the fluorine anion (BF_4_^−^), while the aromatic fluorine was recorded at δ_F_ −109.90 to −109.82 ppm and −109.45 to −109.37 ppm as two multiplets. The structure of IL **32** has also been established based on its electron impact mass spectrum, which shows a molecular ion peak at 415.22 [M^+^]. The anion exchange with trifluoroacetate has also been investigated and gave IL **33**, as was also confirmed by its ^19^F NMR spectrum, which clearly shows a singlet at δ_F_ −73.50 ppm due to the CF_3_COO^−^ anion. The aromatic fluorine atom resonated at the expected area. The mass spectral data reveals the presence of the molecular ion peak at 441.18 [M^+^] as evidence for the formation of compound **33**.

Because **34** and **35** carrying NO_3_^−^ and/or SCN^−^ anion head-groups display similar ^1^H and ^13^C NMR spectra compared to their precursor **9**, their formation becomes more evident based on their mass spectra. The mass spectra of compounds **34** and **35** display molecular ion peaks at 390.37 [M^+^] and 386.56 [M^+^], respectively.

### 2.2. Biological Assay

#### 2.2.1. Antimicrobial Activity

Compounds **5**–**15**, **21**–**25** and **31**–**40** were assessed *in vitro* for their efficacy as antimicrobial agents by the minimum inhibitory concentration (MIC) using the broth dilution method [19,20] against six standard bacterial strains (*Streptococcus pneumonia*, *Bacillus subtilis*, *Staphylococcus aureus*, *Pseudomonas aeuroginosa*, *Escherichia coli* and *Klebsiella pneumonia*) and two fungi (*Aspergillus fumigatus* and *Candida albicans*). The MIC results are summarized in Table 3.

The antibacterial activity screening for the halogenated pyridinium salts **5**–**10** against all of the bacterial strains demonstrated that all the compounds reveal promising antibacterial activities, with an MIC range of 8–16 µg/mL. In contrast, the opposite result was observed for the two fungal species, towards which the compounds showed no activity.

From the antibacterial activity results of compounds **11**–**40**, it can be stated that those resulting from the metathetical anion exchange with fluorinated metal salts (PF_6_^−^, BF_4_^−^ or CF_3_COO^−^) are more effective against all bacterial strains at an MIC of 4–8 µg/mL.

The antifungal bioassay results summarized in Table 3 reveal that, among the tested salts **11**–**40**, compounds **21**–**23**, **31**–**33** and **36**–**38** show good to excellent potency against all of the tested fungal strains, with an MIC range of 8–16 µg/mL. In fact, the highest antifungal activity, with an MIC of 8 µg/mL, was exhibited by compounds **33** and **38**, possessing a CF_3_COO^−^ counter anion and a C_6_ to C_7_ alkyl chain in the cation head group.

The antimicrobial activity and structure activity relationship reveal that the promising activity displayed by the halogenated **5**–**10** against all of the bacterial strains is presumably due to the chain length. The incorporation of a fluorine atom was found to dramatically increase the antimicrobial activity, as exhibited by the fluorinated **11**–**13**, **21**–**23**, **31**–**33** and **36**–**38** carrying PF_6_^−^, BF_4_^−^ and/or CF_3_COO^−^. In addition, the metathetic exchange with these fluorinated metal salts resulted in higher antifungal activity.

In the antimicrobial screening, it was observed that compounds with long alkyl side chains possessing a fluorine atom in their anion head-group (PF_6_^−^, BF_4_^−^ and CF_3_COO^−^) exhibit excellent activity compared to the corresponding halogenated precursors against all of the bacterial strains, indicating the influence of the presence of the fluorine atom in the structure of the ionic liquids.

#### 2.2.2. Antiproliferative Activity

An *in vitro* evaluation of the antiproliferative activities of the newly synthesized compounds was investigated against four human tumor cell lines by using the protocol described in ISO 10993-5 [21]. The results are presented as IC_50_ ± SD values (Table 4). Each experiment was repeated three times. IC_50_ concentrations were obtained from the dose–response curves using Graph Pad Prism Software 5.

Only the compounds shown in Table 4 demonstrated a measurable IC_50_ against the tested cancer cell lines and thus can be used as model compounds for the construction of novel anticancer drugs. Interestingly, reducing the chain length of the compounds yielded more potent cytotoxic activities, suggesting a steric factor mediating either transport or molecular interaction with the cellular targets.

## 3. Experimental Section

### 3.1. General

Melting points were recorded on a Stuart Scientific SMP1 apparatus (Stuart, Red Hill, UK) and are uncorrected. The IR spectra were recorded using an SHIMADZU FTIR-8400S spectrometer (SHIMADZU, Boston, MA, USA). The NMR spectra were measured with a Bruker spectrometer (400 and 600 MHz, Brucker, Fällanden, Switzerland) using Tetramethylsilane (TMS) (0.00 ppm) as an internal standard. The ESI and EI mass spectra were measured by Finnigan LCQ and Finnigan MAT 95XL spectrometers (Finnigan, Darmstadt, Germany), respectively. Ultrasound-assisted reactions were performed in a Kunshan KQ-250B ultrasound cleaner (50 KHz, 240 W, Kunshan Ultrasonic Instrument, Kunshan, China).

### 3.2. Synthesis and Characterization of N-(4-Fluorobenzylidene) Isonicotinohydrazide (**3**)

#### 3.2.1. Conventional Method

A mixture of isonicotinic acid hydrazide (**1**) (1 mmol) in ethanol (25 mL) and 4-fluorobenzaldehyde (**2**) (1.2 mmol) with three drops of hydrochloric acid was refluxed for 1 h. After cooling, the excess solvent was removed under reduced pressure. The product formed was collected and crystallized from ethyl acetate to furnish the desired compound **3** mp: 214–215 °C (Lit. mp: 216–220 °C) [22]. ^1^H NMR (600 MHz, DMSO-*d*_6_): δ_H_ = 7.27 (dd, 0.25H, *J* = 6 Hz, 12 Hz, Ar–**H**), 7.35 (dd, 1.75H, *J* = 6 Hz, 12 Hz, Ar–**H**), 7.59 (t, 0.25H, *J* = 6 Hz, Ar–**H**), 7.67 (d, 0.25H, *J* = 6 Hz, Ar–**H**), 7.82–7.84 (m, 3.5H, *J* = 6 Hz, Ar–**H**), 8.11 (s, 0.1H, **H**–C=N), 8.48 (s, 0.9H, **H**–C=N), 8.75 (d, 0.25H, *J* = 6 Hz, Ar–**H**), 8.81 (d, 1.75H, *J* = 6 Hz, Ar–**H**), 12.04 (s, 0.2H, CON**H**), 12.09 (s, 0.8H, CON**H**). ^13^C NMR (150 MHz, DMSO-*d*_6_): δ_C_ = 116.38, 116.53, 121.98, 123.53, 129.92, 129.98, 131.12, 140.89, 148.34, 150.02, 150.81 (Ar–**C**), 162.08, 162.94, 164.58 (**C**=N, **C**=O).

#### 3.2.2. Ultrasound Method

A mixture of isonicotinic acid hydrazide (**1**) (1 mmol) in ethanol (25 mL) and 4-fluorobenzaldehyde (**2**) (1.2 mmol) with a few drops of hydrochloric acid was irradiated by ultrasound irradiation for 30 min at room temperature. The reaction proceeded as described above to furnish the same compound **3**.

### 3.3. General Procedures for the Synthesis of Pyridinium Tagged Hydrazones **5**–**10**

#### 3.3.1. Conventional Method

A mixture of compound **3** (1 mmol) and the appropriate alkyl iodide with chain lengths ranging from C_2_ to C_7_ (1.5 mmol) in acetonitrile (30 mL) was refluxed for 72 h. After cooling, the solvent was removed under reduced pressure, and the solid formed was collected by filtration, washed with acetonitrile, and crystallized from dichloromethane to afford the desired pyridinium hydrazones **5**–**10**.

#### 3.3.2. Ultrasound Method

A mixture of compound **1** (1 mmol) and the appropriate alkyl iodide with chain lengths ranging from C_2_ to C_7_ (1.5 mmol) in acetonitrile (30 mL) was irradiated by ultrasound irradiation at room temperature. The reaction proceeded as described above to afford the desired pyridinium hydrazones **5**–**10**.

*1-Ethyl-4-(2-(4-fluorobenzylidene)hydrazinecarbonyl)pyridinium iodide* (***5***). It was obtained as yellow crystals; mp: 223–224 °C. FT-IR (KBr), cm^−1^: $\bar{V}$ = 1613 (C=N), 1690 (C=O), 2893, 2960 (Al–H), 3075 (Ar–H). ^1^H NMR (600 MHz, DMSO-*d*_6_): δ_H_ = 1.58–1.63 (m, 3H, C**H**_3_), 4.70–4.75 (m, 2H, NC**H**_2_), 7.26 (dd, 0.5H, *J* = 6 Hz, 12 Hz, Ar–**H**), 7.37 (dd, 1.5H, *J* = 6 Hz, 12 Hz, Ar–**H**), 7.63 (dd, 0.5H, *J* = 6 Hz, 12 Hz, Ar–**H**), 7.89 (dd, 1.5H, *J* = 6 Hz, 12 Hz, Ar–**H**), 8.17 (s, 0.25H, **H**–C=N), 8.40 (d, 0.5H, *J* = 6 Hz, Ar–**H**), 8.51 (s, 0.75H, **H**–C=N), 8.53 (d, 1.5H, *J* = 6 Hz, Ar–**H**), 9.26 (d, 0.5H, *J* = 6 Hz, Ar–**H**), 9.35 (d, 1.5H, *J* = 6 Hz, Ar–**H**), 12.48 (s, 0.8H, CON**H**), 12.51 (s, 0.2H, CON**H**).^13^C NMR (150 MHz, DMSO-*d*_6_): δ_C_ = 16.59, 16.72 (**C**H_3_), 57.07, 57.16 (N**C**H_2_), 116.32, 116.46, 116.51, 116.65, 126.60, 127.62, 129.91, 129.97, 130.24, 130.30, 130.50, 130.70, 130.71, 145.36, 145.62, 146.01, 147.75, 149.84, 149.99 (Ar–**C**), 159.26, 162.86, 163.18, 164.83, 165.70 (**C**=N, **C**=O). ^19^F NMR (377 MHz, DMSO-*d*_6_): δ_F_ = (−109.89 to −109.81), (−109.43 to −109.35) (2m, 1F, Ar–**F**). MS (ES) *m*/*z* = 399.36 [M^+^].

*4-(2-(4-Fluorobenzylidene)hydrazinecarbonyl)-1-propylpyridinium iodide* (***6***). It was obtained as yellow crystals; mp: 186–187 °C. FT-IR (KBr), cm^−1^: $\bar{V}$ = 1615 (C=N), 1689 (C=O), 2890, 2955 (Al–H), 3065 (Ar–H). ^1^H NMR (400 MHz, DMSO-*d*_6_): δ_H_ = 0.90–0.95 (m, 3H, C**H**_3_), 1.95–2.04 (m, 2H, NCH_2_C**H**_2_), 4.68 (dd, 2H, *J* = 4 Hz, 8 Hz, NC**H**_2_), 7.27 (dd, 0.5H, *J* = 8 Hz, 12 Hz, Ar–**H**), 7.38 (dd, 1.5H, *J* = 8 Hz, 12 Hz, Ar–**H**), 7.62 (dd, 0.5H, *J* = 4 Hz, 8 Hz, Ar–**H**), 7.89 (dd, 1.5H, *J* = 4 Hz, 8 Hz, Ar–**H**), 8.16 (s, 0.25H, **H**–C=N), 8.41 (d, 0.5H, *J* = 4 Hz, Ar–**H**), 8.51 (s, 0.75H, **H**–C=N), 8.55 (d, 1.5H, *J* = 8 Hz, Ar–**H**), 9.25 (d, 0.5H, *J* = 4 Hz, Ar–**H**), 9.34 (d, 1.5H, *J* = 8 Hz, Ar–**H**), 12.49 (s, 0.75H, CON**H**), 12.53 (s, 0.25H, CON**H**). ^13^C NMR (100 MHz, DMSO-*d*_6_): δ_C_ = 9.27, 9.30 (**C**H_3_), 23.11, 23.19 (NCH_2_**C**H_2_), 61.30, 61.38 (N**C**H_2_), 114.86, 115.06, 115.27, 125.22, 126.18, 128.43, 128.52, 128.81, 128.90, 129.11, 129.26, 129.29, 144.14, 144.78, 146.41, 148.39, 148.72 (Ar–**C**), 157.85, 161.35, 163.82, 164.30 (**C**=N, **C**=O). ^19^F NMR (377 MHz, DMSO-*d*_6_): δ_F_ = (−109.89 to −109.83), (−109.41 to −109.33) (2m, 1F, Ar–**F**). MS (ES) *m*/*z* = 413.35 [M^+^].

*1-Butyl-4-(2-(4-fluorobenzylidene)hydrazinecarbonyl)pyridinium iodide* (***7***). It was obtained as yellow crystals; mp: 184–185 °C. FT-IR (KBr), cm^−1^: $\bar{V}$ = 1620 (C=N), 1687 (C=O), 2882, 2961 (Al–H), 3071 (Ar–H). ^1^H NMR (600 MHz, DMSO-*d*_6_): δ_H_ = 0.93–0.96 (m, 3H, C**H**_3_), 1.30–1.38 (m, 2H, C**H**_2_CH_3_), 1.93–2.00 (m, 2H, NCH_2_C**H**_2_), 4.71 (dd, 2H, *J* = 6 Hz, 12 Hz, NC**H**_2_), 7.26 (dd, 0.5H, *J* = 6 Hz, 12 Hz, Ar–**H**), 7.37 (dd, 1.5H, *J* = 6 Hz, 12 Hz, Ar–**H**), 7.62 (dd, 0.5H, *J* = 6 Hz, 12 Hz, Ar–**H**), 7.89 (dd, 1.5H, *J* = 6 Hz, 12 Hz, Ar–**H**), 8.16 (s, 0.25H, **H**–C=N), 8.40 (d, 0.5H, *J* = 6 Hz, Ar–**H**), 8.50 (s, 0.75H, **H**–C=N), 8.54 (d, 1.5H, *J* = 6 Hz, Ar–**H**), 9.25 (d, 0.5H, *J* = 6 Hz, Ar–**H**), 9.33 (d, 1.5H, *J* = 6 Hz, Ar–**H**), 12.48 (bs, 1H, CON**H**). ^13^C NMR (150 MHz, DMSO-*d*_6_): δ_C_ = 13.79, 13.81 (**C**H_3_), 19.23, 19.30 (**C**H_2_CH_3_), 32.99, 33.08 (NCH_2_**C**H_2_), 61.22, 61.29 (N**C**H_2_), 116.31, 116.46, 116.51, 116.65, 126.65, 127.62, 129.86, 129.92, 130.24, 130.30, 130.54, 130.72, 145.58, 146.19, 147.82, 149.82 (Ar–**C**), 159.26, 163.19, 164.84, 165.71 (**C**=N, **C**=O). ^19^F NMR (377 MHz, DMSO-*d*_6_): δ_F_ = (−109.88 to −109.84), (−109.41 to −109.33) (2m, 1F, Ar–**F**). MS (ES) *m*/*z* = 427.28 [M^+^].

*4-(2-(4-Fluorobenzylidene)hydrazinecarbonyl)-1-pentylpyridinium iodide* (***8***). It was obtained as yellow crystals; mp: 216–217 °C. FT-IR (KBr), cm^−1^: $\bar{V}$ = 1622 (C=N), 1682 (C=O), 2891, 2970 (Al–H), 3070 (Ar–H). ^1^H NMR (400 MHz, DMSO-*d*_6_): δ_H_ = 0.86–0.90 (m, 3H, C**H**_3_), 1.25–1.37 (m, 4H, 2×C**H**_2_), 1.93–2.02 (m, 2H, NCH_2_C**H**_2_), 4.68 (t, 2H, *J* = 8 Hz, NC**H**_2_), 7.23 (t, 0.5H, *J* = 8 Hz, Ar–**H**), 7.37 (dd, 1.5H, *J* = 8 Hz, 12 Hz, Ar–**H**), 7.62 (dd, 0.5H, *J* = 4 Hz, 8 Hz, Ar–**H**), 7.89 (dd, 1.5H, *J* = 4 Hz, 8 Hz, Ar–**H**), 8.15 (s, 0.25H, **H**–C=N), 8.40 (d, 0.5H, *J* = 8 Hz, Ar–**H**), 8.50 (s, 0.75H, **H**–C=N), 8.53 (d, 1.5H, *J* = 8 Hz, Ar–**H**), 9.25 (d, 0.5H, *J* = 8 Hz, Ar–**H**), 9.34 (d, 1.5H, *J* = 8 Hz, Ar–**H**), 12.47 (bs, 1H, CON**H**). ^13^C NMR (100 MHz, DMSO-*d*_6_): δ_C_ = 13.70, 13.71 (**C**H_3_), 21.51, 27.47, 27.55 (2×***C***H_2_), 30.20, 30.31 (NCH_2_**C**H_2_), 60.91, 60.99 (N**C**H_2_), 115.76, 115.97, 116.18, 126.15, 127.11, 129.35, 129.44, 129.73, 129.82, 130.20, 130.23, 145.08, 145.68, 147.33, 149.33, 149.63 (Ar–**C**), 158.76, 162.28, 164.75, 165.22 (**C**=N, **C**=O). ^19^F NMR (377 MHz, DMSO-*d*_6_): δ_F_ = (−109.89 to −109.81), (−109.41 to −109.34) (2m, 1F, Ar–**F**). MS (ES) *m*/*z* = 441.10 [M^+^].

*4-(2-(4-Fluorobenzylidene)hydrazinecarbonyl)-1-hexylpyridinium iodide* (***9***). It was obtained as yellow crystals; mp: 227–228 °C. FT-IR (KBr), cm^−1^: $\bar{V}$ = 1625 (C=N), 1695 (C=O), 2883, 2975 (Al–H), 3080 (Ar–H). ^1^H NMR (600 MHz, DMSO-*d*_6_): δ_H_ = 0.88 (t, 3H, *J* = 6 Hz, C**H**_3_), 1.29–1.33 (m, 6H, 3×C**H**_2_), 1.95–1.97 (m, 2H, NCH_2_C**H**_2_), 4.70 (dd, 2H, *J* = 6 Hz, 12 Hz, NC**H**_2_), 7.25 (dd, 0.5H, *J* = 6 Hz, 12 Hz, Ar–**H**), 7.37 (dd, 1.5H, *J* = 6 Hz, 12 Hz, Ar–**H**), 7.62 (dd, 0.5H, *J* = 6 Hz, 12 Hz, Ar–**H**), 7.89 (dd, 1.5H, *J* = 6 Hz, 12 Hz, Ar–**H**), 8.16 (s, 0.25H, **H**–C=N), 8.40 (d, 0.5H, *J* = 6 Hz, Ar–**H**), 8.50 (s, 0.75H, **H**–C=N), 8.53 (d, 1.5H, *J* = 6 Hz, Ar–**H**), 9.25 (d, 0.5H, *J* = 6 Hz, Ar–**H**), 9.33 (d, 1.5H, *J* = 6 Hz, Ar–**H**), 12.47 (s, 0.75H, CON**H**), 12.52 (s, 0.25H, CON**H**). ^13^C NMR (150 MHz, DMSO-*d*_6_): δ_C_ = 14.28, 14.30 (**C**H_3_), 22.32, 25.52, 25.55 (3×**C**H_2_), 31.01, 31.09 (NCH_2_**C**H_2_), 61.43, 61.51 (N**C**H_2_), 116.29, 116.44, 116.51, 116.65, 126.13, 126.65, 127.62, 129.86, 129.91, 130.24, 130.30, 130.70, 130.71, 145.56, 146.18, 147.82, 149.82 (Ar–**C**), 159.27, 163.19, 164.84, 165.72 (**C**=N, **C**=O). ^19^F NMR (377 MHz, DMSO-*d*_6_): δ_F_ = (−109.90 to −109.82), (−109.44 to −109.36) (2m, 1F, Ar–**F**). MS (ES) *m*/*z* = 455.38 [M^+^].

*4-(2-(4-Fluorobenzylidene)hydrazinecarbonyl)-1-heptylpyridinium iodide* (***10***). It was obtained as yellow crystals; mp: 218–219 °C. FT-IR (KBr), cm^−1^: $\bar{V}$ = 1628 (C=N), 1688 (C=O), 2894, 2962 (Al–H), 3075 (A–H). ^1^H NMR (400 MHz, DMSO-*d*_6_): δ_H_ = 0.84–0.88 (m, 3H, C**H**_3_), 1.23–1.31 (m, 8H, 4×C**H**_2_), 1.94–2.00 (m, 2H, NCH_2_C**H**_2_), 4.70 (dd, 2H, *J* = 4 Hz, 8 Hz, NC**H**_2_), 7.22 (t, 0.5H, *J* = 8 Hz, A–**H**), 7.34 (t, 1.5H, *J* = 8 Hz, A–**H**), 7.62 (dd, 0.5H, *J* = 8 Hz, 12 Hz, A–**H**), 7.88 (dd, 1.5H, *J* = 4 Hz, 8 Hz, A–**H**), 8.16 (s, 0.25H, **H**–C=N), 8.40 (d, 0.5H, *J* = 8 Hz, Ar–**H**), 8.50 (s, 0.75H, **H**–C=N), 8.53 (d, 1.5H, *J* = 8 Hz, Ar–**H**), 9.25 (d, 0.5H, *J* = 4 Hz, Ar–**H**), 9.34 (d, 1.5H, *J* = 8 Hz, Ar–**H**), 12.47 (bs, 1H, CON**H**). ^13^C NMR (100 MHz, DMSO-*d*_6_): δ_C_ = 13.86 (**C**H_3_), 21.91, 25.31, 25.36, 27.99, 30.51, 30.63, 30.95, 30.98 (5×**C**H_2_), 60.94, 61.01 (N**C**H_2_), 115.75, 115.95, 116.17, 126.14, 127.11, 129.36, 129.45, 129.72, 129.81, 130.06, 130.21, 130.24, 145.09, 145.67, 147.34, 149.36, 149.63 (Ar–**C**), 158.76, 162.27, 164.75, 165.20 (**C**=N, **C**=O). ^19^F NMR (377 MHz, DMSO-*d*_6_): δ_F_ = (−109.93 to −109.85), (−109.43 to −109.35) (2m, 1F, Ar–**F**). MS (ESI) *m/z* = 469.42 [M^+^].

### 3.4. General Metathesis Procedure for the Synthesis of Specific **11–40**

#### 3.4.1. Conventional Method

A mixture of compounds **5**–**10** (1 mmol) in acetonitrile (8 mL) and potassium hexafluorophosphate, sodium tetrafluoroborate, sodium nitrate, sodium thiocyanate and/or sodium trifluoroacetate (1.2 mmol) was heated under reflux for 16 h. After cooling, the solid formed was filtered and washed with water and/or chloroform to give the desired compounds **11**–**40**.

#### 3.4.2. Ultrasound Method

A mixture of compounds **5**–**10** (1 mmol) in acetonitrile (8 mL) and potassium hexafluorophosphate, sodium tetrafluoroborate, sodium nitrate, sodium thiocyanate and/or sodium trifluoroacetate (1.2 mmol) was irradiated by ultrasound irradiation for 5–6 h at room temperature. The reaction proceeded as described above to afford the same compounds **11**–**40**.

*1-Ethyl-4-(2-(4-fluorobenzylidene)hydrazinecarbonyl)pyridinium hexafluorophosphate* (***11***). It was obtained as yellow crystals; mp: 175–176 °C. ^1^H NMR (400 MHz, DMSO-*d*_6_): δ_H_ = 1.57–1.63 (m, 3H, C**H**_3_), 4.69–4.74 (q, 2H, *J* = 4 Hz, 8 Hz, NC**H**_2_), 7.24 (t, 0.5H, *J* = 8 Hz, Ar–**H**), 7.35 (t, 1.5H, *J* = 8 Hz, Ar–**H**), 7.63 (dd, 0.5H, *J* = 4 Hz, 8 Hz, Ar–**H**), 7.89 (dd, 1.5H, *J* = 4 Hz, 8 Hz, Ar–**H**), 8.16 (s, 0.25H, **H**–C=N), 8.40 (d, 0.5H, *J* = 8 Hz, Ar–**H**), 8.50 (s, 0.75H, **H**–C=N), 8.53 (d, 1.5H, *J* = 8 Hz, Ar–**H**), 9.24 (d, 0.5H, *J* = 4 Hz, Ar–**H**), 9.33 (d, 1.5H, *J* = 8 Hz, Ar–**H**), 12.49 (bs, 1H, CON**H**). ^13^C NMR (100 MHz, DMSO-*d*_6_): δ_C_ = 16.00, 16.12 (**C**H_3_), 56.51, 56.61 (N**C**H_2_), 115.71, 115.89, 116.11, 126.05, 127.08, 129.32, 129.41, 129.66, 129.74, 129.98, 130.16, 130.19, 144.79, 145.09, 145.43, 147.27, 149.32, 149.46 (Ar–**C**), 158.72, 161.91, 162.22, 164.69, 165.13 (**C**=N, **C**=O). ^31^P NMR (162 MHz, DMSO-*d*_6_): δ_P_ = −157.37 to −131.03 (m, 1P, **P**F_6_). ^19^F NMR (377 MHz, DMSO-*d*_6_): δ_F_ = −71.09, −69.20 (2s, 6F, P**F_6_**), (−109.89 to −109.81), (−109.43 to −109.35) (2m, 1F, Ar–**F**). MS (ES) *m*/*z* = 417.95 [M^+^].

*1-Ethyl-4-(2-(4-fluorobenzylidene)hydrazinecarbonyl)pyridinium tetrafluoroborate* (***12***). It was obtained as yellow crystals; mp: 205–206 °C. ^1^H NMR (400 MHz, DMSO-*d*_6_): δ_H_ = 1.57–1.63 (m, 3H, C**H**_3_), 4.69–4.74 (q, 2H, *J* = 4 Hz, 8 Hz, NC**H**_2_), 7.24 (t, 0.5H, *J* = 8 Hz, Ar–**H**), 7.35 (t, 1.5H, *J* = 8 Hz, Ar–**H**), 7.63 (dd, 0.5H, *J* = 4 Hz, 8 Hz, Ar–**H**), 7.89 (dd, 1.5H, *J* = 4 Hz, 8 Hz, Ar–**H**), 8.16 (s, 0.25H, **H**–C=N), 8.40 (d, 0.5H, *J* = 8 Hz, Ar–**H**), 8.50 (s, 0.75H, **H**–C=N), 8.53 (d, 1.5H, *J* = 8 Hz, Ar–**H**), 9.24 (d, 0.5H, *J* = 4 Hz, Ar–**H**), 9.33 (d, 1.5H, *J* = 8 Hz, Ar–**H**), 12.48 (s, 1H, CON**H**). ^13^C NMR (100 MHz, DMSO-*d*_6_): δ_C_ = 16.06, 16.19 (**C**H_3_), 56.57, 56.67 (N**C**H_2_), 115.77, 115.96, 116.18, 126.11, 127.13, 129.39, 129.47, 129.72, 129.81, 130.04, 130.21, 130.24, 144.86, 145.14, 145.50, 147.30, 149.37, 149.51 (Ar–**C**), 158.77, 162.28, 164.76, 165.20 (**C**=N, **C**=O). ^11^B NMR (128 MHz, DMSO-*d*_6_): δ_B_ = −1.29 (d, 1B, **B**F_4_). ^19^F NMR (377 MHz, DMSO-*d*_6_): δ_F_ = (−109.85 to −109.82), (−109.42 to −109.34) (2m, 1F, Ar–**F**), −148.28, −148.29 (2d, 4F, B**F**_4_). MS (ES) *m*/*z* = 359.46 [M^+^].

*1-Ethyl-4-(2-(4-fluorobenzylidene)hydrazinecarbonyl)pyridinium trifluoroacetate* (***13***). It was obtained as yellow crystals; mp: 211–212 °C. ^1^H NMR (400 MHz, DMSO-*d*_6_): δ_H_ = 1.57–1.63 (m, 3H, C**H**_3_), 4.69–4.75 (q, 2H, *J* = 8 Hz, 12 Hz, NC**H**_2_), 7.27 (dd, 0.5H, *J* = 8 Hz, 12 Hz, Ar–**H**), 7.35 (t, 1.5H, *J* = 8 Hz, Ar–**H**), 7.63 (dd, 0.5H, *J* = 4 Hz, 8 Hz, Ar–**H**), 7.89 (dd, 1.5H, *J* = 4 Hz, 8 Hz, Ar–**H**), 8.16 (s, 0.25H, **H**–C=N), 8.40 (d, 0.5H, *J* = 8 Hz, Ar–**H**), 8.51 (s, 0.75H, **H**–C=N), 8.53 (d, 1.5H, *J* = 4 Hz, Ar–**H**), 9.25 (d, 0.5H, *J* = 4 Hz, Ar–**H**), 9.34 (d, 1.5H, *J* = 8 Hz, Ar–**H**), 12.49 (bs, 1H, CON**H**). ^13^C NMR (100 MHz, DMSO-*d*_6_): δ_C_ = 16.00, 16.13 (**C**H_3_), 56.50, 56.59 (N**C**H_2_), 115.71, 115.89, 116.11, 126.03, 127.06, 129.33, 129.41, 129.64, 129.73, 129.97, 130.16, 130.19, 144.80, 145.07, 145.42, 147.29, 149.30, 149.44 (Ar–**C**), 158.73, 161.89, 162.20, 164.67, 165.12 (**C**=N, **C**=O). ^19^F NMR (377 MHz, DMSO-*d*_6_): δ_F_ = −73.49 (s, 3F, C**F_3_**), (−109.89 to −109.82), (−109.45 to −109.37) (2m, 1F, Ar–**F**). MS (ES) *m*/*z* = 385.22 [M^+^].

*1-Ethyl-4-(2-(4-fluorobenzylidene)hydrazinecarbonyl)pyridinium nitrate* (***14***). It was obtained as yellow crystals; mp: 210–211 °C. ^1^H NMR (400 MHz, DMSO-*d*_6_): δ_H_ = 1.57–1.63 (m, 3H, C**H**_3_), 4.69–4.74 (q, 2H, *J* = 8 Hz, 12 Hz, NC**H**_2_), 7.24 (t, 0.5H, *J* = 8 Hz, Ar–**H**), 7.35 (t, 1.5H, *J* = 8 Hz, Ar–**H**), 7.63 (dd, 0.5H, *J* = 4 Hz, 8 Hz, Ar–**H**), 7.89 (dd, 1.5H, *J* = 4 Hz, 8 Hz, Ar–**H**), 8.16 (s, 0.25H, **H**–C=N), 8.40 (d, 0.5H, *J* = 8 Hz, Ar–**H**), 8.51 (s, 0.75H, **H**–C=N), 8.53 (d, 1.5H, *J* = 4 Hz, Ar–**H**), 9.25 (d, 0.5H, *J* = 4 Hz, Ar–**H**), 9.34 (d, 1.5H, *J* = 4 Hz, Ar–**H**), 12.49 (bs, 1H, CON**H**). ^13^C NMR (100 MHz, DMSO-*d*_6_): δ_C_ = 16.00, 16.13 (**C**H_3_), 56.50, 56.59 (N**C**H_2_), 115.71, 115.89, 116.11, 126.03, 127.06, 129.33, 129.41, 126.65, 129.73, 129.97, 130.16, 130.19, 144.81, 145.08, 145.46, 147.24, 149.30, 149.44 (Ar–**C**), 158.72, 161.89, 162.21, 164.36, 164.68, 165.13 (**C**=N, **C**=O). ^19^F NMR (377 MHz, DMSO-*d*_6_): δ_F_ = (−109.89 to −109.81), (−109.43 to −109.35) (2m, 1F, Ar–**F**). MS (ES) *m*/*z* = 334.37 [M^+^].

*1-Ethyl-4-(2-(4-fluorobenzylidene)hydrazinecarbonyl)pyridinium thiocyanate* (***15***). It was obtained as yellow crystals; mp: 203–205 °C. ^1^H NMR (400 MHz, DMSO-*d*_6_): δ_H_ = 1.58–1.63 (m, 3H, C**H**_3_), 4.69–4.75 (q, 2H, *J* = 8 Hz, 12 Hz, NC**H**_2_), 7.24 (t, 0.5H, *J* = 8 Hz, Ar–**H**), 7.35 (t, 1.5H, *J* = 8 Hz, Ar–**H**), 7.63 (dd, 0.5H, *J* = 4 Hz, 8 Hz, Ar–**H**), 7.89 (dd, 1.5H, *J* = 4 Hz, 8 Hz, Ar–**H**), 8.16 (s, 0.25H, **H**–C=N), 8.40 (d, 0.5H, *J* = 8 Hz, Ar–**H**), 8.50 (s, 0.75H, **H**–C=N), 8.53 (d, 1.5H, *J* = 8 Hz, Ar–**H**), 9.24 (d, 0.5H, *J* = 4 Hz, Ar–**H**), 9.33 (d, 1.5H, *J* = 8 Hz, Ar–**H**), 12.49 (bs, 1H, CON**H**). ^13^C NMR (100 MHz, DMSO-*d*_6_): δ_C_ = 16.01, 16.12 (**C**H_3_), 56.52, 56.62 (N**C**H_2_), 115.70, 115.89, 116.11, 126.04, 127.07, 129.32, 129.40, 129.50, 129.65, 129.74, 129.95, 130.15, 130.18, 144.79, 145.09, 145.44, 147.24, 149.31, 149.45 (Ar–**C**), 158.72, 162.21, 164.69, 165.12 (**C**=N, **C**=O). ^19^F NMR (377 MHz, DMSO-*d*_6_) δ_F_ = (−109.89 to −109.81), (−109.43 to −109.35) (2m, 1F, Ar–**F**). MS (ES) *m*/*z* = 330.17 [M^+^].

*4-(2-(4-Fluorobenzylidene)hydrazinecarbonyl)-1-propylpyridinium hexafluorophosphate* (***16***). It was obtained as yellow crystals; mp: 180–181 °C. ^1^H NMR (400 MHz, DMSO-*d*_6_): δ_H_ = 0.89–0.95 (m, 3H, C**H**_3_), 1.94–2.05 (m, 2H, NCH_2_C**H**_2_), 4.67 (dd, 2H, *J* = 4 Hz, 8 Hz, NC**H**_2_), 7.26 (dd, 0.5H, *J* = 8 Hz, 12 Hz, Ar–**H**), 7.37 (dd, 1.5H, *J* = 8 Hz, 12 Hz, Ar–**H**), 7.62 (dd, 0.5H, *J* = 4 Hz, 8 Hz, Ar–**H**), 7.89 (dd, 1.5H, *J* = 4 Hz, 8 Hz, Ar–**H**), 8.16 (s, 0.25H, **H**–C=N), 8.41 (d, 0.5H, *J* = 8 Hz, Ar–**H**), 8.50 (s, 0.75H, **H**–C=N), 8.54 (d, 1.5H, *J* = 8 Hz, Ar–**H**), 9.24 (d, 0.5H, *J* = 8 Hz, Ar–**H**), 9.33 (d, 1.5H, *J* = 8 Hz, Ar–**H**), 12.47 (bs, 1H, CON**H**). ^13^C NMR (100 MHz, DMSO-*d*_6_): δ_C_ = 11.16, 11.19 (**C**H_3_), 25.00, 25.08 (NCH_2_***C***H_2_), 63.22, 63.30 (N**C**H_2_), 116.75, 116.94, 117.16, 127.13, 128.09, 130.33, 130.41, 130.71, 130.79, 130.99, 131.18, 131.21, 146.06, 146.67, 148.34, 150.32, 150.64 (Ar–**C**), 159.75, 163.26, 165.73, 166.19 (**C**=N, **C**=O).^31^P NMR (162 MHz, DMSO-*d*_6_): δ_P_ = −152.98 to −135.42 (m, 1P, **P**F_6_). ^19^F NMR (377 MHz, DMSO-*d*_6_): δ_F_ = −71.08, −69.19, (2s, 6F, P**F_6_**), (−109.89 to −109.83), (−109.41 to −109.33) (2m, 1F, Ar–**F**). MS (ES) *m*/*z* = 431.37 [M^+^].

*4-(2-(4-Fluorobenzylidene)hydrazinecarbonyl)-1-propylpyridinium tetrafluoroborate* (***17***). It was obtained as yellow crystals; mp: 162–163 °C. ^1^H NMR (400 MHz, DMSO-*d*_6_): δ_H_ = 0.90–0.95 (m, 3H, C**H**_3_), 1.94–2.05 (m, 2H, NCH_2_C**H**_2_), 4.65 (t, 2H, *J* = 8 Hz, NC**H**_2_), 7.26 (dd, 0.5H, *J* = 8 Hz, 12 Hz, Ar–**H**), 7.35 (t, 1.5H, *J* = 8 Hz, Ar–**H**), 7.62 (dd, 0.5H, *J* = 4 Hz, 8 Hz, Ar–**H**), 7.89 (dd, 1.5H, *J* = 4 Hz, 8 Hz, Ar–**H**), 8.16 (s, 0.25H, **H**–C=N), 8.41 (d, 0.5H, *J* = 8 Hz, Ar–**H**), 8.50 (s, 0.75H, **H**–C=N), 8.54 (d, 1.5H, *J* = 8 Hz, Ar–**H**), 9.23 (d, 0.5H, *J* = 8 Hz, Ar–**H**), 9.31 (d, 1.5H, *J* = 4 Hz, Ar–**H**), 12.46 (s, 0.75H, CON**H**), 12.50 (s, 0.25H, CON**H**). ^13^C NMR (100 MHz, DMSO-*d*_6_): δ_C_ = 10.18 (**C**H_3_), 24.02, 24.10 (NCH_2_***C***H_2_), 62.26, 62.35 (N**C**H_2_), 115.77, 115.96, 116.18, 126.15, 127.12, 129.34, 129.43, 129.73, 129.82, 130.05, 130.20, 130.23, 145.10, 145.69, 147.39, 149.36 (Ar–**C**), 158.78, 162.29, 164.76, 165.21 (**C**=N, **C**=O). ^11^B NMR (128 MHz, DMSO-*d*_6_): δ_B_ = −1.29 (d, 1B, **B**F_4_). ^19^F NMR (377 MHz, DMSO-*d*_6_): δ_F_ = (−109.89 to −109.80), (−109.41 to −109.33) (2m, 1F, Ar–**F**); −148.29, −148.24 (2d, 4F, B**F**_4_). MS (ESI) *m*/*z* = 373.49 [M^+^].

*4-(2-(4-Fluorobenzylidene)hydrazinecarbonyl)-1-propylpyridinium trifluoroacetate* (***18***). It was obtained as yellow crystals; mp: 151–152 °C. ^1^H NMR (400 MHz, DMSO-*d*_6_): δ_H_ =0.89–0.95 (m, 3H, C**H**_3_), 1.94–2.03 (m, 2H, NCH_2_C**H**_2_), 4.65 (t, 2H, *J* = 8 Hz, NC**H**_2_), 7.24 (t, 0.5H, *J* = 8 Hz, Ar–**H**), 7.38 (dd, 1.5H, *J* = 8 Hz, 12 Hz, Ar–**H**), 7.62 (dd, 0.5H, *J* = 4 Hz, 8 Hz, Ar–**H**), 7.89 (dd, 1.5H, *J* = 4 Hz, 8 Hz, Ar–**H**), 8.16 (s, 0.25H, **H**–C=N), 8.41 (d, 0.5H, *J* = 8 Hz, Ar–**H**), 8.50 (s, 0.75H, **H**–C=N), 8.54 (d, 1.5H, *J* = 8 Hz, Ar–**H**), 9.24 (d, 0.5H, *J* = 8 Hz, Ar–**H**), 9.33 (d, 1.5H, *J* = 8 Hz, Ar–**H**), 12.50 (bs, 1H, CON**H**). ^13^C NMR (100 MHz, DMSO-*d*_6_): δ_C_ = 10.12, 10.15 (**C**H_3_), 23.97, 24.06 (NCH_2_***C***H_2_), 62.15, 62.24 (N**C**H_2_), 115.71, 115.90, 116.12, 126.08, 127.04, 129.27, 129.36, 129.66, 129.74, 129.97, 130.15, 144.99, 145.62, 147.29, 149.23 (Ar–**C**), 158.71, 162.21, 165.17 (**C**=N, **C**=O). ^19^F NMR (377 MHz, DMSO-*d*_6_): δ_F_ = −73.47 (s, 3F, C**F_3_**), (−109.87 to −109.79), (−109.40 to −109.31) (2m, 1F, Ar–**F**). MS (ES) *m*/*z* = 399.00 [M^+^].

*4-(2-(4-Fluorobenzylidene)hydrazinecarbonyl)-1-propylpyridinium nitrate* (***19***). It was obtained as yellow crystals; mp: 172–173 °C. ^1^H NMR (400 MHz, DMSO-*d*_6_): δ_H_ = 0.90–0.95 (m, 3H, C**H**_3_), 1.94–2.05 (m, 2H, NCH_2_C**H**_2_), 4.69 (dd, 2H, *J* = 4 Hz, 8 Hz, NC**H**_2_), 7.25 (dd, 0.5H, *J* = 8 Hz, 12 Hz, Ar–**H**), 7.36 (dd, 1.5H, *J* = 8 Hz, 12 Hz, Ar–**H**), 7.62 (dd, 0.5H, *J* = 4 Hz, 8 Hz, Ar–**H**), 7.89 (dd, 1.5H, *J* = 4 Hz, 8 Hz, Ar–**H**), 8.16 (s, 0.25H, **H**–C=N), 8.40 (d, 0.5H, *J* = 8 Hz, Ar–**H**), 8.52 (s, 0.75H, **H**–C=N), 8.54 (d, 1.5H, *J* = 8 Hz, Ar–**H**), 9.26 (d, 0.5H, *J* = 8 Hz, Ar–**H**), 9.35 (d, 1.5H, *J* = 4 Hz, Ar–**H**), 12.45 (ds, 1H, CON**H**). ^13^C NMR (100 MHz, DMSO-*d*_6_): δ_C_ = 8.49 (**C**H_3_), 22.31, 22.41 (NCH_2_***C***H_2_), 60.58, 60.64 (N**C**H_2_), 114.08, 114.26, 114.48, 124.45, 125.43, 127.69, 127.78, 128.04, 128.13, 128.35, 128.51, 128.54, 143.38, 143.43, 14.00, 145.65, 147.72, 147.96 (Ar–**C**), 157.06, 160.26, 160.59, 162.73, 163.06, 163.46 (**C**=N, **C**=O). ^19^F NMR (377 MHz, DMSO-*d*_6_): δ_F_ = (−109.89 to −109.80), (−109.41 to −109.33) (2m, 1F, Ar–**F**). MS (ES) *m*/*z* = 348.29 [M^+^].

*4-(2-(4-Fluorobenzylidene)hydrazinecarbonyl)-1-propylpyridinium thiocyanate* (***20***). It was obtained as yellow crystals; mp: 160–161 °C. ^1^H NMR (400 MHz, DMSO-*d*_6_): δ_H_ = 0.91–0.96 (m, 3H, C**H**_3_), 1.96–2.05 (m, 2H, NCH_2_C**H**_2_), 4.68 (dd, 2H, *J* = 4 Hz, 8 Hz, NC**H**_2_), 7.24 (t, 0.5H, *J* = 8 Hz, Ar–**H**), 7.35 (t, 1.5H, *J* = 8 Hz, Ar–**H**), 7.63 (dd, 0.5H, *J* = 4 Hz, 8 Hz, Ar–**H**), 7.89 (dd, 1.5H, *J* = 4 Hz, 8 Hz, Ar–**H**), 8.16 (s, 0.25H, **H**–C=N), 8.42 (d, 0.5H, *J* = 8 Hz, Ar–**H**), 8.50 (s, 0.75H, **H**–C=N), 8.55 (d, 1.5H, *J* = 8 Hz, Ar–**H**), 9.24 (d, 0.5H, *J* = 4 Hz, Ar–**H**), 9.32 (d, 1.5H, *J* = 4 Hz, Ar–**H**), 12.50 (bs, 1H, CON**H**). ^13^C NMR (100 MHz, DMSO-*d*_6_): δ_C_ = 10.20 (**C**H_3_), 25.04, 24.12 (NCH_2_***C***H_2_), 62.28, 62.36 (N**C**H_2_), 115.76, 115.95, 116.16, 126.15, 127.13, 129.35, 129.44, 129.65, 129.72, 129.81, 130.02, 130.05, 130.22, 130.25, 145.10, 145.67, 147.40, 149.36, 149.66 (Ar–**C**), 158.79, 161.96, 162.27, 164.42, 164.74, 165.19 (**C**=N, **C**=O). ^19^F NMR (377 MHz, DMSO-*d*_6_): δ_F_ = (−109.89 to −109.83), (−109.41 to −109.33) (2m, 1F, Ar–**F**). MS (ES) *m*/*z* = 344.48 [M^+^].

*1-Butyl-4-(2-(4-fluorobenzylidene)hydrazinecarbonyl)pyridinium hexafluorophosphate* (***21***). It was obtained as yellow crystals; mp: 145–146 °C. ^1^H NMR (400 MHz, DMSO-*d*_6_): δ_H_ = 0.91–0.96 (m, 3H, C**H**_3_), 1.28–1.39 (m, 2H, C**H**_2_CH_3_), 1.91–2.00 (m, 2H, NCH_2_C**H**_2_), 4.70 (dd, 2H, *J* = 4 Hz, 8 Hz, NC**H**_2_), 7.26 (t, 0.5H, *J* = 8 Hz, 12 Hz, Ar–**H**), 7.35 (t, 1.5H, *J* = 8 Hz, Ar–**H**), 7.62 (dd, 0.5H, *J* = 4 Hz, 8 Hz, Ar–**H**), 7.89 (dd, 1.5H, *J* = 8 Hz, 12 Hz, Ar–**H**), 8.15 (s, 0.25H, **H**–C=N), 8.40 (d, 0.5H, *J* = 8 Hz, Ar–**H**), 8.50 (s, 0.75H, **H**–C=N), 8.53 (d, 1.5H, *J* = 8 Hz, Ar–**H**), 9.24 (d, 0.5H, *J* = 4 Hz, Ar–**H**), 9.33 (d, 1.5H, *J* = 8 Hz, Ar–**H**), 12.46 (s, 0.75H, CON**H**), 12.51 (s, 0.25H, CON**H**). ^13^C NMR (100 MHz, DMSO-*d*_6_): δ_C_ = 13.27, 13.30 (**C**H_3_), 18.73, 18.80 (**C**H_2_CH_3_), 32.48, 32.57 (NCH_2_***C***H_2_), 60.79 (N**C**H_2_), 115.77, 115.96, 116.18, 126.16, 127.13, 129.35, 129.44, 129.73, 129.82, 130.05, 130.20, 130.23, 145.08, 145.69, 147.33, 149.34 (Ar–**C**), 158.76, 162.28, 164.76, 165.21 (**C**=N, **C**=O). ^31^P NMR (162 MHz, DMSO-*d*_6_): δ_P_ = −152.98 to −135.42 (m, 1P, **P**F_6_). ^19^F NMR (377 MHz, DMSO-*d*_6_): δ_F_ = −71.09, −69.19 (2s, 6F, P**F_6_**), (−109.88 to −109.84), (−109.41 to −109.33) (2m, 1F, Ar–**F**). MS (ES) *m*/*z* = 445.02 [M^+^].

*1-Butyl-4-(2-(4-fluorobenzylidene)hydrazinecarbonyl)pyridiniumtetrafluoroborate* (***22***). It was obtained as yellow crystals; mp: 172–173 °C. ^1^H NMR (400 MHz, DMSO-*d*_6_): δ_H_ = 0.92–0.97 (m, 3H, C**H**_3_), 1.28–1.39 (m, 2H, C**H**_2_CH_3_), 1.92–2.01 (m, 2H, NCH_2_C**H**_2_), 4.71 (t, 2H, *J* = 8 Hz, NC**H**_2_), 7.27 (dd, 0.5H, *J* = 8 Hz, 12 Hz, Ar–**H**), 7.38 (dd, 1.5H, *J* = 8 Hz, 12 Hz, Ar–**H**), 7.63 (dd, 0.5H, *J* = 4 Hz, 8 Hz, Ar–**H**), 7.90 (dd, 1.5H, *J* = 8 Hz, 12 Hz, Ar–**H**), 8.16 (s, 0.25H, **H**–C=N), 8.41 (d, 0.5H, *J* = 4 Hz, Ar–**H**), 8.51 (s, 0.75H, **H**–C=N), 8.55 (d, 1.5H, *J* = 8 Hz, Ar–**H**), 9.27 (d, 0.5H, *J* = 4 Hz, Ar–**H**), 9.36 (d, 1.5H, *J* = 4 Hz, Ar–**H**), 12.49 (s, 0.75H, CON**H**), 12.53 (s, 0.25H, CON**H**). ^13^C NMR (100 MHz, DMSO-*d*_6_): δ_C_ = 11.76 (**C**H_3_), 17.20, 17.27 (**C**H_2_CH_3_), 30.96, 31.06 (NCH_2_**C**H_2_), 59.19, 59.24 (N**C**H_2_), 114.25, 114.44, 114.66, 124.62, 125.59, 127.84, 127.93, 128.20, 128.29, 128.50, 128.66, 128.69, 143.54, 144.17, 145.75, 147.79, 148.07 (Ar–**C**), 157.23, 160.41, 160.74, 163.21, 164.68 (**C**=N, **C**=O). ^11^B NMR (128 MHz, DMSO-*d*_6_): δ_B_ = −1.29 (d, 1B, **B**F_4_). ^19^F NMR (377 MHz, DMSO-*d*_6_): δ_F_ = (−109.89 to −109.81), (−109.40 to −109.33) (2m, 1F, Ar–**F**); −148.22, −148.16 (2d, 4F, B**F**_4_). MS (ES) *m*/*z* = 387.27 [M^+^].

*1-Butyl-4-(2-(4-fluorobenzylidene)hydrazinecarbonyl)pyridinium trifluoroacetate* (***23***). It was obtained as yellow crystals; mp: 178–179 °C. ^1^H NMR (400 MHz, DMSO-*d*_6_): δ_H_ = 0.91–0.96 (m, 3H, C**H**_3_), 1.28–1.37 (m, 2H, C**H**_2_CH_3_), 1.92–2.01 (m, 2H, NCH_2_C**H**_2_), 4.71 (t, 2H, *J* = 8 Hz, NC**H**_2_), 7.25 (dd, 0.5H, *J* = 8 Hz, 12 Hz, Ar–**H**), 7.36 (dd, 1.5H, *J* = 8 Hz, 12 Hz, Ar–**H**), 7.62 (dd, 0.5H, *J* = 8 Hz, 12 Hz, Ar–**H**), 7.88 (dd, 1.5H, *J* = 4 Hz, 8 Hz, Ar–**H**), 8.16 (s, 0.25H, **H**–C=N), 8.40 (d, 0.5H, *J* = 8 Hz, Ar–**H**), 8.51 (s, 0.75H, **H**–C=N), 8.54 (d, 1.5H, *J* = 8 Hz, Ar–**H**), 9.27 (d, 0.5H, *J* = 8 Hz, Ar–**H**), 9.36 (d, 1.5H, *J* = 8 Hz, Ar–**H**), 12.45 (bs, 1H, CON**H**). ^13^C NMR (100 MHz, DMSO-*d*_6_): δ_C_ = 13.26, 13.28 (**C**H_3_), 18.71, 18.78 (**C**H_2_CH_3_), 32.45, 32.55 (NCH_2_**C**H_2_), 60.75, 60.79 (N**C**H_2_), 115.76, 115.94, 116.16, 126.14, 127.12, 129.38, 129.47, 129.72, 129.81, 130.01, 130.21, 130.24, 145.11, 145.70, 147.31, 149.40, 149.62 (Ar–**C**), 158.93, 161.95, 162.27, 164.42, 164.74, 165.14 (**C**=N, **C**=O). ^19^F NMR (377 MHz, DMSO-*d*_6_): δ_F_ = −73.47 (s, 3F, C**F_3_**), (−109.87 to −109.81), (−109.40 to −109.32) (2m, 1F, Ar–**F**). MS (ESI) *m*/*z* = 414.00 [M^+^ + H].

*1-Butyl-4-(2-(4-fluorobenzylidene)hydrazinecarbonyl)pyridinium nitrate **(24)***. It was obtained as yellow crystals; mp: 170–171 °C. ^1^H NMR (400 MHz, DMSO-*d*_6_): δ_H_ = 0.92–0.97 (m, 3H, C**H**_3_), 1.29–1.38 (m, 2H, C**H**_2_CH_3_), 1.92–2.02 (m, 2H, NCH_2_C**H**_2_), 4.71 (t, 2H, *J* = 8 Hz, NC**H**_2_), 7.27 (dd, 0.5H, *J* = 8 Hz, 12 Hz, Ar–**H**), 7.38 (dd, 1.5H, *J* = 8 Hz, 12 Hz, Ar–**H**), 7.63 (dd, 0.5H, *J* = 4 Hz, 8 Hz, Ar–**H**), 7.90 (dd, 1.5H, *J* = 4 Hz, 8 Hz, Ar–**H**), 8.17 (s, 0.25H, **H**–C=N), 8.41 (d, 0.5H, *J* = 4 Hz, Ar–**H**), 8.52 (s, 0.75H, **H**–C=N), 8.55 (d, 1.5H, *J* = 8 Hz, Ar–**H**), 9.28 (d, 0.5H, *J* = 8 Hz, Ar–**H**), 9.36 (d, 1.5H, *J* = 4 Hz, Ar–**H**), 12.49 (s, 0.75H, CON**H**), 12.53 (s, 0.25H, CON**H**). ^13^C NMR (100 MHz, DMSO-*d*_6_): δ_C_ = 15.03, 15.06 (**C**H_3_), 20.47, 20.53 (**C**H_2_CH_3_), 34.22, 34.32 (NCH_2_**C**H_2_), 62.46, 62.51 (N**C**H_2_), 117.52, 117.71, 117.93, 127.89, 128.86, 131.12, 131.20, 131.47, 131.56, 131.77, 131.93, 131.96, 146.81, 147.44, 149.02, 151.06, 151.34 (Ar–**C**), 160.50, 163.68, 164.01, 166.15, 166.48, 166.94 (**C**=N, **C**=O).^19^F NMR (377 MHz, DMSO-*d*_6_): δ_F_ = (−109.89 to −109.81), (−109.40 to −109.33) (2m, 1F, Ar–**F**). MS (ESI) *m*/*z* = 362.27 [M^+^].

*1-Butyl-4-(2-(4-fluorobenzylidene)hydrazinecarbonyl)pyridinium thiocyanate* (***25***). It was obtained as yellow crystals; mp: 172–173 °C. ^1^H NMR (400 MHz, DMSO-*d*_6_): δ_H_ = 0.92−0.96 (m, 3H, C**H**_3_), 1.28−1.37 (m, 2H, C**H**_2_CH_3_), 1.92−2.01 (m, 2H, NCH_2_C**H**_2_), 4.72 (dd, 2H, *J* = 4 Hz, 8 Hz, NC**H**_2_), 7.24 (t, 0.5H, *J* = 8 Hz, Ar–**H**), 7.38 (dd, 1.5H, *J* = 8 Hz, 12 Hz, Ar–**H**), 7.63 (dd, 0.5H, *J* = 8 Hz, 12 Hz, Ar–**H**), 7.89 (dd, 1.5H, *J* = 4 Hz, 8 Hz, Ar–**H**), 8.16 (s, 0.25H, **H**–C=N), 8.41 (d, 0.5H, *J* = 8 Hz, Ar–**H**), 8.51 (s, 0.75H, **H**–C=N), 8.54 (d, 1.5H, *J* = 4 Hz, Ar–**H**), 9.27 (d, 0.5H, *J* = 8 Hz, Ar–**H**), 9.36 (d, 1.5H, *J* = 8 Hz, Ar–**H**), 12.49 (bs, 1H, CON**H**). ^13^C NMR (100 MHz, DMSO-*d*_6_): δ_C_ = 13.46, 13.48 (**C**H_3_), 18.89, 18.96 (**C**H_2_CH_3_), 32.65, 32.74 (NCH_2_**C**H_2_), 60.88, 60.94 (N**C**H_2_), 115.94, 116.13, 116.35, 126.32, 127.29, 129.53, 129.62, 129.90, 129.99, 130.20, 130.36, 130.39, 145.24, 145.87, 147.46, 149.49, 149.77 (Ar–**C**), 158.93, 162.44, 164.91, 165.38 (**C**=N, **C**=O). ^19^F NMR (377 MHz, DMSO-*d*_6_): δ_F_ = (−109.88 to −109.84), (−109.41 to −109.33) (2m, 1F, Ar–**F**). MS (ES) *m*/*z* = 358.26 [M^+^].

*4-(2-(4-Fluorobenzylidene)hydrazinecarbonyl)-1-pentylpyridinium hexafluorophosphate* (***26***). It was obtained as yellow crystals; mp: 195–196 °C. ^1^H NMR (400 MHz, DMSO-*d*_6_): δ_H_ = 0.86−0.91 (m, 3H, C**H**_3_), 1.23−1.37 (m, 4H, 2×C**H**_2_), 1.93–2.02 (m, 2H, NCH_2_C**H**_2_), 4.69 (dd, 2H, *J* = 4 Hz, 8 Hz, NC**H**_2_), 7.23 (t, 0.5H, *J* = 8 Hz, Ar–**H**), 7.37 (dd, 1.5H, *J* = 8 Hz, 12 Hz, Ar–**H**), 7.62 (dd, 0.5H, *J* = 4 Hz, 8 Hz, Ar–**H**), 7.89 (dd, 1.5H, *J* = 8 Hz, 12 Hz, Ar–**H**), 8.15 (s, 0.25H, **H**–C=N), 8.40 (d, 0.5H, *J* = 8 Hz, Ar–**H**), 8.50 (s, 0.75H, **H**–C=N), 8.53 (d, 1.5H, *J* = 8 Hz, Ar–**H**), 9.24 (d, 0.5H, *J* = 4 Hz, Ar–**H**), 9.33 (d, 1.5H, *J* = 8 Hz, Ar–**H**), 12.46 (s, 0.75H, CON**H**), 12.51 (s, 0.25H, CON**H**). ^13^C NMR (100 MHz, DMSO-*d*_6_): δ_C_ = 13.69 (**C**H_3_), 21.50, 27.47, 27.55 (2×***C***H_2_), 30.20, 30.31 (NCH_2_**C**H_2_), 60.92, 61.00 (N**C**H_2_), 115.76, 115.96, 116.18, 126.16, 127.12, 129.34, 129.43, 129.73, 129.81, 130.20, 130.23, 145.09, 145.69, 147.34, 149.34 (Ar–**C**), 158.76, 162.28, 164.76, 165.22 (**C**=N, **C**=O).^31^P NMR (162 MHz, DMSO-*d*_6_): δ_P_ = −152.98 to −135.42 (m, 1P, **P**F_6_). ^19^F NMR (377 MHz, DMSO-*d*_6_): δ_F_ = −69.21, −71.10 (2s, 6F, P**F_6_**), (−109.89 to −109.81), (−109.41 to −109.34) (2m, 1F, Ar–**F**). MS (ES) *m*/*z* = 459.90 [M^+^].

*4-(2-(4-Fluorobenzylidene)hydrazinecarbonyl)-1-pentylpyridinium tetrafluoroborate* (***27***). It was obtained as yellow crystals; mp: 205–206 °C. ^1^H NMR (400 MHz, DMSO-*d*_6_): δ_H_ = 0.86–0.90 (m, 3H, C**H**_3_), 1.23–1.37 (m, 4H, 2×C**H**_2_), 1.93–2.00 (m, 2H, NCH_2_C**H**_2_), 4.68 (t, 2H, *J* = 4 Hz, 8 Hz, NC**H**_2_), 7.23 (t, 0.5H, *J* = 8 Hz, Ar–**H**), 7.37 (dd, 1.5H, *J* = 8 Hz, 12 Hz, Ar–**H**), 7.62 (dd, 0.5H, *J* = 8 Hz, 12 Hz, Ar–**H**), 7.89 (dd, 1.5H, *J* = 4 Hz, 8 Hz, Ar–**H**), 8.15 (s, 0.25H, **H**–C=N), 8.40 (d, 0.5H, *J* = 8 Hz, Ar–**H**), 8.50 (s, 0.75H, **H**–C=N), 8.53 (d, 1.5H, *J* = 8 Hz, Ar–**H**), 9.25 (d, 0.5H, *J* = 4 Hz, Ar–**H**), 9.34 (d, 1.5H, *J* = 8 Hz, Ar–**H**), 12.47 (bs, 1H, CON**H**). ^13^C NMR (100 MHz, DMSO-*d*_6_): δ_C_ = 13.70 (**C**H_3_), 21.51, 27.47, 27.55 (2×**C**H_2_), 30.20, 30.31 (NCH_2_**C**H_2_), 60.99 (N**C**H_2_), 115.76, 115.96, 116.18, 126.15, 127.11, 129.35, 129.44, 129.73, 129.82, 130.20, 130.23, 145.08, 145.68, 147.32, 149.33, 149.63(Ar–**C**), 158.76, 162.28, 164.75, 165.22 (**C**=N, **C**=O).^11^B NMR (128 MHz, DMSO-*d*_6_): δ_B_ = −1.29 (d, 1B, **B**F_4_). ^19^F NMR (377 MHz, DMSO-*d*_6_): δ_F_ = (−109.89 to −109.81), (−109.42 to −109.34) (2m, 1F, Ar–**F**); −148.28, −148.23 (2d, 4F, B**F**_4_). MS (ES) *m*/*z* = 401.00 [M^+^].

*4-(2-(4-Fluorobenzylidene)hydrazinecarbonyl)-1-pentylpyridinium trifluoroacetate* (***28***). It was obtained as yellow crystals; mp: 201–202 °C. ^1^H NMR (400 MHz, DMSO-*d*_6_): δ_H_ = 0.86–0.90 (m, 3H, C**H**_3_), 1.23–1.37 (m, 4H, 2×C**H**_2_), 1.93–2.00 (m, 2H, NCH_2_C***H***_2_), 4.68 (t, 2H, *J* = 8 Hz, NC**H**_2_), 7.23 (t, 0.5H, *J* = 8 Hz, Ar–**H**), 7.37 (dd, 1.5H, *J* = 8 Hz, 12 Hz, Ar–**H**), 7.62 (dd, 0.5H, *J* = 8 Hz, 12 Hz, Ar–**H**), 7.89 (dd, 1.5H, *J* = 4 Hz, 8 Hz, Ar–**H**), 8.15 (s, 0.25H, **H**–C=N), 8.40 (d, 0.5H, *J* = 8 Hz, Ar–**H**), 8.50 (s, 0.75H, **H**–C=N), 8.53 (d, 1.5H, *J* = 8 Hz, Ar–**H**), 9.25 (d, 0.5H, *J* = 8 Hz, Ar–**H**), 9.34 (d, 1.5H, *J* = 8 Hz, Ar–**H**), 12.48 (bs, 1H, CON**H**). ^13^C NMR (100 MHz, DMSO-*d*_6_): δ_C_ = 13.70, 13.72 (**C**H_3_), 21.51, 27.47, 27.55 (2×**C**H_2_), 30.20, 30.31 (NCH_2_**C**H_2_), 60.92, 60.99 (N**C**H_2_), 115.76, 115.96, 116.18, 126.15, 127.11, 129.35, 129.44, 129.73, 129.81, 130.21, 130.24, 145.08, 145.69, 147.34, 149.33 (Ar–**C**), 158.77, 162.28, 164.75, 165.22 (**C**=N, **C**=O). ^19^F NMR (377 MHz, DMSO-*d*_6_): δ_F_ = −73.50 (s, 3F, C**F_3_**), (−109.87 to −109.83), (−109.42 to −109.35) (2m, 1F, Ar–**F**). MS (ESI) *m*/*z* = 428.00 [M^+^ + H].

*4-(2-(4-Fluorobenzylidene)hydrazinecarbonyl)-1-pentylpyridinium nitrate* (***29***). It was obtained as yellow crystals; mp: 200–201 °C. ^1^H NMR (400 MHz, DMSO-*d*_6_): δ_H_ = 0.86–0.90 (m, 3H, C**H**_3_), 1.23–1.37 (m, 4H, 2×C**H**_2_), 1.93–2.00 (m, 2H, NCH_2_C**H**_2_), 4.68 (t, 2H, *J* = 8 Hz, NC**H**_2_), 7.23 (t, 0.5H, *J* = 8 Hz, Ar–**H**), 7.37 (dd, 1.5H, *J* = 8 Hz, 12 Hz, Ar–**H**), 7.62 (dd, 0.5H, *J* = 8 Hz, 12 Hz, Ar–**H**), 7.89 (dd, 1.5H, *J* = 4 Hz, 8 Hz, Ar–**H**), 8.15 (s, 0.25H, **H**–C=N), 8.40 (d, 0.5H, *J* = 8 Hz, Ar–**H**), 8.50 (s, 0.75H, **H**–C=N), 8.53 (d, 1.5H, *J* = 8 Hz, Ar–**H**), 9.25 (d, 0.5H, *J* = 8 Hz, Ar–**H**), 9.34 (d, 1.5H, *J* = 8 Hz, Ar–**H**), 12.47 (bs, 1H, CON**H**). ^13^C NMR (100 MHz, DMSO-*d*_6_): δ_C_ = 13.70, 1371 (**C**H_3_), 21.51, 24.47, 27.55 (2×***C***H_2_), 30.20, 30.31 (NCH_2_***C***H_2_), 60.91, 60.99 (N**C**H_2_), 115.76, 115.97, 116.19, 126.15, 127.12, 129.35, 129.44, 129.73, 129.82, 130.20, 130.23, 145.08, 145.69, 147.33, 149.33 (Ar–**C**), 158.76, 162.28, 164.75, 165.22 (**C**=N, **C**=O). ^19^F NMR (377 MHz, DMSO-*d*_6_): δ_F_ = (−109.89 to −109.81), (−109.42 to −109.34) (2m, 1F, Ar–**F**). MS (ES) *m*/*z* = 376.70 [M^+^].

*4-(2-(4-fluorobenzylidene)hydrazinecarbonyl)-1-pentylpyridinium thiocyanate* (***30***). It was obtained as yellow crystals; mp: 197–198 °C. ^1^H NMR (400 MHz, DMSO-*d*_6_): δ_H_ = 0.86–0.90 (m, 3H, C**H**_3_), 1.23–1.37 (m, 4H, 2×C**H**_2_), 1.93–2.00 (m, 2H, NCH_2_C**H**_2_), 4.68 (t, 2H, *J* = 8 Hz, NC**H**_2_), 7.23 (t, 0.5H, *J* = 8 Hz, Ar–**H**), 7.37 (dd, 1.5H, *J* = 8 Hz, 12 Hz, Ar–**H**), 7.62 (dd, 0.5H, *J* = 8 Hz, 12 Hz, Ar–**H**), 7.89 (dd, 1.5H, *J* = 4 Hz, 8 Hz, Ar–**H**), 8.15 (s, 0.25H, **H**–C=N), 8.40 (d, 0.5H, *J* = 8 Hz, Ar–**H**), 8.50 (s, 0.75H, **H**–C=N), 8.53 (d, 1.5H, *J* = 8 Hz, Ar–**H**), 9.25 (d, 0.5H, *J* = 8 Hz, Ar–**H**), 9.34 (d, 1.5H, *J* = 8 Hz, Ar–**H**), 12.47 (s, 0.75H, CON**H**), 12.51 (s, 0.25H, CON**H**). ^13^C NMR (100 MHz, DMSO-*d*_6_): δ_C_ = 13.70, 1371 (**C**H_3_), 21.51, 27.47, 27.55 (2×**C**H_2_), 30.20, 30.31 (NCH_2_**C**H_2_), 60.92, 61.00 (N**C**H_2_), 115.76, 115.96, 116.18, 126.15, 127.12, 129.36, 129.44, 129.73, 129.82, 130..02, 130.20, 130.23, 145.08, 145.69, 147.32, 149.34, 149.63(Ar–**C**), 158.76, 162.28, 164.75, 165.21 (**C**=N, **C**=O).^19^F NMR (377 MHz, DMSO-*d*_6_): δ_F_ = (−109.89 to −109.81), (−109.42 to −109.34) (2m, 1F, Ar–**F**). MS (ES) *m*/*z* = 372.00 [M^+^].

*4-(2-(4-Fluorobenzylidene)hydrazinecarbonyl)-1-hexylpyridinium hexafluorophosphate* (***31***). It was obtained as yellow crystals; mp: 143–144 °C. ^1^H NMR (400 MHz, DMSO-*d*_6_): δ_H_ = 0.86–0.93 (m, 3H, C**H**_3_), 1.28–1.34 (m, 6H, 3×C**H**_2_), 1.94–2.00 (m, 2H, NCH_2_C***H***_2_), 4.69 (dd, 2H, *J* = 4 Hz, 8 Hz, NC**H**_2_), 7.25 (dd, 0.5H, *J* = 8 Hz, 12 Hz, Ar–**H**), 7.37 (dd, 1.5H, *J* = 8 Hz, 12 Hz, Ar–**H**), 7.62 (dd, 0.5H, *J* = 4 Hz, 8 Hz, Ar–**H**), 7.89 (dd, 1.5H, *J* = 4 Hz, 8 Hz, Ar–**H**), 8.16 (s, 0.25H, **H**–C=N), 8.40 (d, 0.5H, *J* = 4 Hz, Ar–**H**), 8.50 (s, 0.75H, **H**–C=N), 8.53 (d, 1.5H, *J* = 4 Hz, Ar–**H**), 9.24 (d, 0.5H, *J* = 8 Hz, Ar–**H**), 9.31 (d, 1.5H, *J* = 4 Hz, Ar–**H**), 12.49 (bs, 1H, CON**H**). ^13^C NMR (100 MHz, DMSO-*d*_6_): δ_C_ = 13.74, 13.76 (**C**H_3_), 21.80, 25.01, 25.04, 30.50, 30.58 (4×**C**H_2_), 60.96, 61.04 (N**C**H_2_), 115.73, 115.94, 116.16, 126.15, 127.14, 129.33, 129.42, 129.72, 129.80, 130.06, 130.20, 130.25, 145.04, 145.12, 145.65, 147.40, 149.37, 149.65 (Ar–**C**), 158.77, 161.96, 162.28, 164.43, 164.76, 165.20 (**C**=N, **C**=O). ^31^P NMR (162 MHz, DMSO-*d*_6_) δ_P_ = −157.37 to −131.02 (m, 1P, **P**F_6_). ^19^F NMR (377 MHz, DMSO-*d*_6_): δ_F_ = −71.10, −69.22 (2s, 6F, P**F_6_**), (−109.90 to −109.82), (−109.44 to −109.36) (2m, 1F, Ar–F). MS (ES) *m*/*z* = 473.39 [M^+^].

*4-(2-(4-Fluorobenzylidene)hydrazinecarbonyl)-1-hexylpyridinium tetrafluoroborate* (***32***). It was obtained as yellow crystals; mp: 204–205 °C. ^1^H NMR (400 MHz, DMSO-*d*_6_): δ_H_ = 0.86–0.92 (m, 3H, C**H**_3_), 1.29–1.30 (m, 6H, 3×C**H**_2_), 1.94–2.00 (m, 2H, NCH_2_C**H**_2_), 4.68 (t, 2H, *J* = 8 Hz, NC**H**_2_), 7.23 (t, 0.5H, *J* = 8 Hz, Ar–**H**), 7.35 (t, 1.5H, *J* = 8 Hz, Ar–**H**), 7.62 (dd, 0.5H, *J* = 4 Hz, 8 Hz, Ar–**H**), 7.89 (dd, 1.5H, *J* = 4 Hz, 8 Hz, Ar–**H**), 8.16 (s, 0.25H, **H**–C=N), 8.40 (d, 0.5H, *J* = 4 Hz, Ar–**H**), 8.50 (s, 0.75H, **H**–C=N), 8.54 (d, 1.5H, *J* = 8 Hz, Ar–**H**), 9.25 (d, 0.5H, *J* = 8 Hz, Ar–**H**), 9.33 (d, 1.5H, *J* = 8 Hz, Ar–**H**), 12.48 (bs, 1H, CON**H**). ^13^C NMR (100 MHz, DMSO-*d*_6_): δ_C_ = 13.70, 13.72 (**C**H_3_), 21.75, 24.95, 24.99, 30.40, 30.45, 30.53 (4×**C**H_2_), 60.89, 60.97 (N**C**H_2_), 115.69, 115.89, 116.11, 126.09, 127.07, 129.29, 129.38, 129.66, 129.75, 129.98, 130.17, 130.20, 145.01, 145.61, 147.34, 149.31, 149.59 (Ar–**C**), 158.72, 162.22, 164.70, 165.15 (**C**=N, **C**=O). ^11^B NMR (128 MHz, DMSO-*d*_6_): δ_B_ = −1.28 (d, 1B, **B**F_4_). ^19^F NMR (377 MHz, DMSO-*d*_6_): δ_F_ = (−109.90 to −109.82), (−109.45 to −109.37) (2m, 1F, Ar–**F**); −148.30, −145.25 (2d, 4F, B**F**_4_). MS (ES) *m*/*z* = 415.22 [M^+^].

*4-(2-(4-fluorobenzylidene)hydrazinecarbonyl)-1-hexylpyridinium trifluoroacetate* (***33***). It was obtained as yellow crystals; mp: 214–215 °C. ^1^H NMR (400 MHz, DMSO-*d*_6_): δ_H_ = 0.86–0.92 (m, 3H, C**H**_3_), 1.28–1.30 (m, 6H, 3×C**H**_2_), 1.94–2.00 (m, 2H, NCH_2_C**H**_2_), 4.68 (t, 2H, *J* = 8 Hz, NC**H**_2_), 7.23 (t, 0.5H, *J* = 8 Hz, Ar–**H**), 7.35 (t, 1.5H, *J* = 8 Hz, Ar–**H**), 7.62 (dd, 0.5H, *J* = 4 Hz, 8 Hz, Ar–**H**), 7.89 (dd, 1.5H, *J* = 4 Hz, 8 Hz, Ar–**H**), 8.16 (s, 0.25H, **H**–C=N), 8.40 (d, 0.5H, *J* = 8 Hz, Ar–**H**), 8.50 (s, 0.75H, **H**–C=N), 8.54 (d, 1.5H, *J* = 8 Hz, Ar–**H**), 9.25 (d, 0.5H, *J* = 8 Hz, Ar–**H**), 9.33 (d, 1.5H, *J* = 8 Hz, Ar–**H**), 12.50 (bs, 1H, CON**H**). ^13^C NMR (100 MHz, DMSO-*d*_6_): δ_C_ = 13.76, 13.79 (**C**H_3_), 21.81, 25.01, 25.04, 30.50, 30.59 (4×**C**H_2_), 60.93, 61.00 (N**C**H_2_), 115.75, 115.96, 116.17, 126.14, 127.12, 129.35, 129.43, 129.71, 129.80, 130.03, 130.24, 130.27, 145.09, 145.66, 147.43, 149.34, 149.64 (Ar–**C**), 158.80, 162.27, 164.42, 164.74, 165.22 (**C**=N, **C**=O). ^19^F NMR (377 MHz, DMSO-*d*_6_): δ_F_ = −73.50 (s, 3F, C**F_3_**), (−109.90 to −109.82), (−109.46 to −109.38) (2m, 1F, Ar–**F**). MS (ES) *m*/*z* = 441.18 [M^+^].

*4-(2-(4-Fluorobenzylidene)hydrazinecarbonyl)-1-hexylpyridinium nitrate* (***34***). It was obtained as yellow crystals; mp: 214–215 °C^1^H NMR (400 MHz, DMSO-*d*_6_): δ_H_ = 0.86–0.92 (m, 3H, C**H**_3_), 1.28–1.33 (m, 6H, 3×C**H**_2_), 1.94–2.00 (m, 2H, NCH_2_C**H**_2_), 4.70 (t, 2H, *J* = 8 Hz, NC**H**_2_), 7.26 (dd, 0.5H, *J* = 8 Hz, 12 Hz, Ar–**H**), 7.35 (t, 1.5H, *J* = 8 Hz, Ar–**H**), 7.63 (dd, 0.5H, *J* = 4 Hz, 8 Hz, Ar–**H**), 7.89 (dd, 1.5H, *J* = 4 Hz, 8 Hz, Ar–**H**), 8.17 (s, 0.25H, **H**–C=N), 8.41 (d, 0.5H, *J* = 8 Hz, Ar–**H**), 8.51 (s, 0.75H, **H**–C=N), 8.54 (d, 1.5H, *J* = 4 Hz, Ar–**H**), 9.27 (d, 0.5H, *J* = 8 Hz, Ar–**H**), 9.36 (d, 1.5H, *J* = 8 Hz, Ar–**H**), 12.50 (bs, 1H, CON**H**). ^13^C NMR (100 MHz, DMSO-*d*_6_): δ_C_ = 13.76, 13.78 (**C**H_3_), 21.81, 25.01, 25.04, 30.46, 30.50, 30.59 (4×**C**H_2_), 60.93, 60.99 (N**C**H_2_), 115.75, 115.95, 116.17, 126.14, 127.12, 129.36, 129.45, 129.72, 129.80, 130.05, 130.23, 130.26, 145.08, 145.68, 147.37, 149.35, 149.62 (Ar–**C**), 158.78, 161.94, 162.26, 164.41, 164.73, 165.20 (**C**=N, **C**=O). ^19^F NMR (377 MHz, DMSO-*d*_6_): δ_F_ = (−109.90 to −109.82), (−109.45 to −109.37) (2m, 1F, Ar–**F**). MS (ESI) *m*/*z* = 390.37 [M^+^].

*4-(2-(4-Fluorobenzylidene)hydrazinecarbonyl)-1-hexylpyridinium thiocyanate* (***35***). It was obtained as yellow crystals; mp: 189–190 °C. ^1^H NMR (400 MHz, DMSO-*d*_6_): δ_H_ = 0.86–0.92 (m, 3H, C**H**_3_), 1.28–1.33 (m, 6H, 3×C**H**_2_), 1.94–2.00 (m, 2H, NCH_2_C**H**_2_), 4.70 (dd, 2H, *J* = 4 Hz, 8 Hz, NC**H**_2_), 7.23 (t, 0.5H, *J* = 8 Hz, Ar–**H**), 7.35 (t, 1.5H, *J* = 8 Hz, Ar–**H**), 7.62 (dd, 0.5H, *J* = 4 Hz, 8 Hz, Ar–**H**), 7.89 (dd, 1.5H, *J* = 4 Hz, 8 Hz, Ar–**H**), 8.16 (s, 0.25H, **H**–C=N), 8.41 (d, 0.5H, *J* = 8 Hz, Ar–**H**), 8.50 (s, 0.75H, **H**–C=N), 8.54 (d, 1.5H, *J* = 8 Hz, Ar–**H**), 9.25 (d, 0.5H, *J* = 8 Hz, Ar–**H**), 9.33 (d, 1.5H, *J* = 8 Hz, Ar–**H**), 12.50 (bs, 1H, CON**H**). ^13^C NMR (100 MHz, DMSO-*d*_6_): δ_C_ = 13.70, 13.72 (**C**H_3_), 21.75, 24.96, 24.99, 30.45, 30.54 (4×**C**H_2_), 60.89, 60.97 (N**C**H_2_), 115.69, 115.89, 116.11, 126.09, 127.07,129.29, 129.37, 129.52, 129.75, 130.00, 130.17, 130.20, 145.01, 145.61, 147.32, 149.29, 149.57 (Ar–**C**), 158.72,161.89, 162.21, 164.36, 164.69, 165.15 (**C**=N, **C**=O). ^19^F NMR (377 MHz, DMSO-*d*_6_): δ_F_ = (−109.90 to −109.82), (−109.45 to −109.37) (2m, 1F, Ar–**F**). MS (ES) *m*/*z* = 386.56 [M^+^].

*4-(2-(4-Fluorobenzylidene)hydrazinecarbonyl)-1-heptylpyridinium hexafluorophosphate* (***36***). It was obtained as yellow crystals; mp: 209–210 °C. ^1^H NMR (400 MHz, DMSO-*d*_6_): δ_H_ = 0.85–0.88 (m, 3H, C**H**_3_), 1.25–1.31 (m, 8H, 4×C**H**_2_), 1.91–2.00 (m, 2H, NCH_2_C**H**_2_), 4.69 (t, 2H, *J* = 8 Hz, NC**H**_2_), 7.23 (t, 0.5H, *J* = 8 Hz, Ar–**H**), 7.35 (t, 1.5H, *J* = 8 Hz, Ar–**H**), 7.62 (dd, 0.5H, *J* = 4 Hz, 8 Hz, Ar–**H**), 7.89 (dd, 1.5H, *J* = 4 Hz, 8 Hz, Ar–**H**), 8.16 (s, 0.25H, **H**–C=N), 8.40 (d, 0.5H, *J* = 4 Hz, Ar–**H**), 8.51 (s, 0.75H, **H**–C=N), 8.54 (d, 1.5H, *J* = 8 Hz, Ar–**H**), 9.25 (d, 0.5H, *J* = 4 Hz, Ar–**H**), 9.34 (d, 1.5H, *J* = 8 Hz, Ar–**H**), 12.49 (bs, 1H, CON**H**). ^13^C NMR (100 MHz, DMSO-*d*_6_): δ_C_ = 13.87 (**C**H_3_), 21.91, 25.31, 25.36, 28.00, 30.51, 30.64, 30.96, 30.98 (5×**C**H_2_), 60.94, 61.01 (N**C**H_2_), 115.75, 115.96, 116.18, 126.14, 127.11, 126.36, 129.44, 129.73, 129.81, 130.05, 130.21, 130.24, 145.07, 145.67, 147.33, 149.34, 149.63 (Ar–**C**), 158.77, 162.28, 164.75, 165.22 (**C**=N, **C**=O). ^31^P NMR (162 MHz, DMSO-*d*_6_): δ_P_ = −157.37 to −131.02 (m, 1P, **P**F_6_). ^19^F NMR (377 MHz, DMSO-*d*_6_) δ_F_ = −71.10, −69.21 (2s, 6F, P**F_6_**), (−109.93 to −109.85), (−109.43 to −109.35) (2m, 1F, Ar–**F**). MS (ES) *m*/*z* = 487.37 [M^+^].

*4-(2-(4-fluorobenzylidene)hydrazinecarbonyl)-1-heptylpyridinium tetrafluoroborate* (***37***). It was obtained as yellow crystals; mp: 203–204 °C. ^1^H NMR (400 MHz, DMSO-*d*_6_): δ_H_ = 0.85–0.89 (m, 3H, C**H**_3_), 1.25–1.31 (m, 8H, 4×C**H**_2_), 1.92–2.01 (m, 2H, NCH_2_C**H**_2_), 4.71 (dd, 2H, *J* = 4 Hz, 8 Hz, NC**H**_2_), 7.26 (dd, 0.5H, *J* = 8 Hz, 12 Hz, Ar–**H**), 7.38 (dd, 1.5H, *J* = 8 Hz, 12 Hz, Ar–**H**), 7.63 (dd, 0.5H, *J* = 4 Hz, 8 Hz, Ar–**H**), 7.90 (dd, 1.5H, *J* = 4 Hz, 8 Hz, Ar–**H**), 8.16 (s, 0.25H, **H**–C=N), 8.41 (d, 0.5H, *J* = 8 Hz, Ar–**H**), 8.51 (s, 0.75H, **H**–C=N), 8.54 (d, 1.5H, *J* = 4 Hz, Ar–**H**), 9.27 (d, 0.5H, *J* = 8 Hz, Ar–**H**), 9.35 (d, 1.5H, *J* = 4 Hz, Ar–**H**), 12.49 (s, 0.75H, CON**H**), 12.53 (s, 0.25H, CON**H**). ^13^C NMR (100 MHz, DMSO-*d*_6_): δ_C_ = 14.04 (**C**H_3_), 22.09, 25.47, 25.53, 28.17, 30.68, 30.81, 31.12, 31.15 (5×**C**H_2_), 61.09, 61.16 (N**C**H_2_), 115.92, 116.13, 116.35, 126.31, 127.27, 129.53, 129.62, 129.90, 129.99, 130.18, 130.35, 130.38, 145.23, 145.84, 147.43, 149.49, 149.77 (Ar–**C**), 158.93, 162.09, 162.43, 164.56, 164.91, 165.39 (**C**=N, **C**=O). ^11^B NMR (128 MHz, DMSO-*d*_6_): δ_B_ = −1.14 (d, 1B, **B**F_4_). ^19^F NMR (377 MHz, DMSO-*d*_6_): δ_F_ = (−109.76 to −109.69), (−109.25 to −109.17) (2m, 1F, Ar–**F**); −148.08, −148.03 (2d, 4F, B**F**_4_). MS (ES) *m*/*z* = 429.21 [M^+^].

*4-(2-(4-Fluorobenzylidene)hydrazinecarbonyl)-1-heptylpyridinium trifluoroacetate* (***38***). It was obtained as yellow crystals; mp: 203–204 °C. ^1^H NMR (400 MHz, DMSO-*d*_6_): δ_H_ = 0.85–0.88 (m, 3H, C**H**_3_), 1.23–1.31 (m, 8H, 4×C**H**_2_), 1.94–2.02 (m, 2H, NCH_2_C***H***_2_), 4.69 (dd, 2H, *J* = 4 Hz, 8 Hz, NC**H**_2_), 7.23 (t, 0.5H, *J* = 8 Hz, Ar–**H**), 7.38 (dd, 1.5H, *J* = 8 Hz, 12 Hz, Ar–**H**), 7.62 (dd, 0.5H, *J* = 4 Hz, 8 Hz, Ar–**H**), 7.89 (dd, 1.5H, *J* = 4 Hz, 8 Hz, Ar–**H**), 8.16 (s, 0.25H, **H**–C=N), 8.40 (d, 0.5H, *J* = 8 Hz, Ar–**H**), 8.50 (s, 0.75H, **H**–C=N), 8.53 (d, 1.5H, *J* = 4 Hz, Ar–**H**), 9.25 (d, 0.5H, *J* = 8 Hz, Ar–**H**), 9.33 (d, 1.5H, *J* = 8 Hz, Ar–**H**), 12.50 (bs, 1H, CON**H**). ^13^C NMR (100 MHz, DMSO-*d*_6_): δ_C_ = 12.67 (**C**H_3_), 20.71, 24.11, 24.16, 26.08, 29.31, 29.44, 29.75, 29.78 (5×**C**H_2_), 59.71, 59.78 (N**C**H_2_), 114.54, 114.76, 114.98, 124.94, 125.90, 128.22, 128.51, 128.59, 128.85, 129.01, 129.04, 143.85, 144.46, 146.19, 148.09 (Ar–**C**), 157.59, 161.06, 163.53, 164.04 (**C**=N, **C**=O). ^19^F NMR (377 MHz, DMSO-*d*_6_): δ_F_ = −73.48 (s, 3F, C**F_3_**), (−109.91 to −109.83), (−109.43 to −109.35) (2m, 1F, Ar–**F**). MS (ES) *m*/*z* = 455.90 [M^+^].

*4-(2-(4-Fluorobenzylidene)hydrazinecarbonyl)-1-heptylpyridinium nitrate* (***39***). It was obtained as yellow crystals; mp: 204–205 °C. ^1^H NMR (400 MHz, DMSO-*d*_6_): δ_H_ = 0.85–0.89 (m, 3H, C**H**_3_), 1.24–1.32 (m, 8H, 4×C**H**_2_), 1.94–2.01 (m, 2H, NCH_2_C**H**_2_), 4.70 (t, 2H, *J* = 8 Hz, NC**H**_2_), 7.26 (dd, 0.5H, *J* = 8 Hz, 12 Hz, Ar–**H**), 7.38 (dd, 1.5H, *J* = 8 Hz, 12 Hz, Ar–**H**), 7.63 (dd, 0.5H, *J* = 4 Hz, 8 Hz, Ar–**H**), 7.90 (dd, 1.5H, *J* = 8 Hz, 12 Hz, Ar–**H**), 8.17 (s, 0.25H, **H**–C=N), 8.41 (d, 0.5H, *J* = 8 Hz, Ar–**H**), 8.52 (s, 0.75H, **H**–C=N), 8.55 (d, 1.5H, *J* = 8 Hz, Ar–**H**), 9.27 (d, 0.5H, *J* = 4 Hz, Ar–**H**), 9.36 (d, 1.5H, *J* = 4 Hz, Ar–**H**), 12.50 (bs, 1H, CON**H**). ^13^C NMR (100 MHz, DMSO-*d*_6_): δ_C_ = 14.08 (**C**H_3_), 22.12, 25.51, 25.56, 28.20,30.72, 30.84, 31.16, 31.18 (5×**C**H_2_), 61.12, 61.18 (N**C**H_2_), 115.95, 116.16, 116.37, 126.34, 127.30, 129.57, 129.65, 129.93, 130.01, 130.21, 130.39, 130.42, 145.25, 145.88, 147.47, 149.51, 149.80 (Ar–**C**), 158.95, 162.12, 162.46, 164.93, 165.42 (**C**=N, **C**=O). ^19^F NMR (377 MHz, DMSO-*d*_6_): δ_F_ = (−109.93 to −109.85), (−109.43 to −109.35) (2m, 1F, Ar–**F**). MS (ES) *m*/*z* = 404.90 [M^+^].

*4-(2-(4-Fluorobenzylidene)hydrazinecarbonyl)-1-heptylpyridinium thiocyanate* (***40***). It was obtained as yellow crystals; mp: 175–176 °C. ^1^H NMR (400 MHz, DMSO-*d*_6_): δ_H_ = 0.85–0.89 (m, 3H, C**H**_3_), 1.25–1.32 (m, 8H, 4×C**H**_2_), 1.94–2.01 (m, 2H, NCH_2_C**H**_2_), 4.70 (t, 2H, *J* = 8 Hz, NC**H**_2_), 7.26 (dd, 0.5H, *J* = 8 Hz, 12 H, Ar–**H**), 7.38 (dd, 1.5H, *J* = 8 Hz, 12 Hz, Ar–**H**), 7.63 (dd, 0.5H, *J* = 4 Hz, 8 Hz, Ar–**H**), 7.90 (dd, 1.5H, *J* = 8 Hz, 12 Hz, Ar–**H**), 8.17 (s, 0.25H, **H**–C=N), 8.40 (d, 0.5H, *J* = 4 Hz, Ar–**H**), 8.51 (s, 0.75H, **H**–C=N), 8.54 (d, 1.5H, *J* = 4 Hz, Ar–**H**), 9.27 (d, 0.5H, *J* = 8 Hz, Ar–**H**), 9.35 (d, 1.5H, *J* = 4 Hz, Ar–**H**), 12.51 (bs, 1H, CON**H**). ^13^C NMR (100 MHz, DMSO-*d*_6_): δ_C_ = 14.02 (**C**H_3_), 22.07, 25.47, 28.52, 28.16, 30.69, 30.81, 31.11, 31.14 (5×**C**H_2_), 61.09, 61.17 (N**C**H_2_), 115.89, 116.10, 116.32, 126.30, 127.27, 129.51, 129.60, 129.88, 129.97, 130.16, 130.19, 130.34, 130.37, 145.21, 145.82, 147.42, 149.48, 149.75 (Ar–**C**), 158.91, 162.08, 162.41, 164.55, 164.89, 165.36 (**C**=N, **C**=O). ^19^F NMR (377 MHz, DMSO-*d*_6_): δ_F_ = (−109.93 to −109.85), (−109.43 to −109.35) (2m, 1F, Ar–**F**). MS (ES) *m*/*z* = 400.00 [M^+^].

### 3.5. Biological Activity

#### 3.5.1. Antimicrobial Activity

##### Antimicrobial Evaluation Using the MIC Assay

The *in vitro* minimum inhibitory concentrations (MICs) of the antimicrobial activities were measured by the broth microdilution method [19,20]. The tested pathogenic strains were provided by the Regional Center for Mycology and Biotechnology (RCMB).

##### Cell Lines

The synthesized compounds **5**–**40** were evaluated for their antimicrobial activity against *Streptococcus pneumonia* RCMB 010010, *Bacillus subtilis* RCMB 010067, *Staphylococcus aureus* RCMB 010025 (Gram-positive bacteria), *Pseudomonas aeuroginosa* RCMB 010043, *Escherichia coli* RCMB 010052, *Klebsiella pneumonia* RCMB 010058 (Gram-negative bacteria), *Aspergillus fumigates* RCMB 02568 and *Candida albicans* RCMB 05036 (Fungi). The tested compounds (10 mg) were dissolved in dimethylsulfoxide (DMSO, 1 mL) and then diluted in culture medium (Mueller-Hinton Broth for bacteria and Sabouraud Liquid Medium for fungi) with further progressive dilutions to obtain final concentrations of 1, 2, 4, 8, 16, 31.25, 62.5, 125, 250 and 500 mg·mL^−1^. The DMSO content never exceeded 1% *v*/*v*. The tubes were inoculated with 105 cfu·mL^−1^ (colony forming units/mL) and incubated at 37 °C for 24 h. Growth controls consisting of media and media with DMSO at the same dilutions as used in the experiments were employed.

#### 3.5.2. Anticancer Activity

##### Cell Lines

Human breast adenocarcinoma MCF-7, human ductal breast epithelial tumor T47D, human epithelial carcinoma Hela and human epithelial colorectal adenocarcinoma Caco-2 were cultivated in Dulbecco’s modified Eagle medium (DMEM, Biochrom, Berlin, Germany). All cell lines were cultured at 37 °C, and all media were supplemented with 1% 2 mM l-glutamine (Lonza), 10% fetal calf serum (Gibco, Paisley, UK), 50 IU/mL penicillin/streptomycin (Sigma, St. Louis, MO, USA) and amphotericin B (Sigma, St. Louis, MO, USA). For the cytotoxicity test, each examined compound was added to the culture medium and incubated for 48 h in an atmosphere of 5% CO_2_ and 95% relative humidity at 37 °C.

##### Cytotoxicity Evaluation Using the MTT Assay

The cytotoxic effects associated with the examined compounds were evaluated according to the protocol described in ISO 10993-5 [21]. Cells were seeded at a density of 8 × 10^3^ cells per well in 96-well plates in the appropriate medium. In all assays, the drugs were dissolved in DMSO immediately before the addition to the cell cultures, and an equal amount of solvent was added to control the cells. At the end of the exposure period, the MTT (3-(4,5-dimethylthiazol-2yl)-2,5-diphenyl tetrazolium bromide) (Sigma-Aldrich, Dorset, UK) assay was carried out as previously described (ISO, 2009). The yellow tetrazolium dye of MTT was reduced by metabolically active cells into an intracellular purple formazan product. The absorbance values of each well were determined with a microplate enzyme-linked immuno-assay (ELISA) reader equipped with a 570-nm filter. The survival rates of the controls were set to represent 100% viability. Untreated cultures were used as the control groups.

## 4. Conclusions

The synthesis of fluorinated pyridinium-based hydrazones **5–10** was successfully achieved through the alkylation of *N*-(4-fluorobenzylidene)isonicotinohydrazide (3) with the appropriate alkyl iodides under both conventional and ultrasound conditions. The ultrasound-assisted metathetic synthesis has also been investigated and gave the desired **11–40**. A comparison of the results from using US with that under the classical method revealed an improvement in the reaction yields and a reduction in the reaction time. Most of the synthesized compounds showed good to excellent antibacterial activity at MIC 4–16 µg/ml, whereas only **12**, **23**, **31–33** and **36–38** were found active against fungal strains at MIC 8–16 µg/ml. On the other hand, the tested **5**, **12** and **14** showed significant antiproliferative activity against four different human cancerous cell lines at moderate doses.

## Figures and Tables

**Table 1:** Conventional *versus* ultrasound times and yields of hydrazone **3** and pyridinium salts **5**–**10**.

Compound No.	R	Conventional Method CM		Ultrasound Method US	
		Time (h)	Yield (%)	Time (h)	Yield (%)
**3**	–	1	90	0.5	96
**5**	C_2_H_5_	72	85	12	94
**6**	C_3_H_7_	72	83	12	93
**7**	C_4_H_9_	72	85	14	90
**8**	C_5_H_11_	72	91	14	94
**9**	C_6_H_13_	72	86	14	92
**10**	C_7_H_15_	72	91	14	94

**Table 2:** Conventional *versus* ultrasound times and yields of compounds **11**–**40**.

Compound No.	R	Y	Conventional Method CM		Ultrasound Method US	
			Time (h)	Yield (%)	Time (h)	Yield (%)
**11**	C_2_H_5_	PF_6_	16	94	6	98
**12**	C_2_H_5_	BF_4_	16	85	6	92
**13**	C_2_H_5_	COOCF_3_	16	80	6	90
**14**	C_2_H_5_	NO_3_	16	89	5	98
**15**	C_2_H_5_	SCN	16	98	6	98
**16**	C_3_H_7_	PF_6_	16	93	6	96
**17**	C_3_H_7_	BF_4_	16	81	6	90
**18**	C_3_H_7_	COOCF_3_	16	85	6	94
**19**	C_3_H_7_	NO_3_	16	82	5	92
**20**	C_3_H_7_	SCN	16	85	5	96
**21**	C_4_H_9_	PF_6_	16	88	6	94
**22**	C_4_H_9_	BF_4_	16	83	6	90
**23**	C_4_H_9_	COOCF_3_	16	85	6	92
**24**	C_4_H_9_	NO_3_	16	83	5	90
**25**	C_4_H_9_	SCN	16	83	5	92
**26**	C_5_H_11_	PF_6_	16	86	6	94
**27**	C_5_H_11_	BF_4_	16	90	6	98
**28**	C_5_H_11_	COOCF_3_	16	80	6	92
**29**	C_5_H_11_	NO_3_	16	94	5	98
**30**	C_5_H_11_	SCN	16	96	5	98
**31**	C_6_H_13_	PF_6_	16	91	6	98
**32**	C_6_H_13_	BF_4_	16	98	6	98
**33**	C_6_H_13_	COOCF_3_	16	89	6	92
**34**	C_6_H_13_	NO_3_	16	87	5	94
**35**	C_6_H_13_	SCN	16	98	5	98
**36**	C_7_H_15_	PF_6_	16	98	6	98
**37**	C_7_H_15_	BF_4_	16	98	6	98
**38**	C_7_H_15_	COOCF_3_	16	80	6	88
**39**	C_7_H_15_	NO_3_	16	83	6	88
**40**	C_7_H_15_	SCN	16	80	6	86

**Table 3:** Antimicrobial activity expressed as MIC (μg/mL).

Compound No.	Gram-Positive Organisms			Gram-Negative Organisms			Fungi	
	*Sp*	*Bs*	*Sa*	*Pa*	*Ec*	*Kp*	*Af*	*Ca*
**5**	16	16	16	16	16	16	125	125
**7**	16	16	16	16	16	16	62.5	125
**9**	8	8	16	16	8	8	62.5	62.5
**10**	8	8	8	16	8	8	62.5	62.5
**11**	8	4	4	8	8	8	31.25	31.25
**12**	8	4	8	4	8	8	16	16
**13**	4	4	8	16	16	16	31.25	31.25
**14**	16	16	16	16	16	16	125	125
**15**	16	16	16	16	16	16	250	250
**21**	8	4	4	8	8	8	31.25	31.25
**22**	8	4	4	4	8	8	31.25	31.25
**23**	4	4	8	8	8	8	16	16
**24**	16	16	16	16	16	16	125	125
**25**	16	16	16	16	16	16	125	125
**31**	8	8	4	8	8	8	16	16
**32**	4	4	4	8	4	4	16	16
**33**	4	4	4	4	4	4	8	8
**34**	8	8	8	8	4	8	125	125
**35**	8	8	8	8	4	8	125	125
**36**	4	4	4	4	4	4	16	16
**37**	4	4	4	4	4	4	8	8
**38**	4	4	4	4	4	4	8	8
**39**	4	4	8	4	4	8	125	125
**40**	4	4	8	4	4	8	125	125
**Ciprofloxacin**	≤5	≤1	≤5	≤5	≤1	≤1	–	–
**Fluconazole**	–	–	–	–	–	–	≤1	≤1

**Table 4:** LD_50_ values (ng/µL) of the examined compounds on four human cancer cell lines. Values are expressed as the mean ± SD of three experiments.

Compound No.	MCF-7	T47D	HeLa	Caco-2
**5**	286 ± 8	278 ± 10	292 ± 9	301 ± 11
**11**	Not active	Not active	Not active	Not active
**12**	512 ± 11	498 ± 6	503 ± 8	528 ± 10
**13**	Not active	Not active	Not active	Not active
**14**	465 ± 8	486 ± 13	471 ± 15	463 ± 9
**15**	Not active	Not active	Not active	Not active

Human ductal breast epithelial tumor **T47D**; Human breast adenocarcinoma **MCF-7**; Human epithelial carcinoma **HeLa**; Human epithelial colorectal adenocarcinoma **Caco-2**.

## Supplementary Materials

Supplementary materials can be found at http://www.mdpi.com/1422-0067/17/5/766/s1.
