# Supplementary material for: An Eco-Friendly Ultrasound-Assisted Synthesis of Novel Fluorinated Pyridinium Salts-Based Hydrazones and Antimicrobial and Antitumor Screening
Source: Int J Mol Sci. 2016 May 21;17(5):766. doi: 10.3390/ijms17050766 (PMC4881586; doi:10.3390/ijms17050766)
Supplement: Supplementary file 1 [file ijms-17-00766-s001.pdf]

Supplementary Materials: An Eco-Friendly Ultrasound-Assisted Synthesis of Novel Fluorinated Pyridinium Salts-Based Hydrazones and Antimicrobial and Antitumor Screening

Nadjet Rezki, Salsabeel A. Al-Sodies, Mohamed R. Aouad, Sanaa Bardaweel, Mouslim Messali and El Sayed H. El Ashry

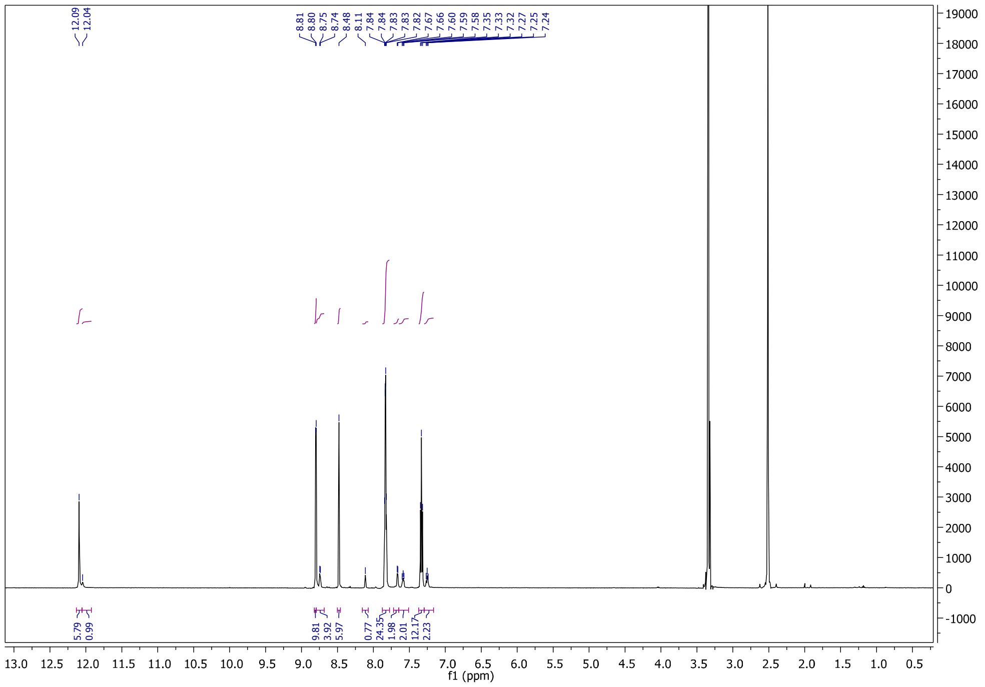

Figure S1. ^{1}H NMR of Compound **3**.

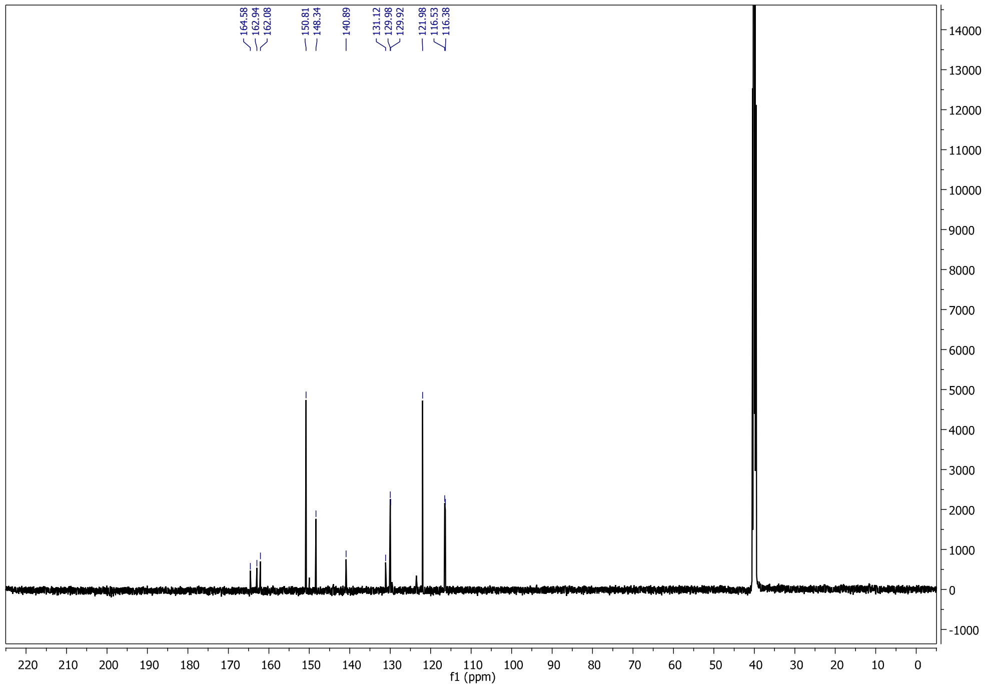

Figure S2. ^{13}C NMR of Compound **3**.

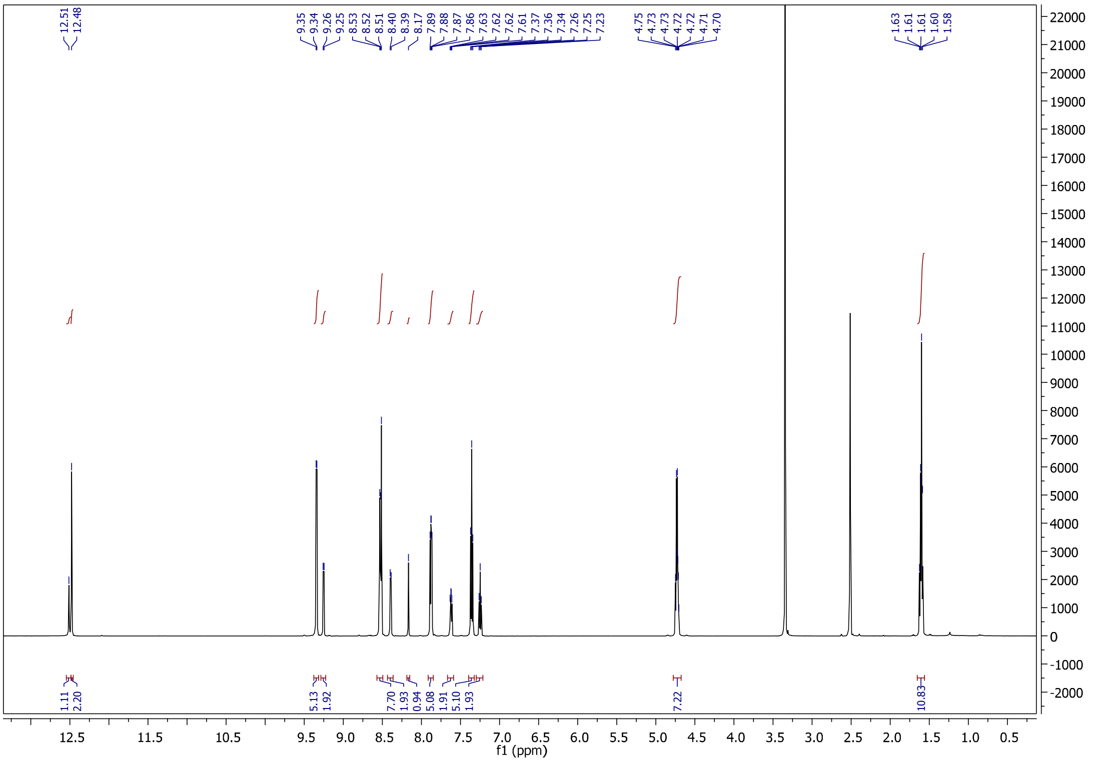

Figure S3. ^{1}H NMR of Compound **5.**

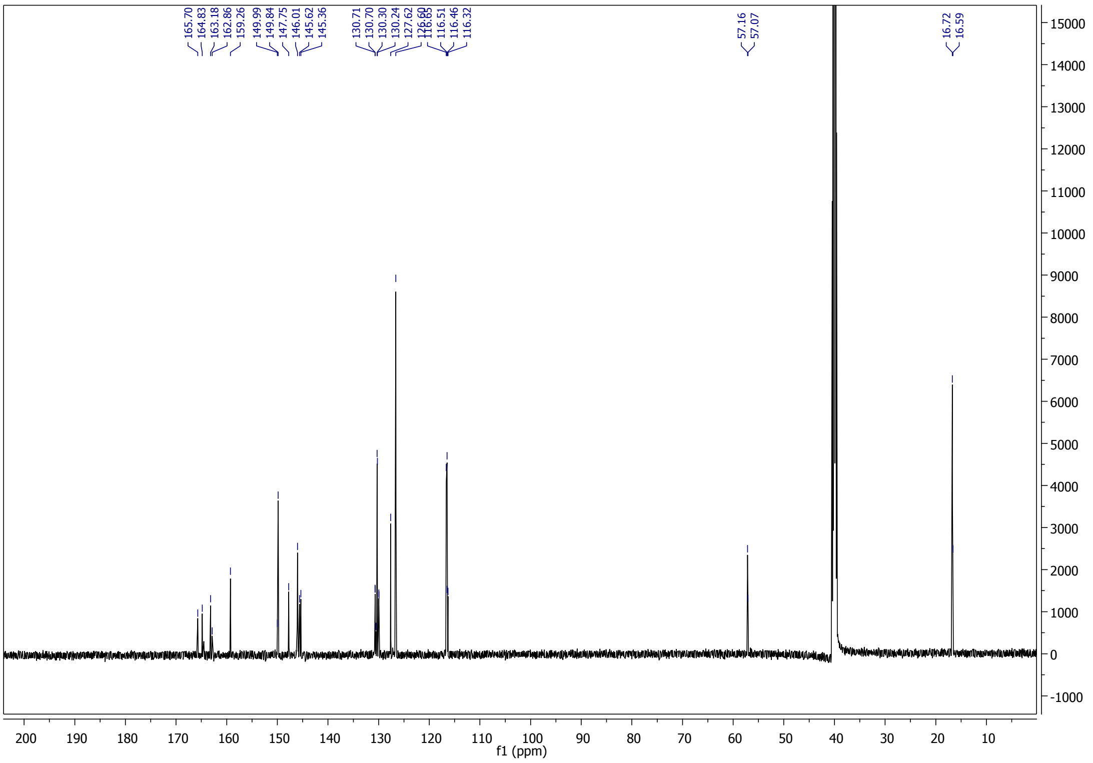

Figure S4. ^{13}C NMR of Compound **5**.

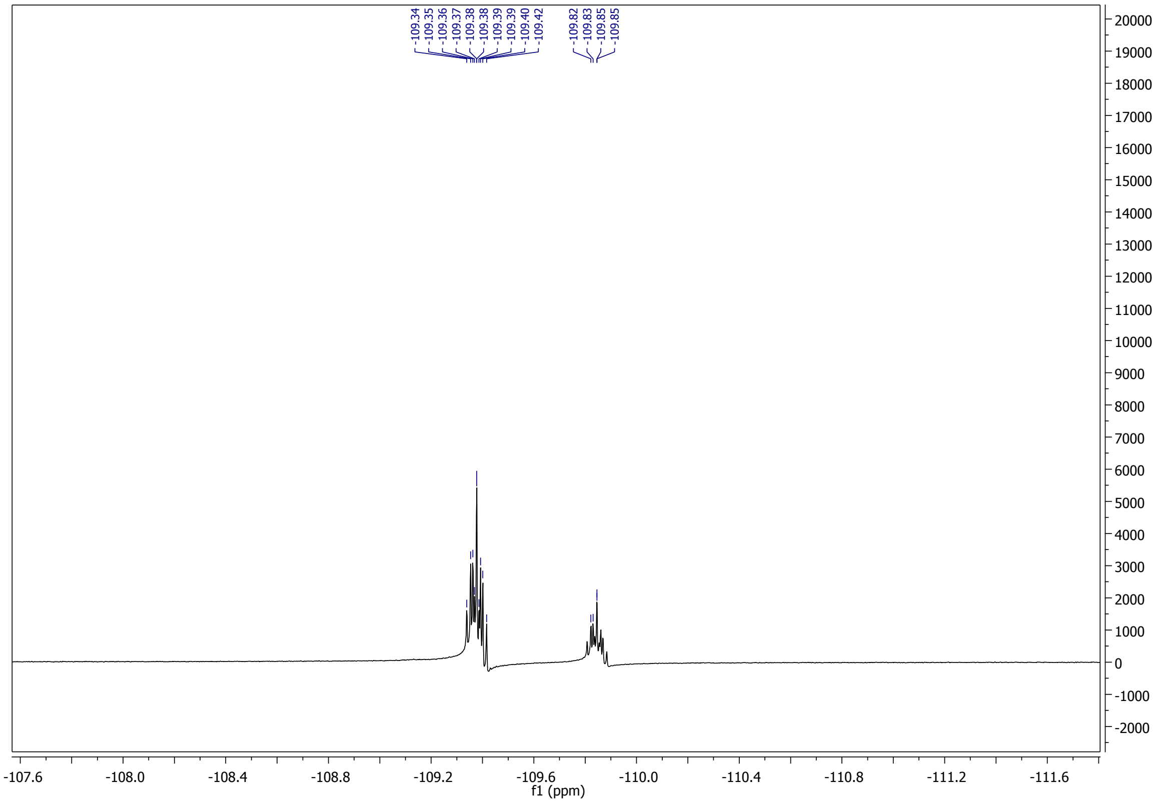

Figure S5. ^{19}F NMR of Compound **5**.

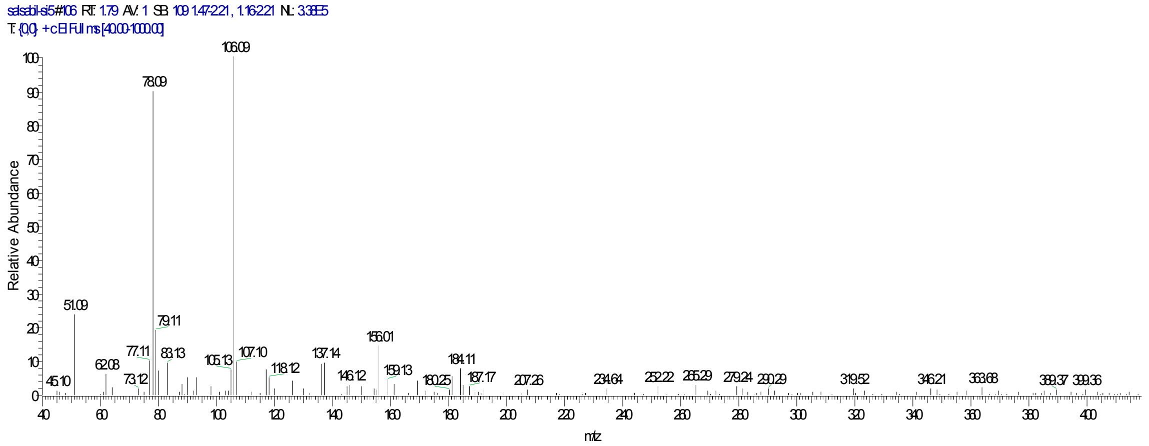

Figure S6. MS (ESI) of Compound **5**.

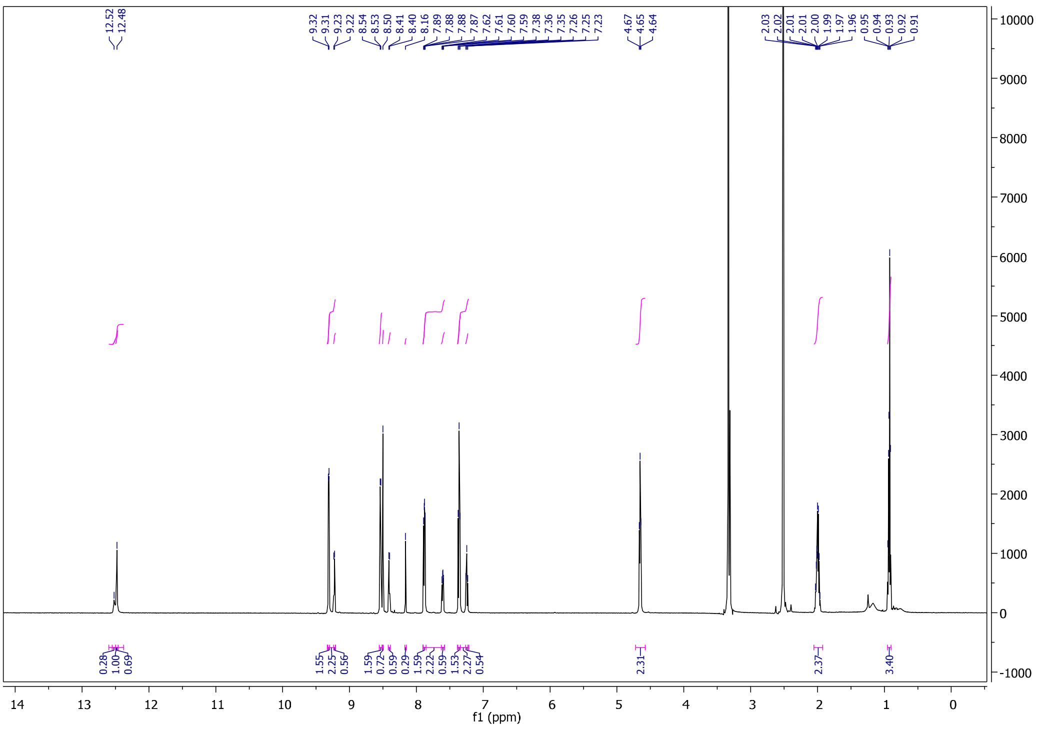

Figure S7. ^{1}H NMR of Compound **6**.

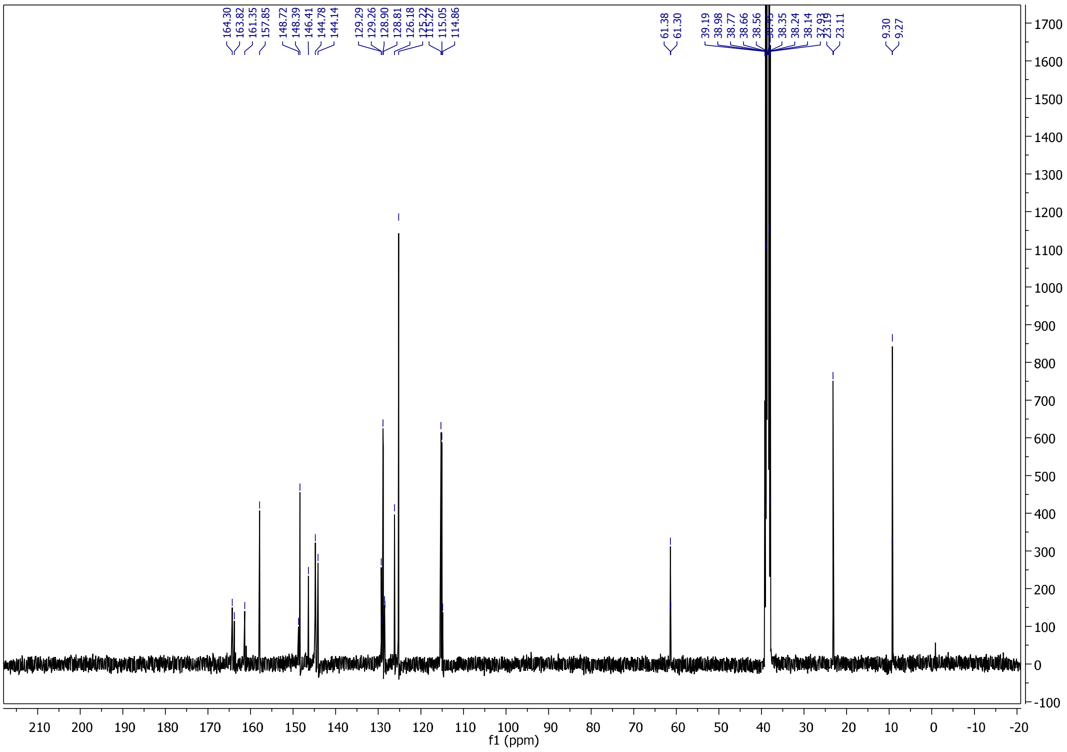

Figure S8. ^{13}C NMR of Compound **6**.

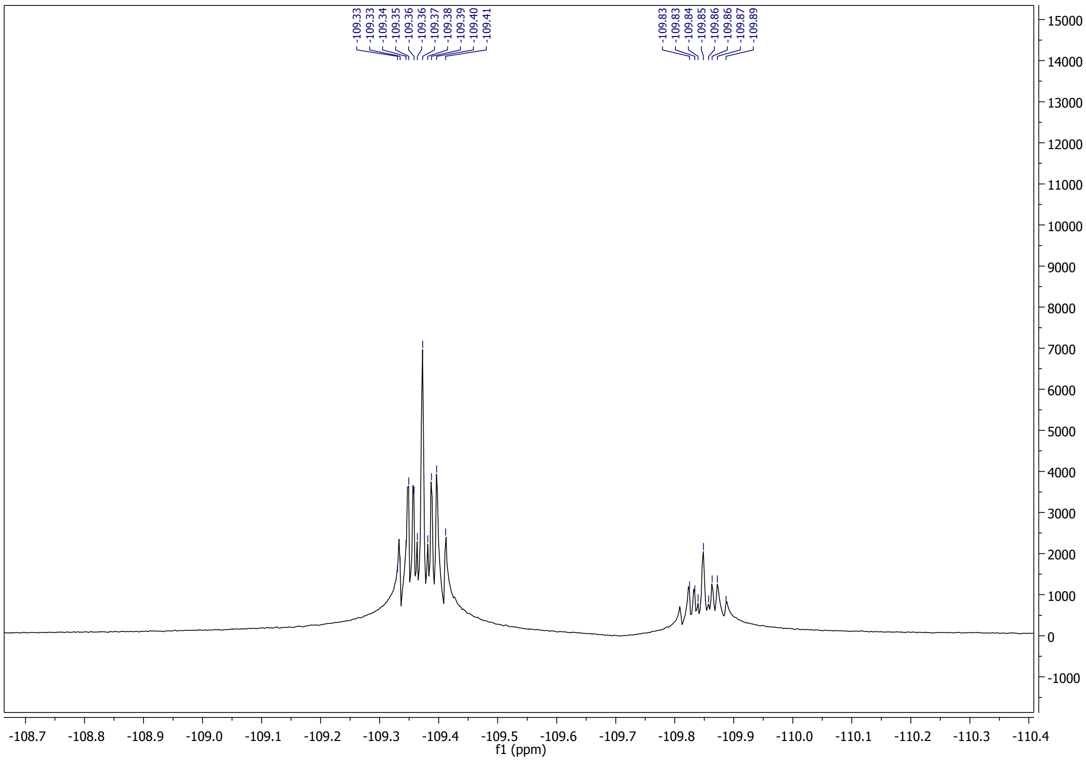

Figure S9. ^{19}F NMR of Compound **6**.

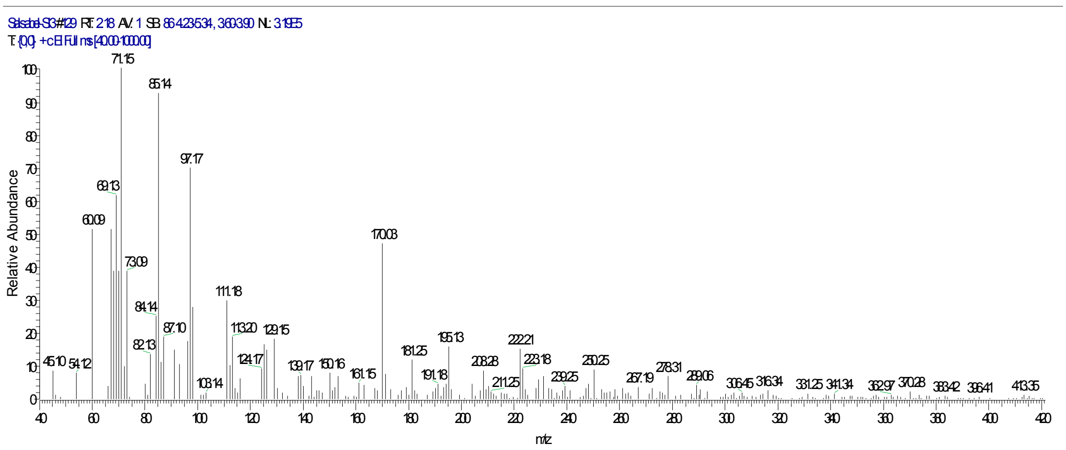

Figure S10. MS (ESI) of Compound **6**.

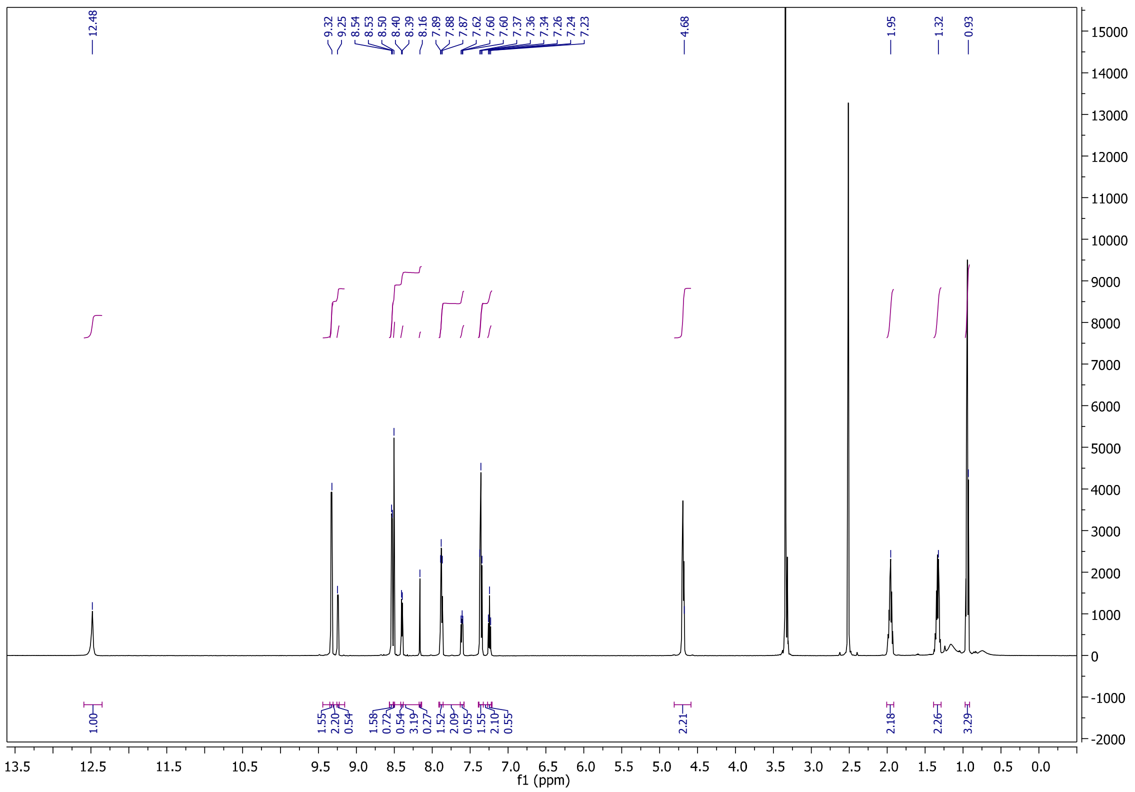

Figure S11. ^{1}H NMR of Compound **7**.

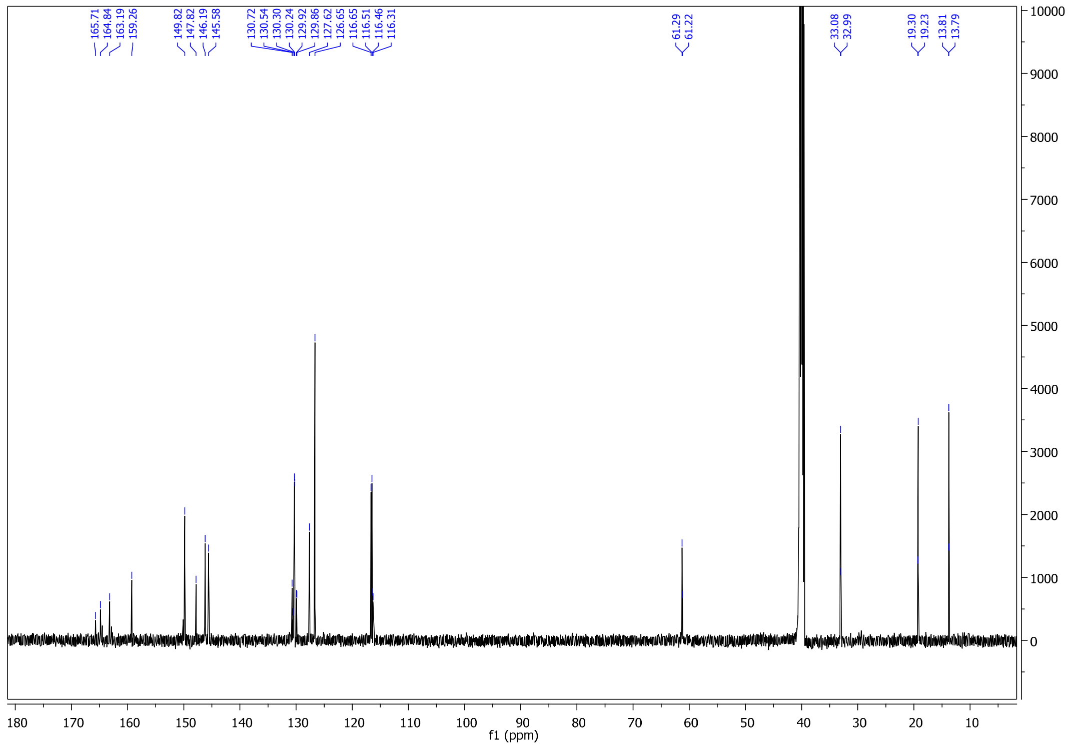

Figure S12. ^{13}C NMR of Compound **7**.

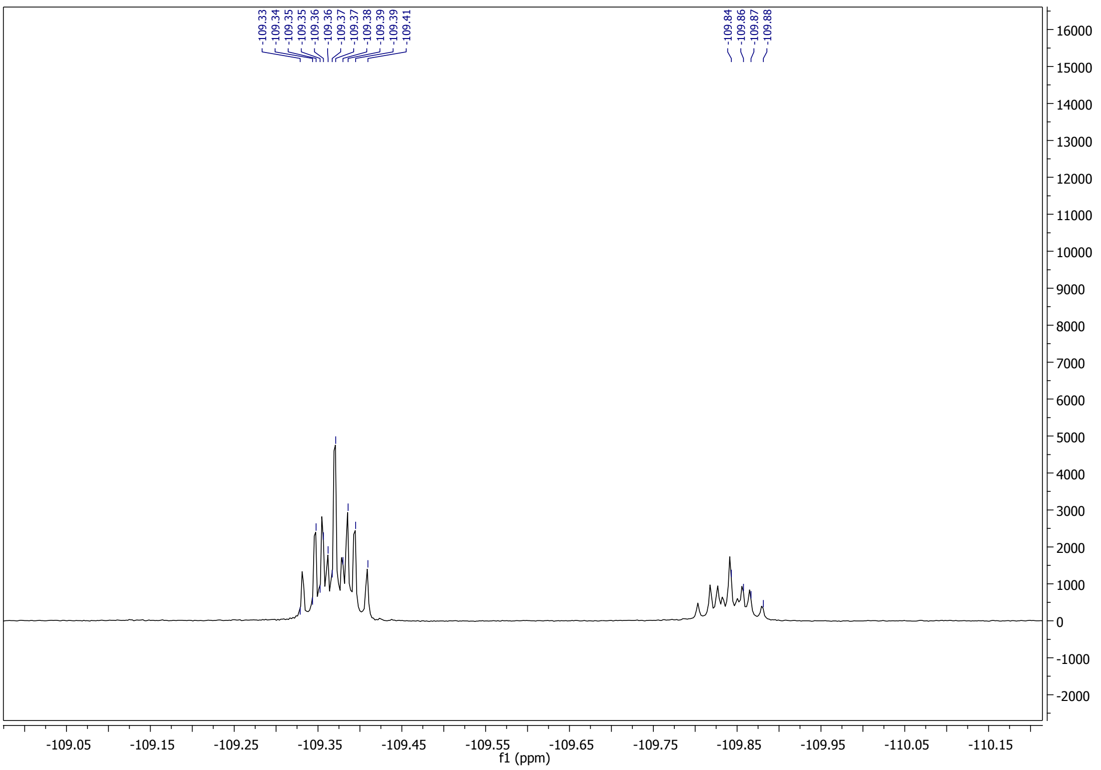

Figure S13. ^{19}F NMR of Compound **7**.

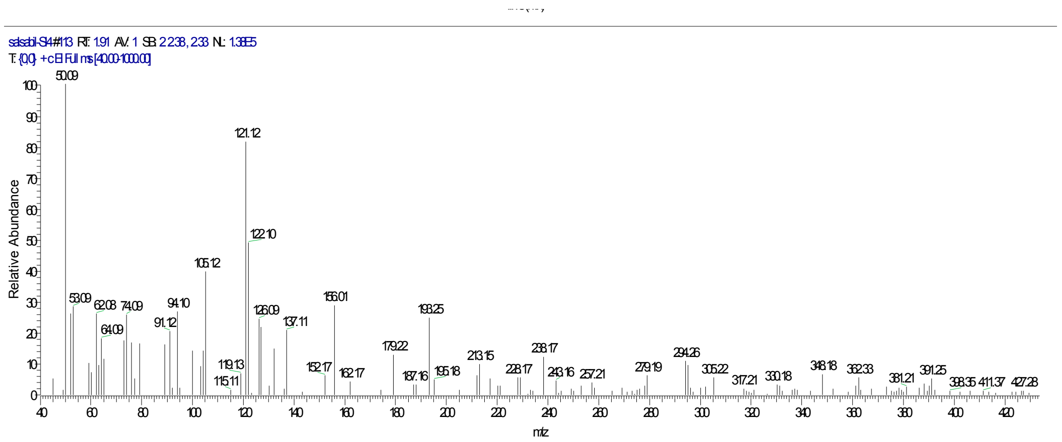

Figure S14. MS (ESI) of Compound **7**.

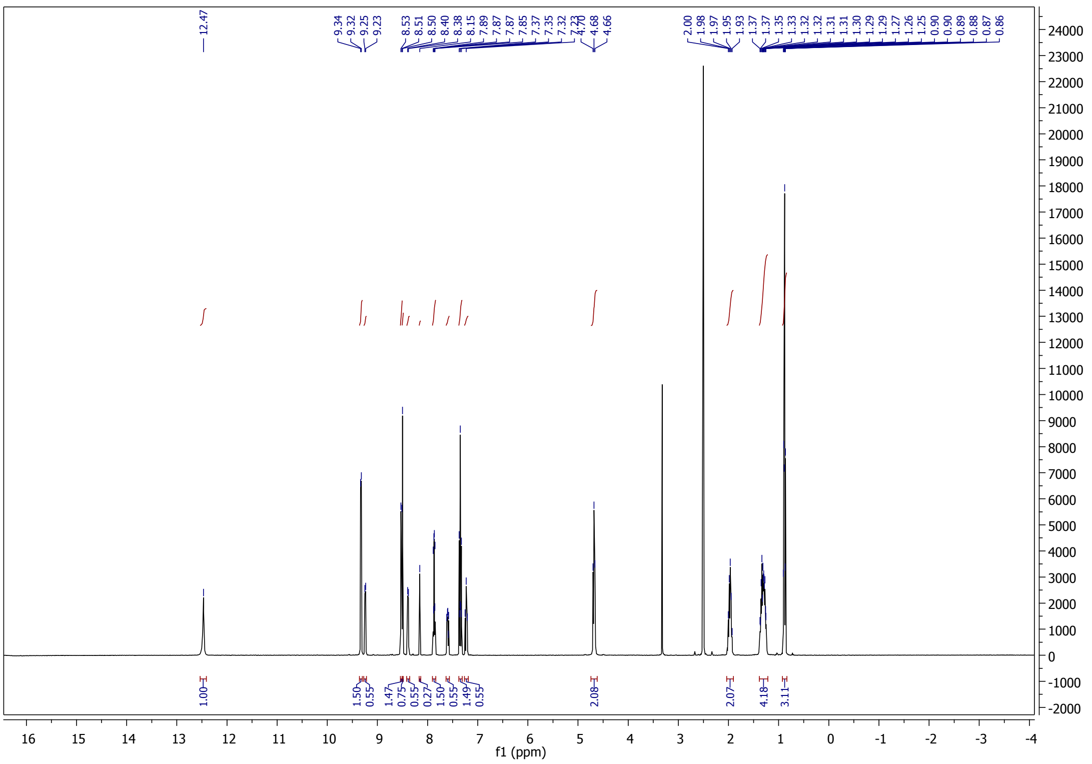

Figure S15. ^{1}H NMR of Compound **8**.

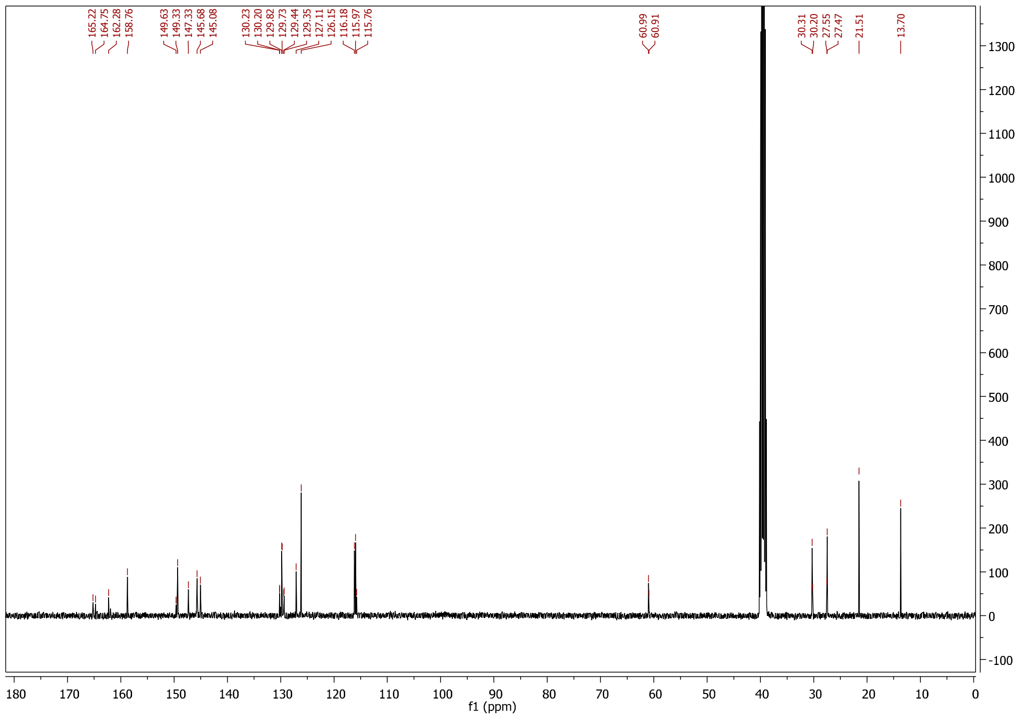

Figure S16. ^{13}C NMR of Compound **8**.

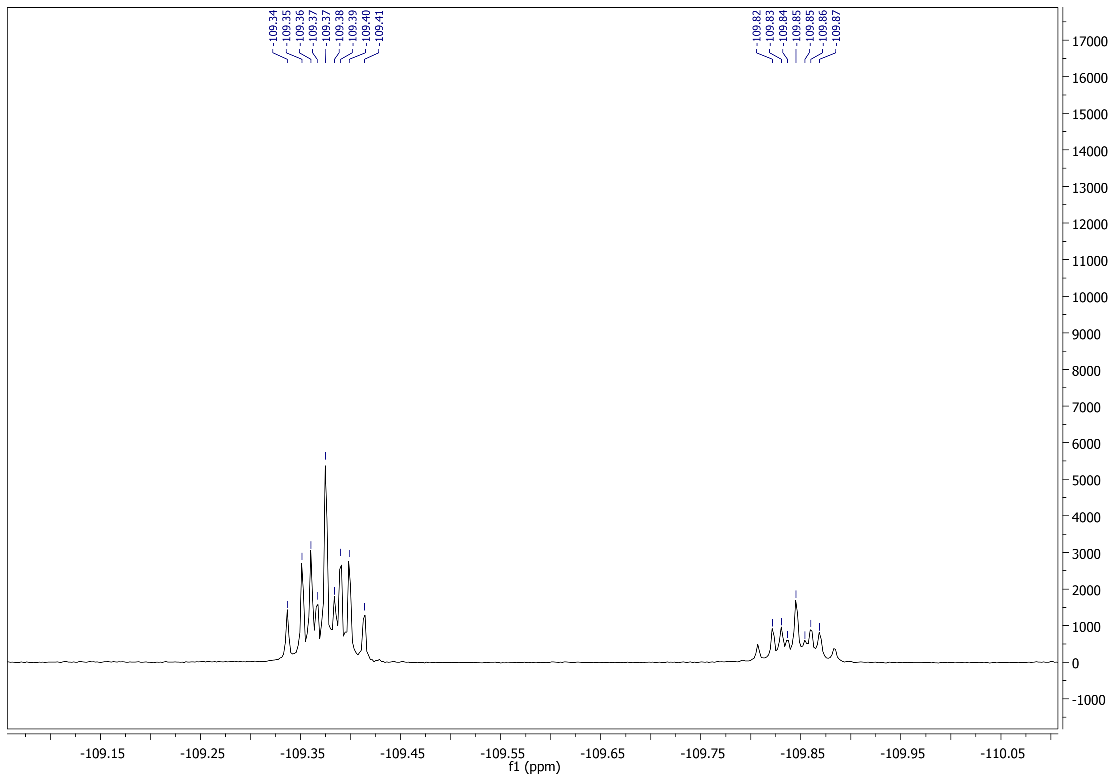

Figure S17. ^{19}F NMR of Compound **8**.

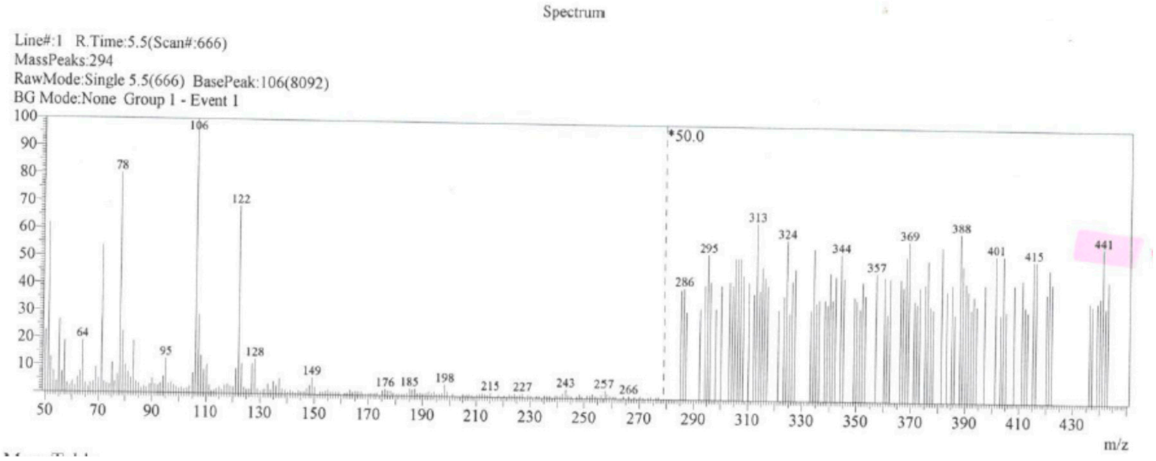

Figure S18. MS (ESI) of Compound **8**.

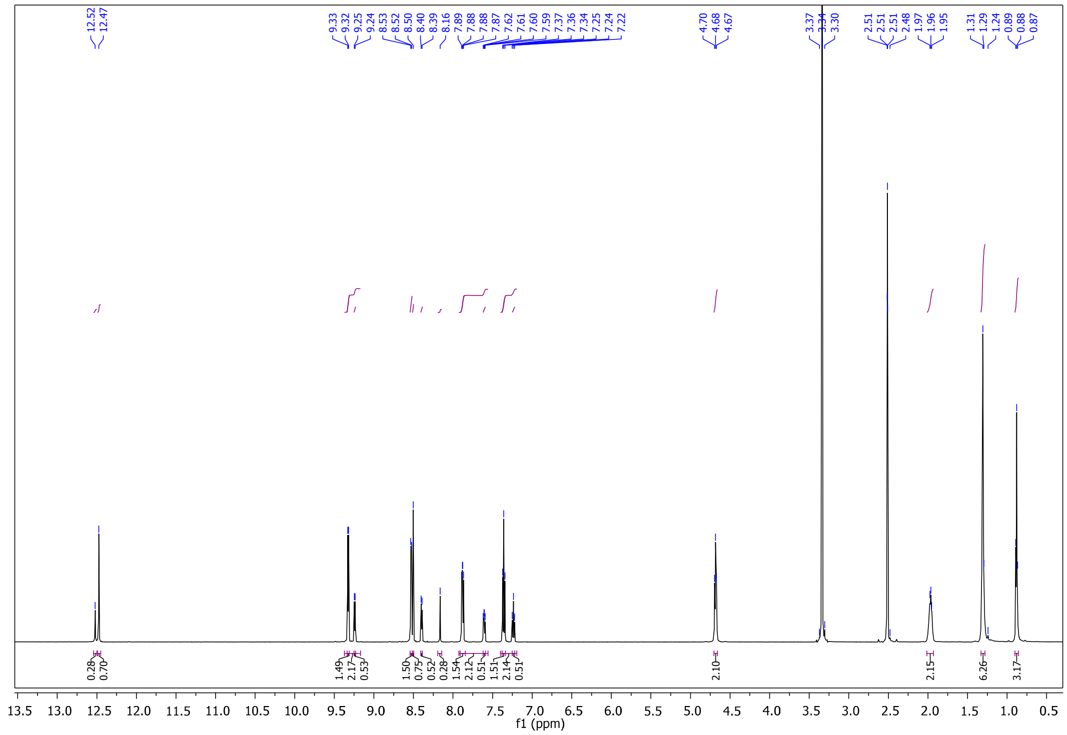

Figure S19. ^{1}H NMR of Compound **9**.

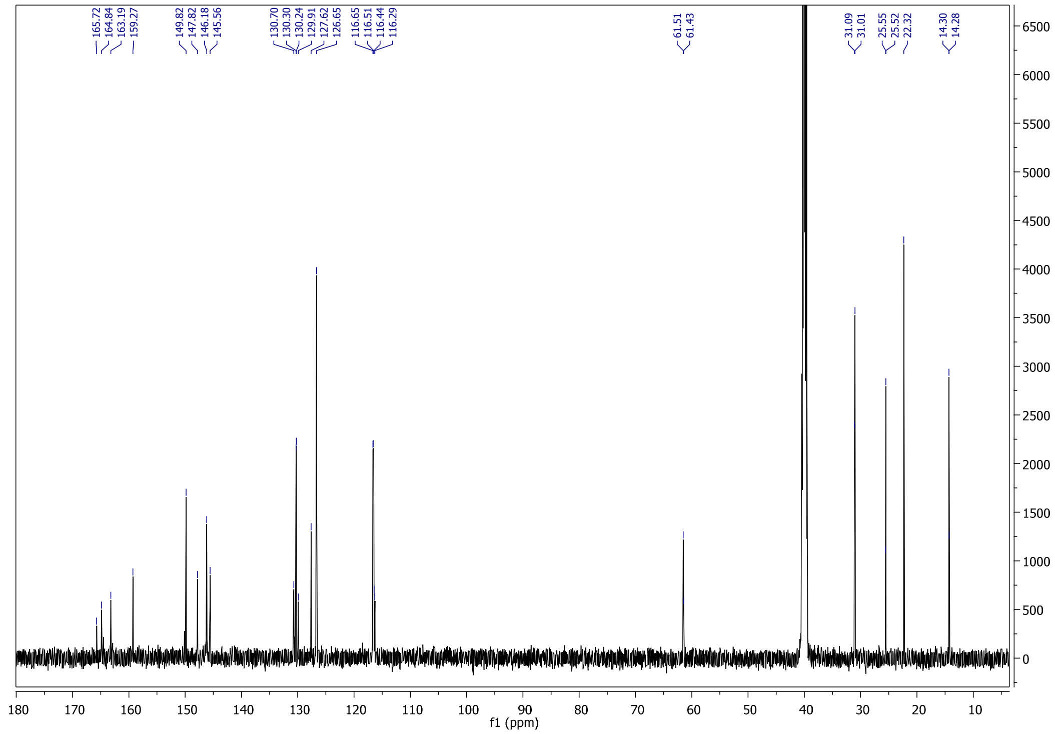

Figure S20. ^{13}C NMR of Compound **9**.

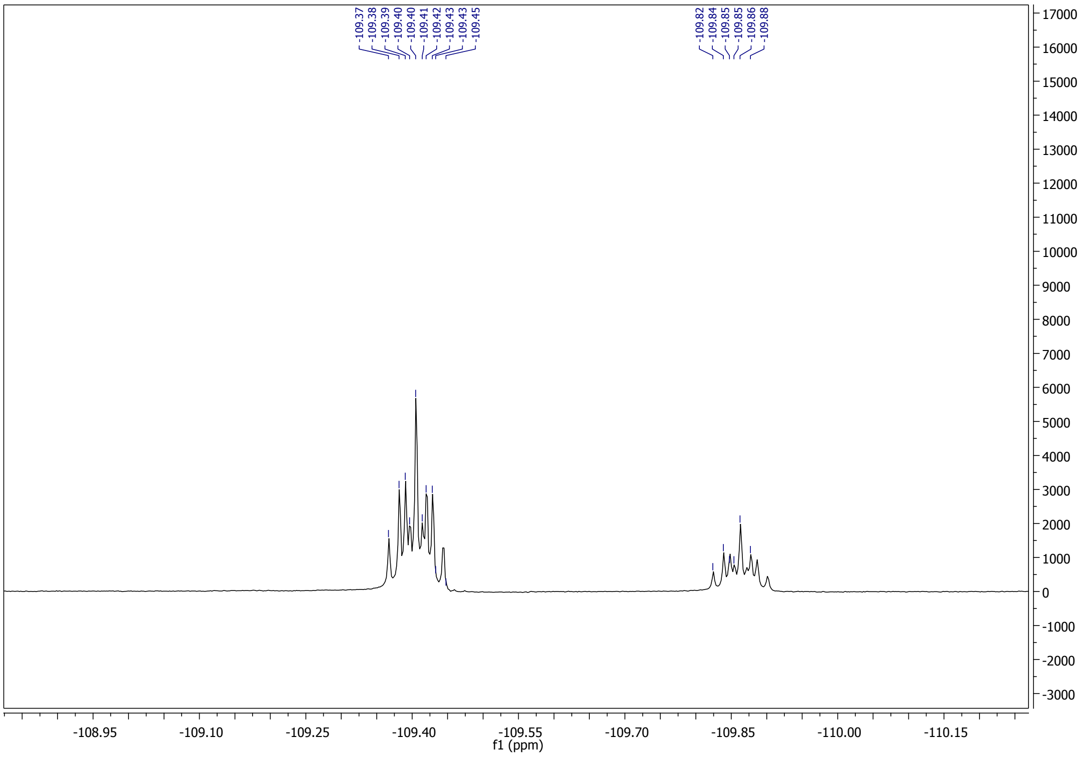

Figure S21. ^{19}F NMR of Compound **9**.

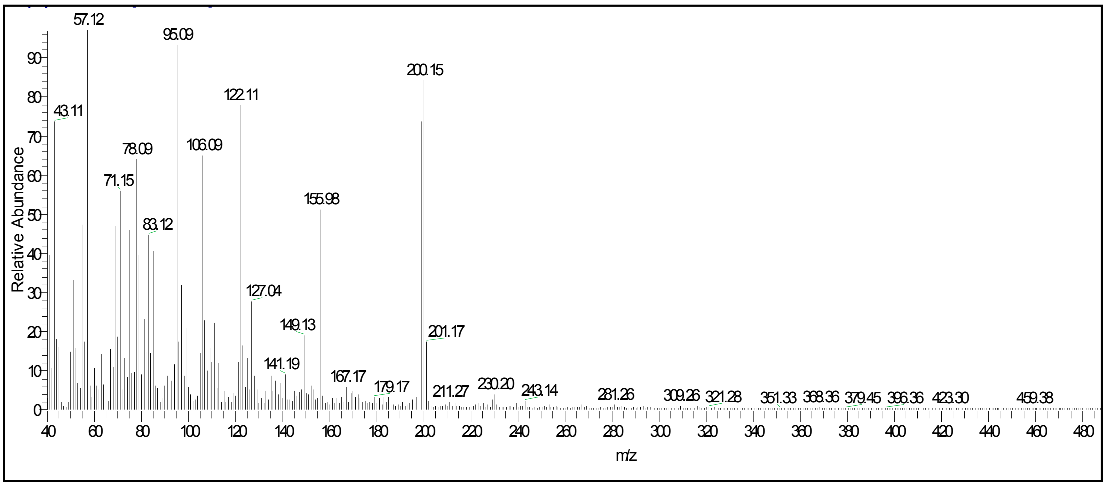

Figure S22. MS (ESI) of Compound **9**.

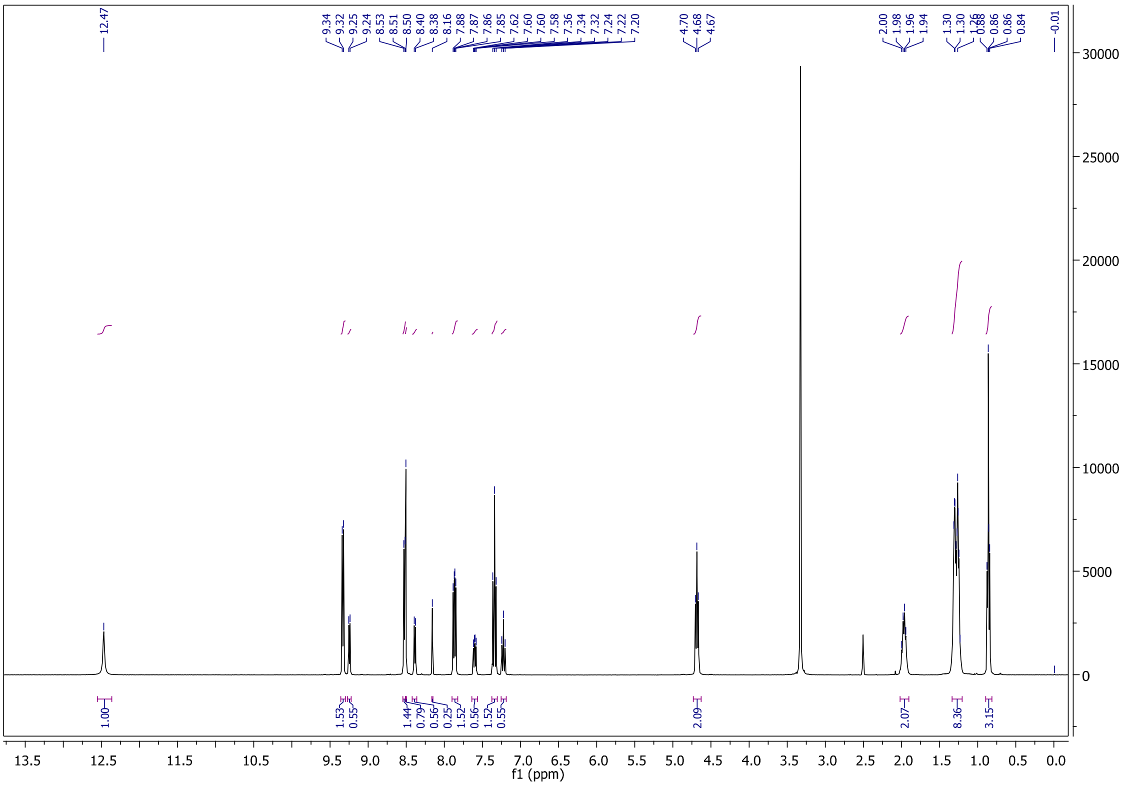

Figure S23. ^{1}H NMR of Compound **10**.

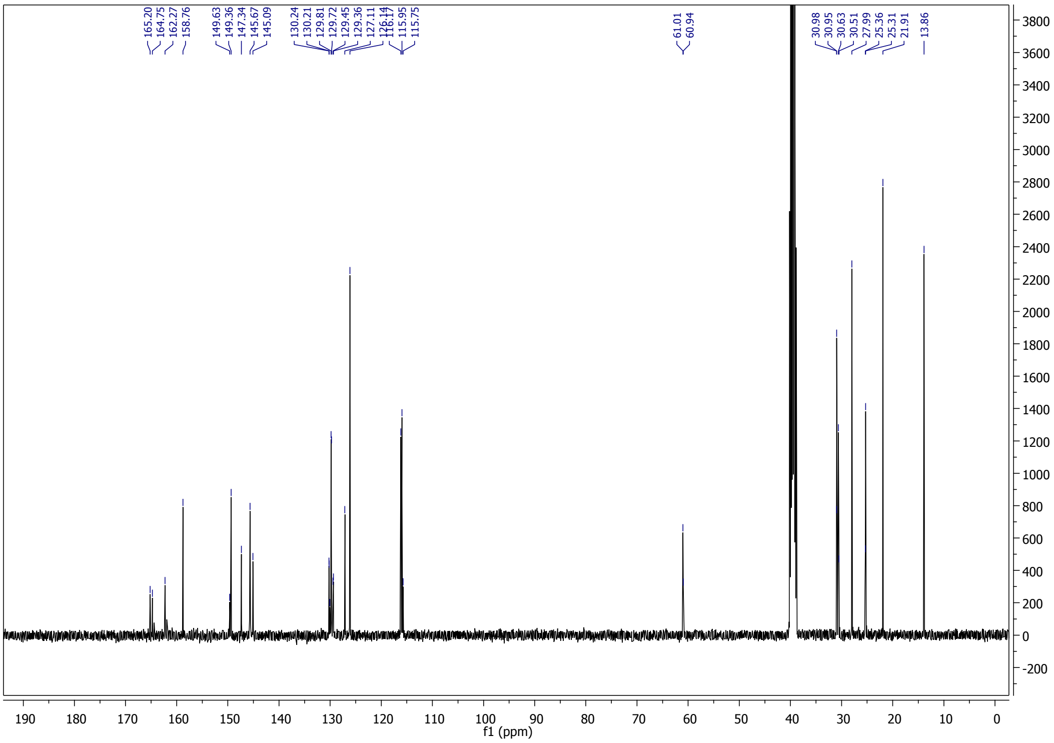

Figure S24. ^{13}C NMR of Compound **10**.

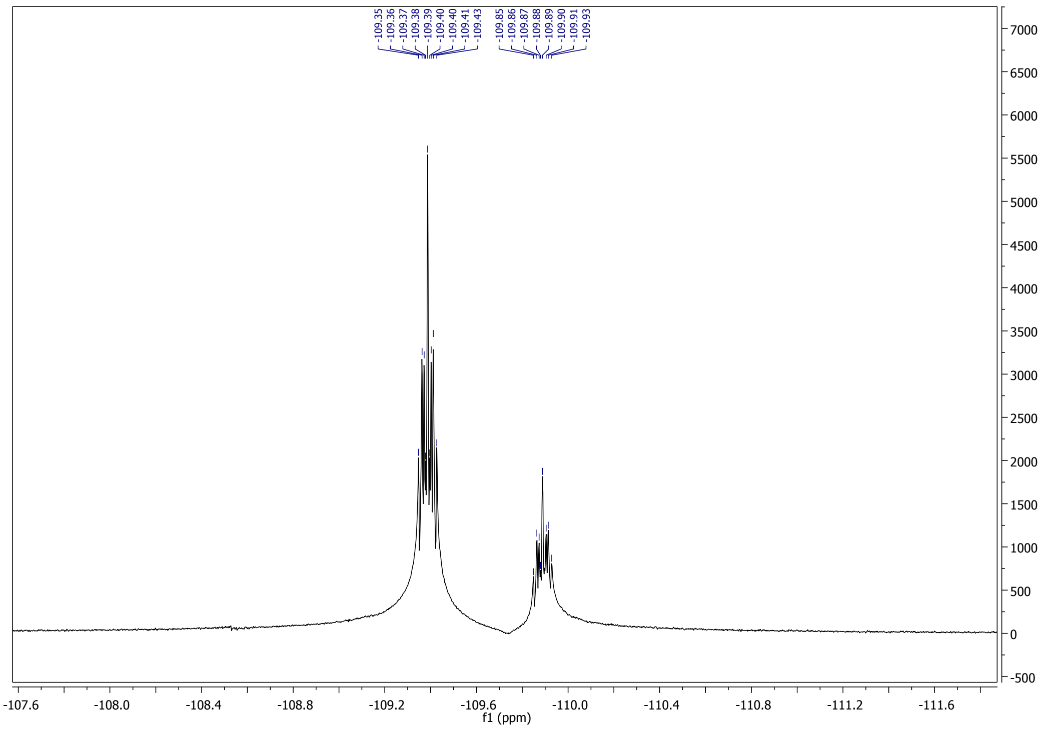

Figure S25. ^{19}F NMR of Compound **10**.

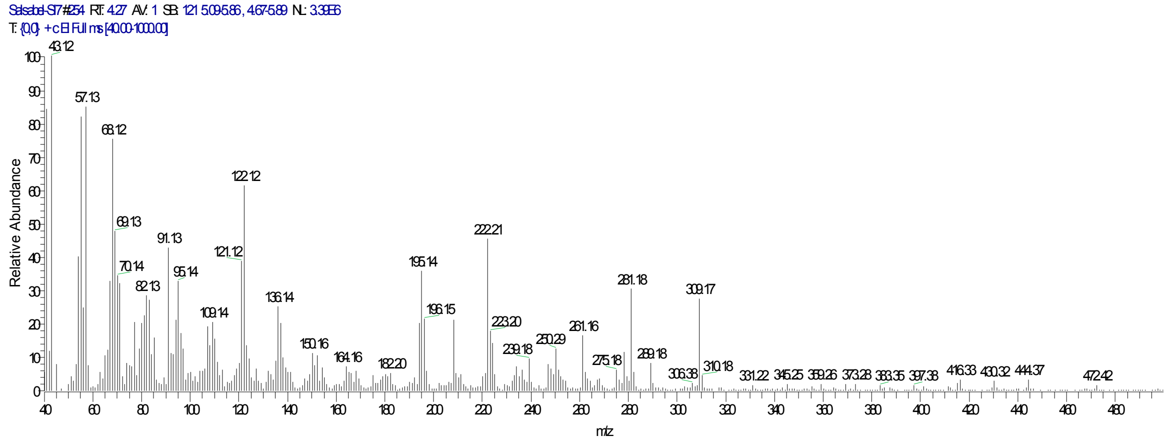

Figure S26. MS (ESI) of Compound **10**.

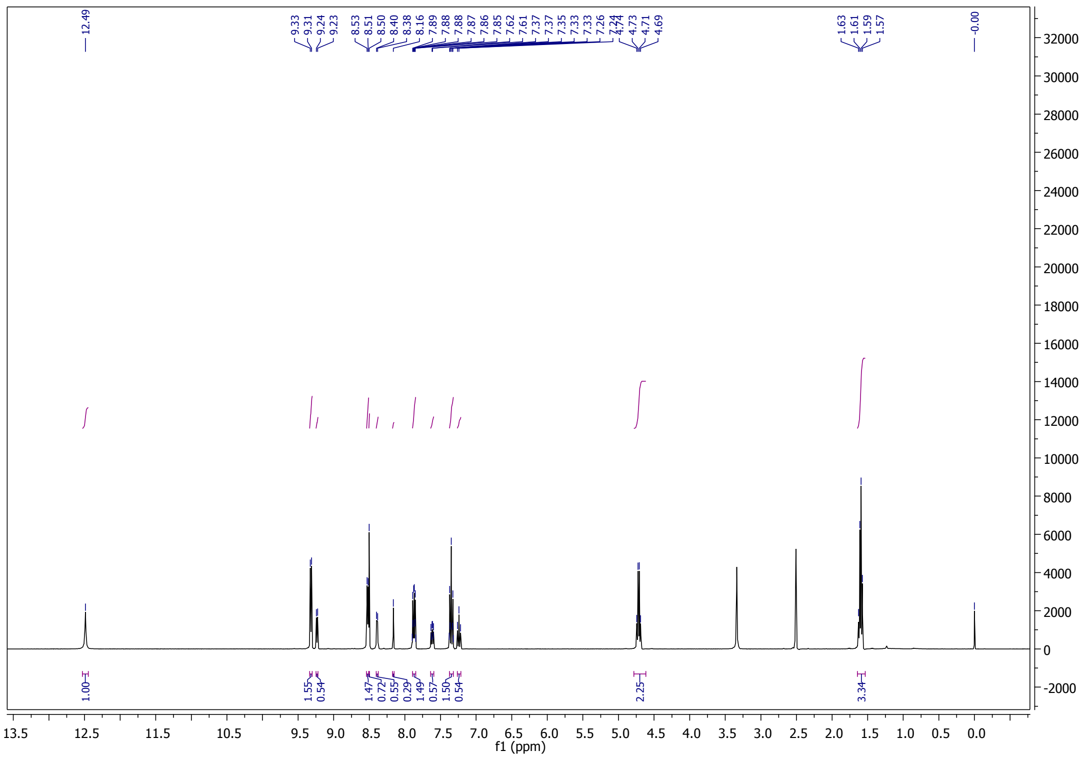

Figure S27. ^{1}H NMR of Compound **11**.

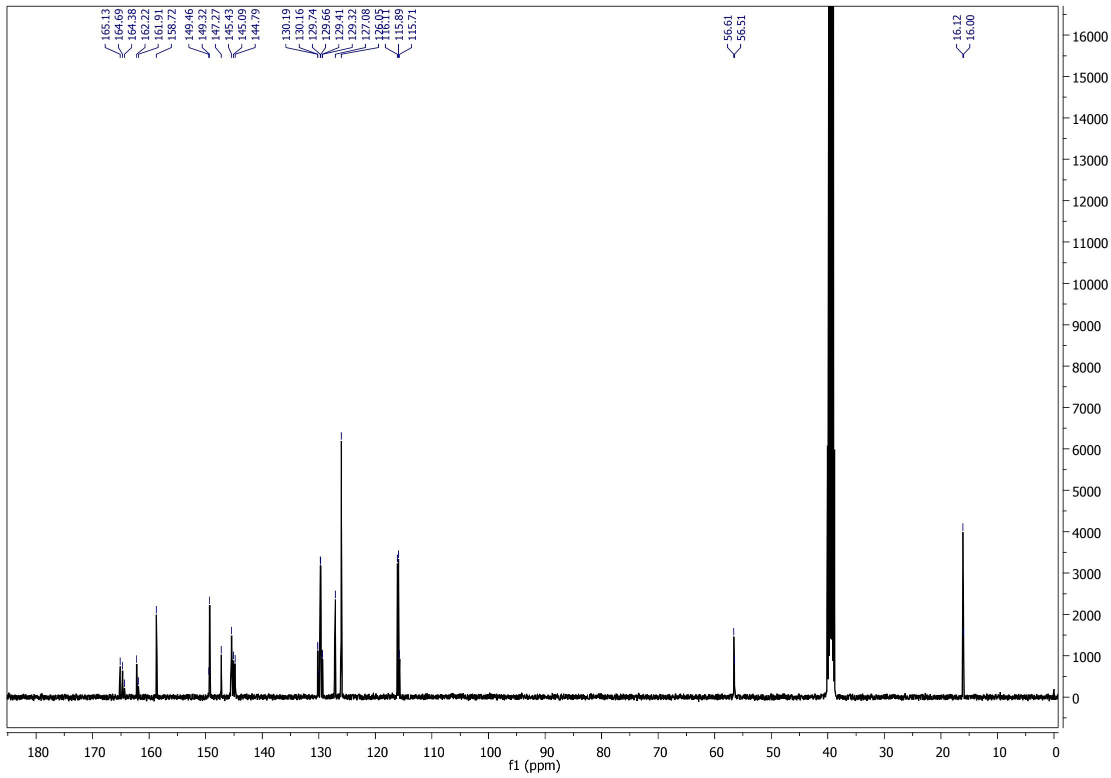

Figure S28. ^{13}C NMR of Compound **11**.

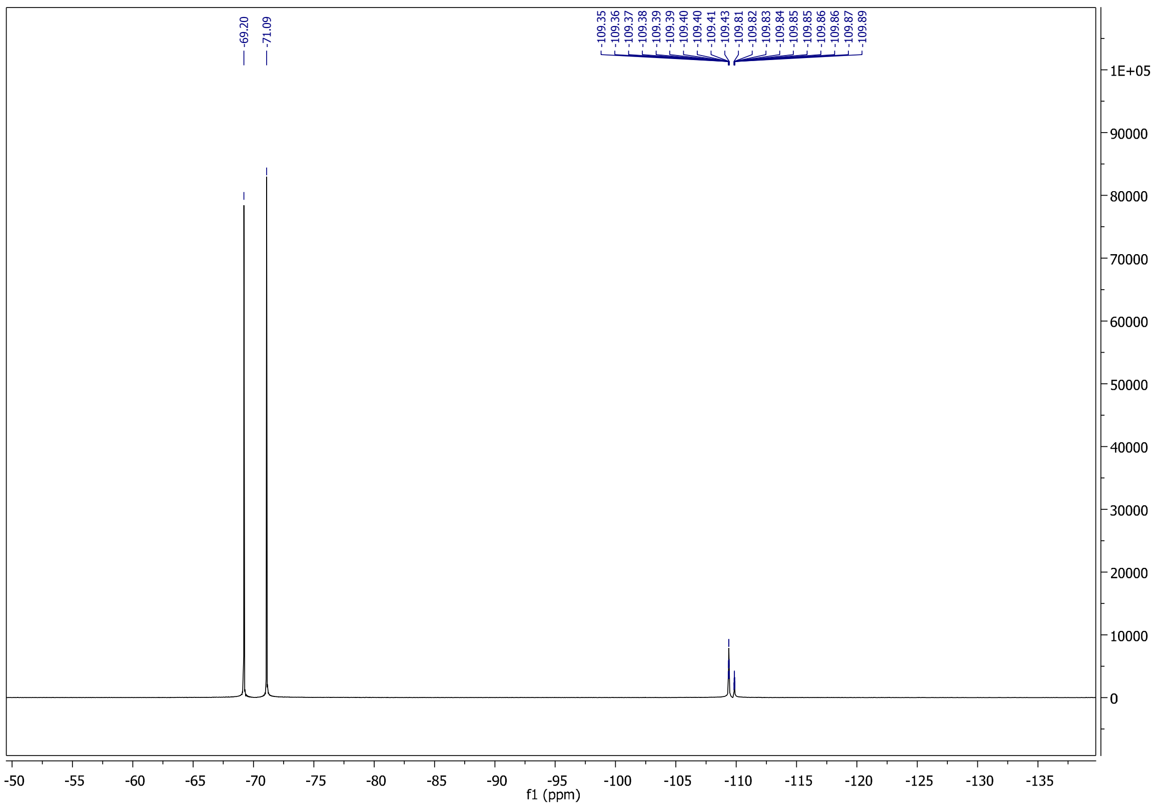

Figure S29. ^{19}F NMR of Compound **11**.

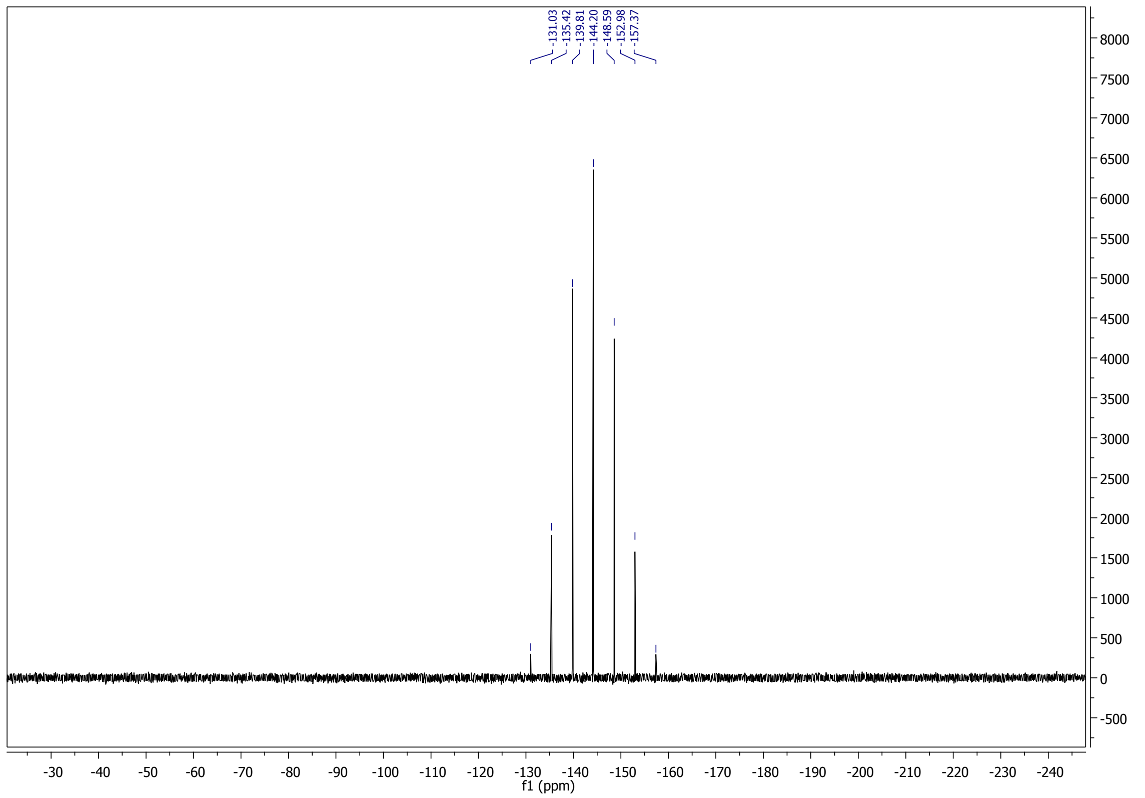

Figure S30. ^{31}P NMR of Compound **11**.

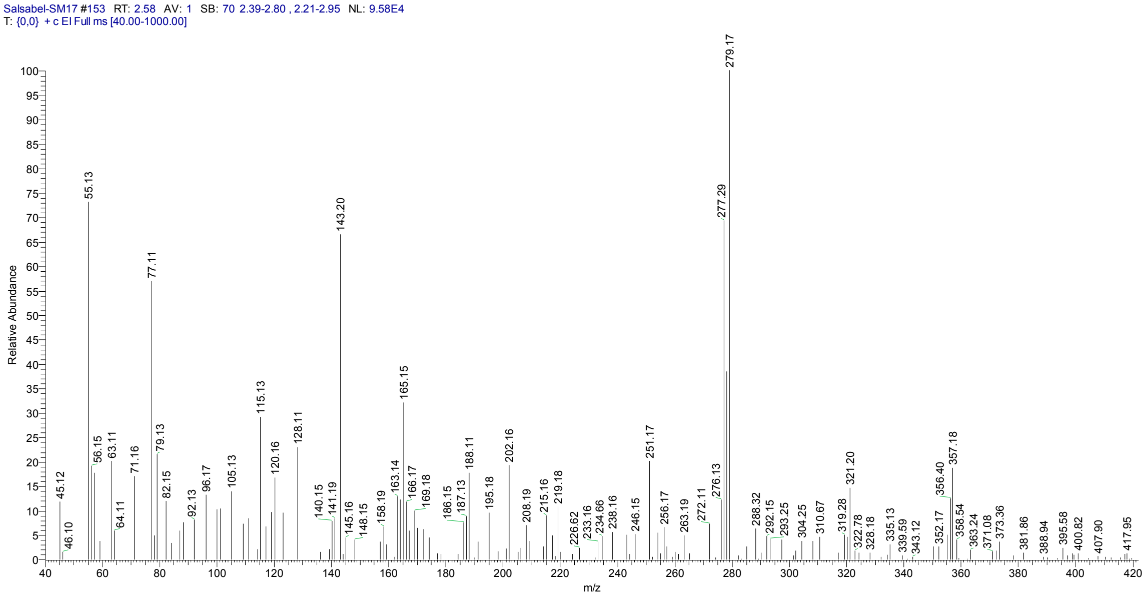

Figure S31. MS (ESI) of Compound **11**.

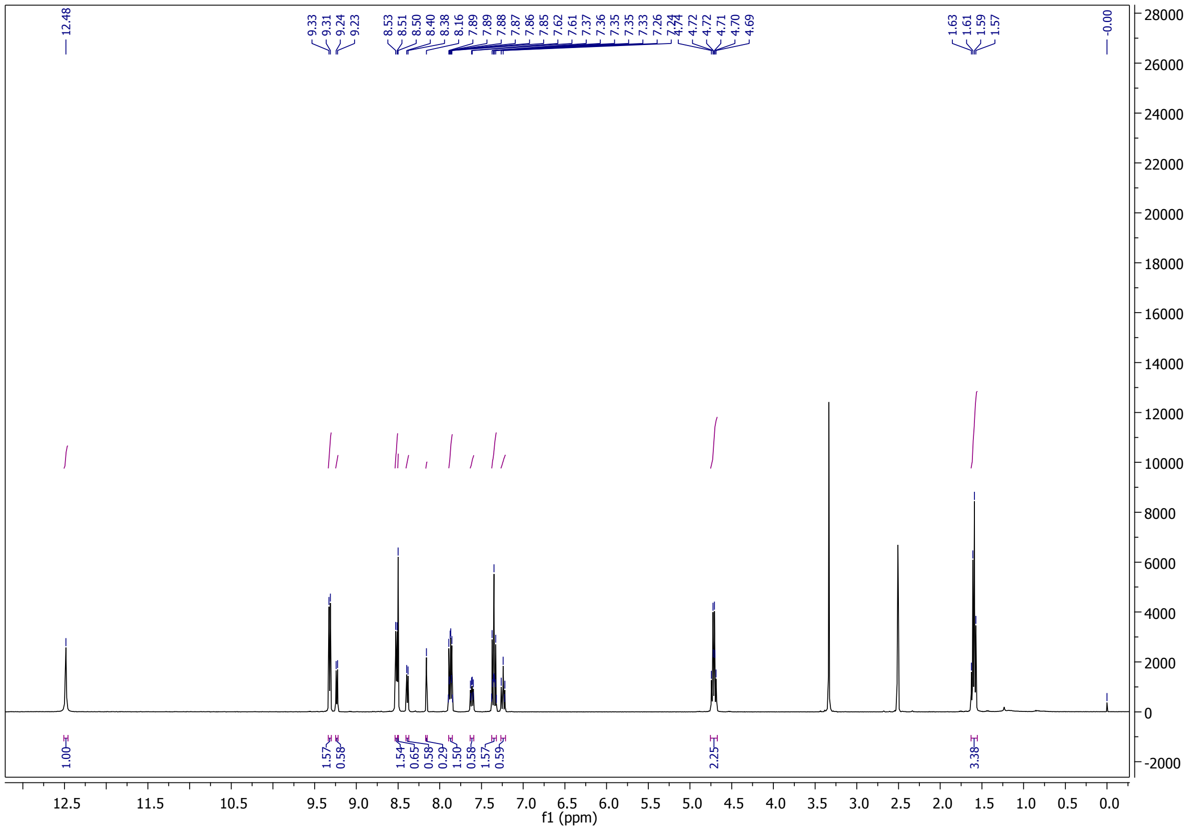

Figure S32. ^{1}H NMR of Compound **12**.

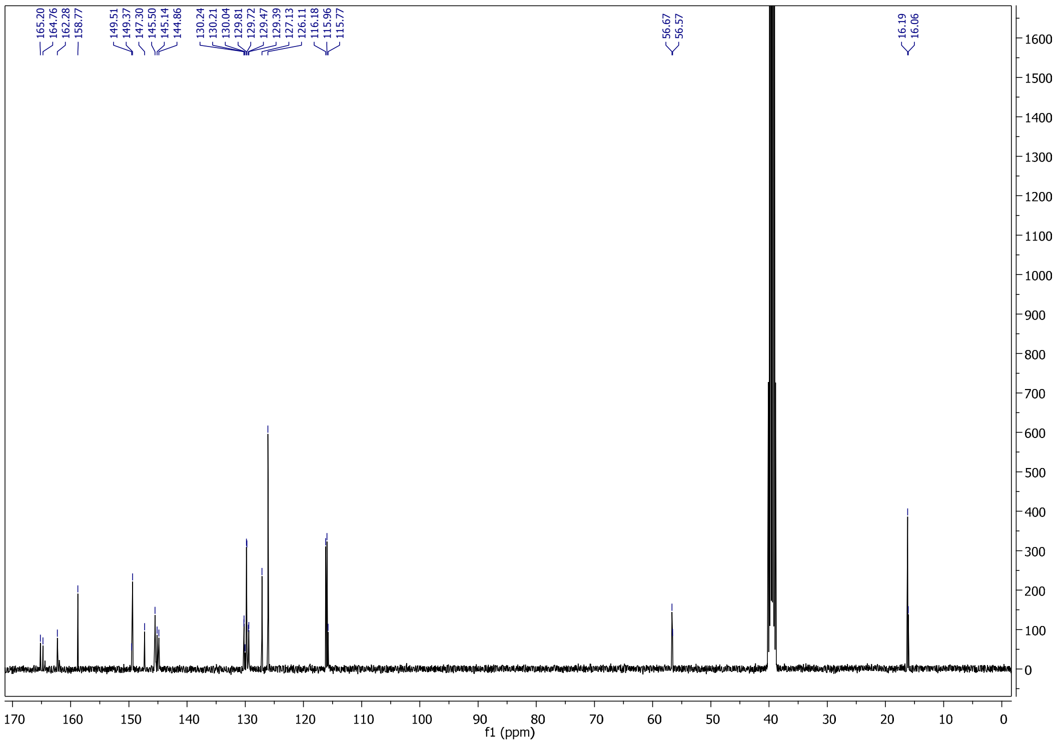

Figure S33. ^{13}C NMR of Compound **12**.

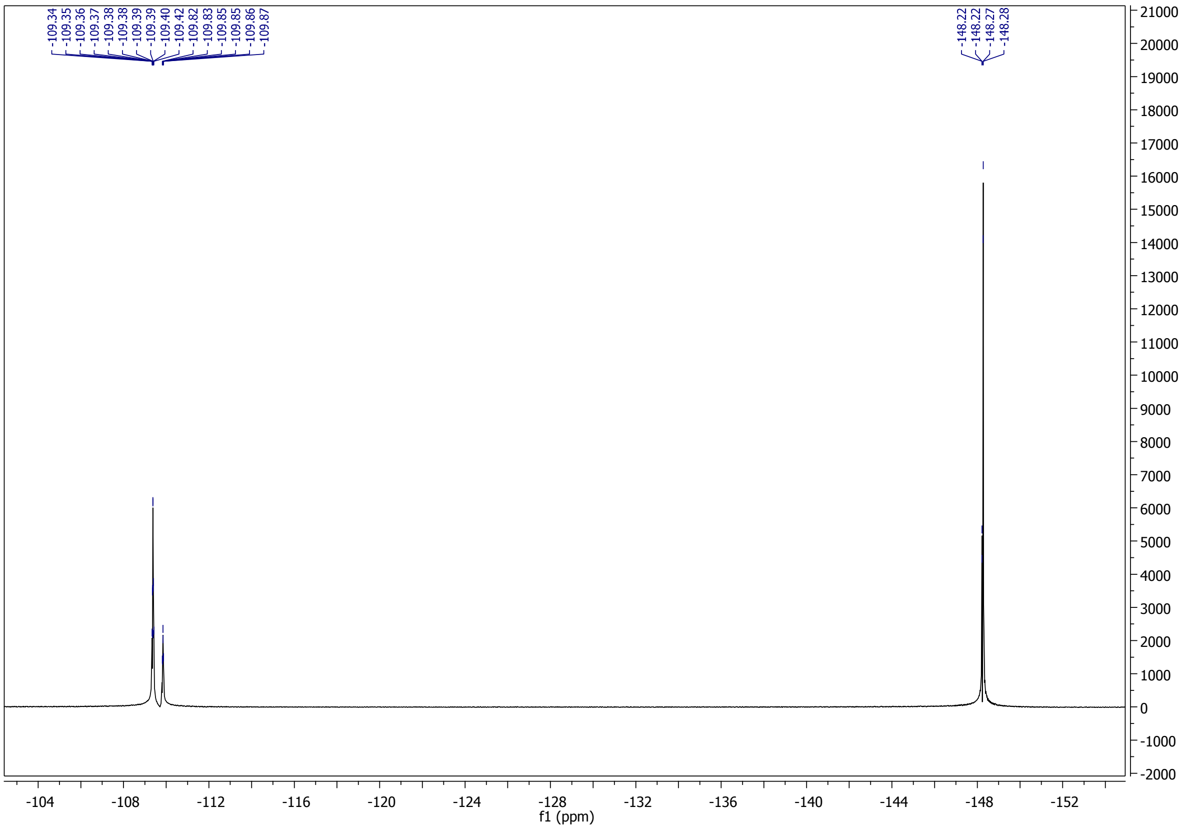

Figure S34. ^{19}F NMR of Compound **12**.

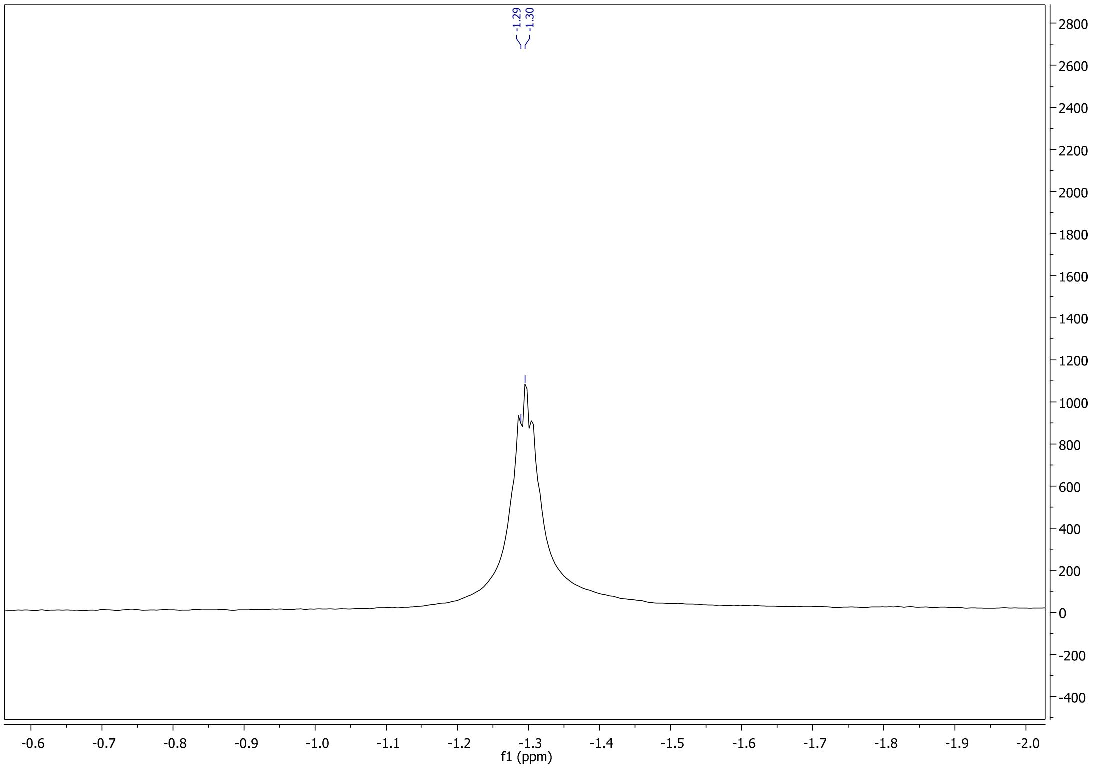

Figure S35. ^{11}B NMR of Compound **12**.

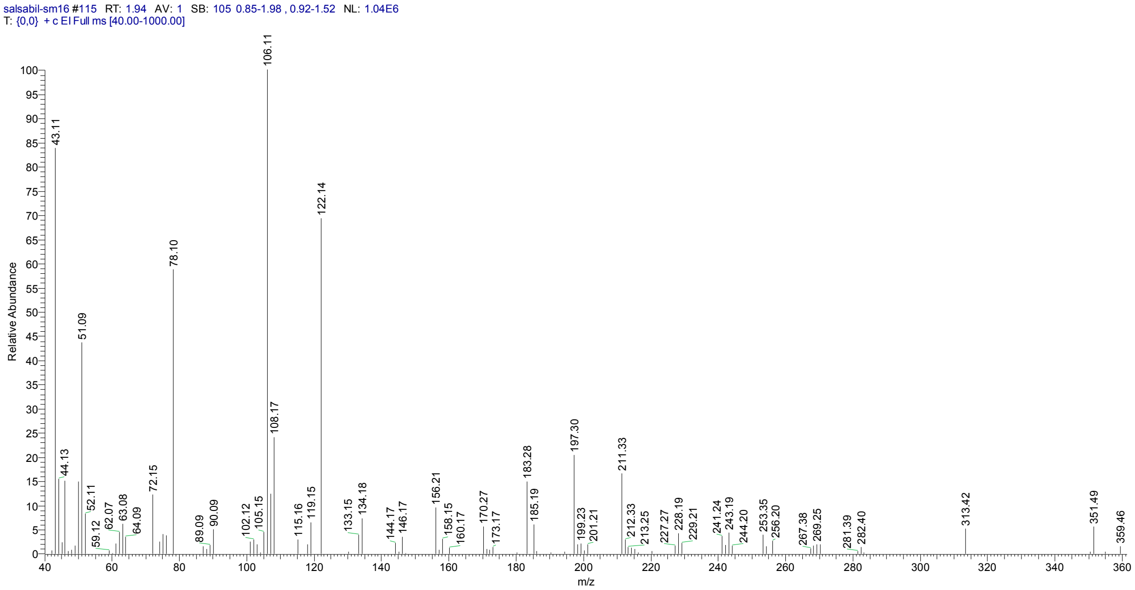

Figure S36. MS (ESI) of Compound **12**.

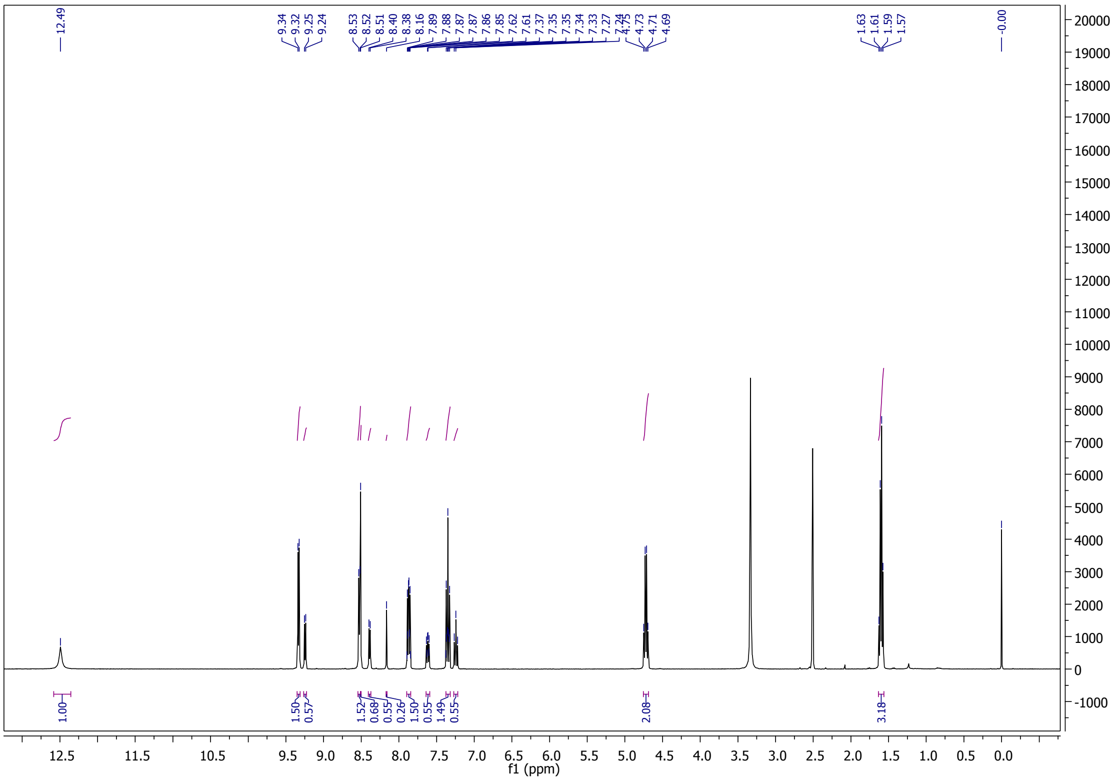

Figure S37. ^{1}H NMR of Compound **13**.

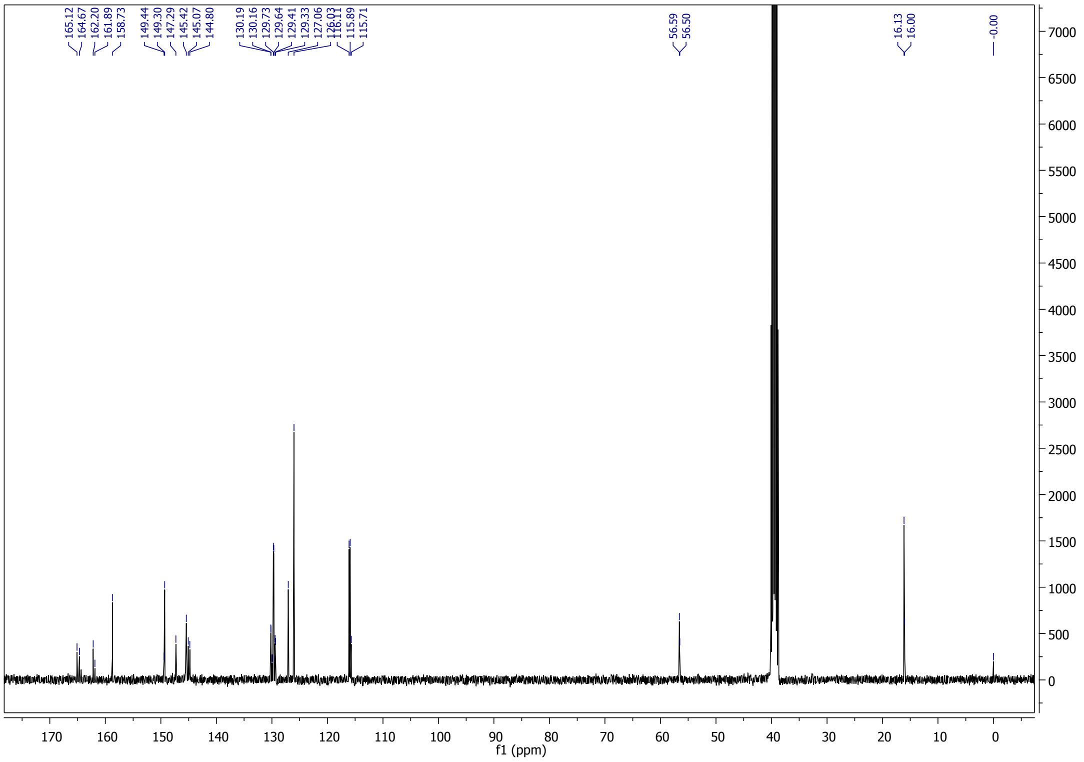

Figure S38. ^{13}C NMR of Compound **13**.

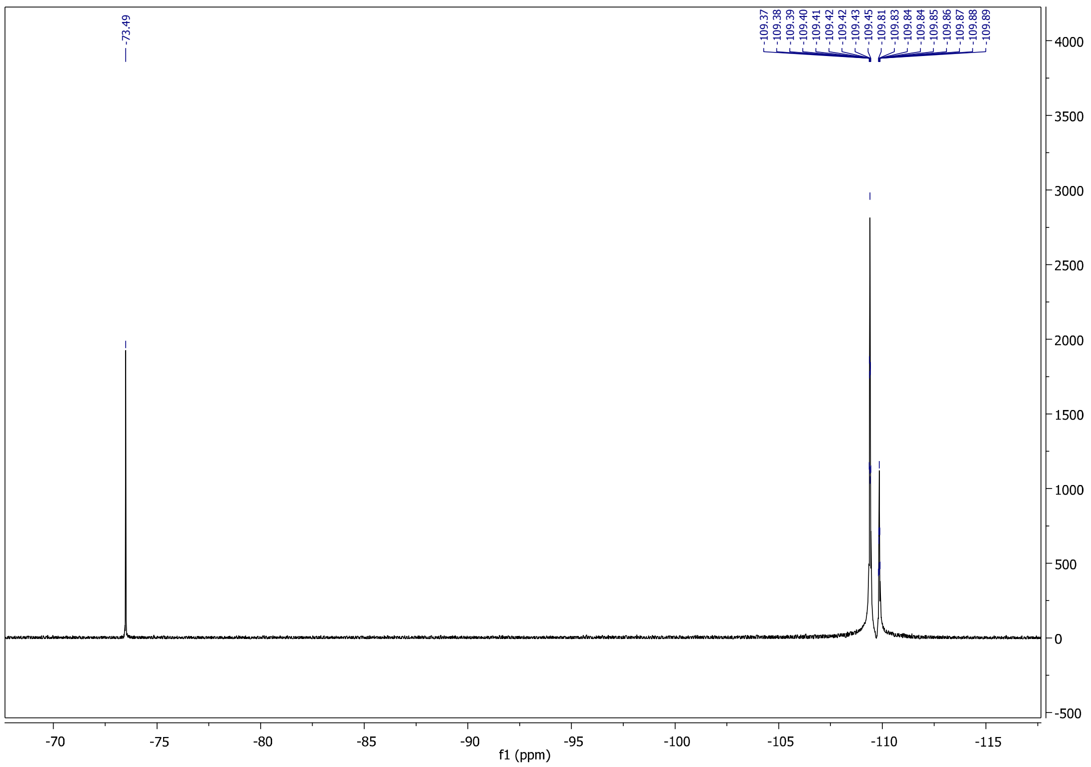

Figure S39. ^{19}F NMR of Compound **13**.

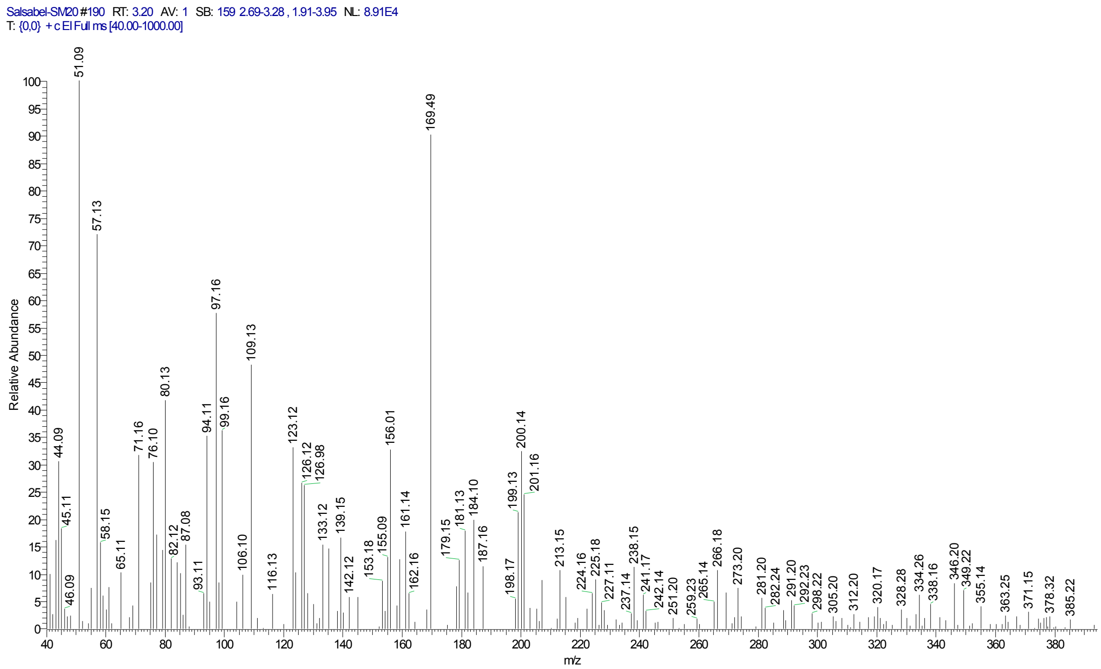

Figure S40. MS (ESI) of Compound **13**.

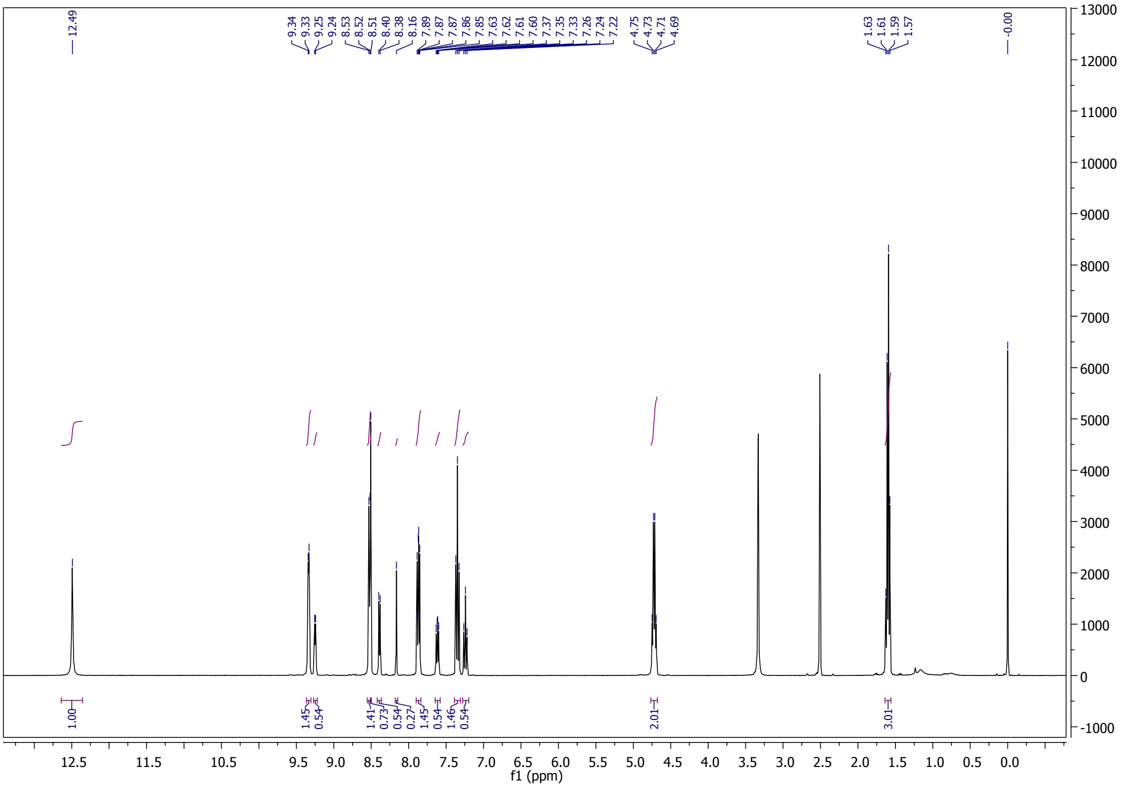

Figure S41. ^{1}H NMR of Compound **14**.

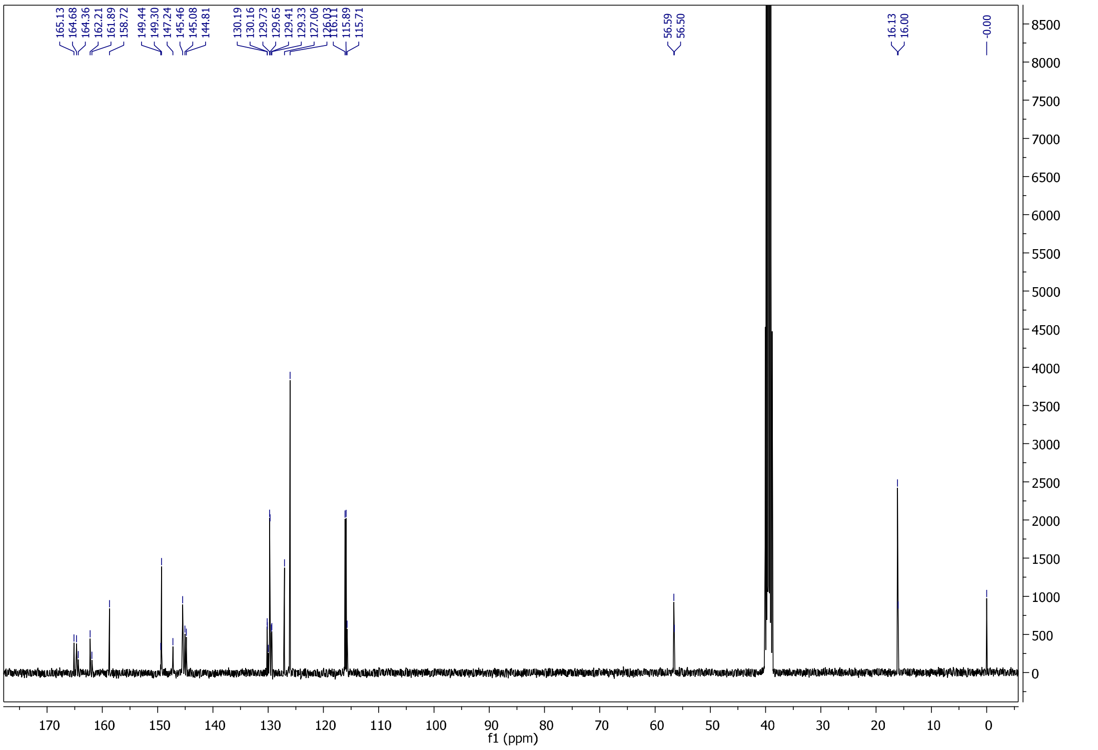

Figure S42. ^{13}C NMR of Compound **14**.

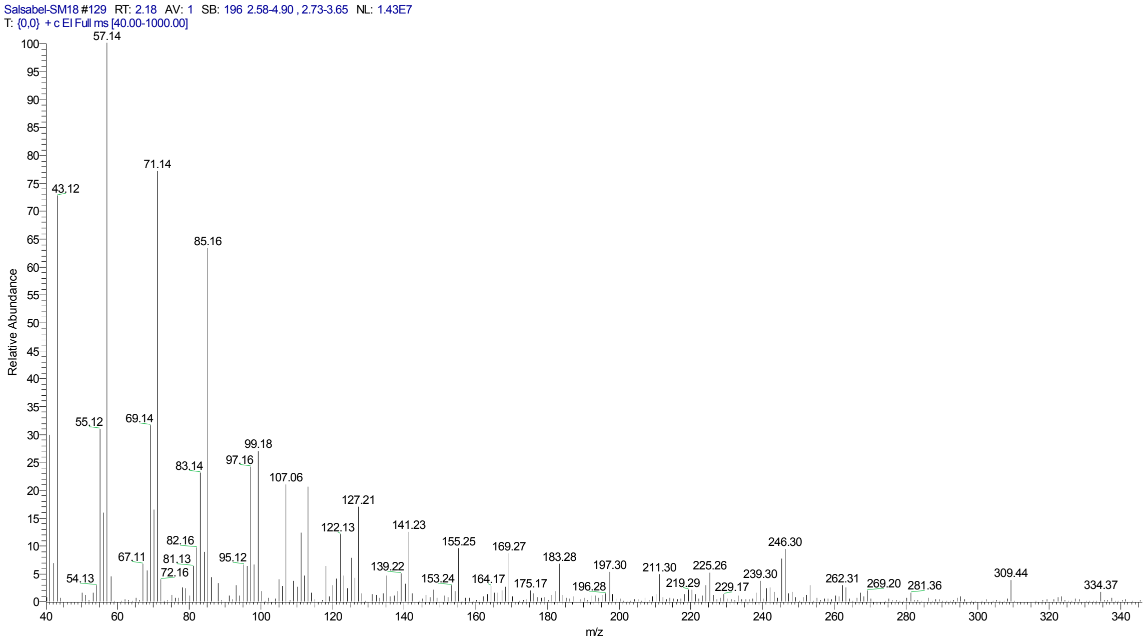

Figure S43. MS (ESI) of Compound **14**.

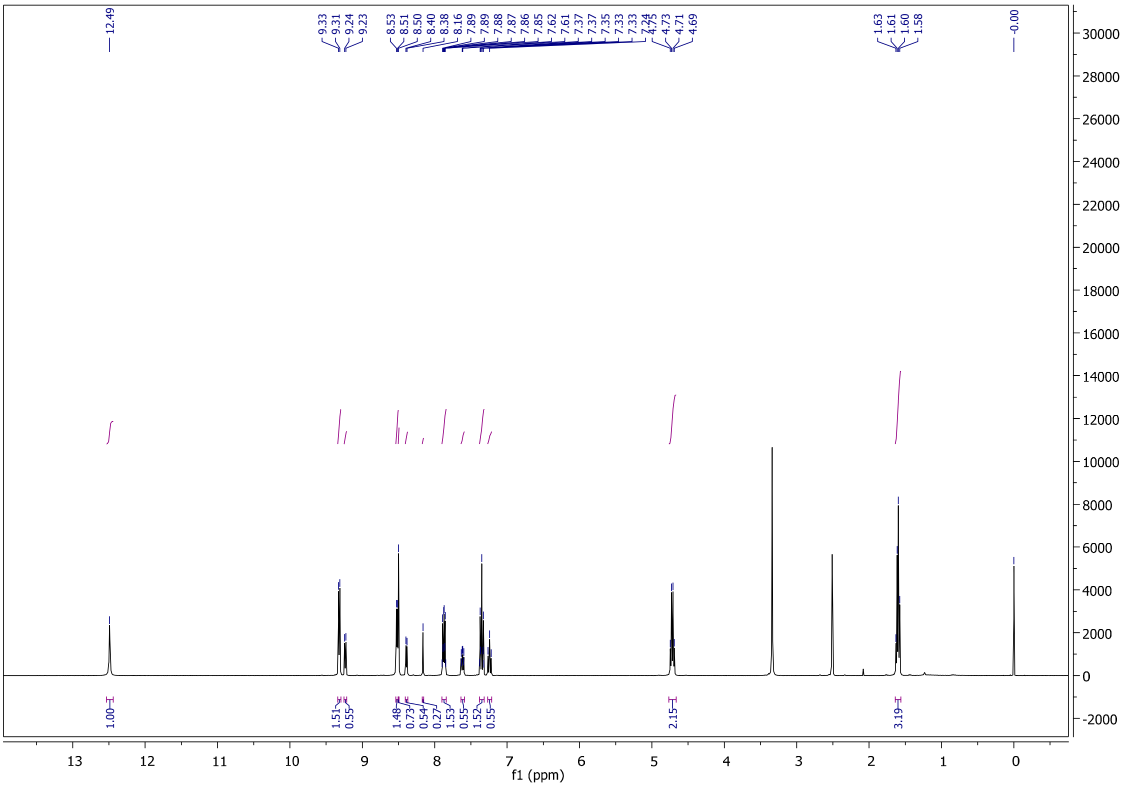

Figure S44. ^{1}H NMR of Compound **15**.

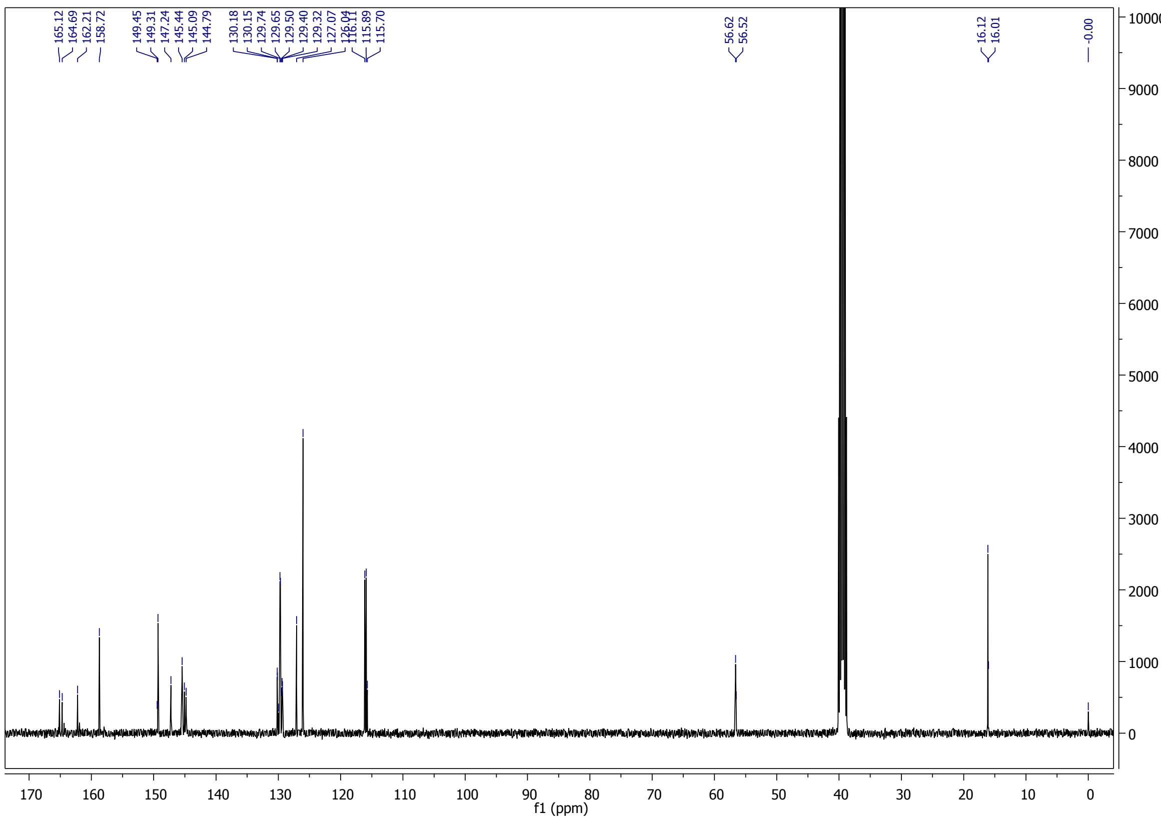

Figure S45. ^{13}C NMR of Compound **15**.

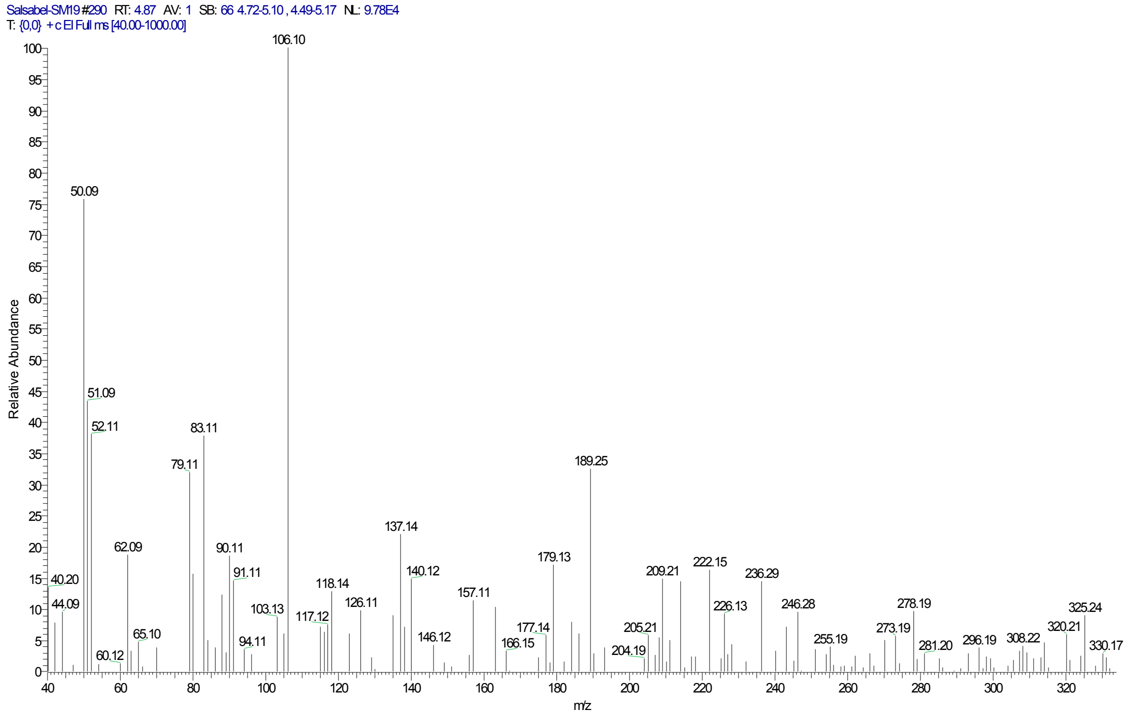

Figure S46. MS (ESI) of Compound **15**.

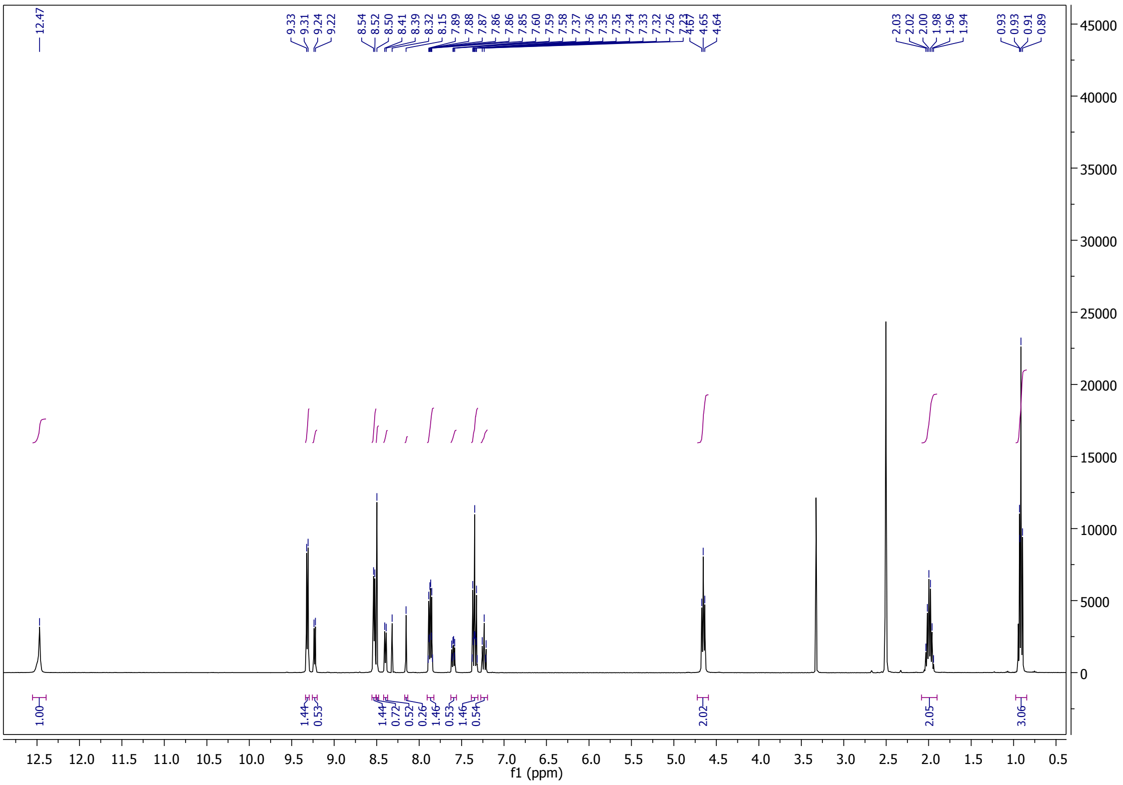

Figure S47. ^{1}H NMR of Compound **16**.

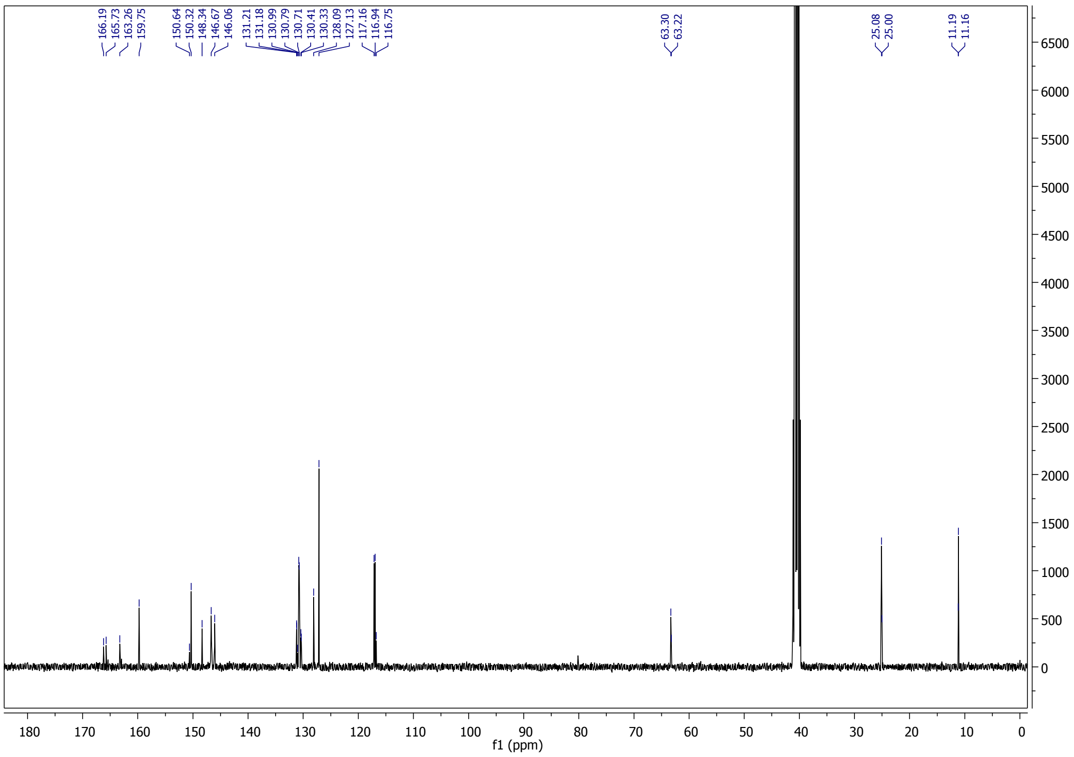

Figure S48. ^{13}C NMR of Compound **16**.

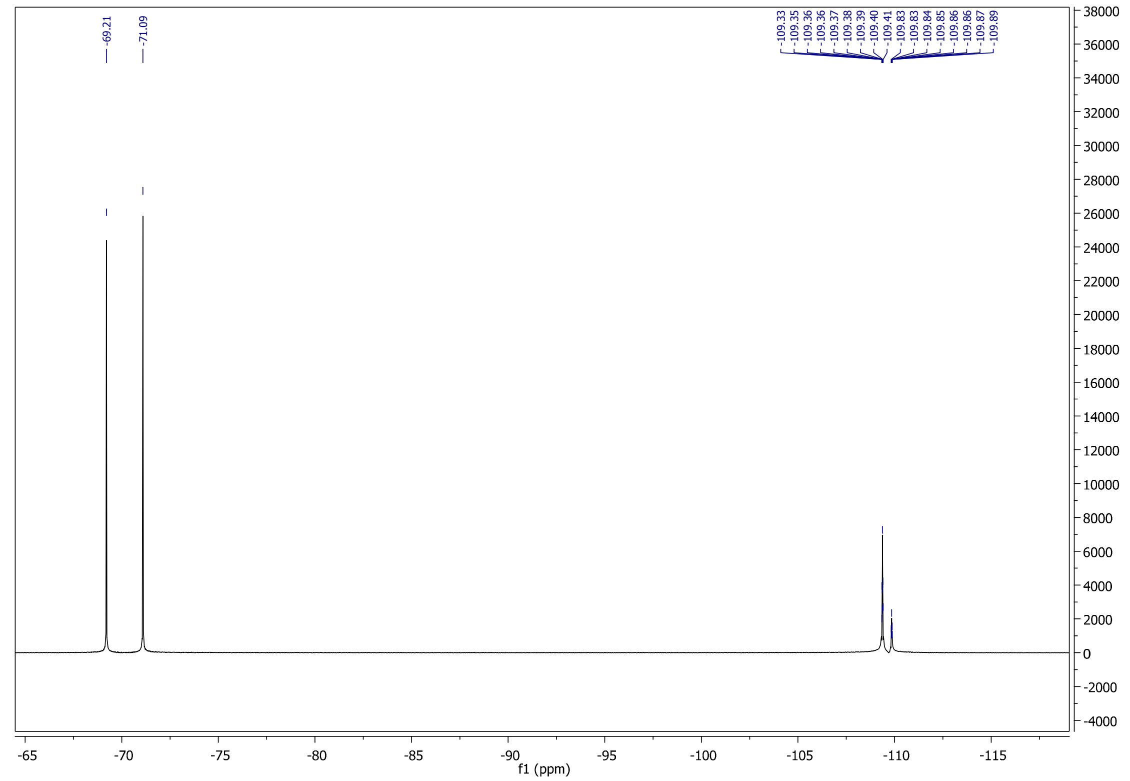

Figure S49. ^{19}F NMR of Compound **16**.

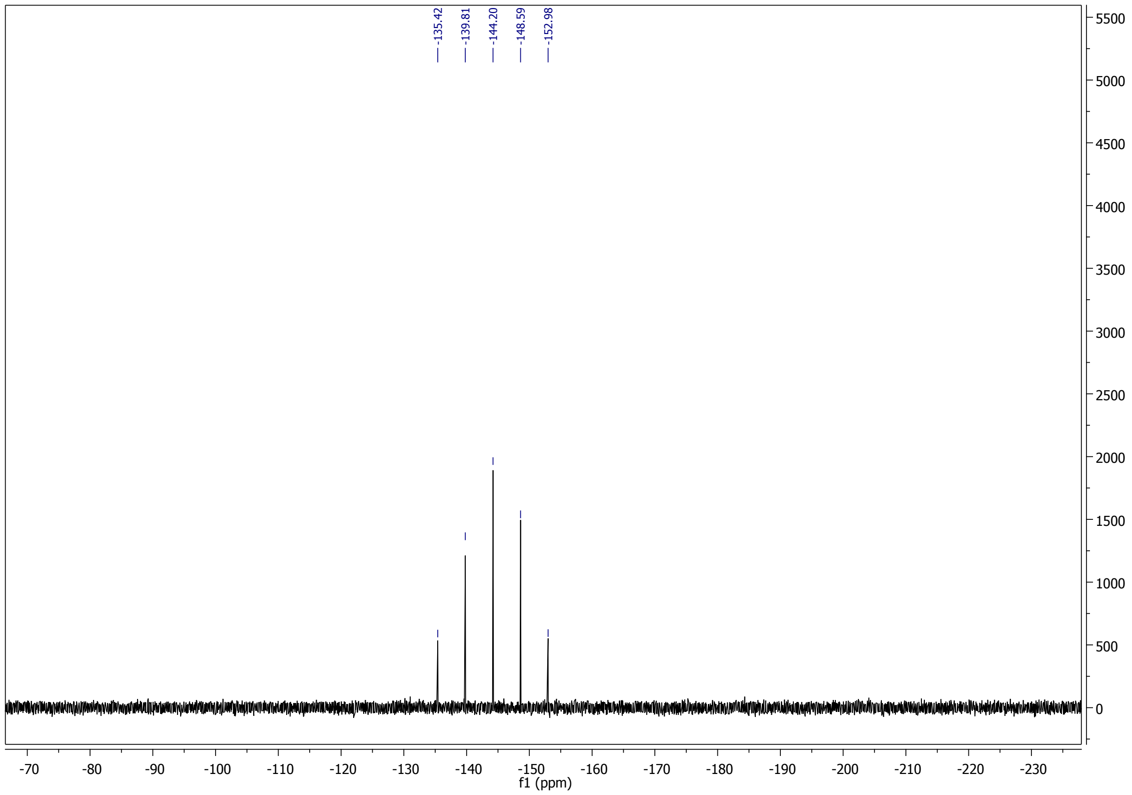

Figure S50. ^{31}P NMR of Compound **16**.

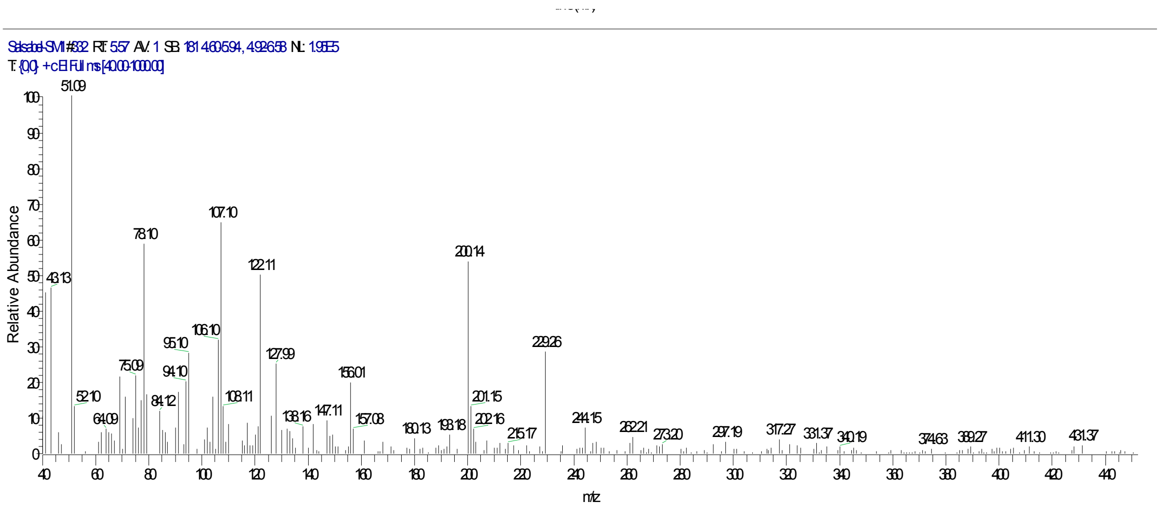

Figure S51. MS (ESI) of Compound **16**.

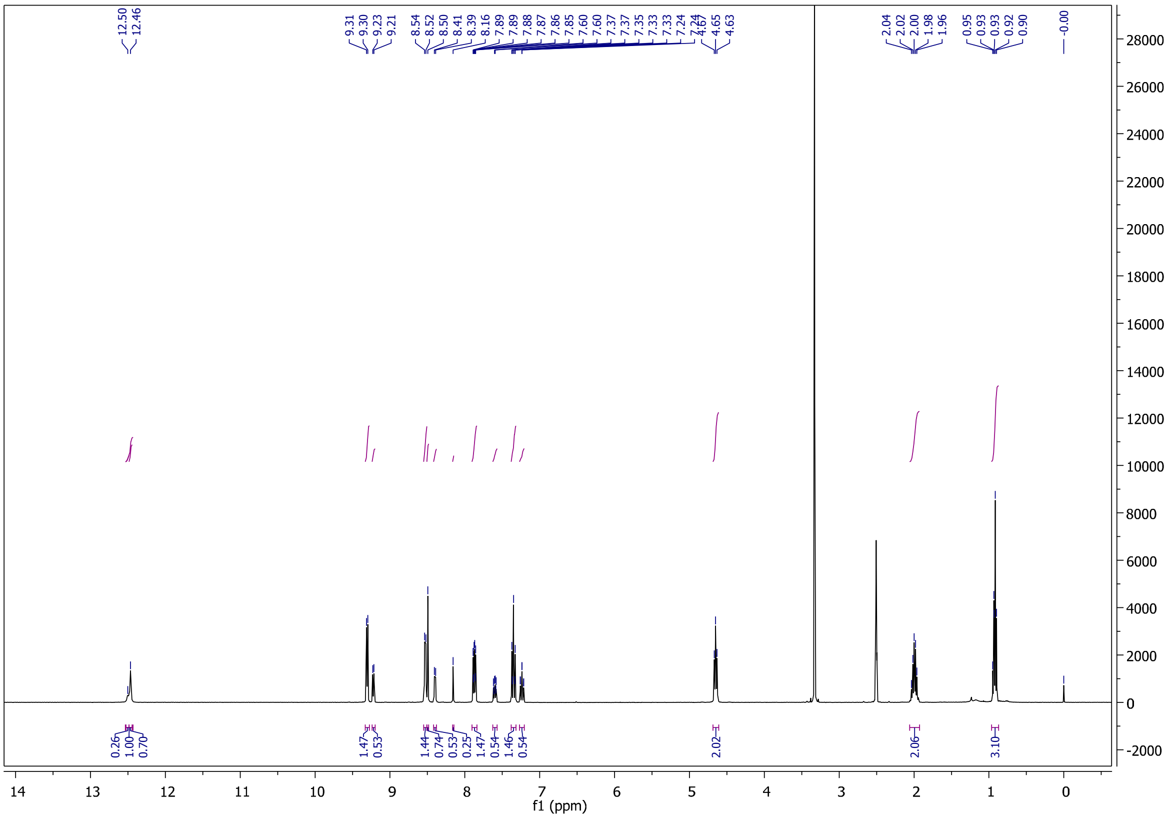

Figure S52. ^{1}H NMR of Compound **17**.

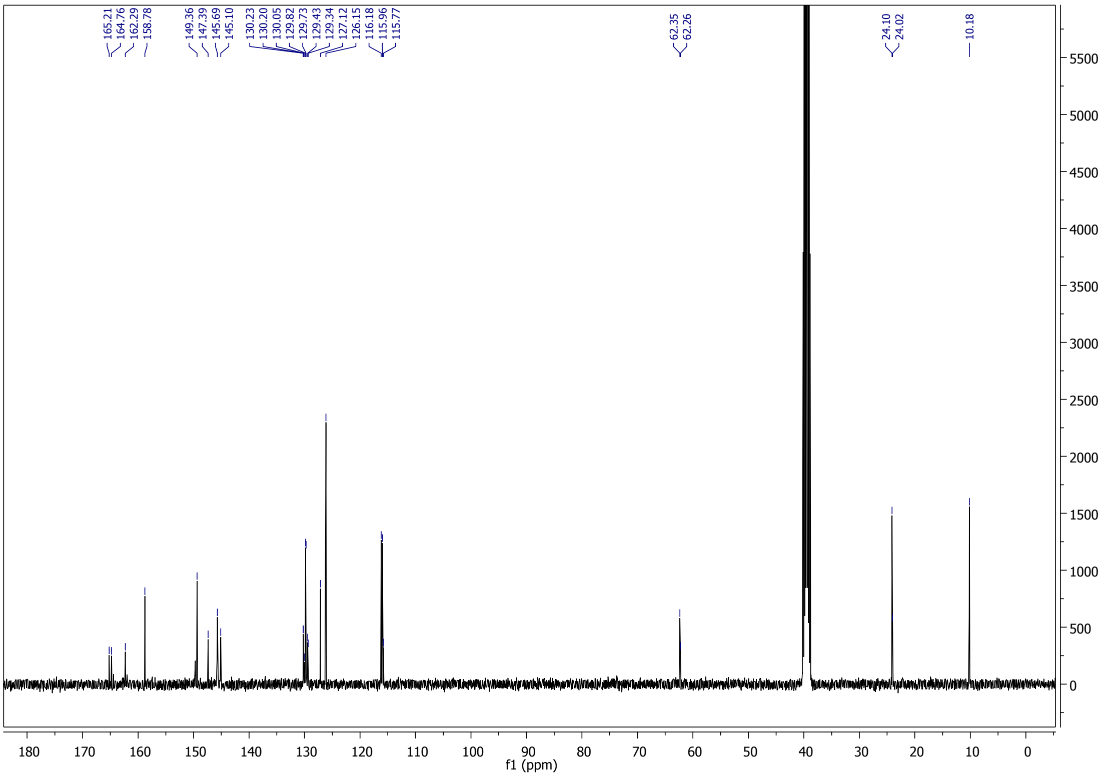

Figure S53. ^{13}C NMR of Compound **17**.

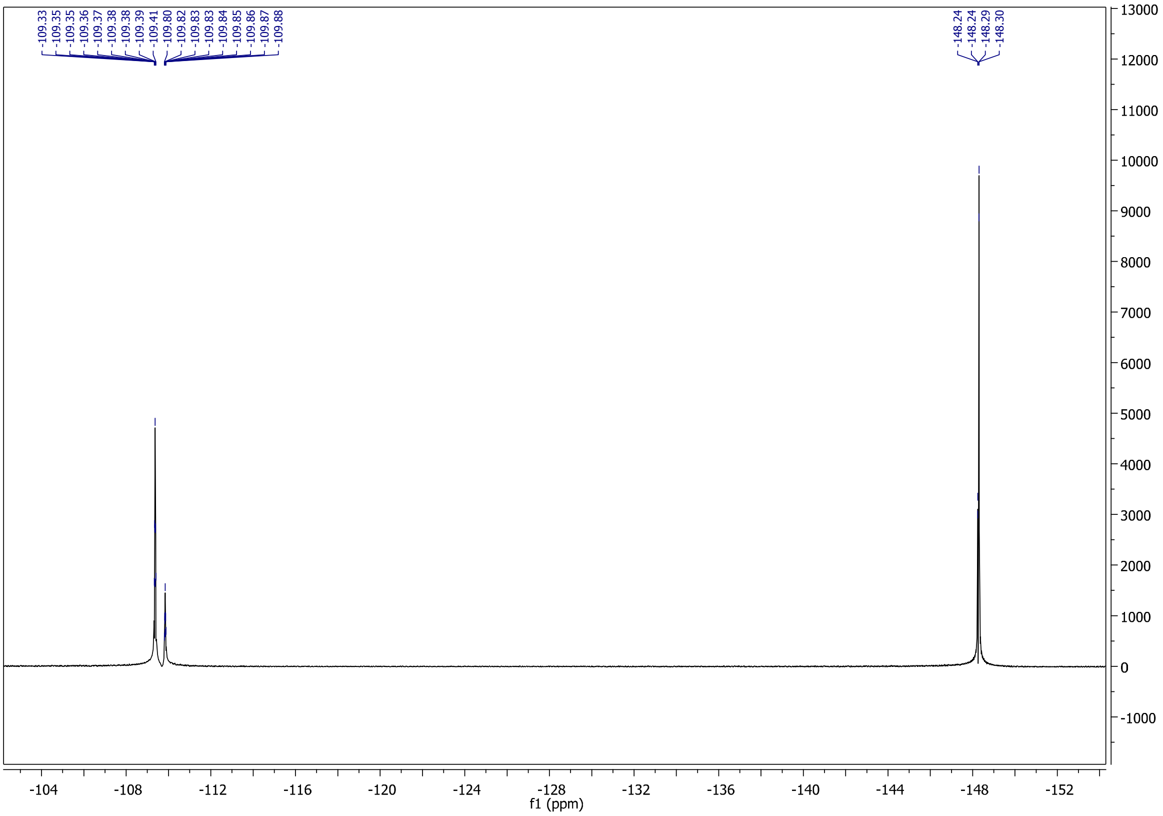

Figure S54. ^{19}F NMR of Compound **17**.

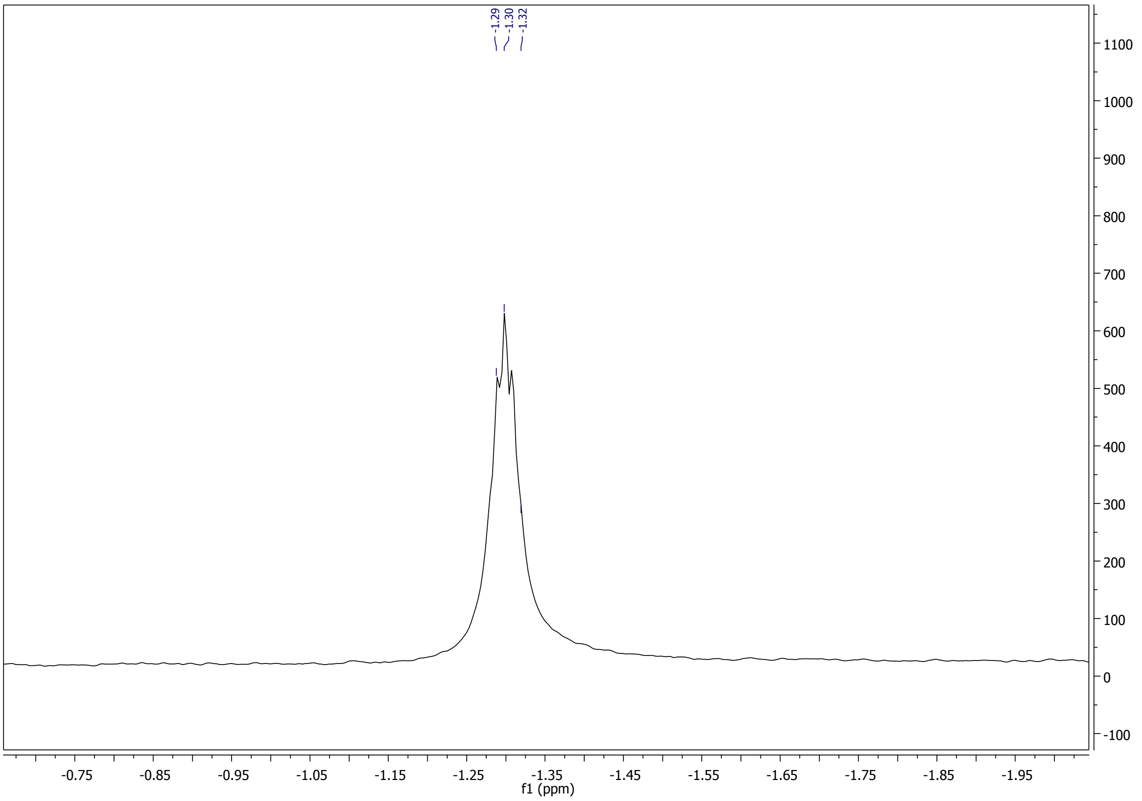

Figure S55. ^{11}B NMR of Compound **17**.

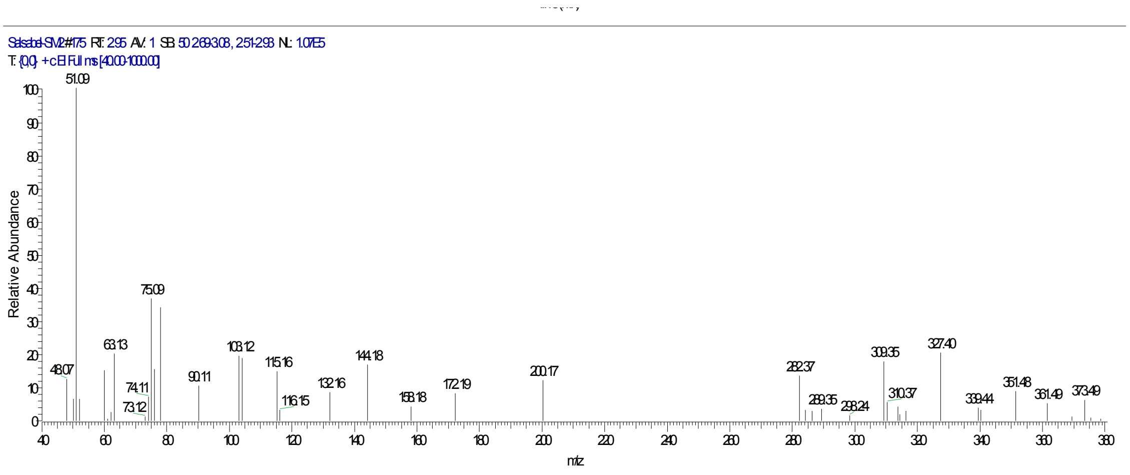

Figure S56. MS (ESI) of Compound **17**.

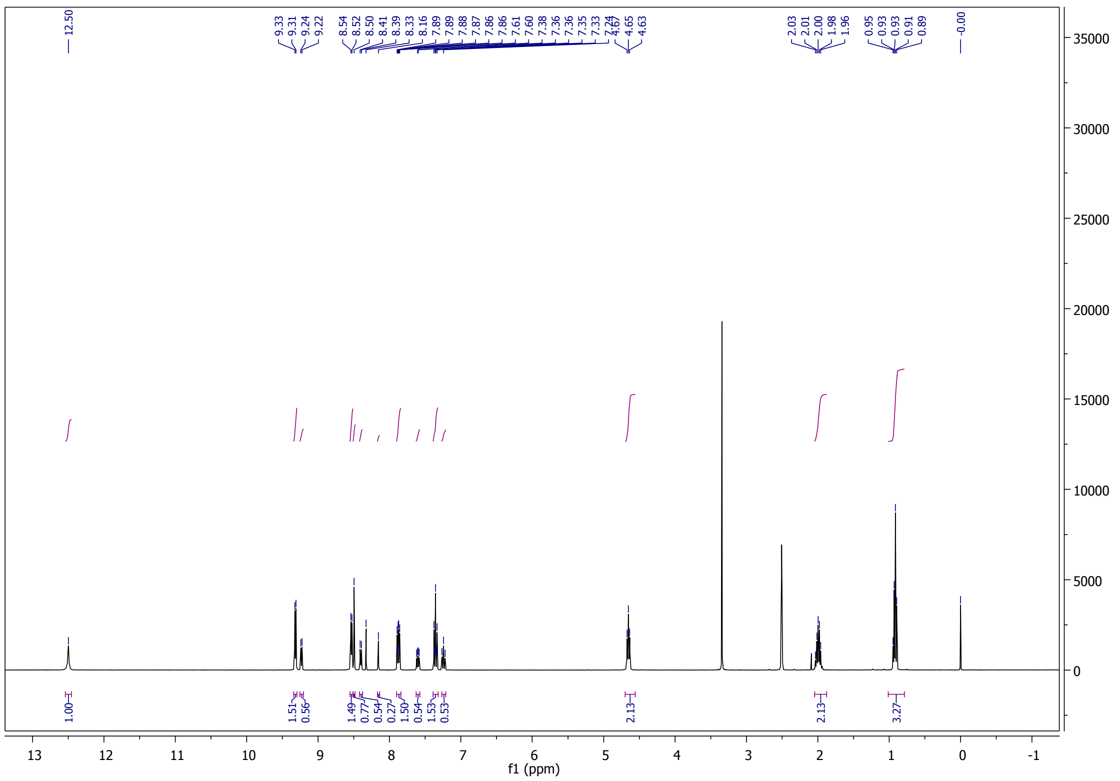

Figure S57. ^{1}H NMR of Compound **18**.

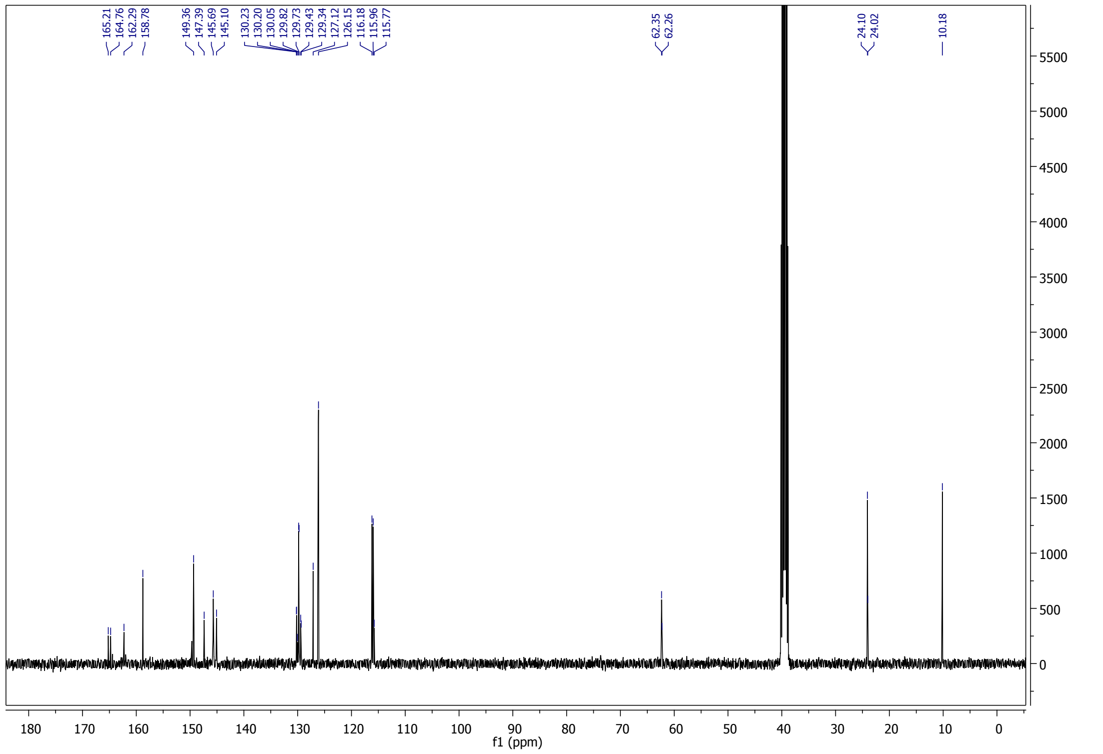

Figure S58. ^{13}C NMR of Compound **18**.

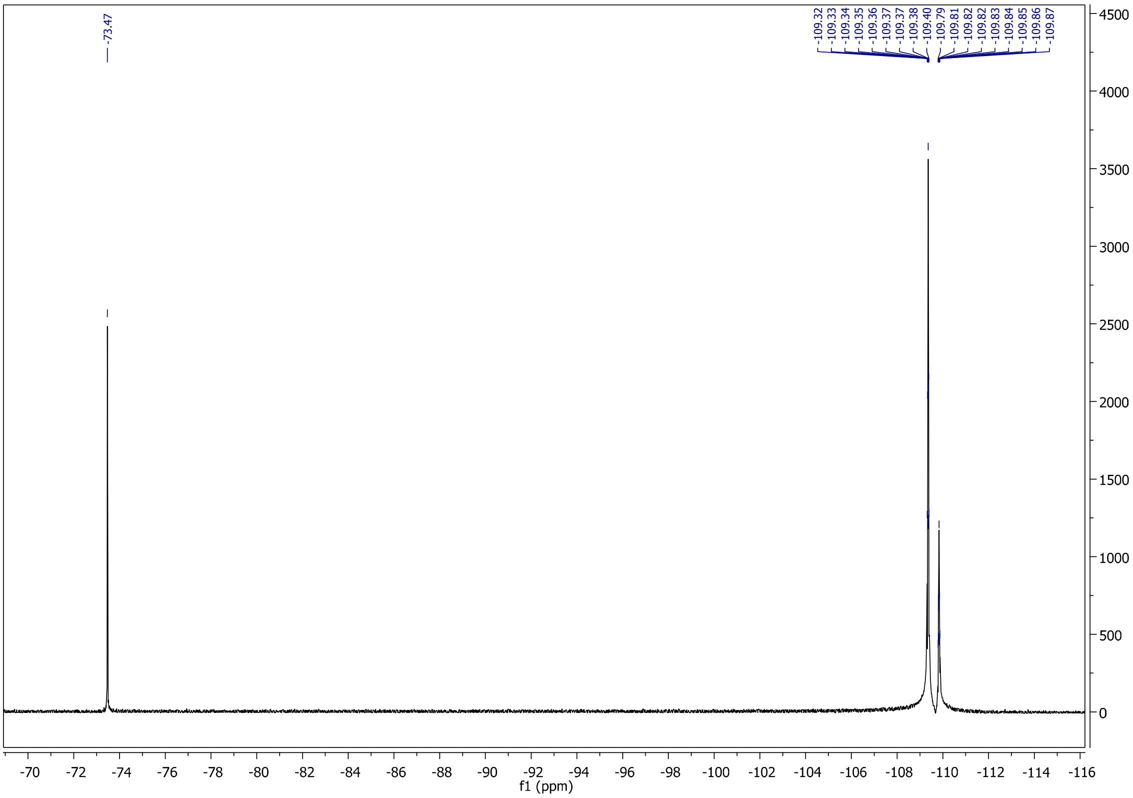

Figure S59. ^{19}F NMR of Compound **18**.

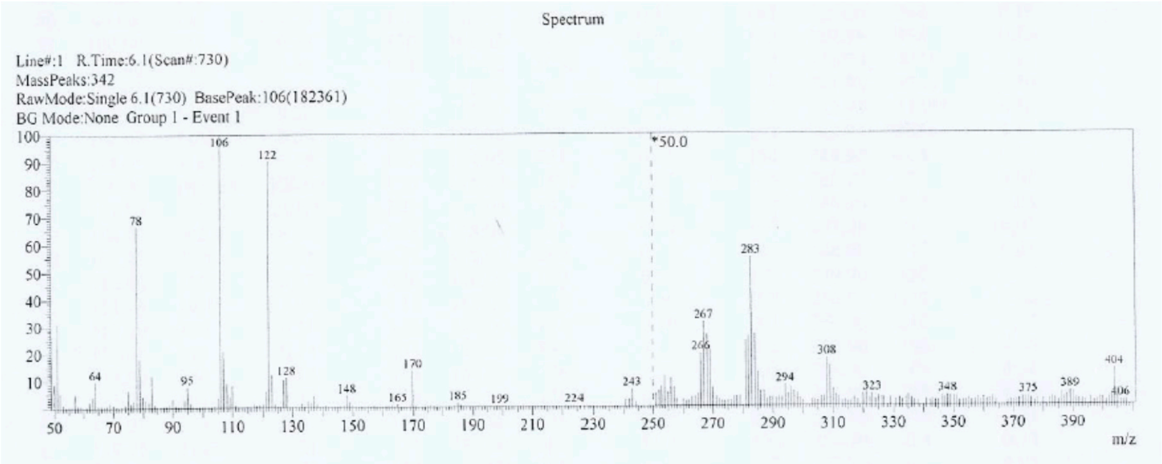

Figure S60. MS (ESI) of Compound **18**.

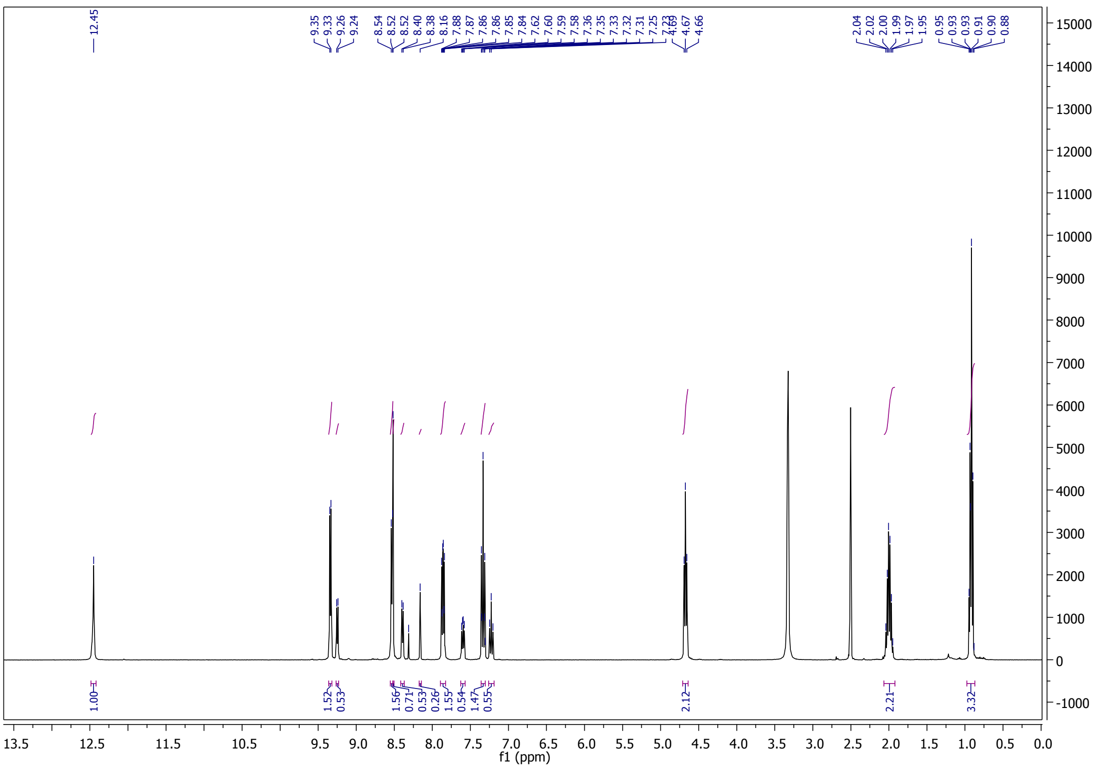

Figure S61. ^{1}H NMR of Compound **19**.

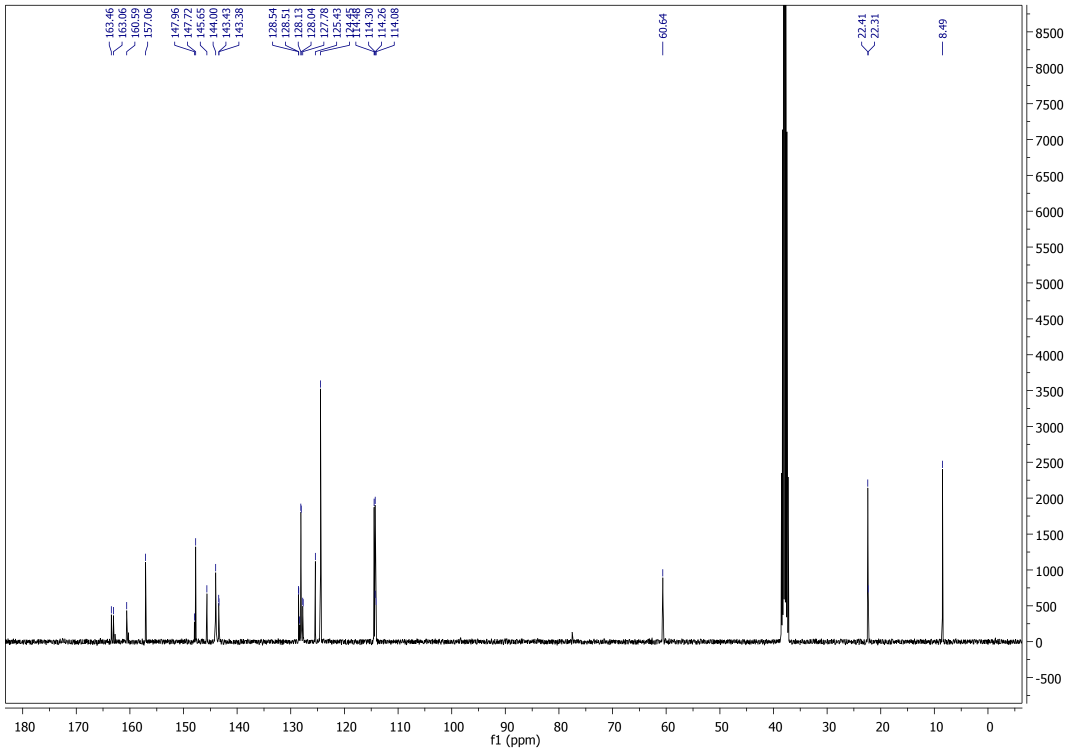

Figure S62. ^{13}C NMR of Compound **19**.

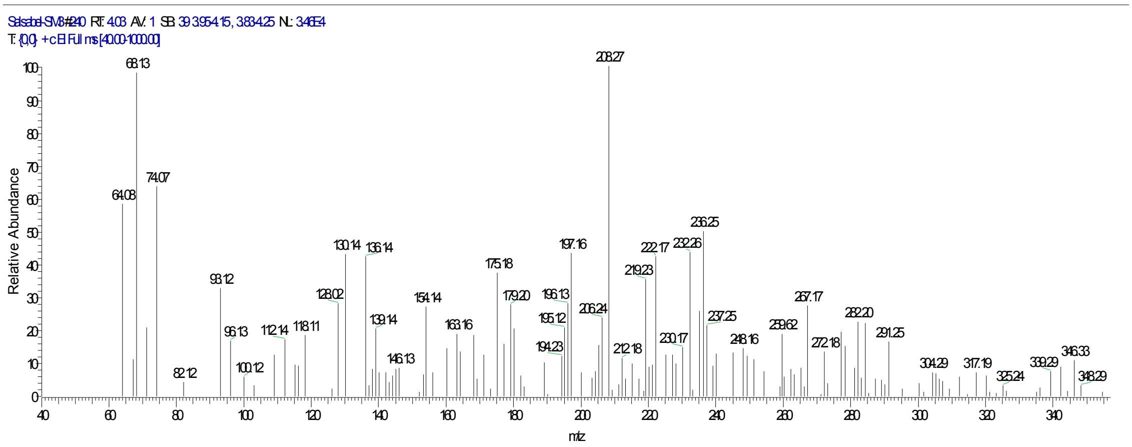

Figure S63. MS (ESI) of Compound **19**.

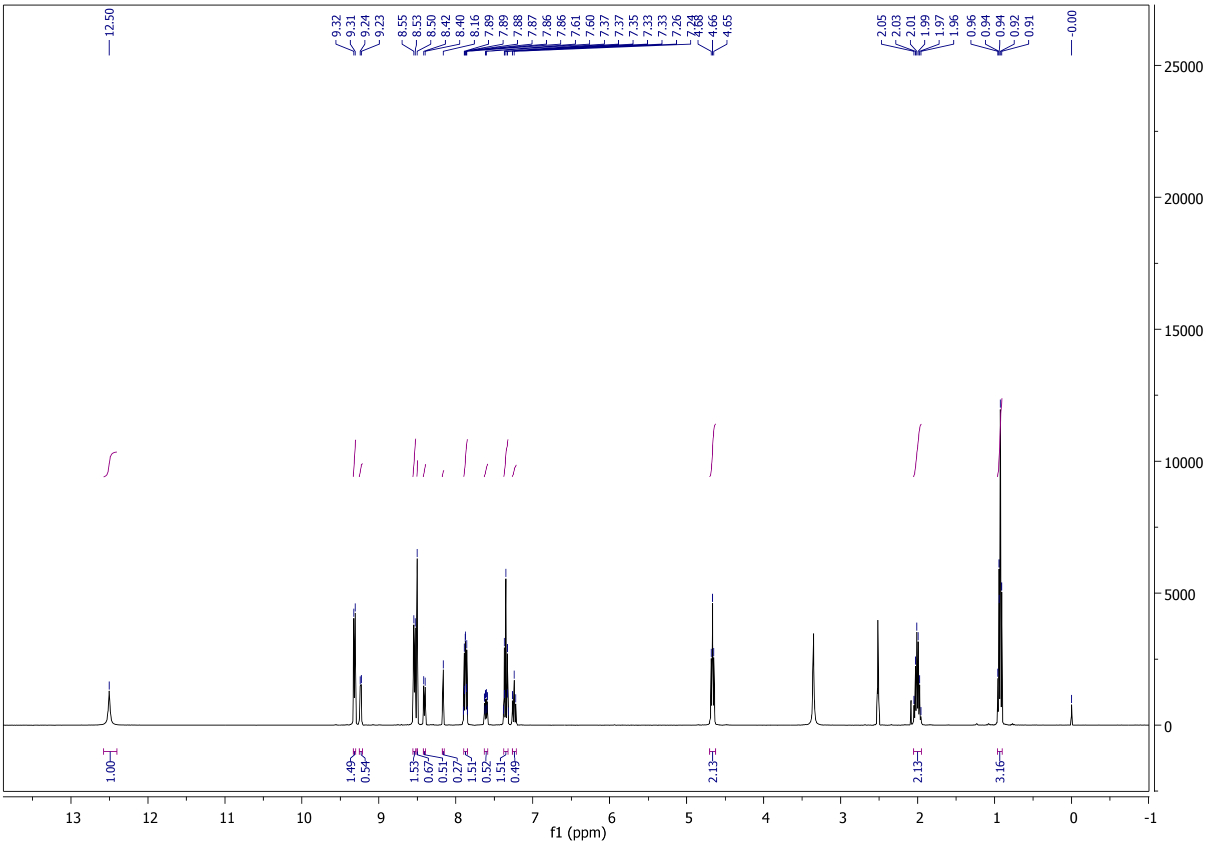

Figure S64. ^{1}H NMR of Compound **20**.

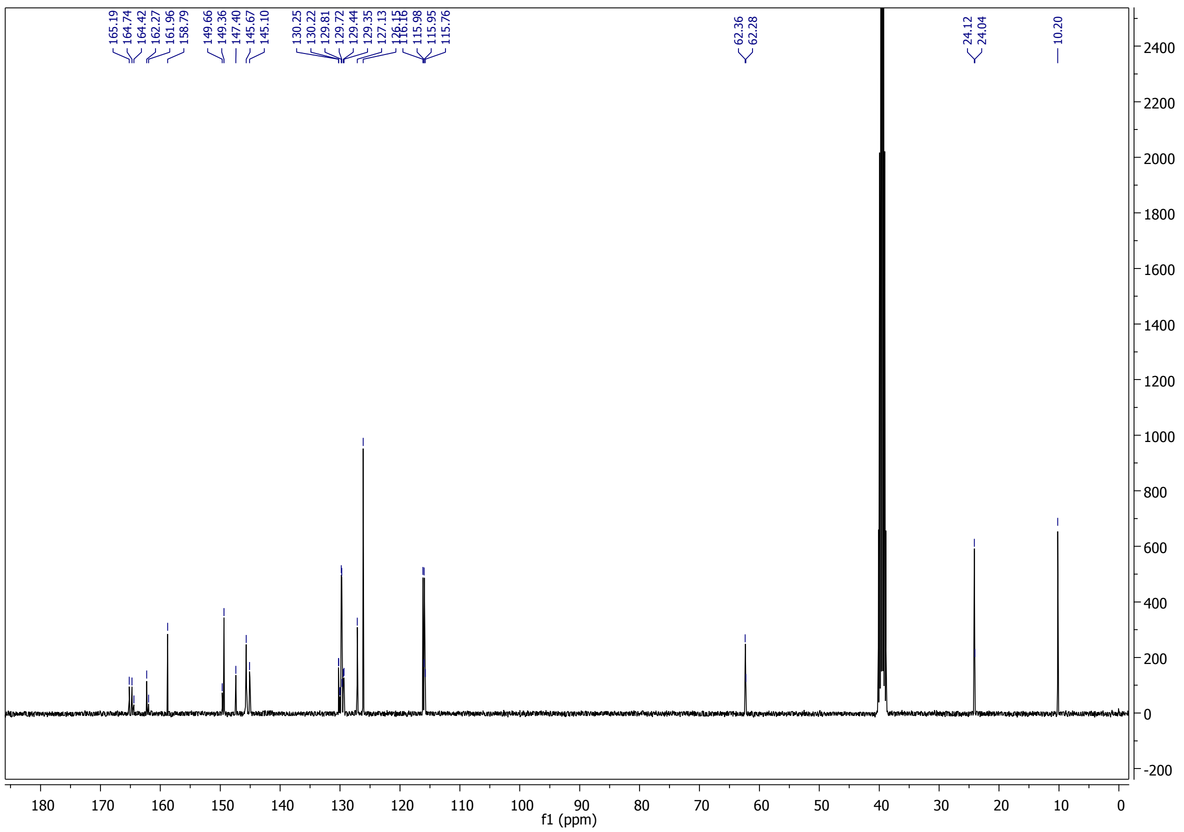

Figure S65. ^{13}C NMR of Compound **20**.

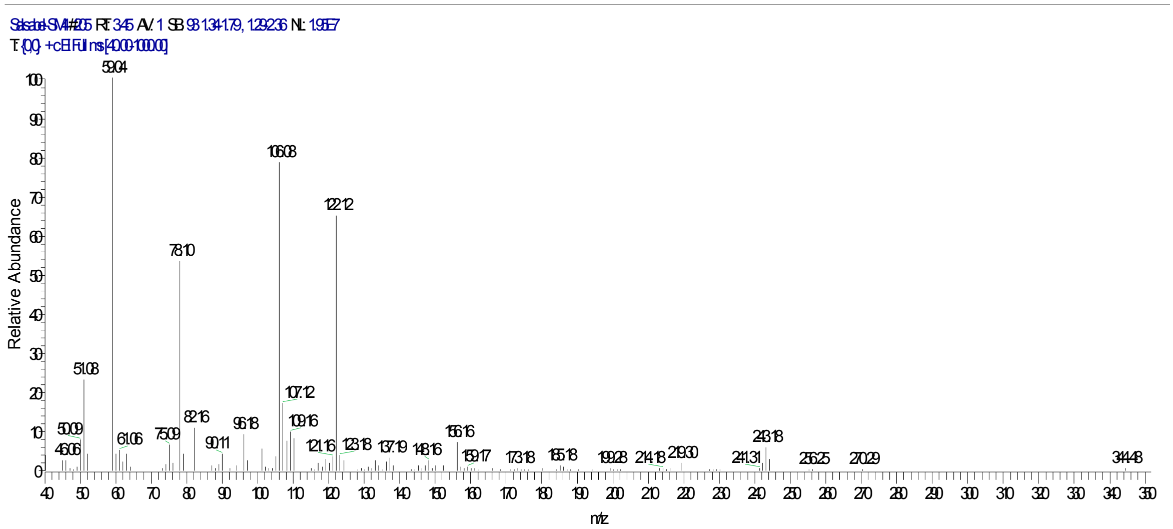

Figure S66. MS (ESI) of Compound **20**.

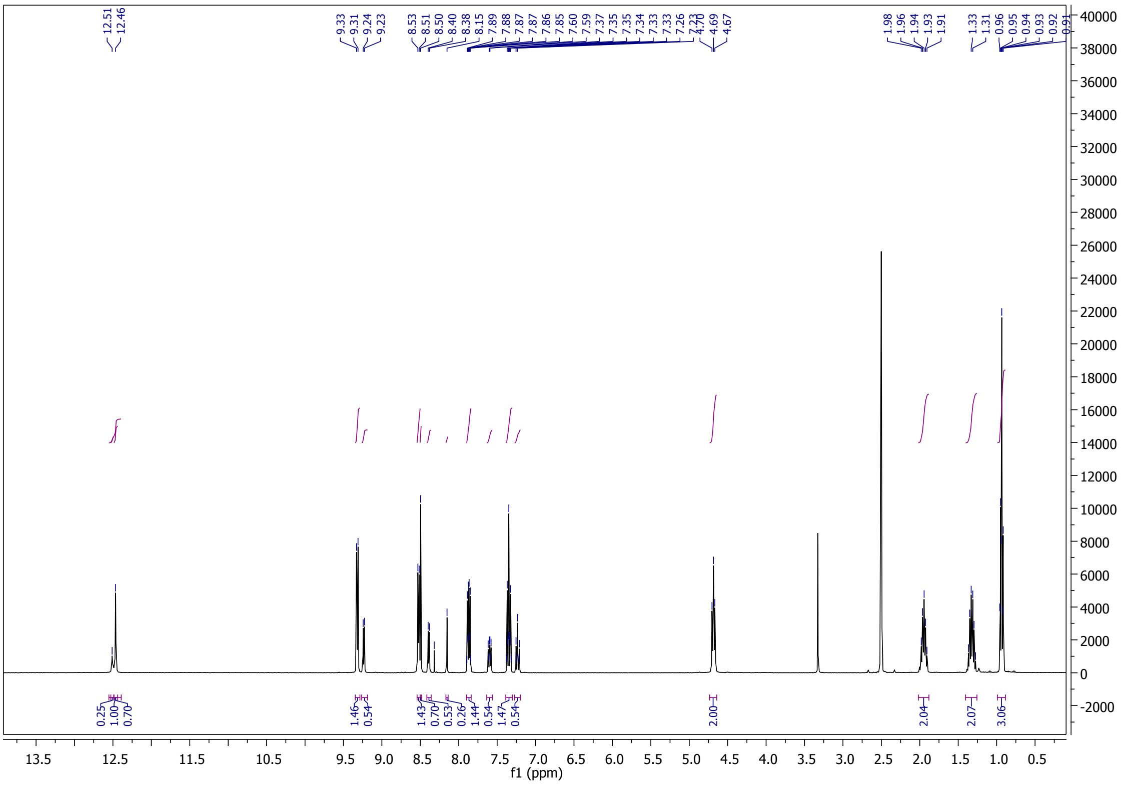

Figure S67. ^{1}H NMR of Compound **21**.

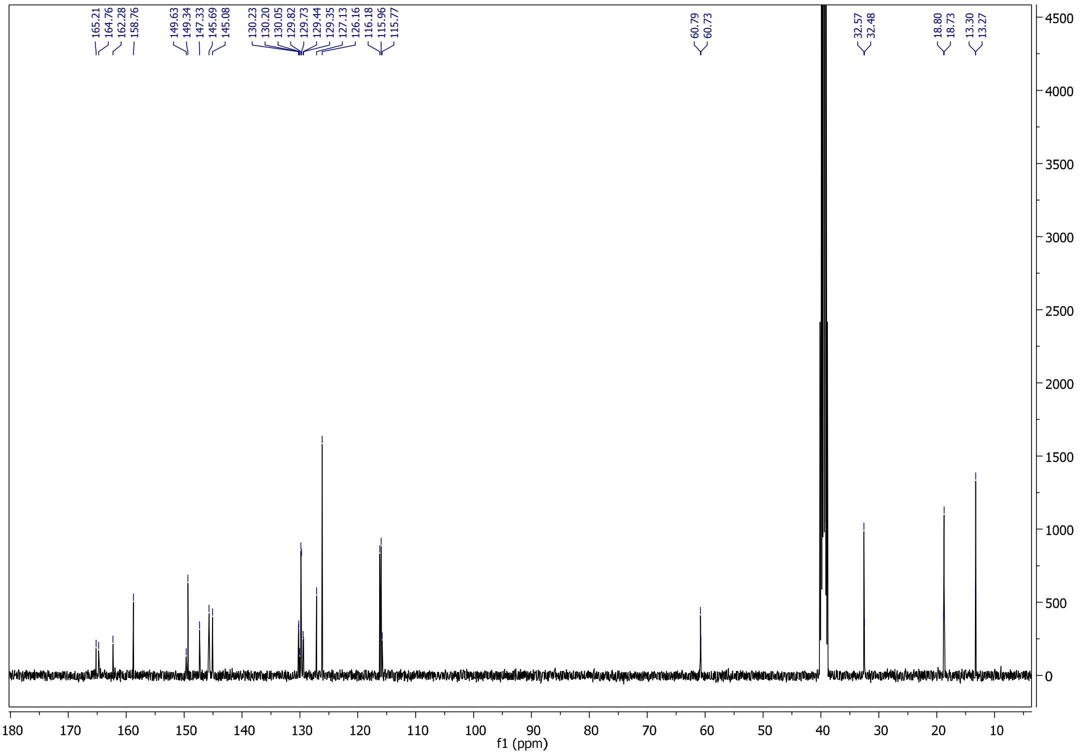

Figure S68. ^{13}C NMR of Compound **21**.

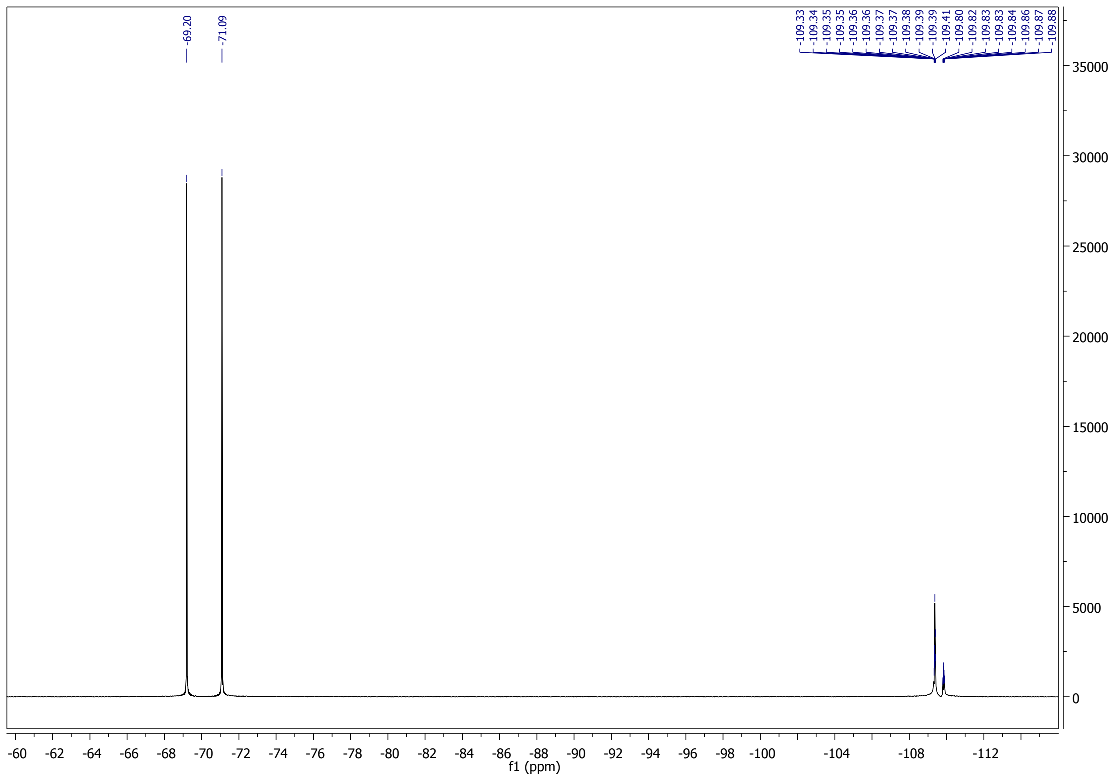

Figure S69. ^{19}F NMR of Compound **21**.

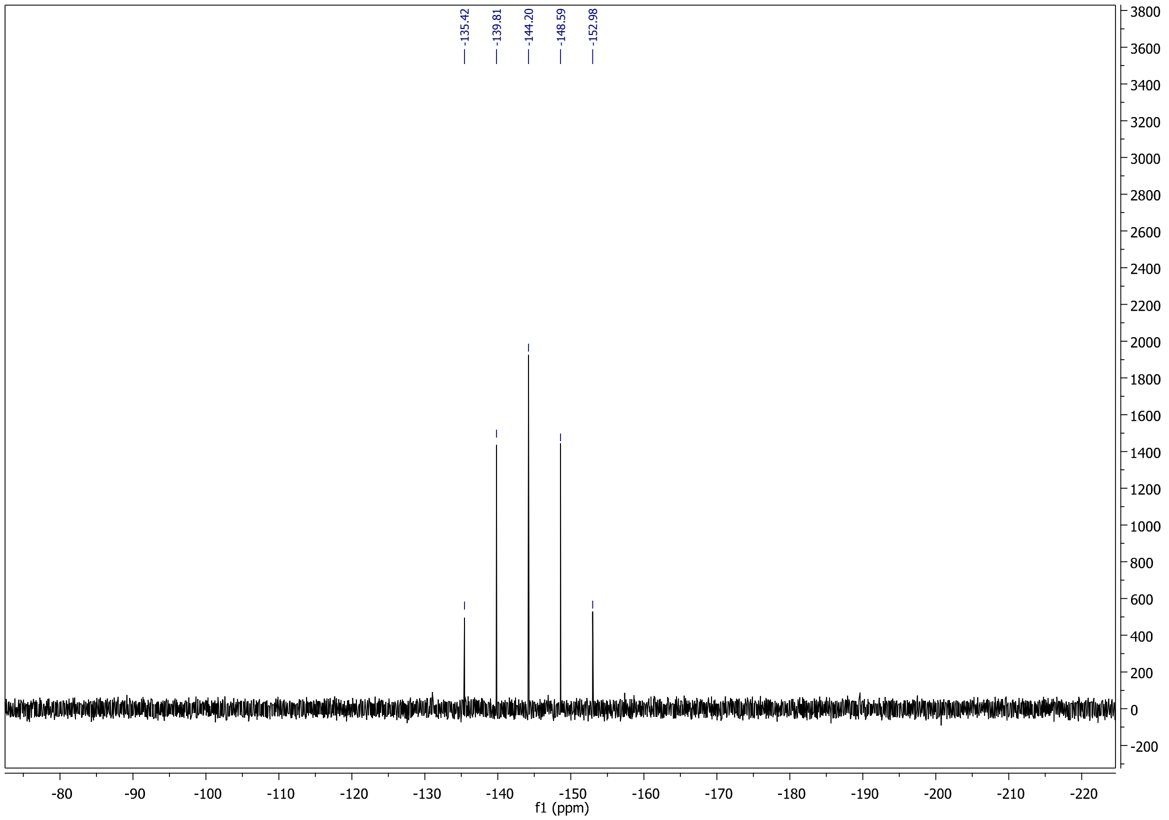

Figure S70. ^{31}P NMR of Compound **21**.

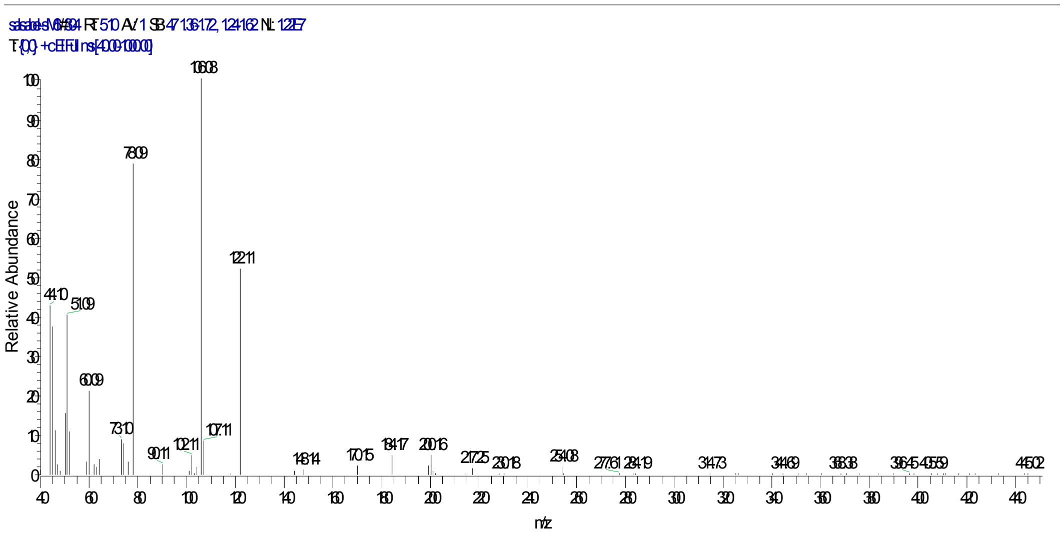

Figure S71. MS (ESI) of Compound **21**.

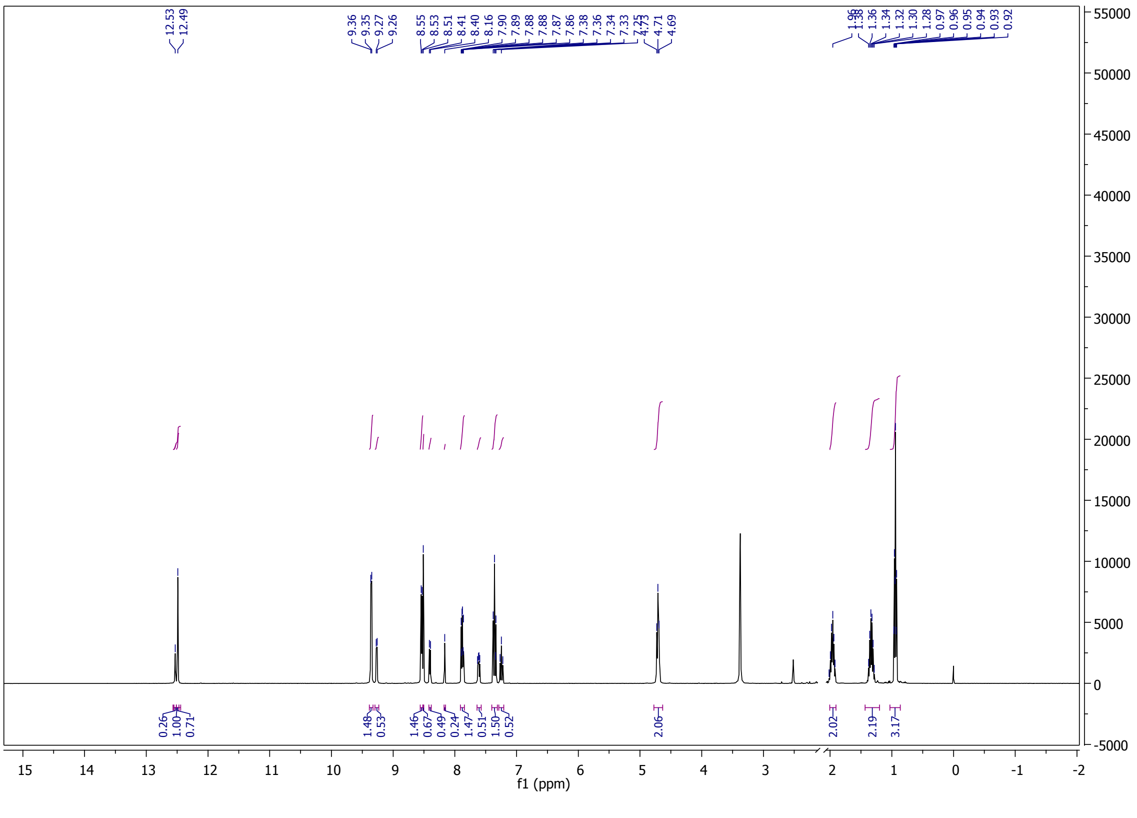

Figure S72. ^{1}H NMR of Compound **22**.

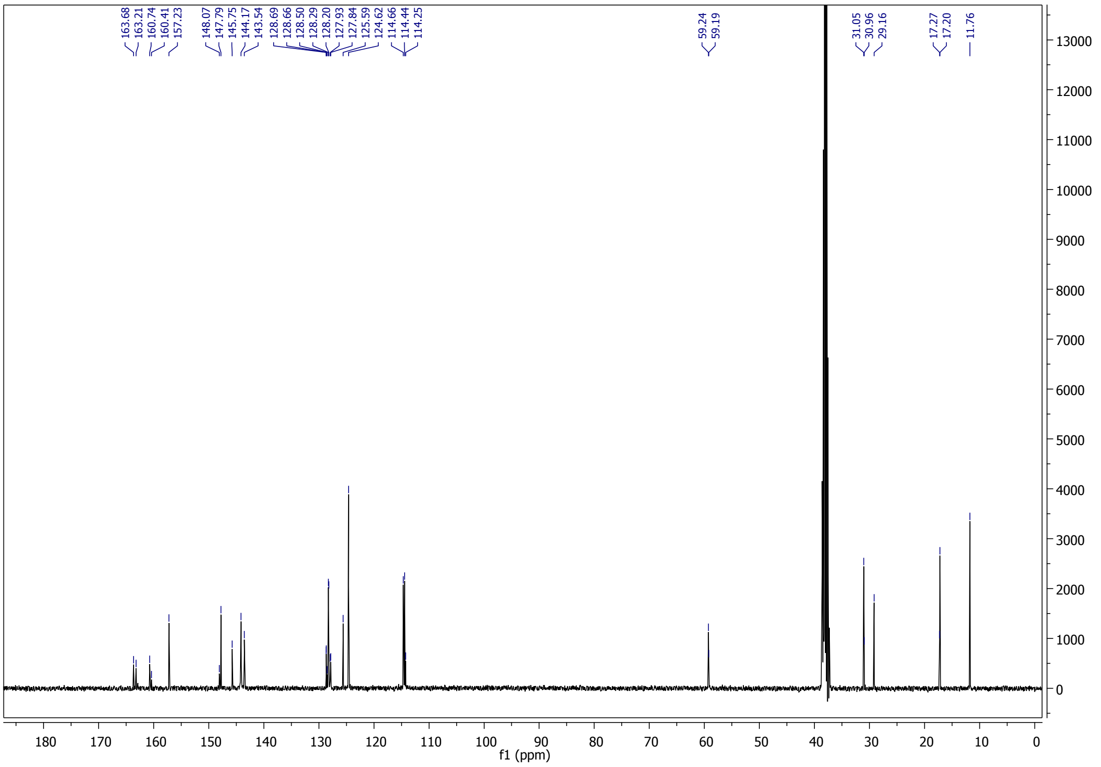

Figure S73. ^{13}C NMR of Compound **22**.

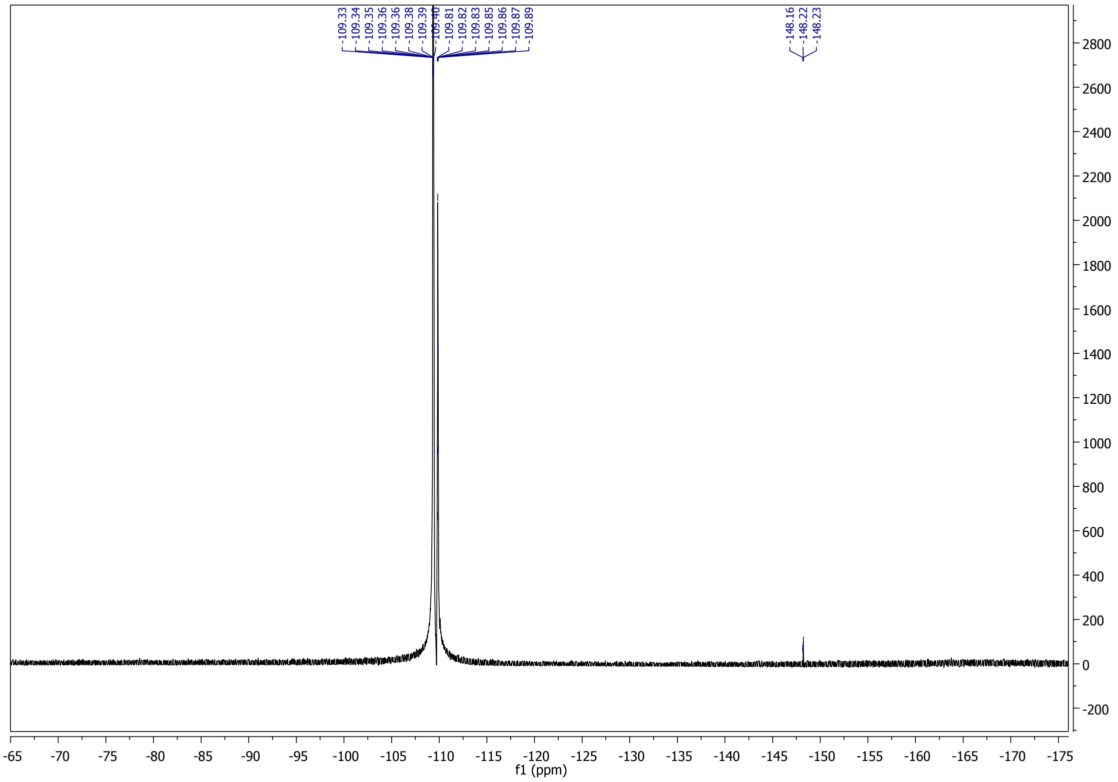

Figure S74. ^{19}F NMR of Compound **22**.

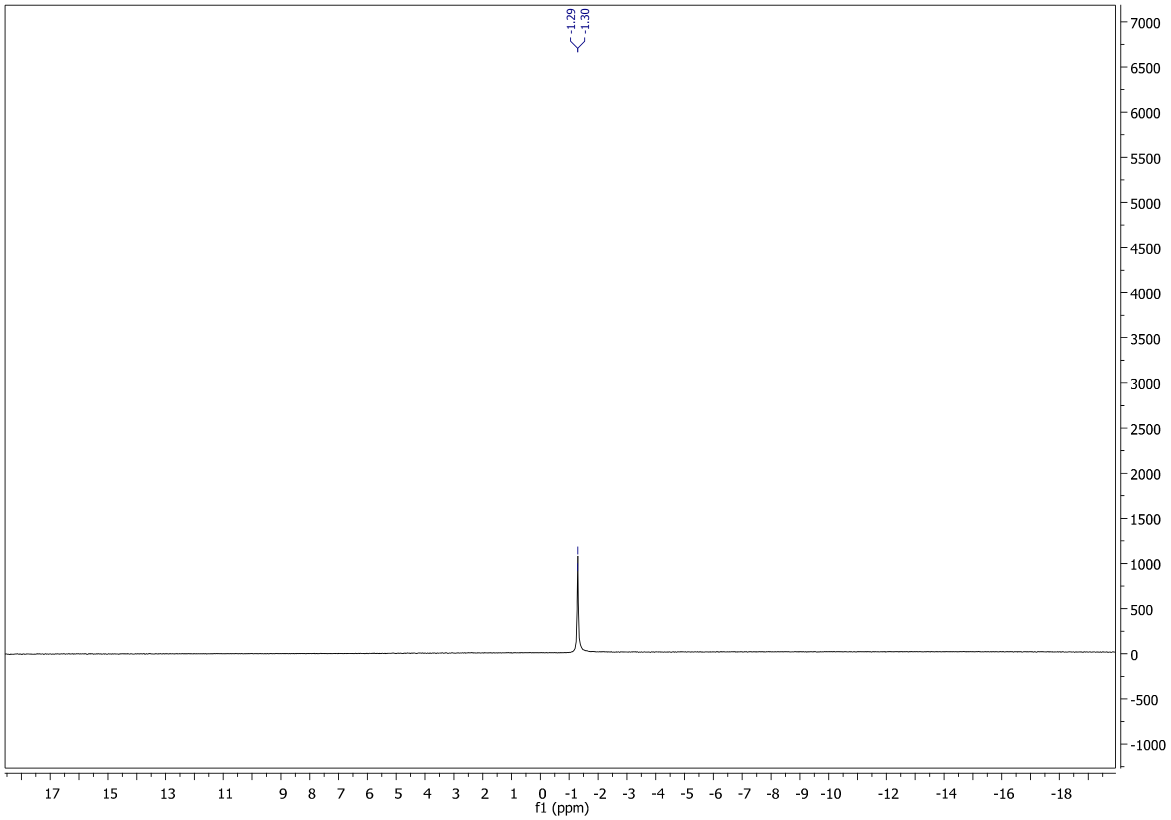

Figure S75. ^{11}B NMR of Compound **22**.

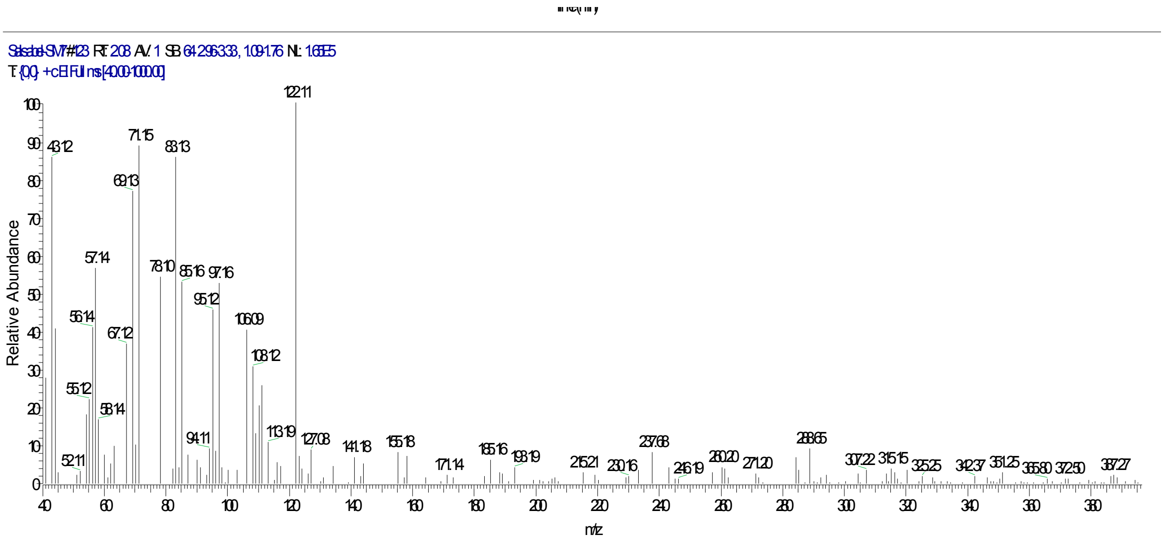

Figure S76. MS (ESI) of Compound **22**.

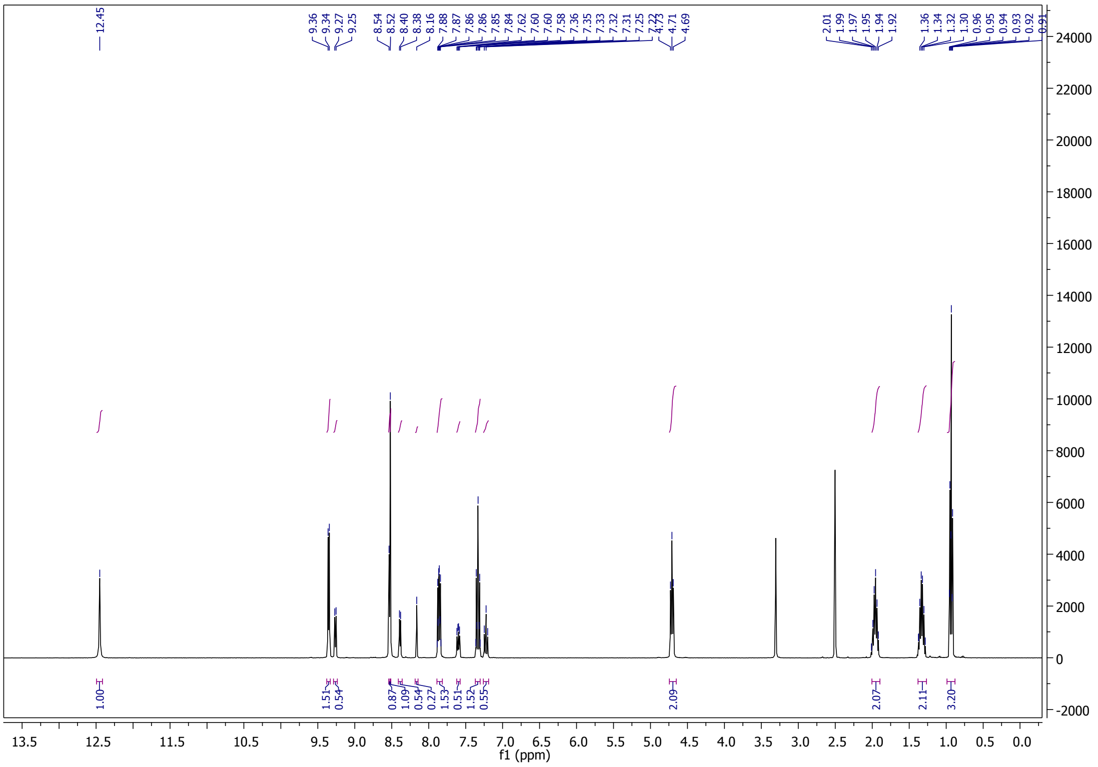

Figure S77. ^{1}H NMR of Compound **23**.

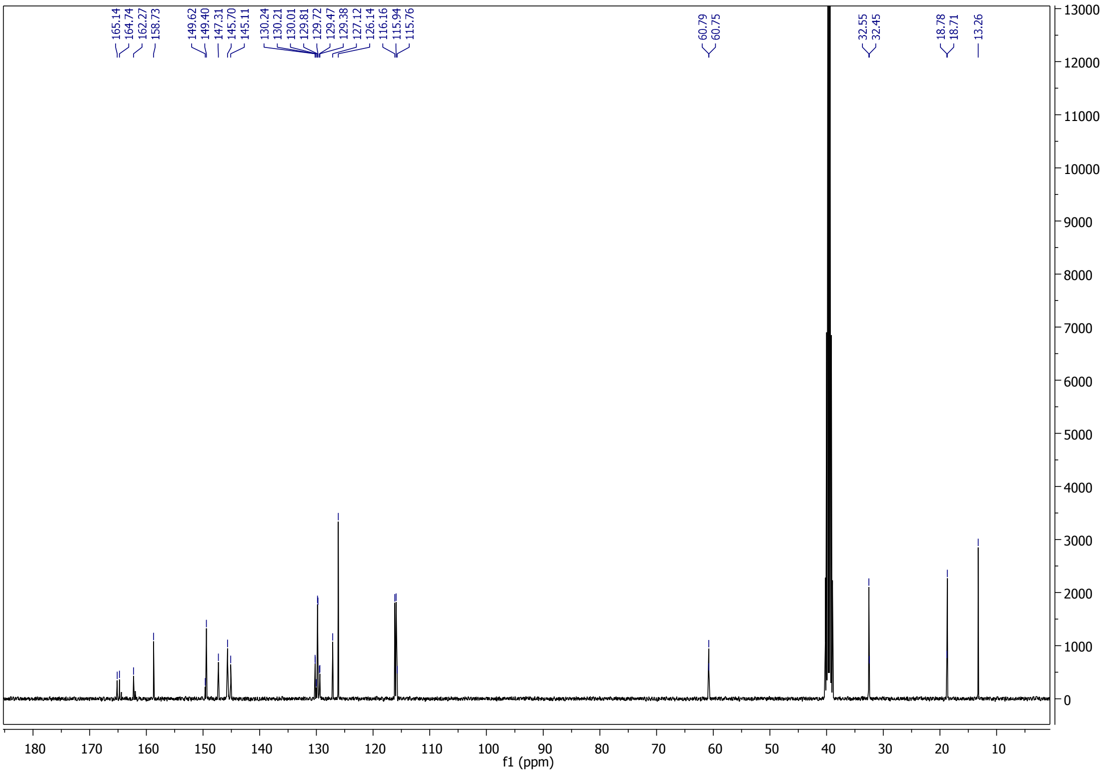

Figure S78. ^{13}C NMR of Compound **23**.

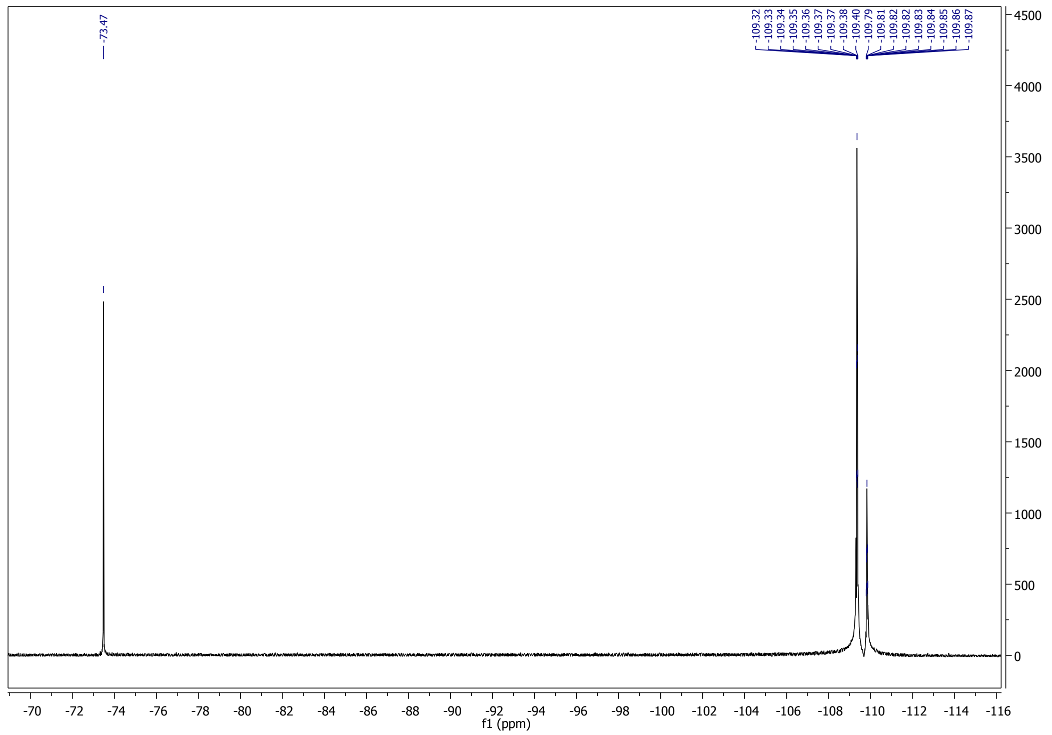

Figure S79. ^{19}F NMR of Compound **23**.

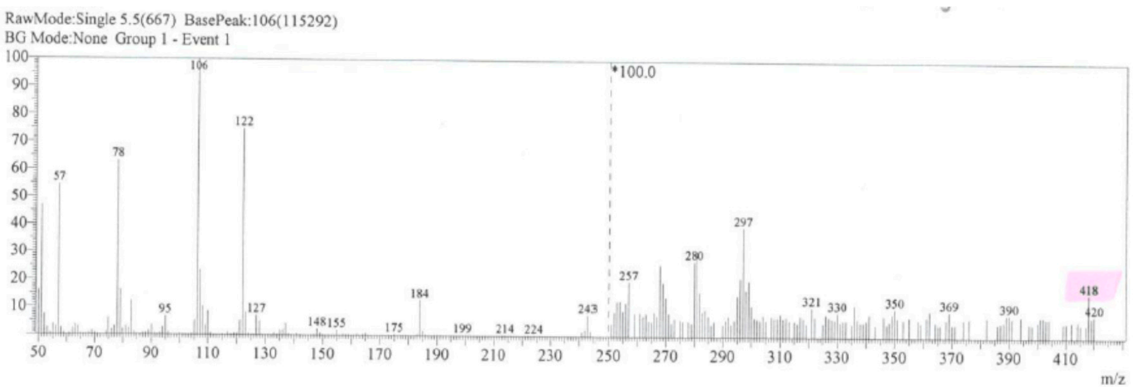

Figure S80. MS (ESI) of Compound **23**.

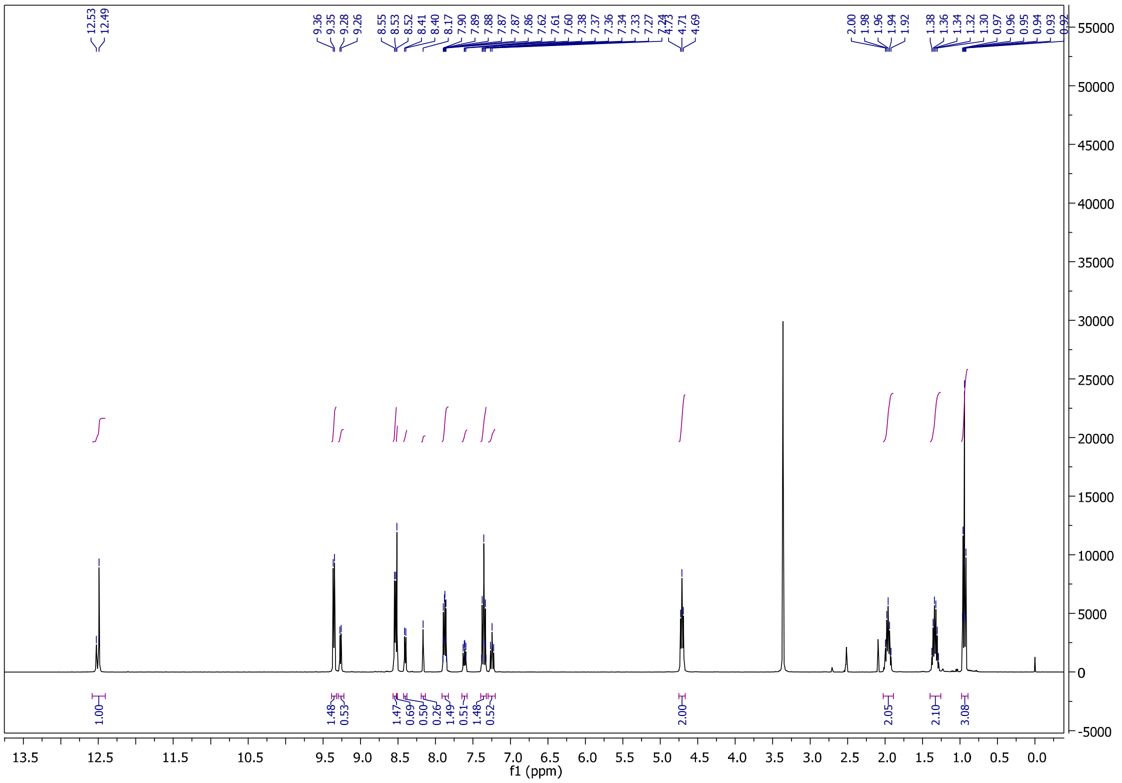

Figure S81. ^{1}H NMR of Compound **24**.

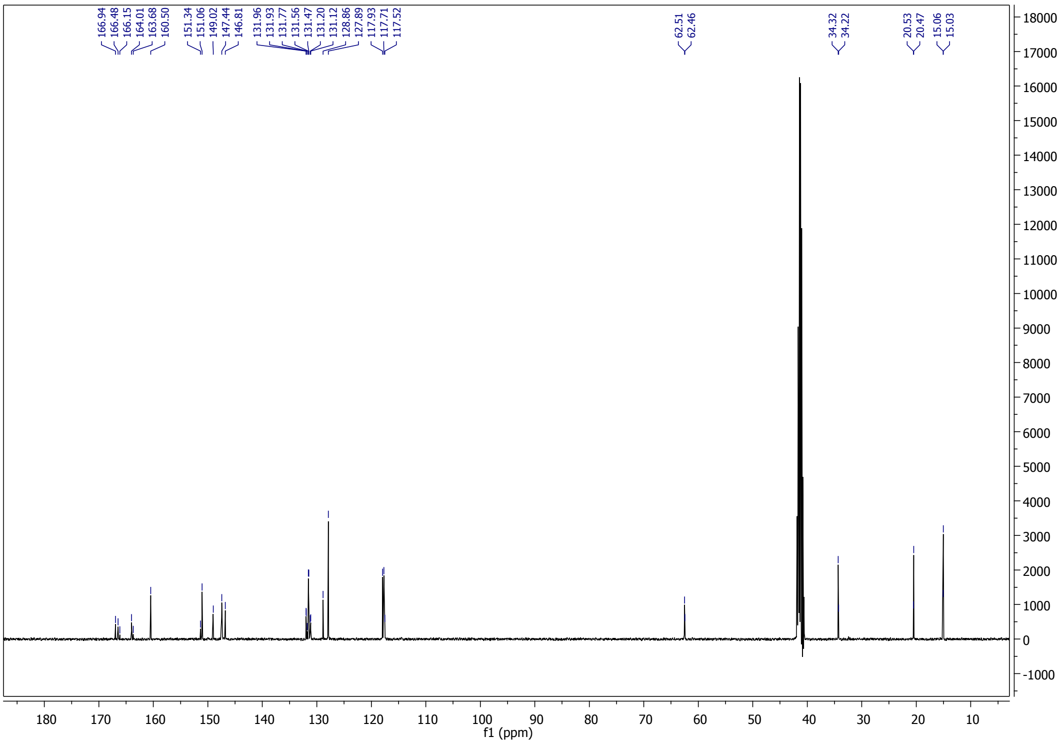

Figure S82. ^{13}C NMR of Compound **24**.

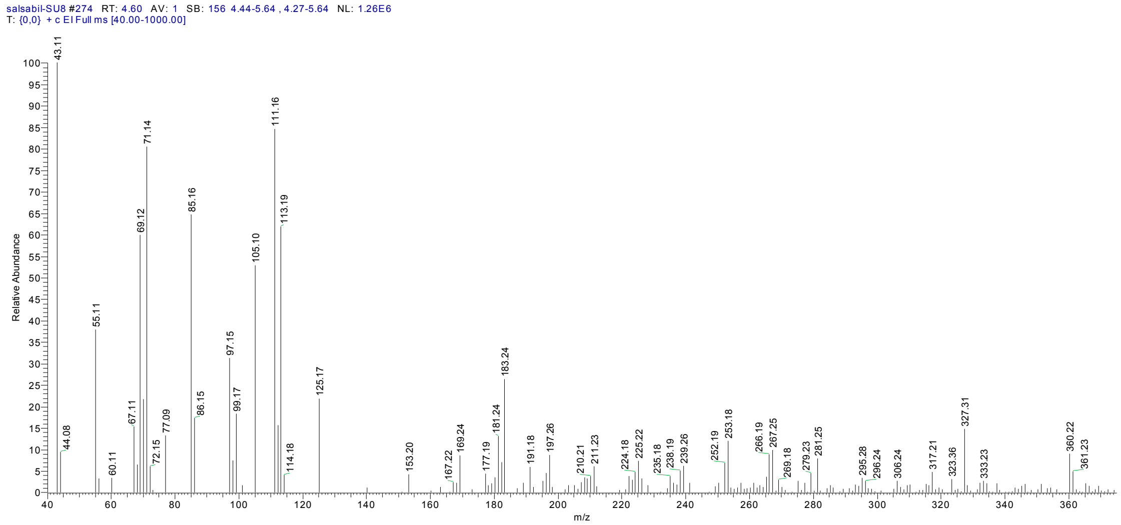

Figure S83. MS (ESI) of Compound **24**.

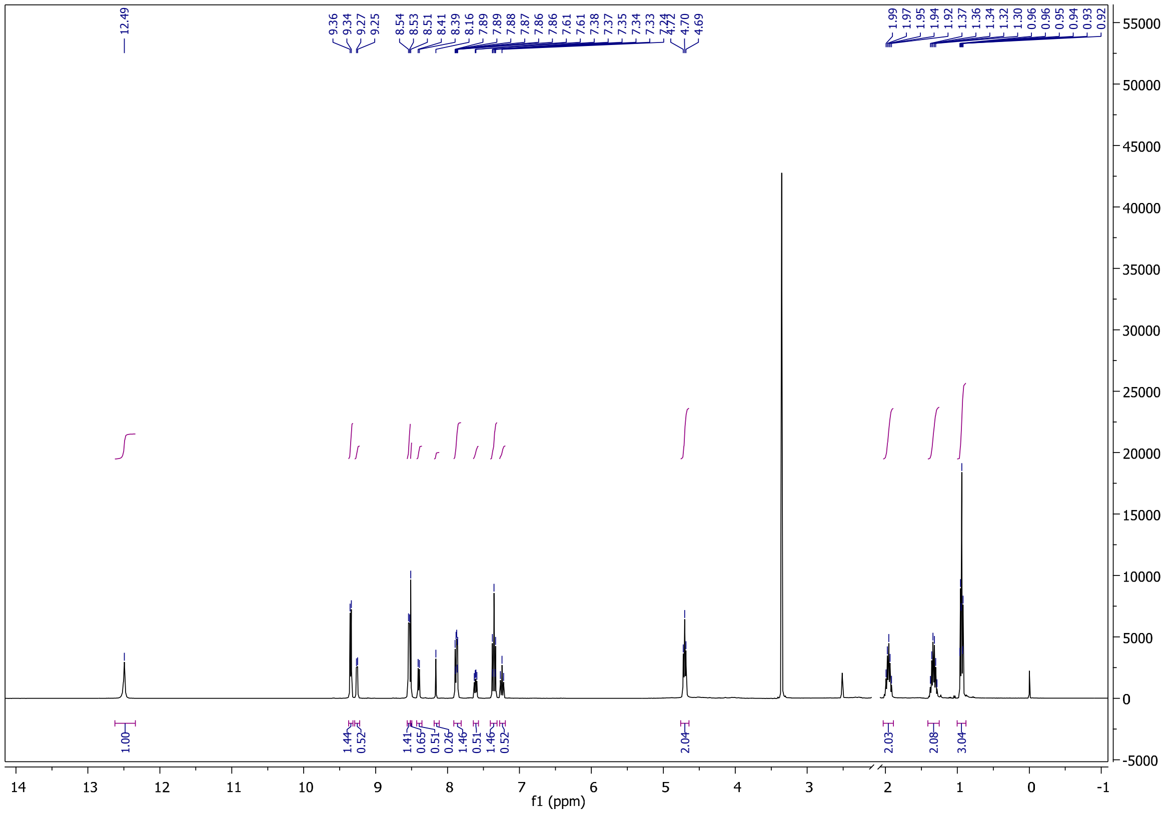

Figure S84. ^{1}H NMR of Compound **25**.

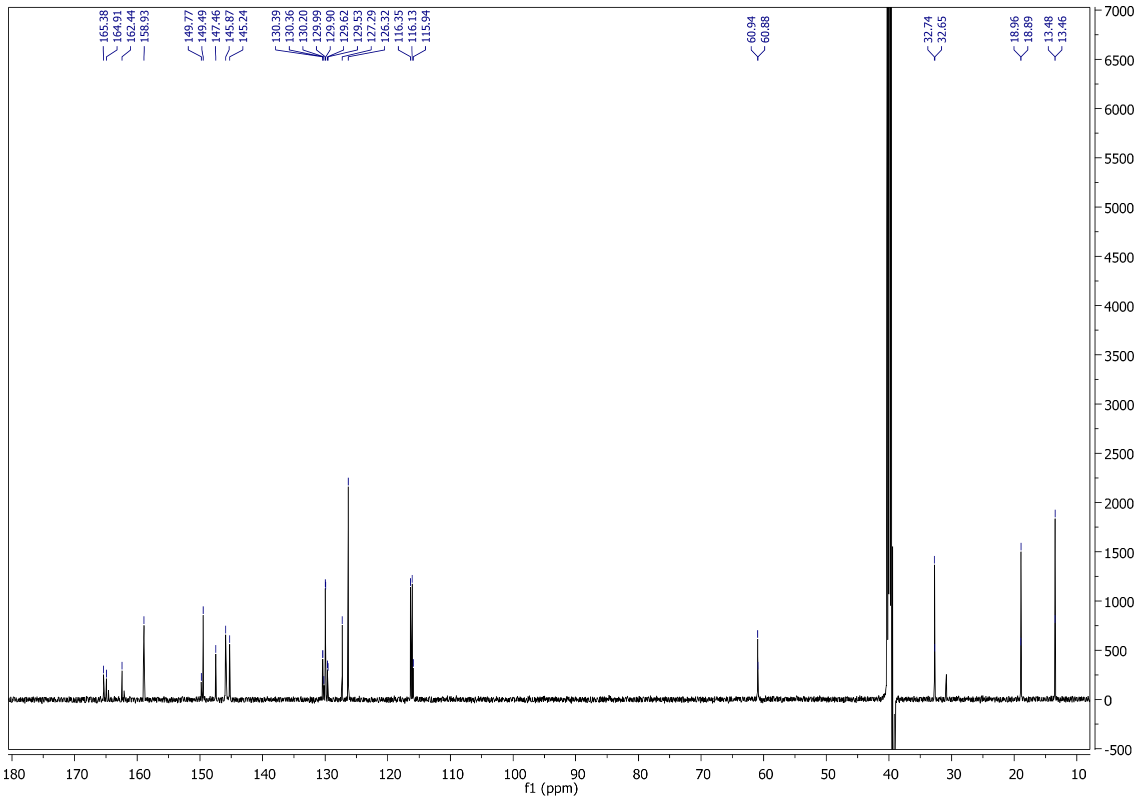

Figure S85. ^{13}C NMR of Compound **25**.

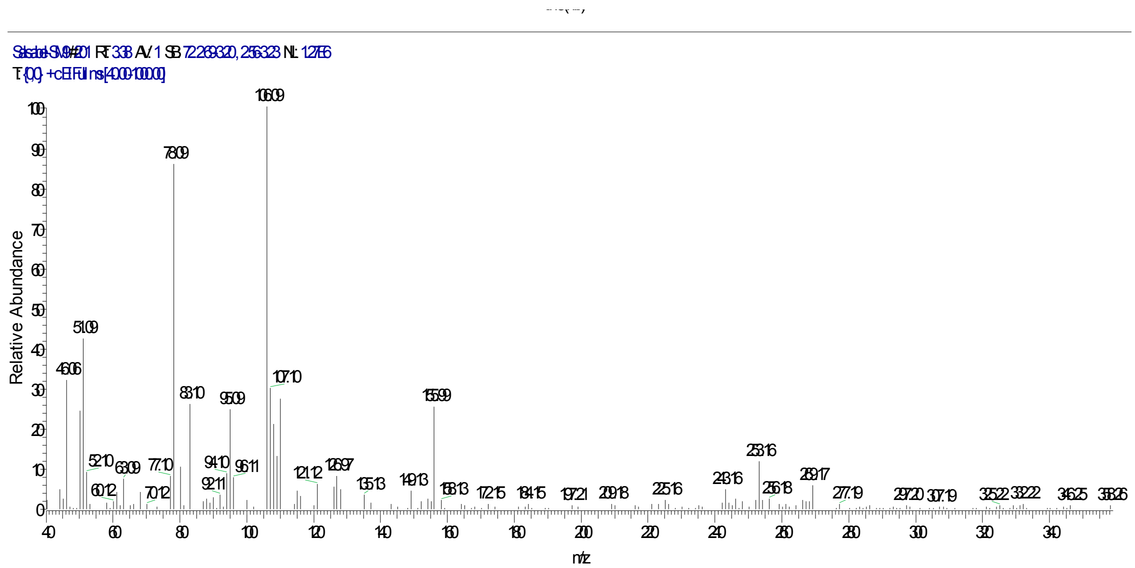

Figure S86. MS (ESI) of Compound **25**.

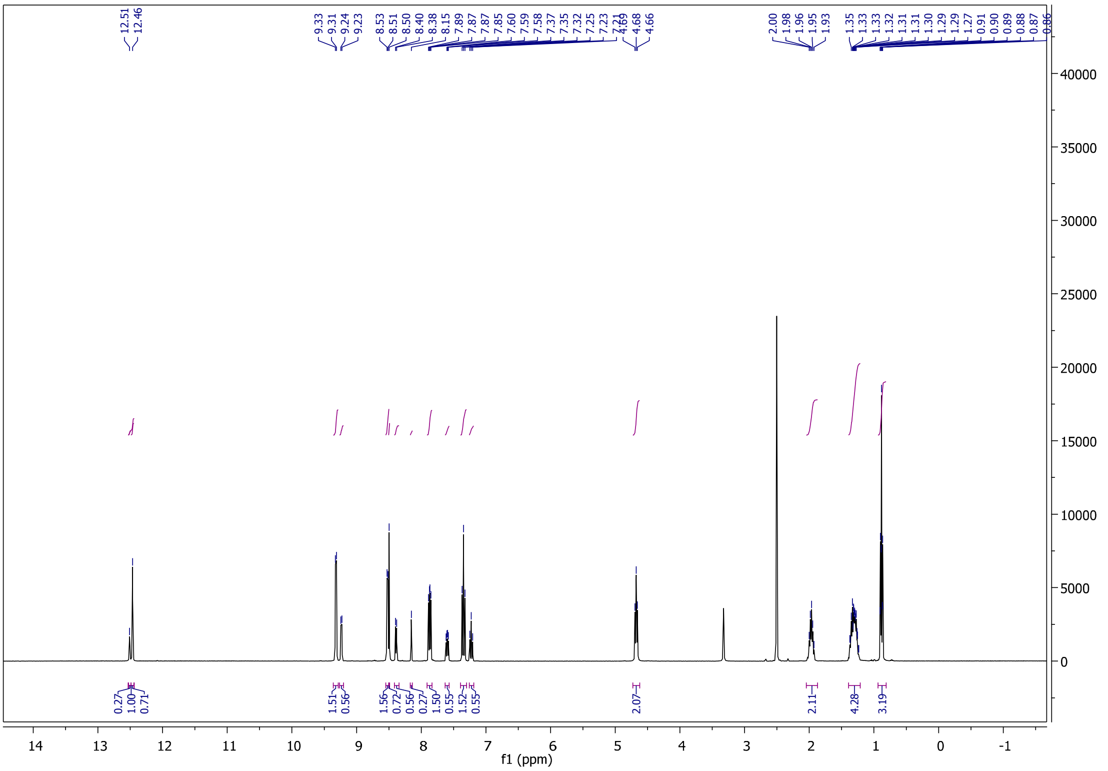

Figure S87. ^{1}H NMR of Compound **26**.

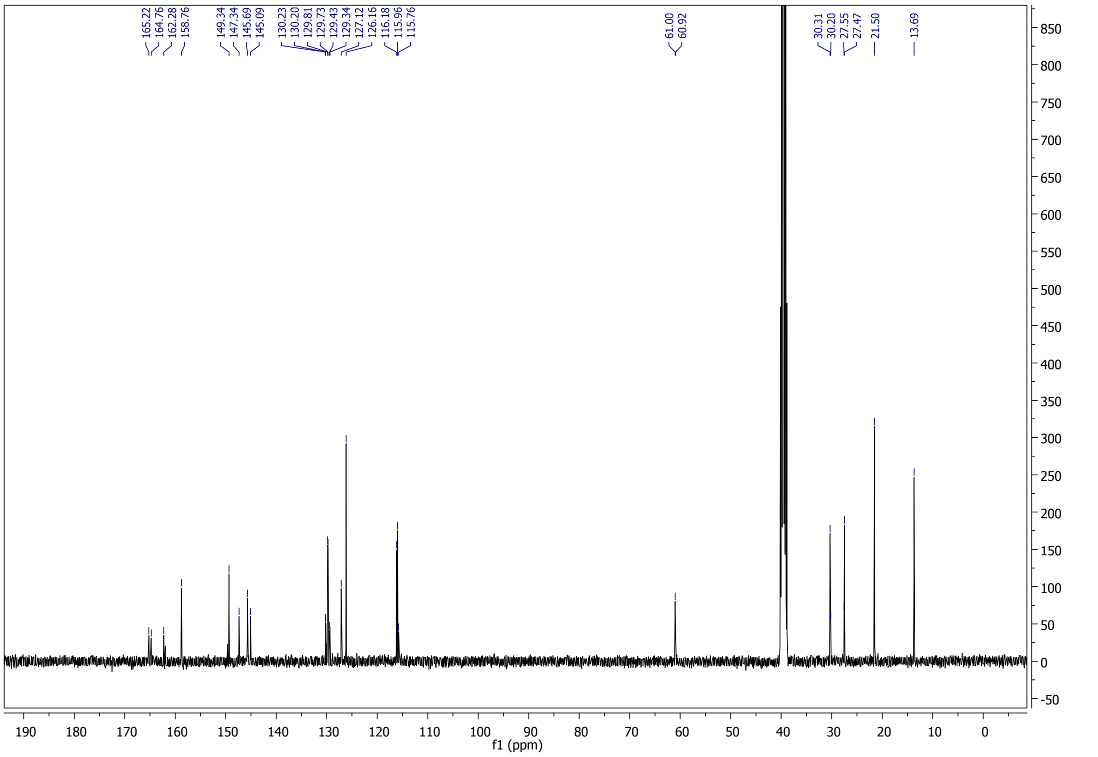

Figure S88. ^{13}C NMR of Compound **26**.

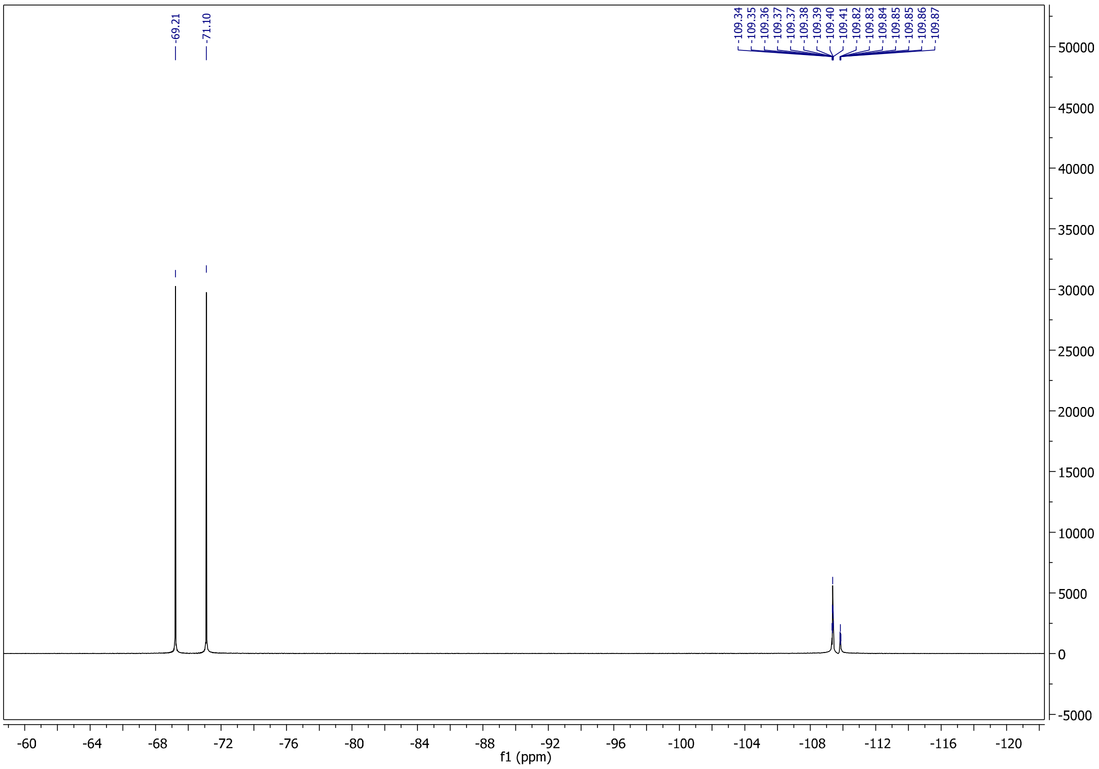

Figure S89. ^{19}F NMR of Compound **26**.

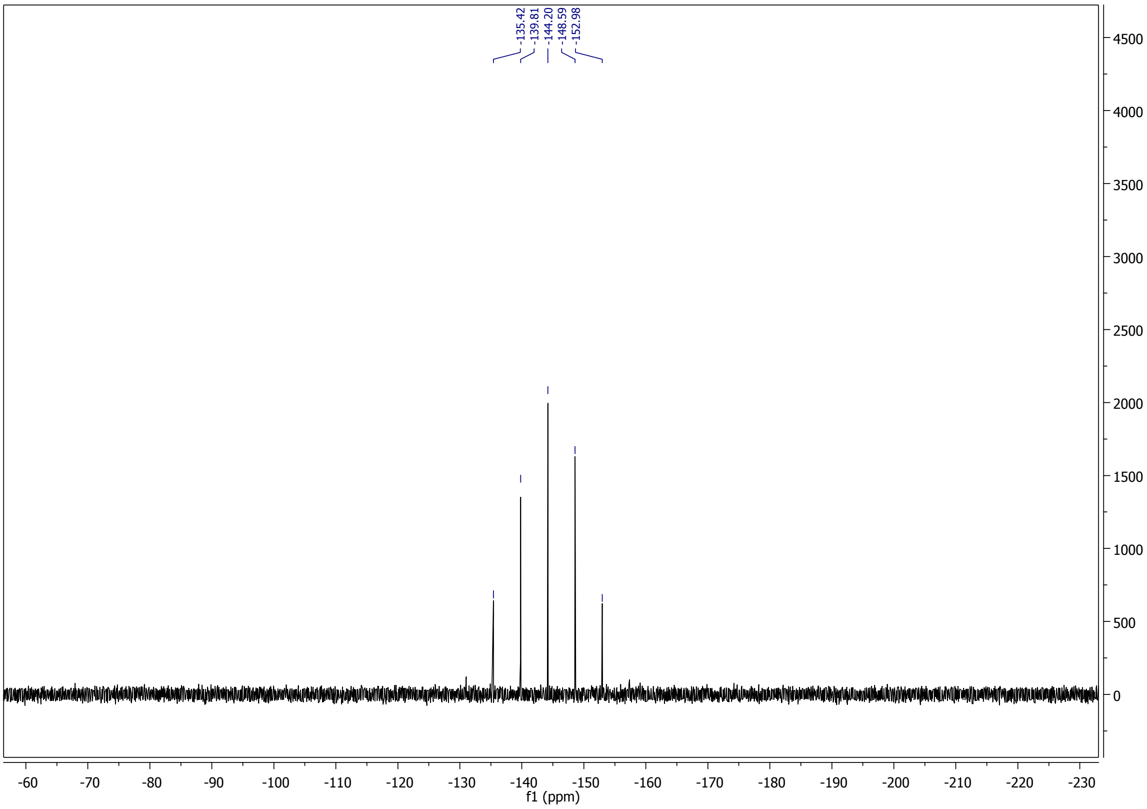

Figure S90. ^{31}P NMR of Compound **26**.

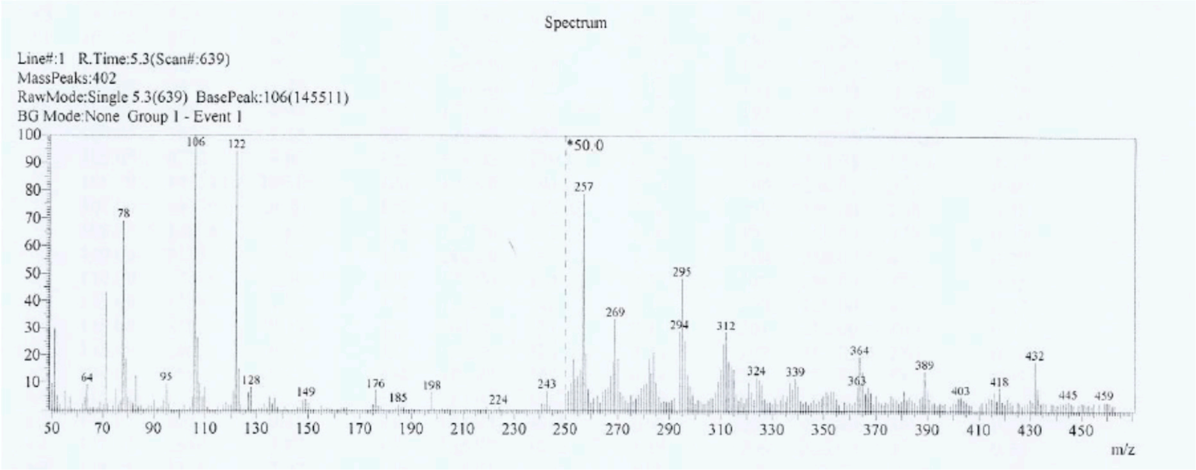

Figure S91. MS (ESI) of Compound **26**.

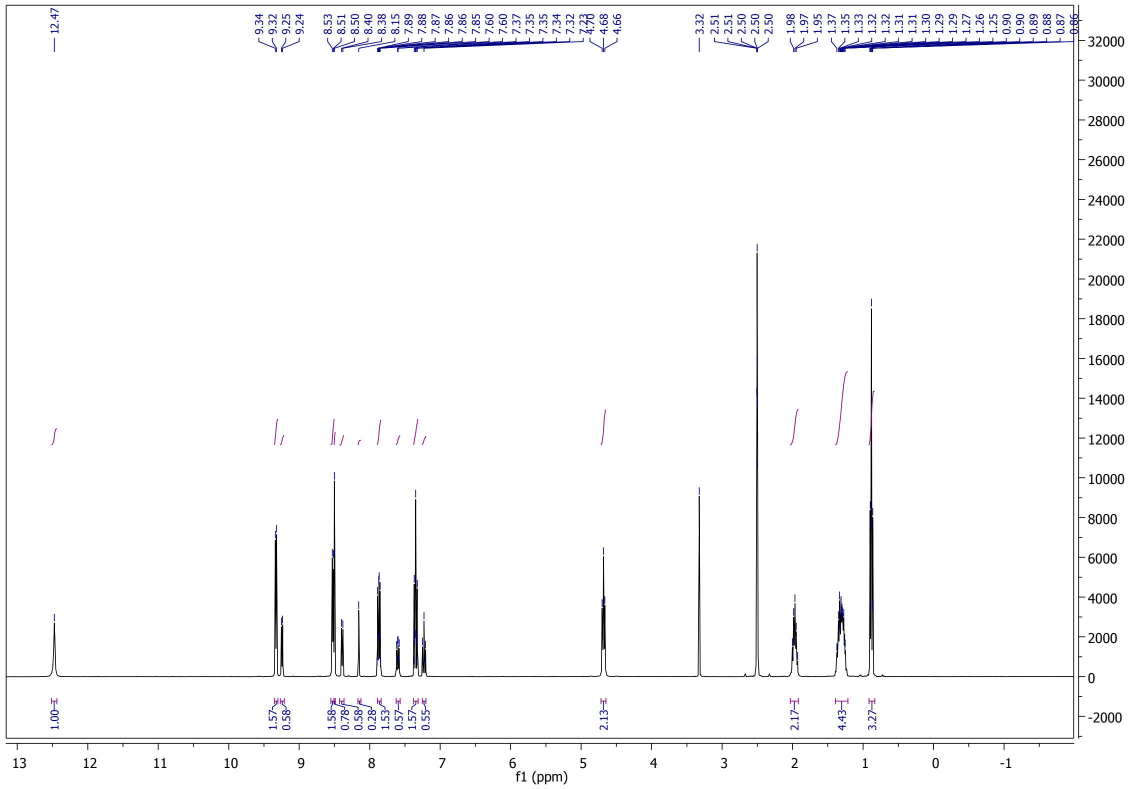

Figure S92. ^{1}H NMR of Compound **27**.

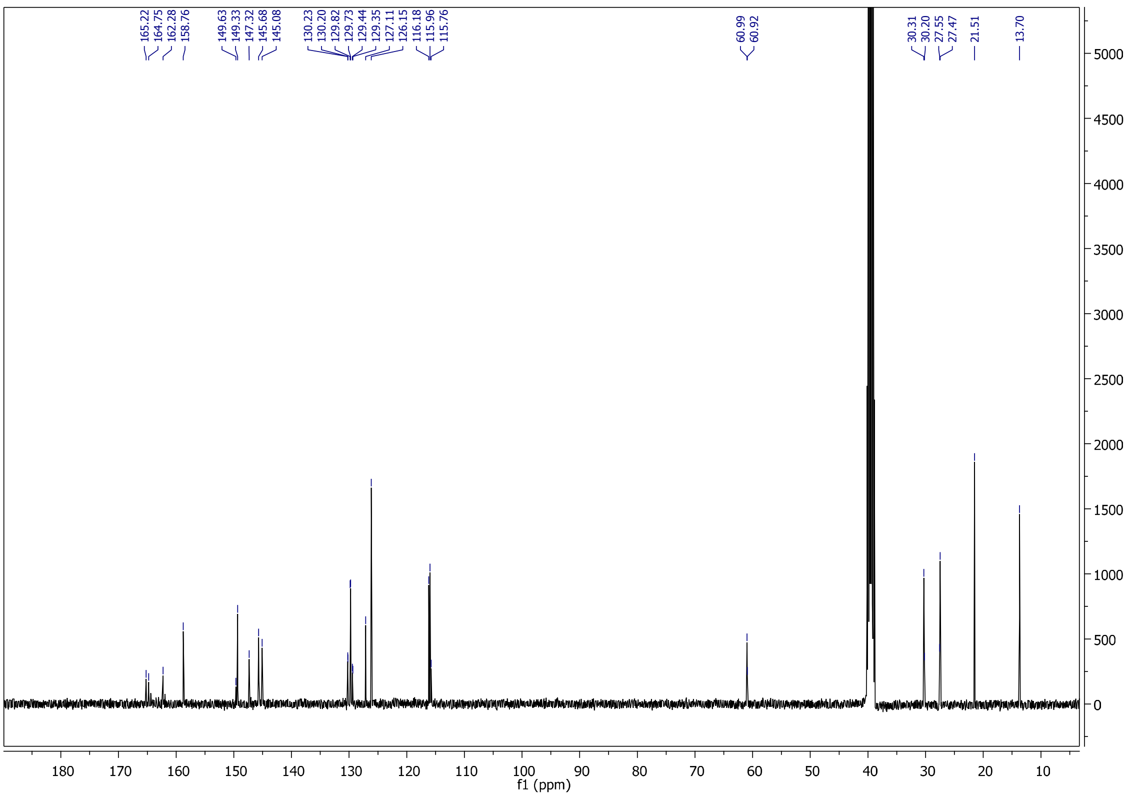

Figure S93. ^{13}C NMR of Compound **27**.

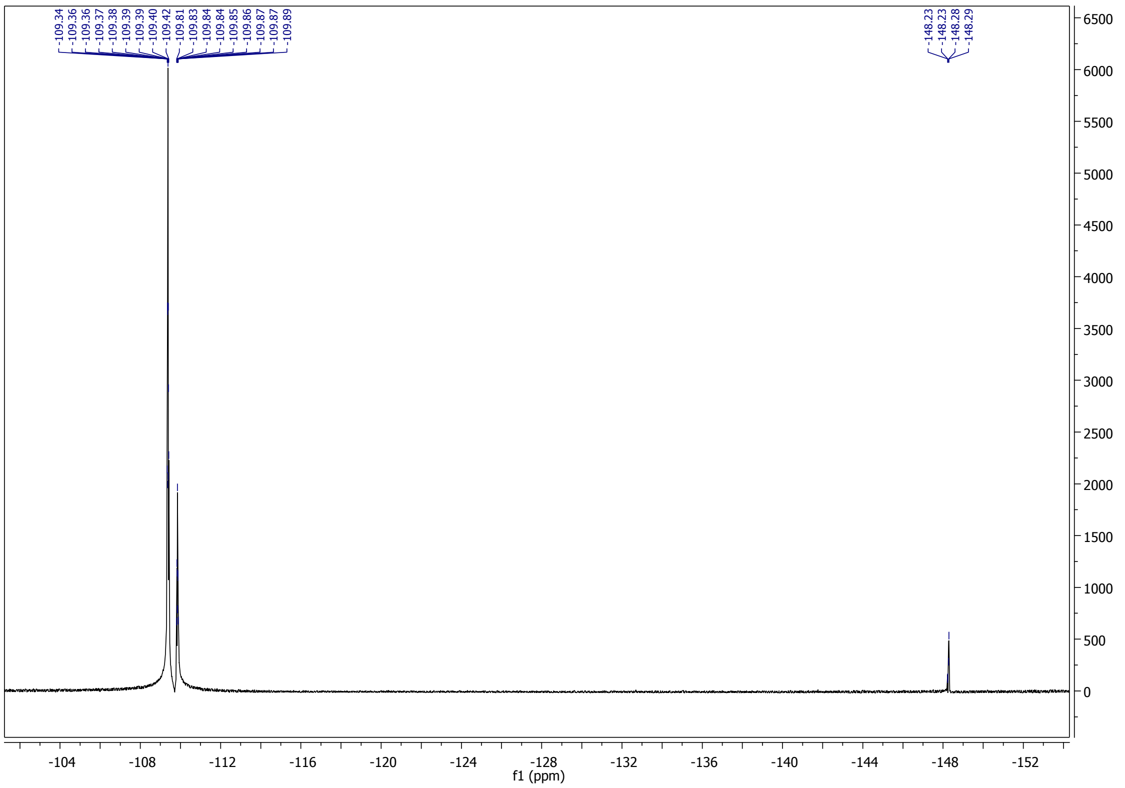

Figure S94. ^{19}F NMR of Compound **27**.

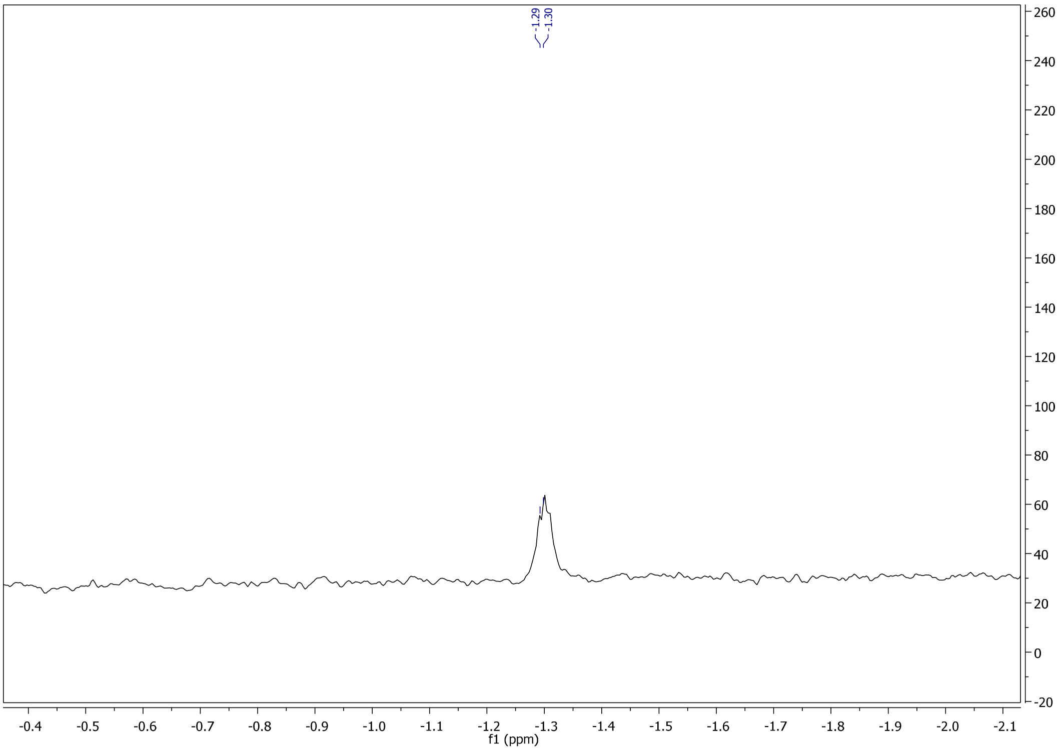

Figure S95. ^{11}B NMR of Compound **27**.

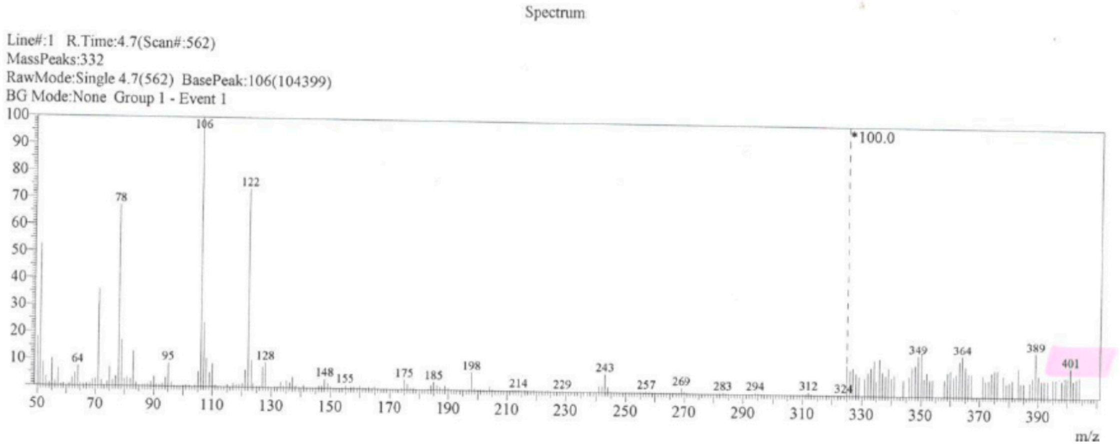

Figure S96. MS (ESI) of Compound **27**.

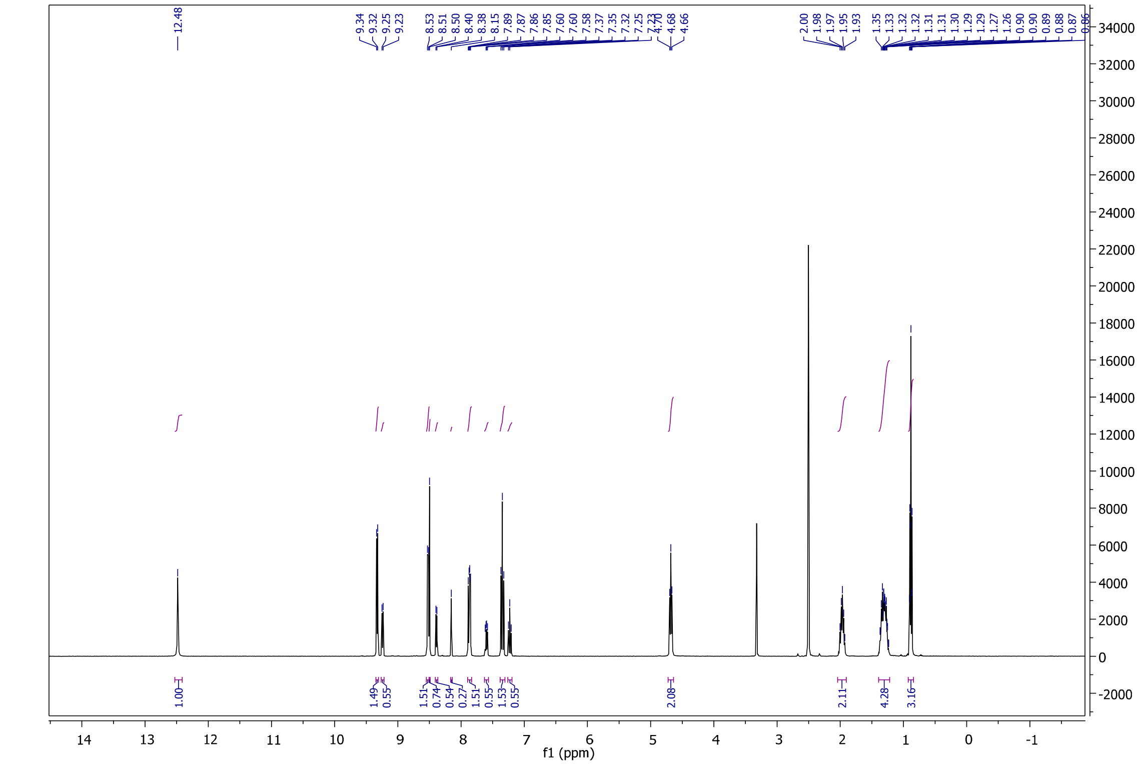

Figure S97. ^{1}H NMR of Compound **28**.

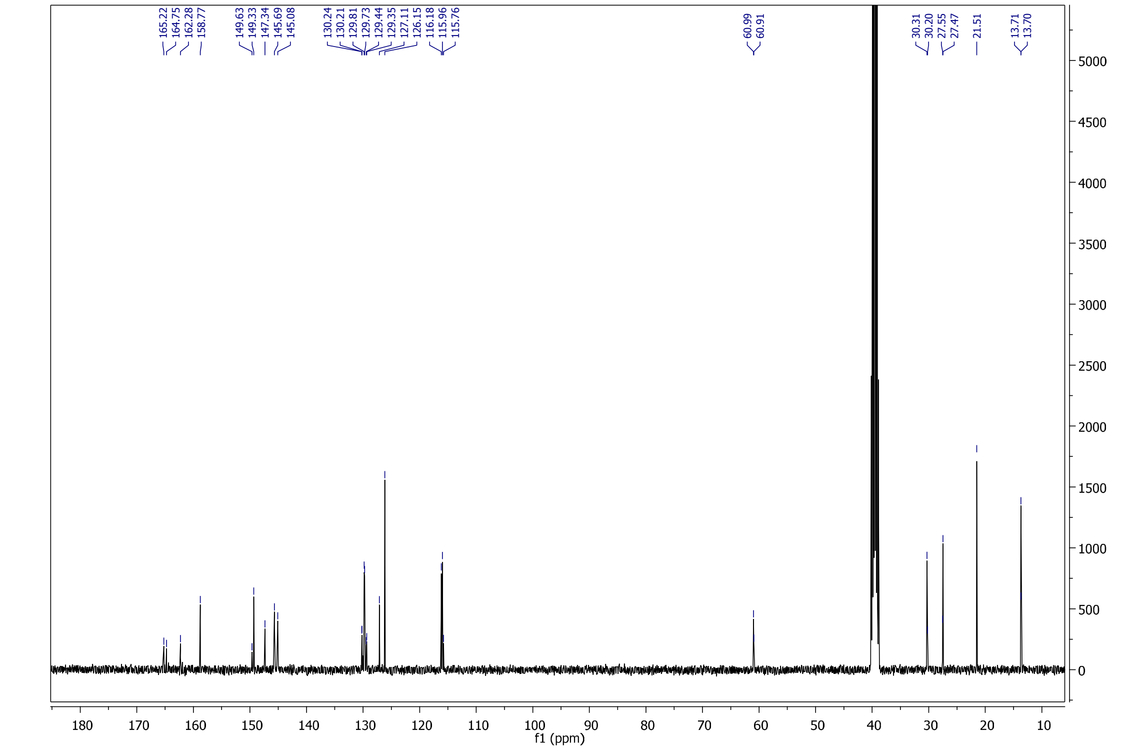

Figure S98. ^{13}C NMR of Compound **28**.

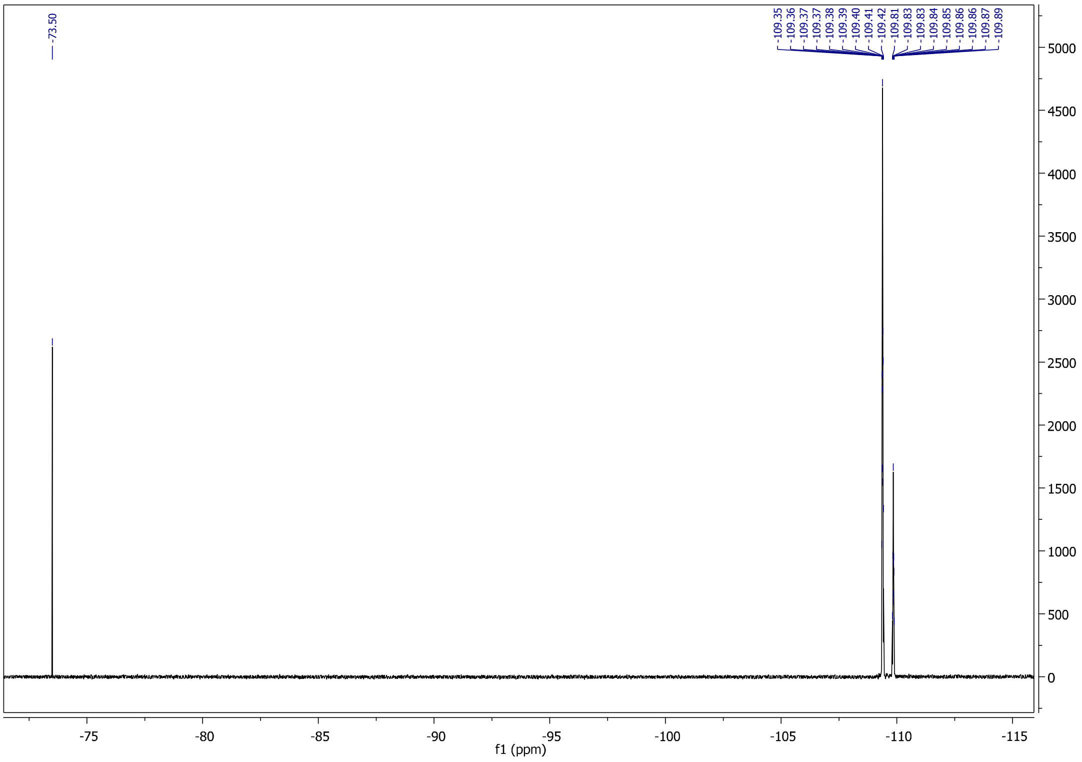

Figure S99. ^{19}F NMR of Compound **28**.

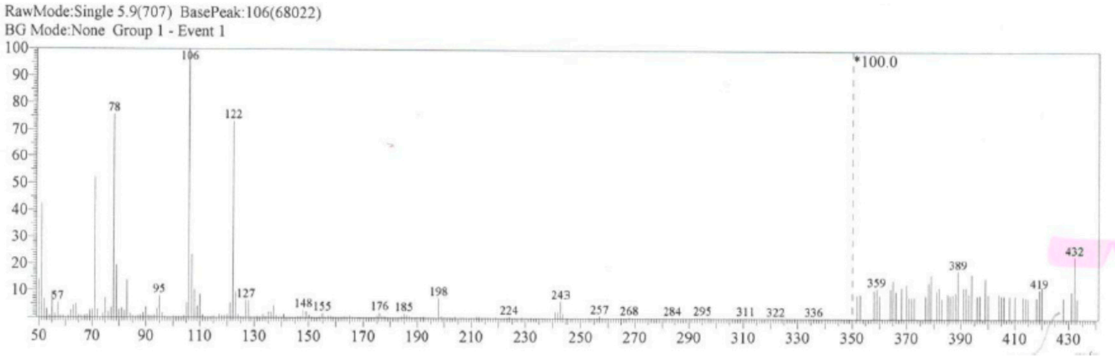

Figure S100. MS (ESI) of Compound **28**.

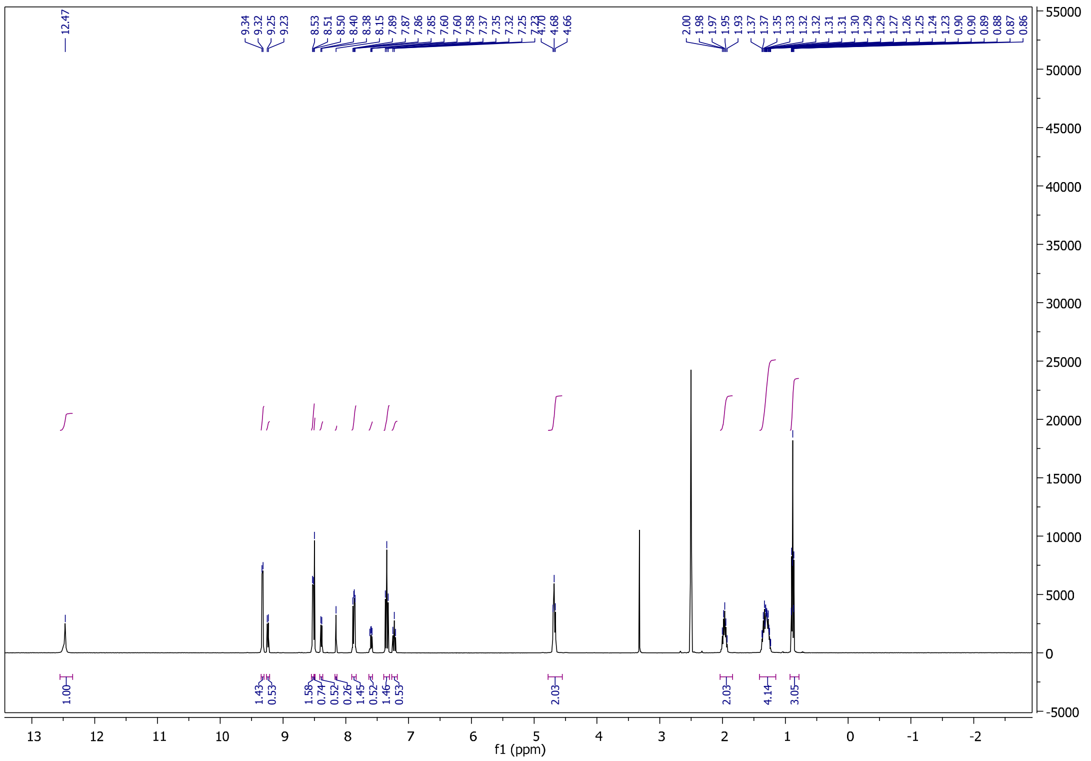

Figure S101. ^{1}H NMR of Compound **29**.

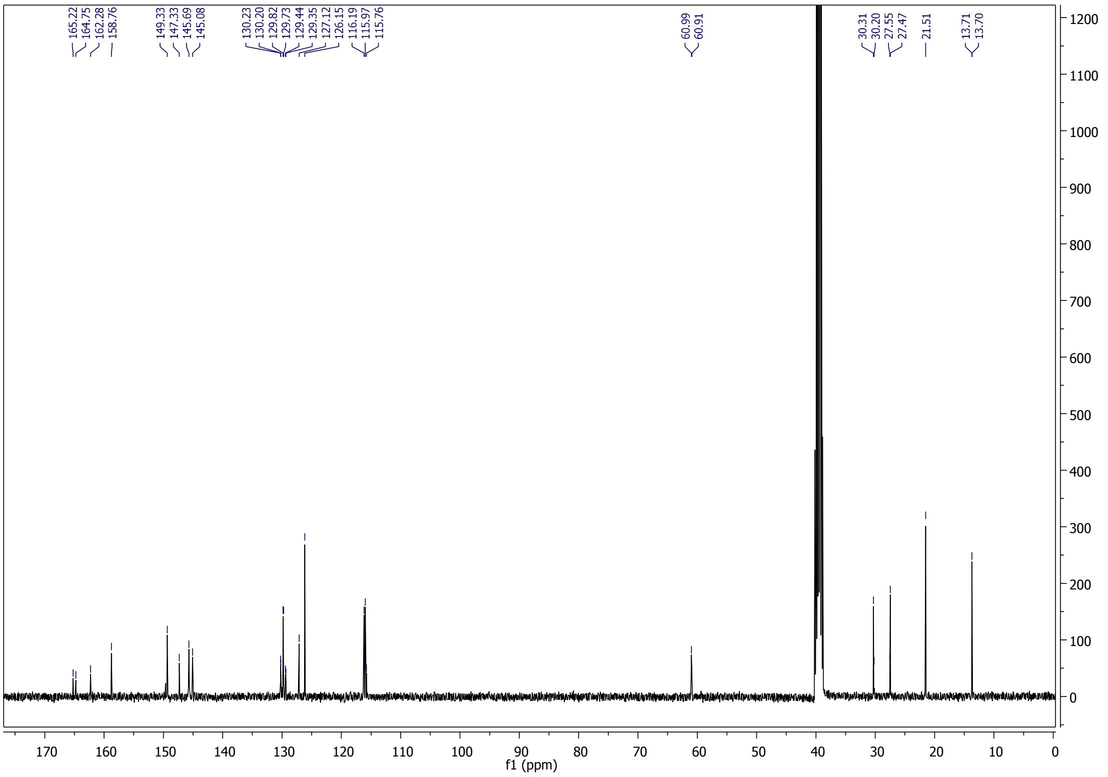

Figure S102. ^{13}C NMR of Compound **29**.

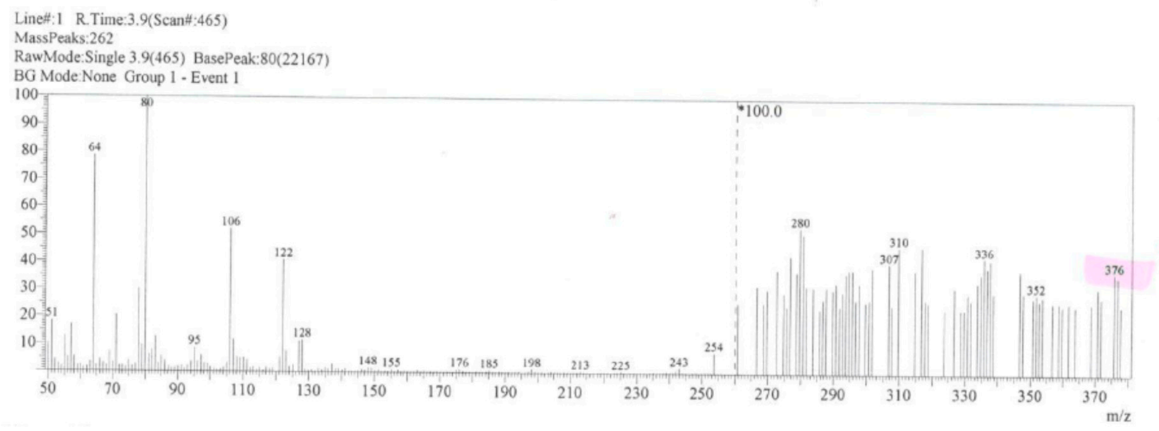

Figure S103. MS (ESI) of Compound **29**.

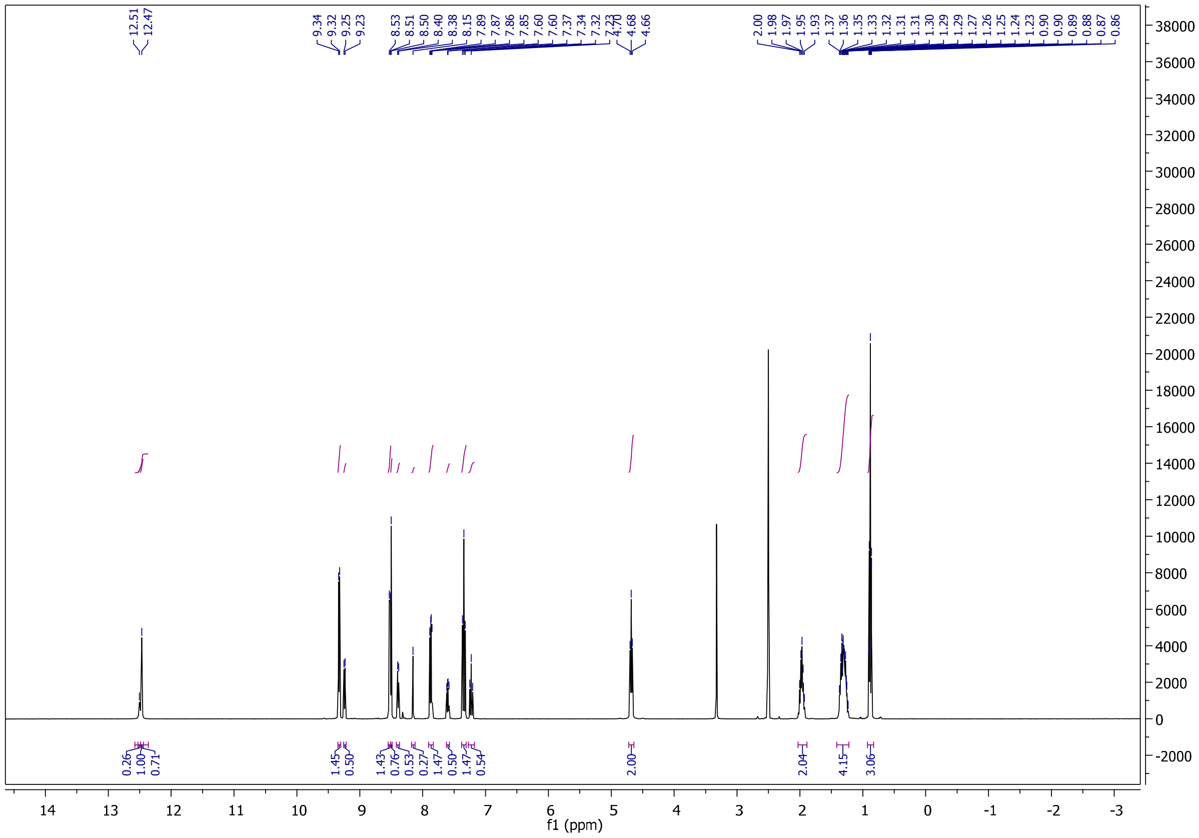

Figure S104. ^{1}H NMR of Compound **30**.

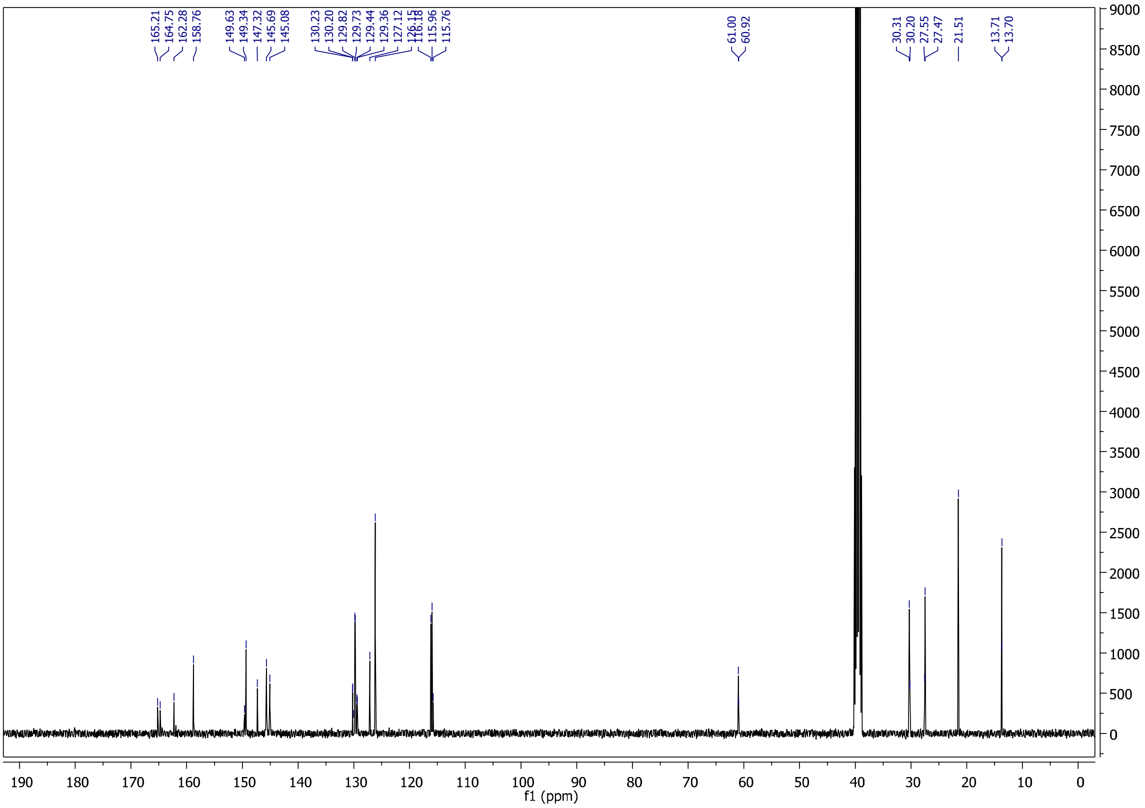

Figure S105. ^{13}C NMR of Compound **30**.

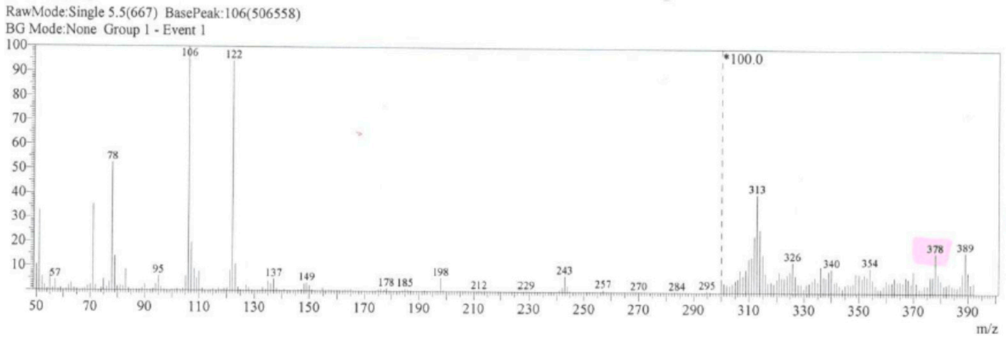

Figure S106. MS (ESI) of Compound **30**.

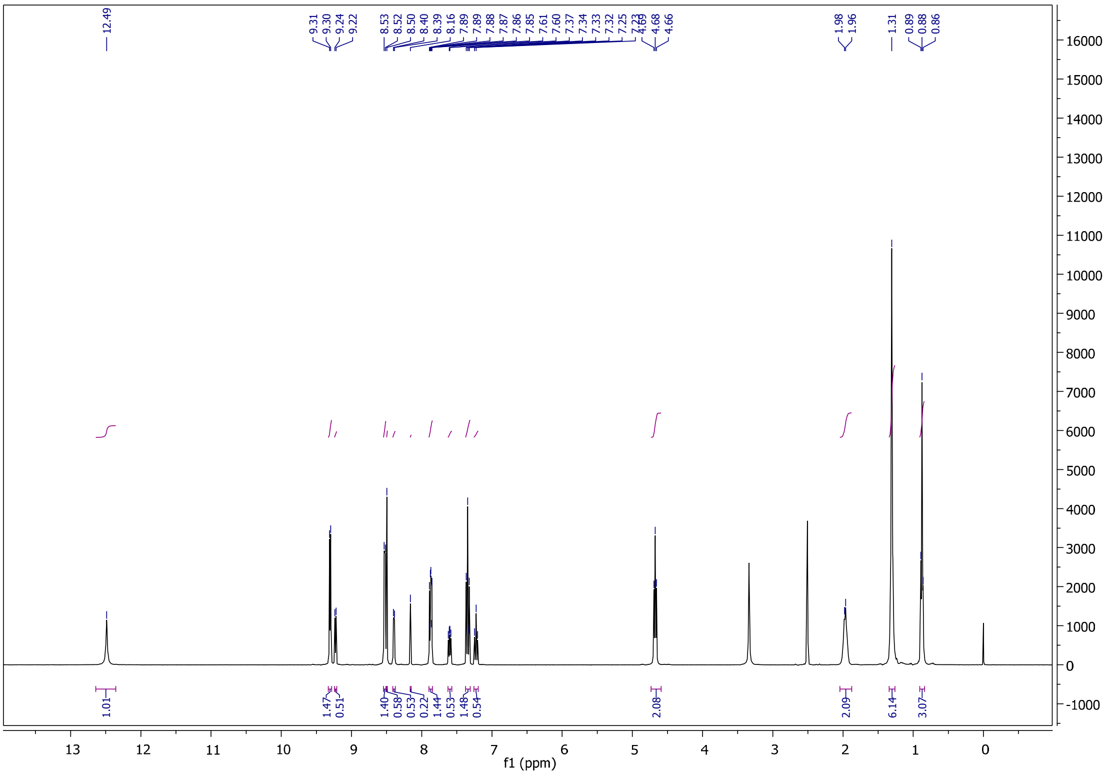

Figure S107. ^{1}H NMR of Compound **31**.

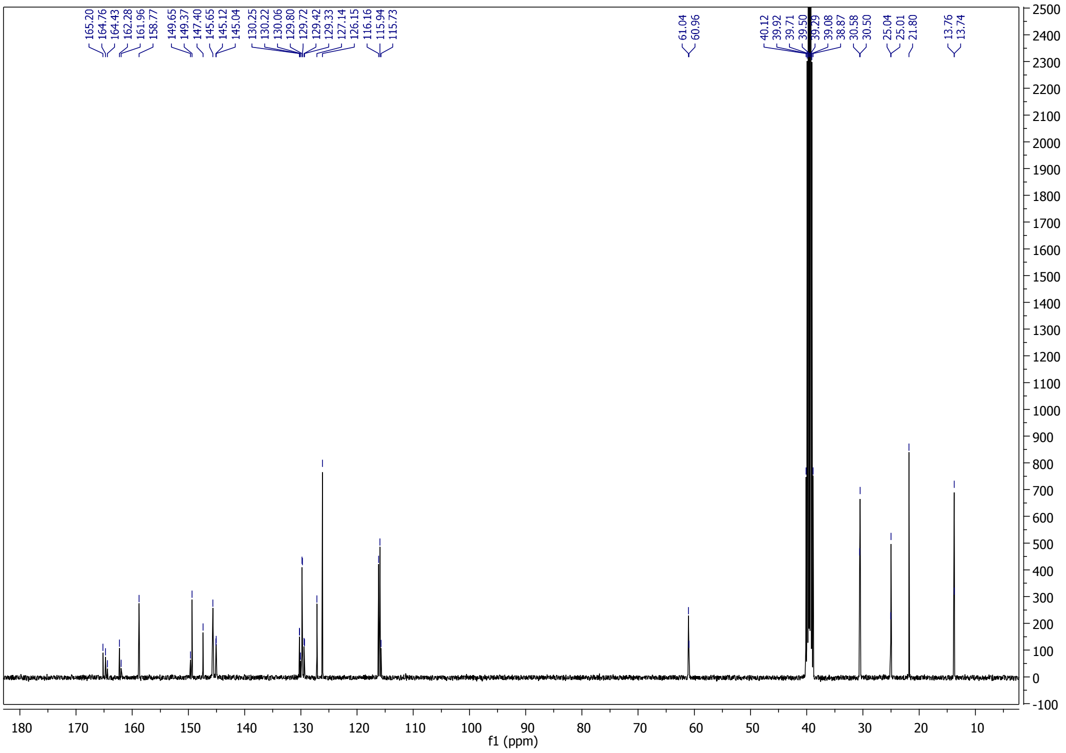

Figure S108. ^{13}C NMR of Compound **31**.

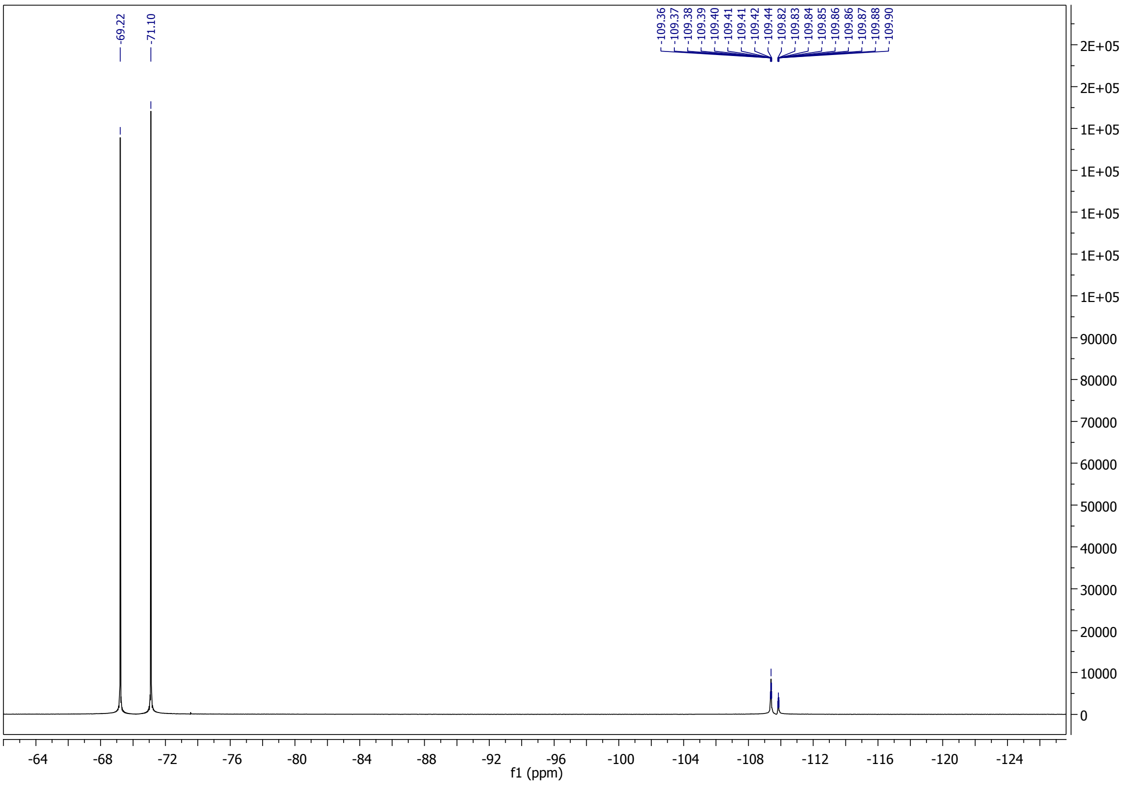

Figure S109. ^{19}F NMR of Compound **31**.

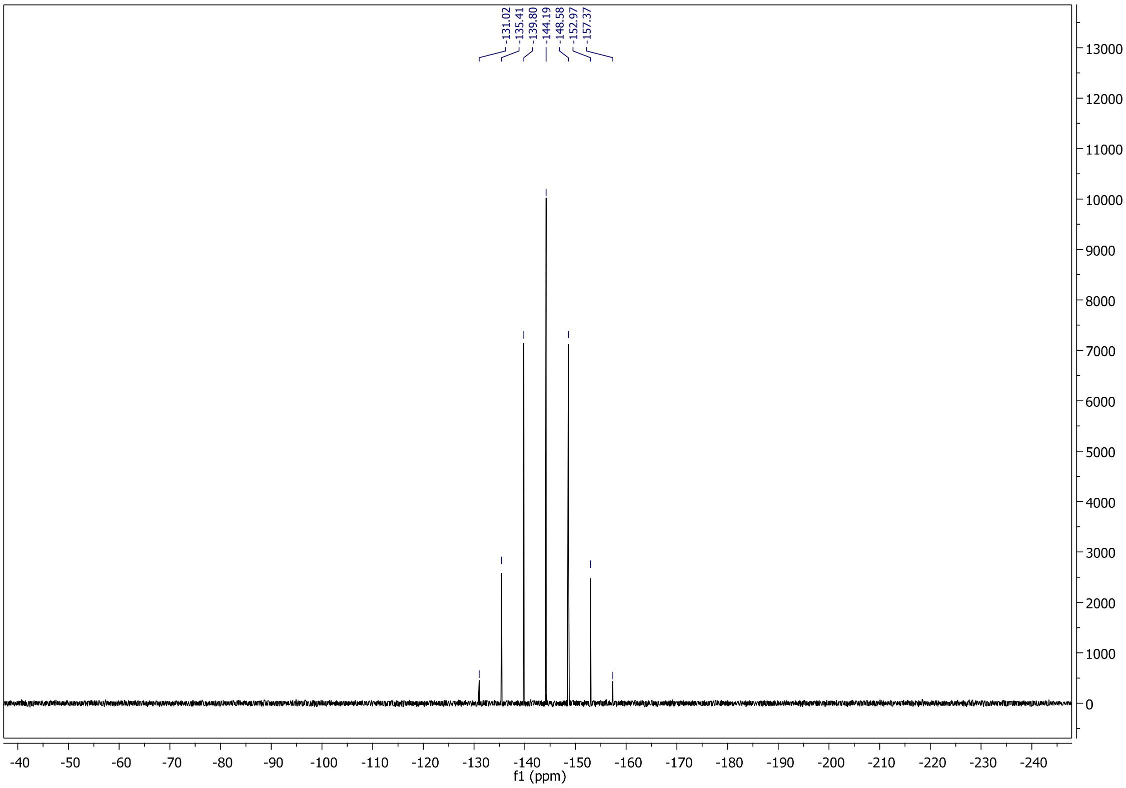

Figure S110. ^{31}P NMR of Compound **31**.

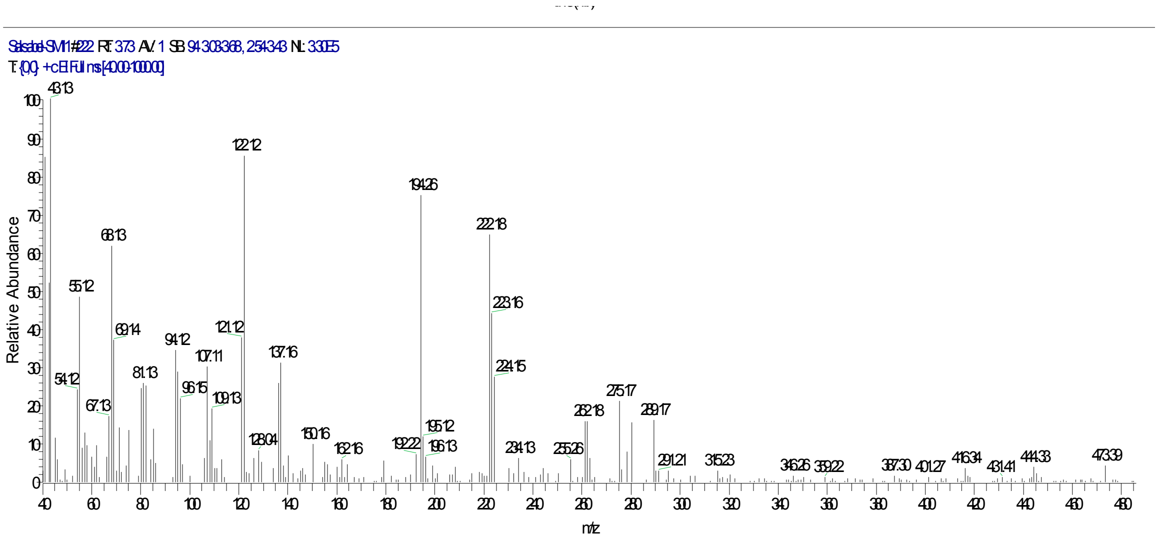

Figure S111. MS (ESI) of Compound **31**.

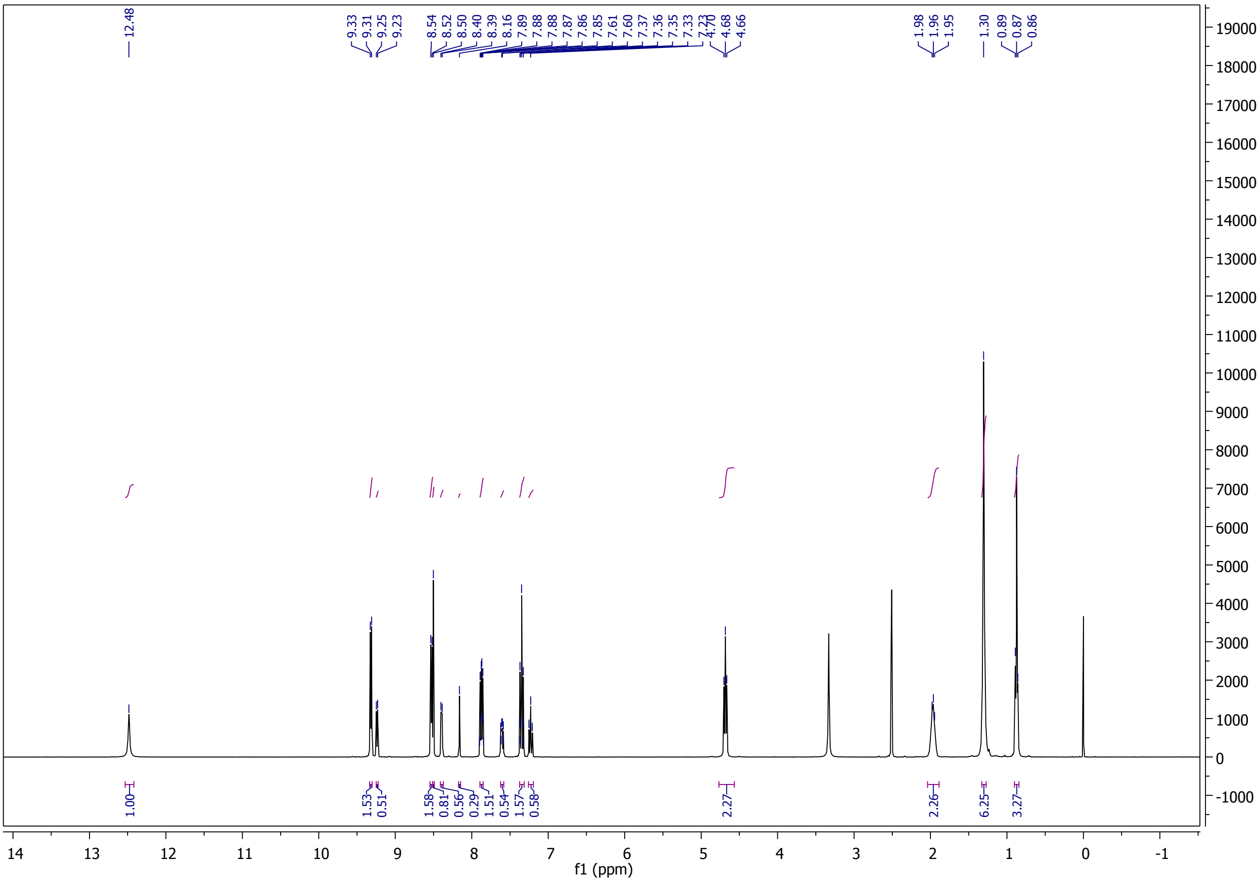

Figure S112. ^{1}H NMR of Compound **32**.

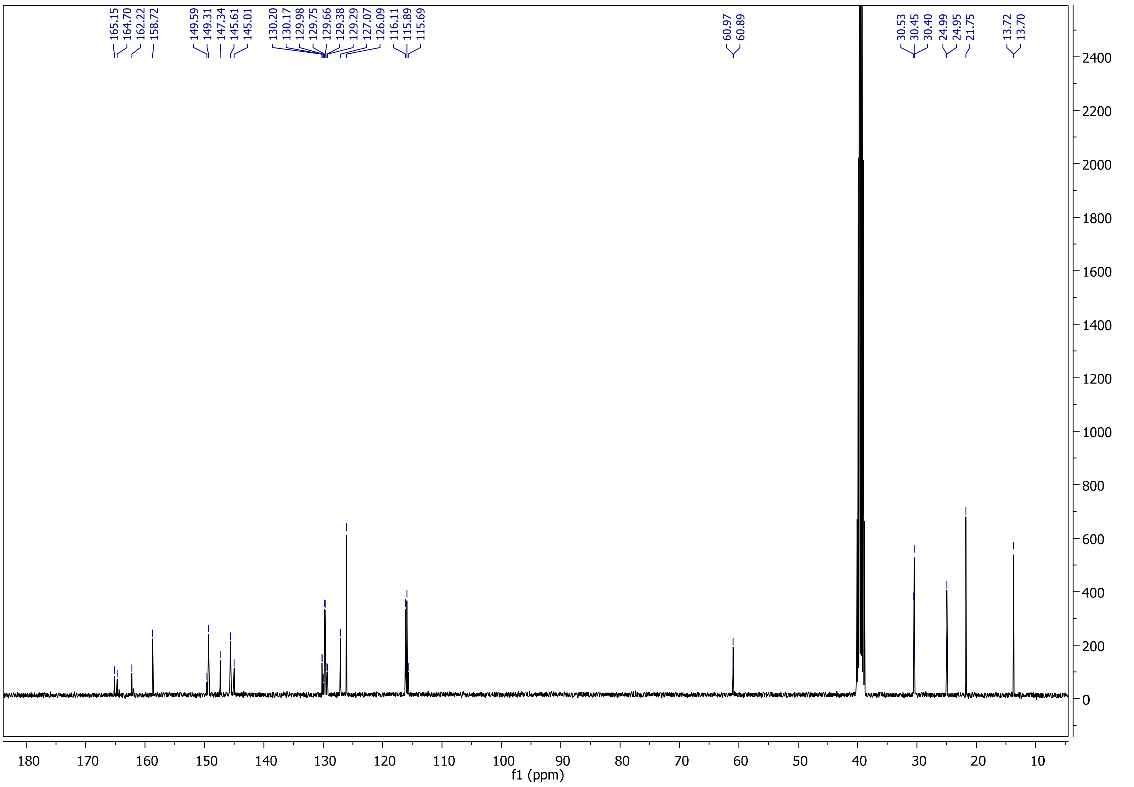

Figure S113. ^{13}C NMR of Compound **32**.

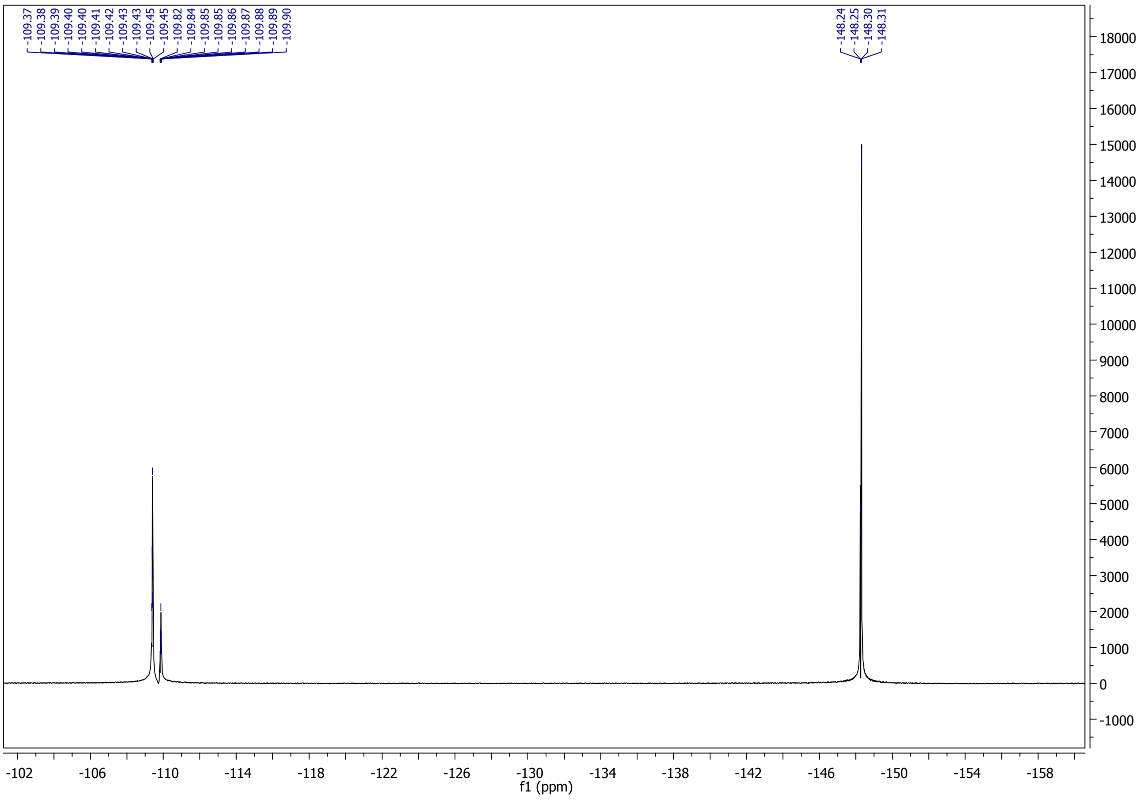

Figure S114. ^{19}F NMR of Compound **32**.

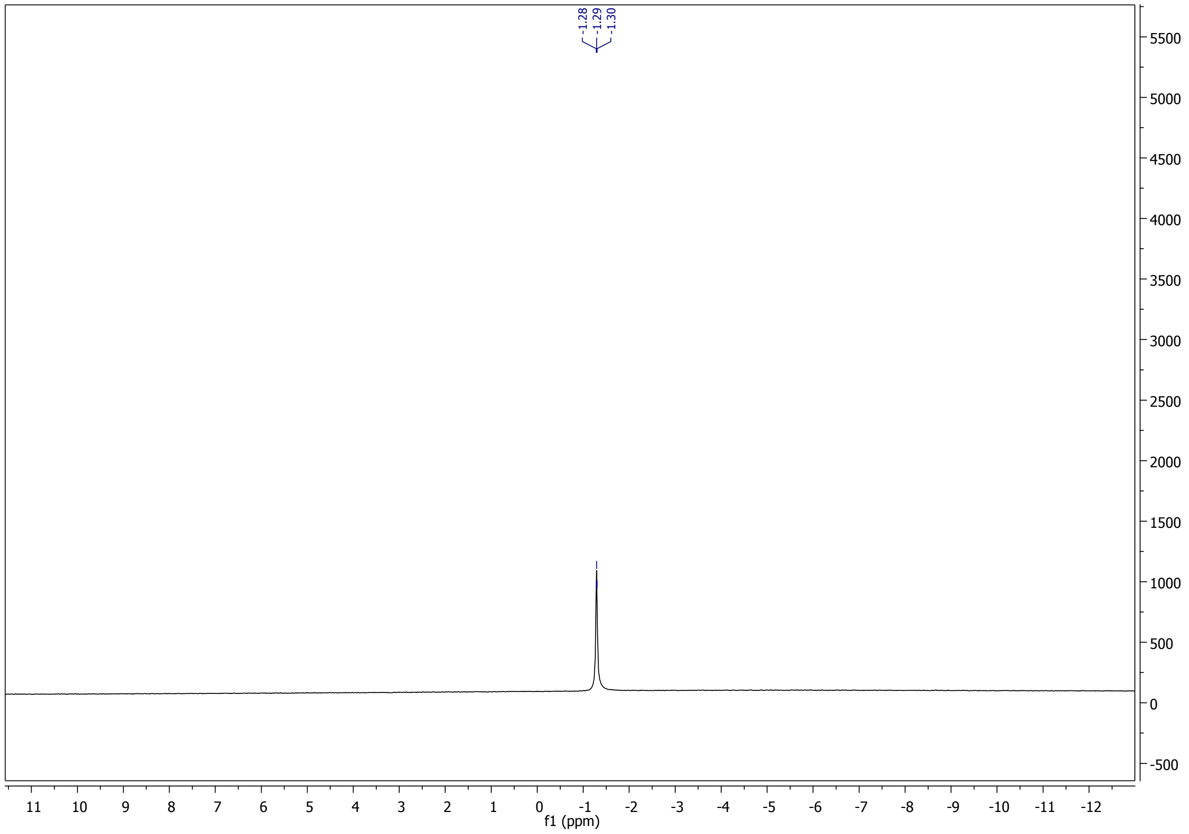

Figure S115. ^{11}B NMR of Compound **32**.

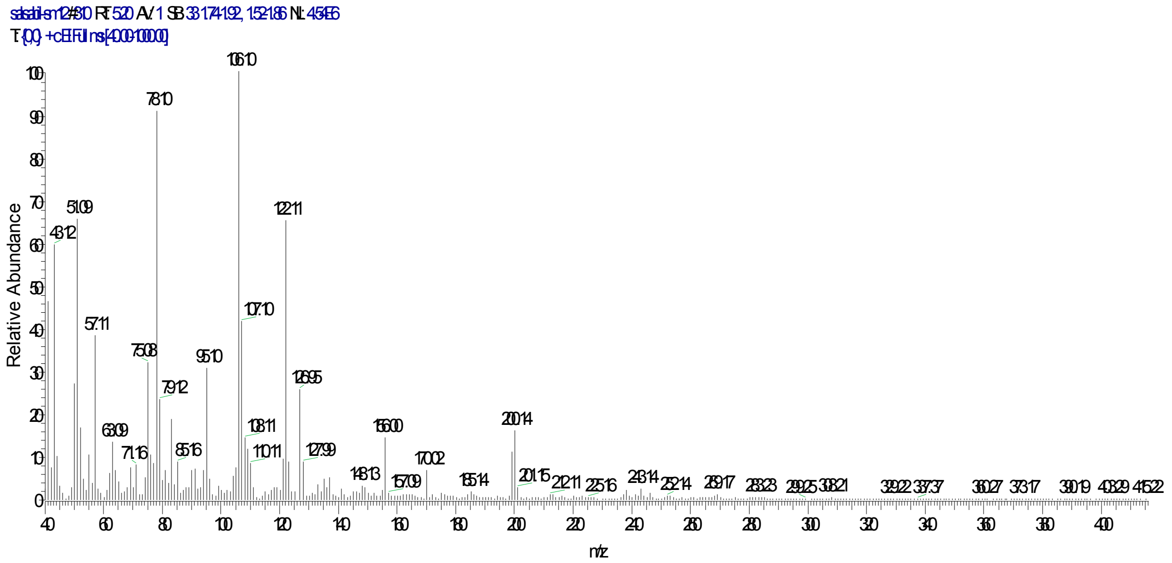

Figure S116. MS (ESI) of Compound **32**.

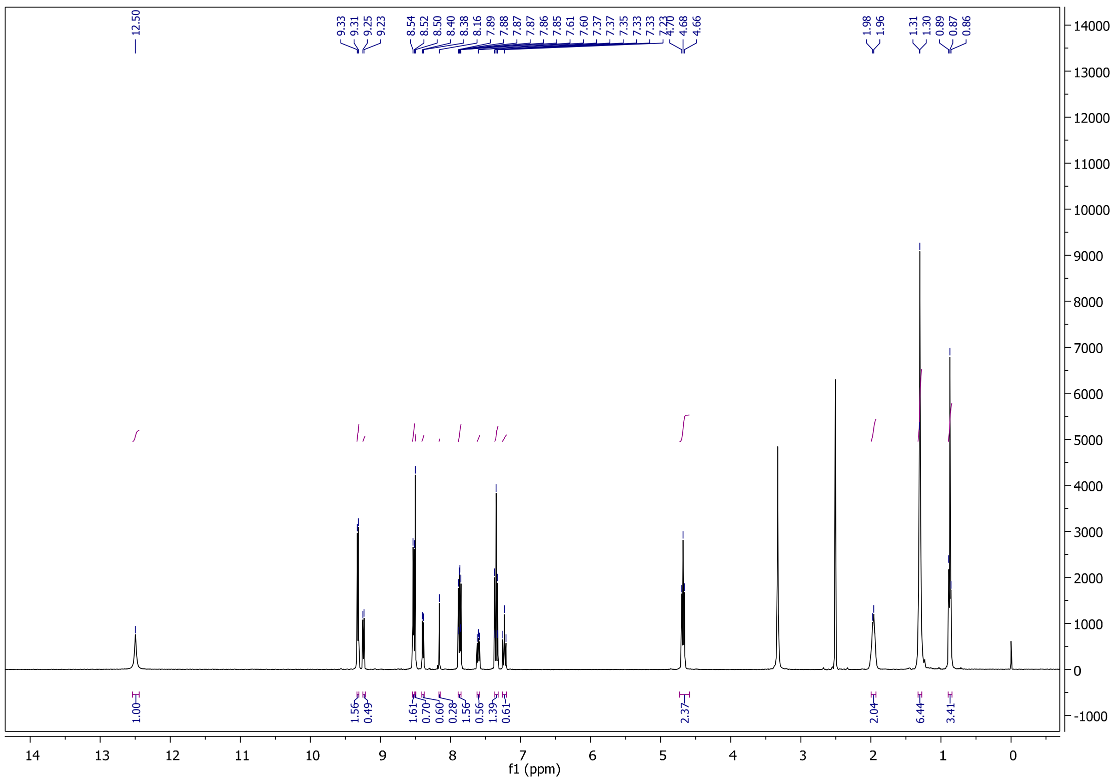

Figure S117. ^{1}H NMR of Compound **33**.

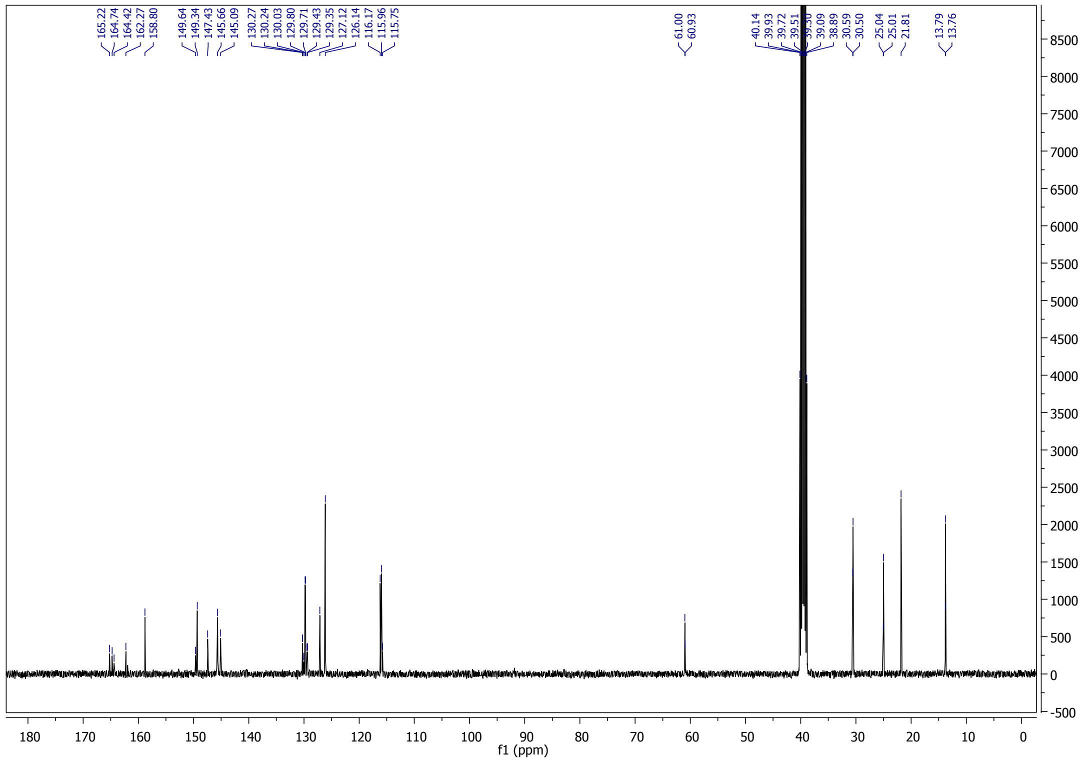

Figure S118. ^{13}C NMR of Compound **33**.

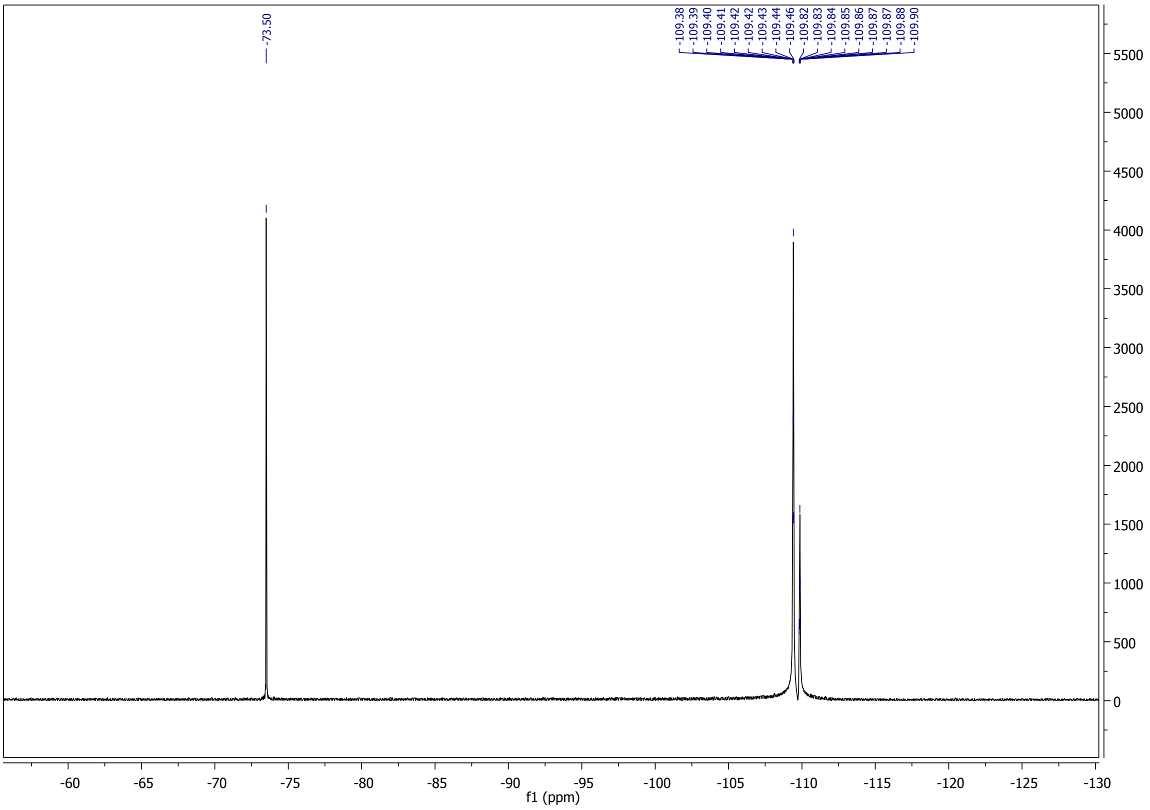

Figure S119. ^{19}F NMR of Compound **33**.

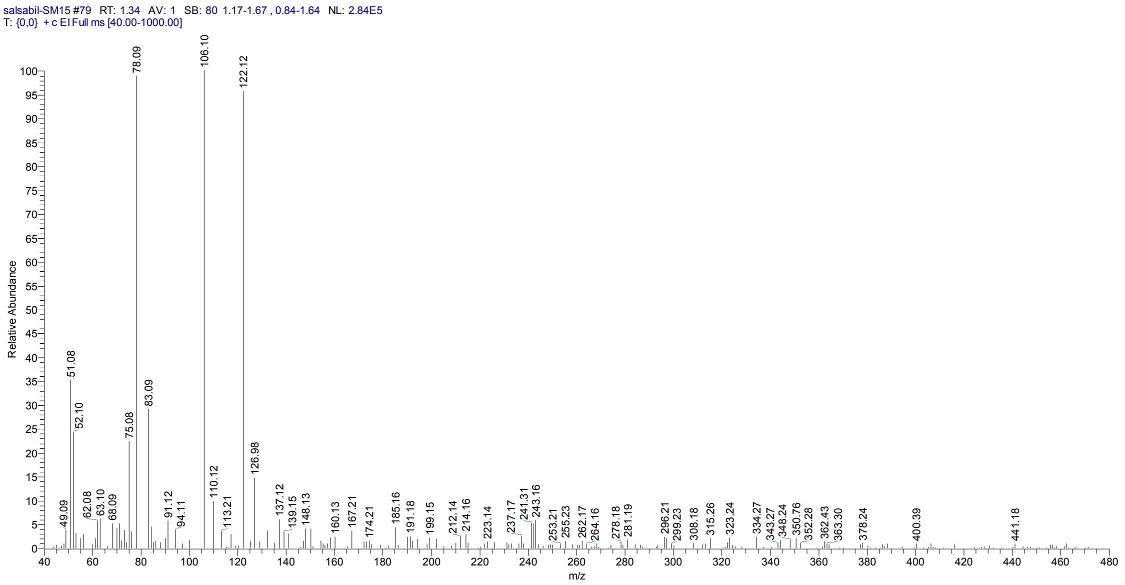

Figure S120. MS (ESI) of Compound **33**.

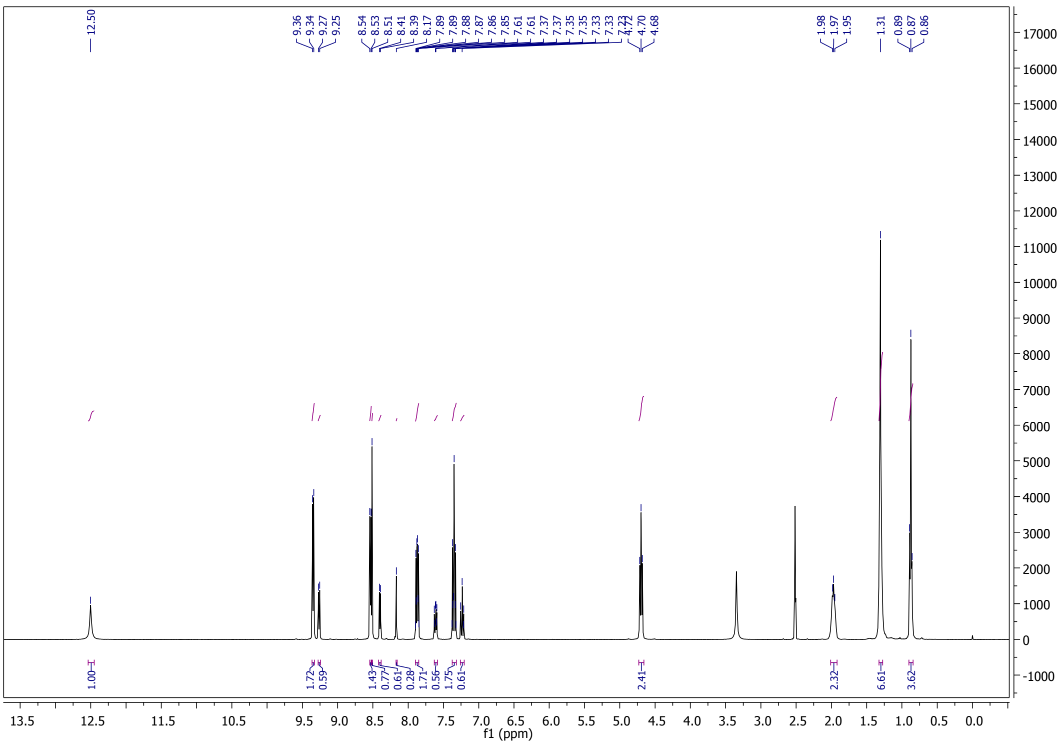

Figure S121. ^{1}H NMR of Compound **34**.

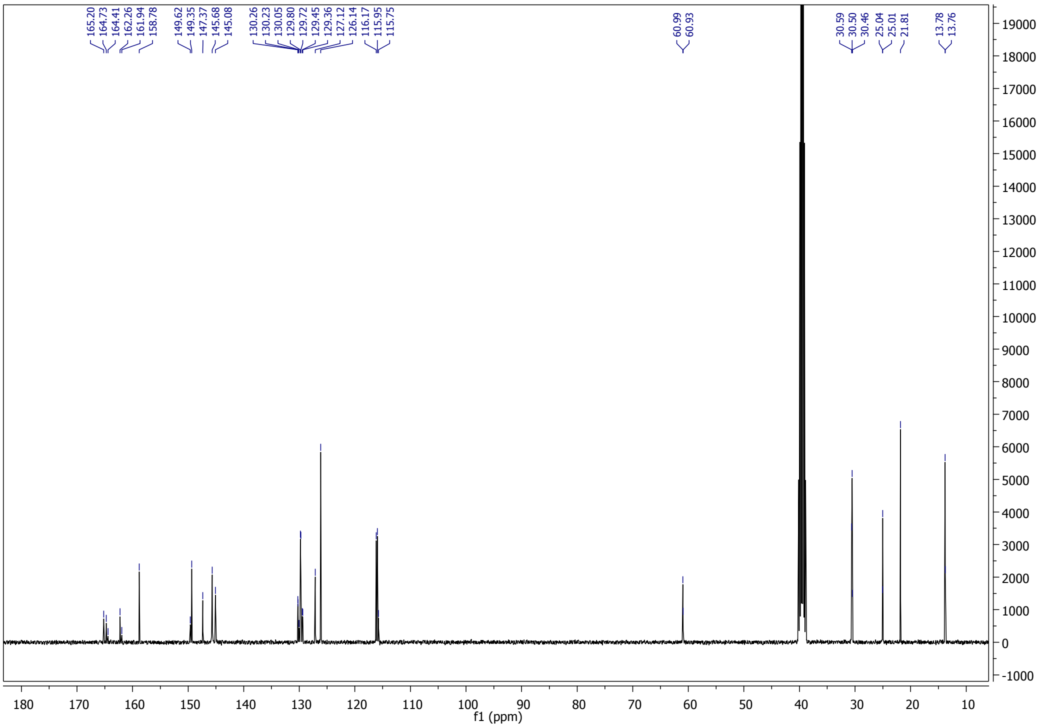

Figure S122. ^{13}C NMR of Compound **34**.

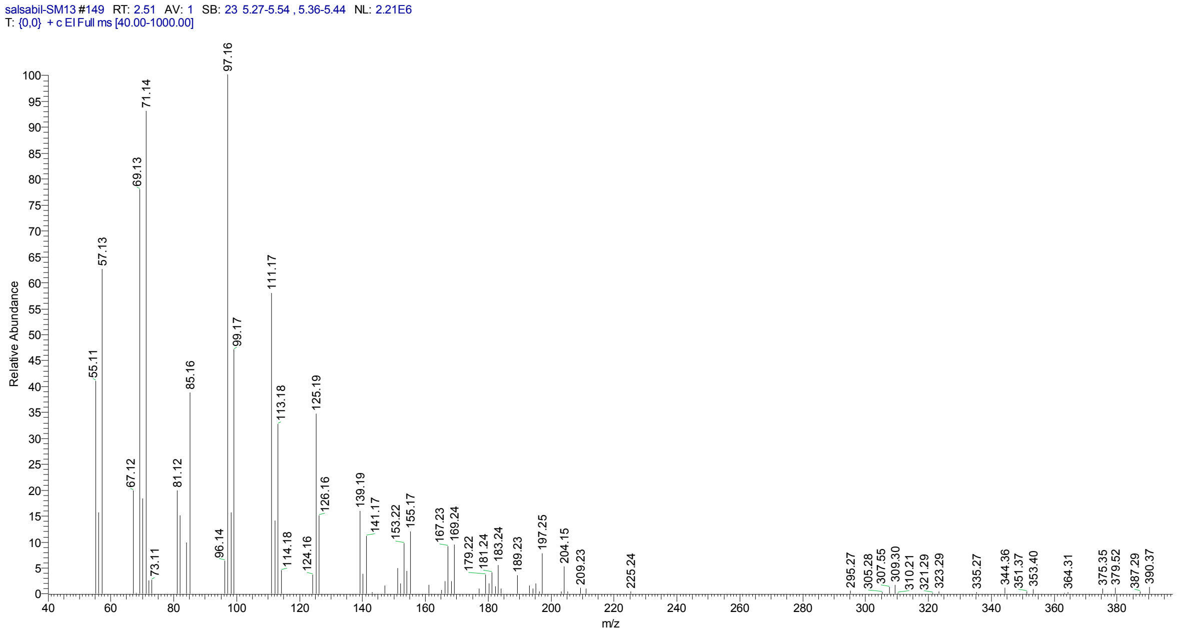

Figure S123. MS (ESI) of Compound **34**.

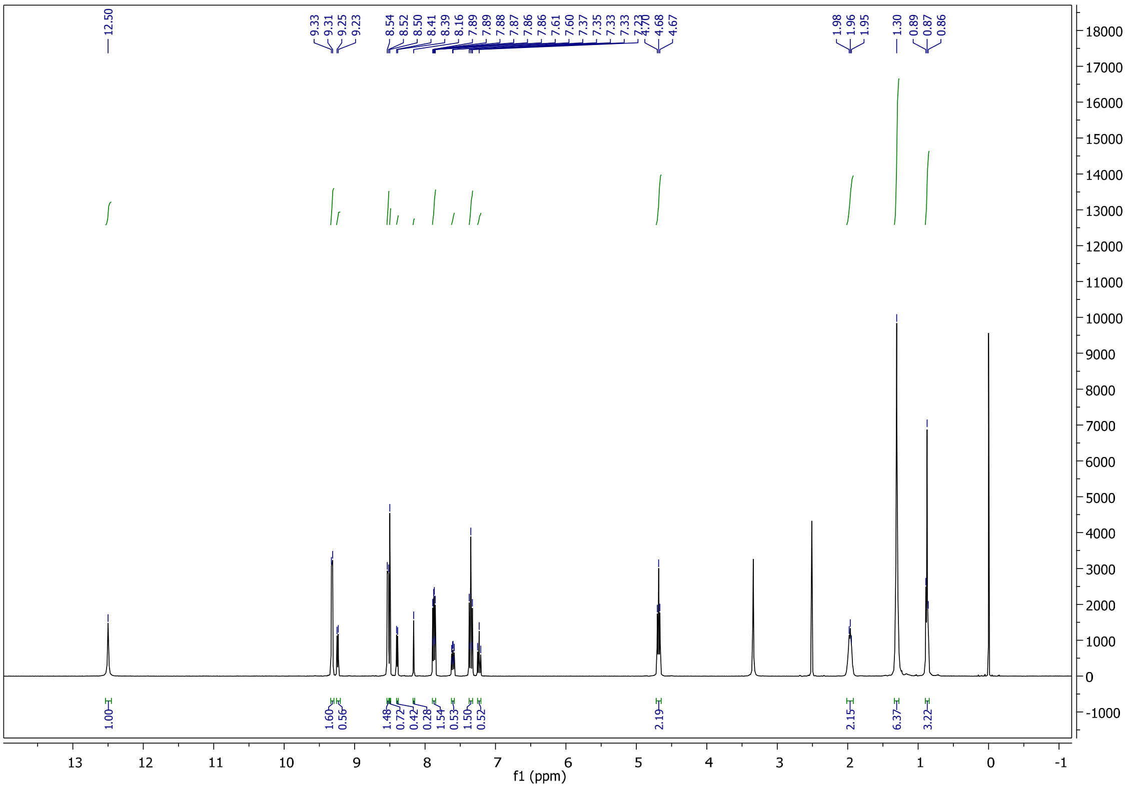

Figure S124. ^{1}H NMR of Compound **35**.

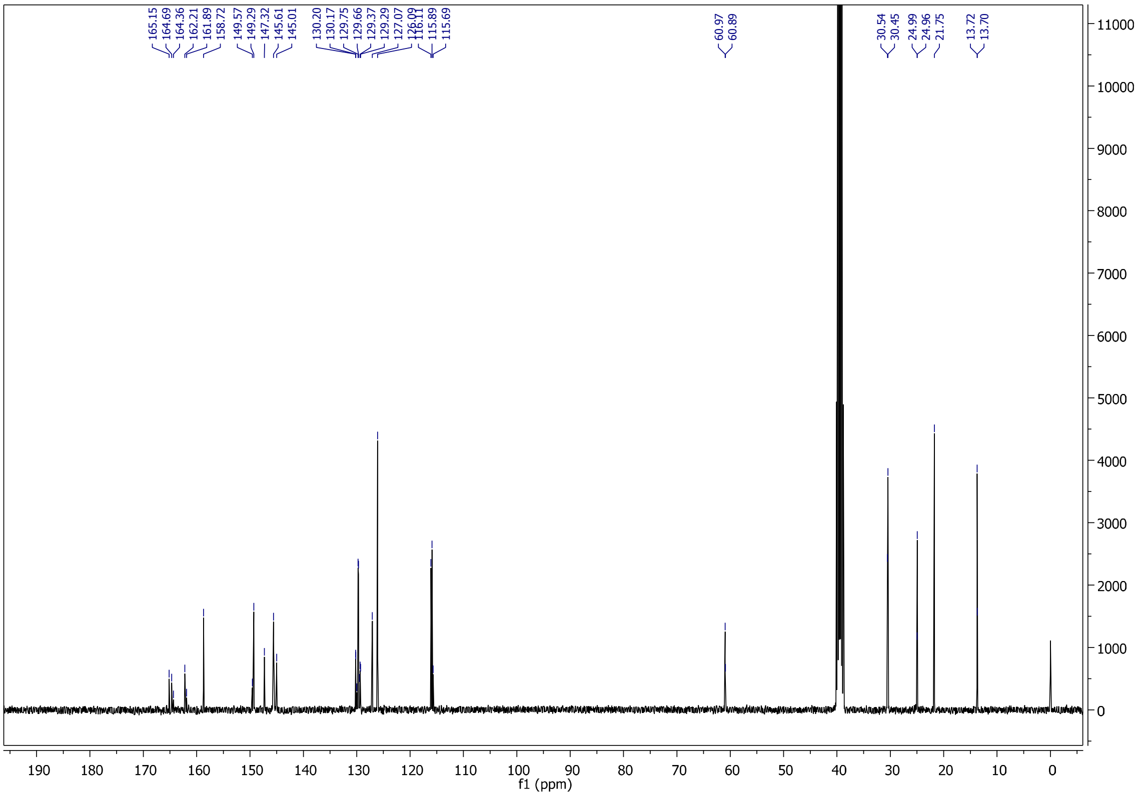

Figure S125. ^{13}C NMR of Compound **35**.

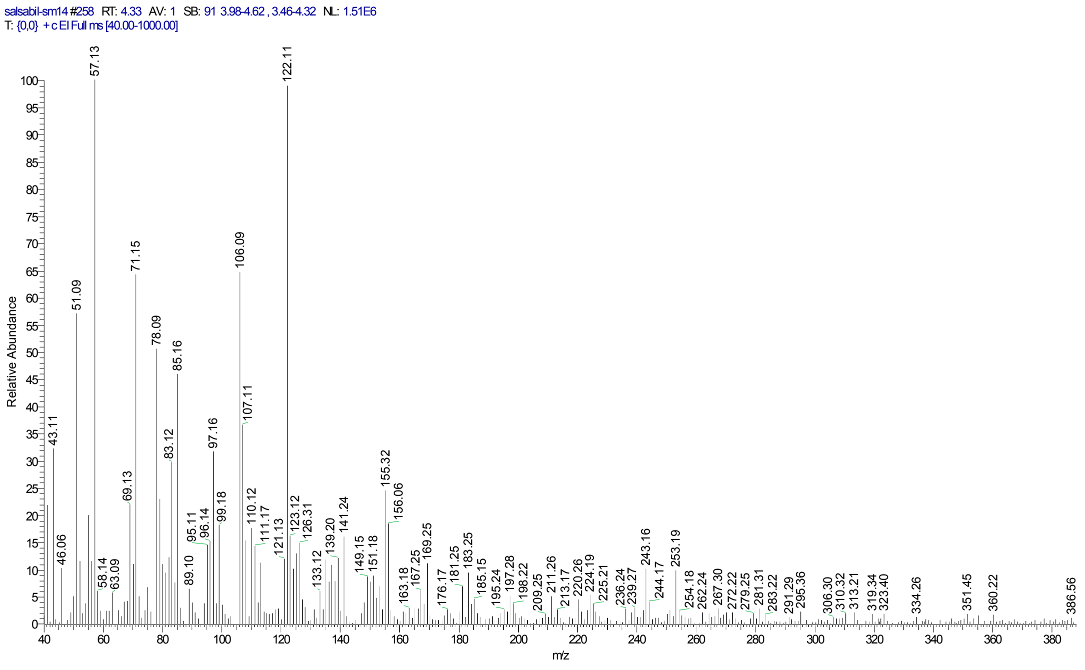

Figure S126. MS (ESI) of Compound **35**.

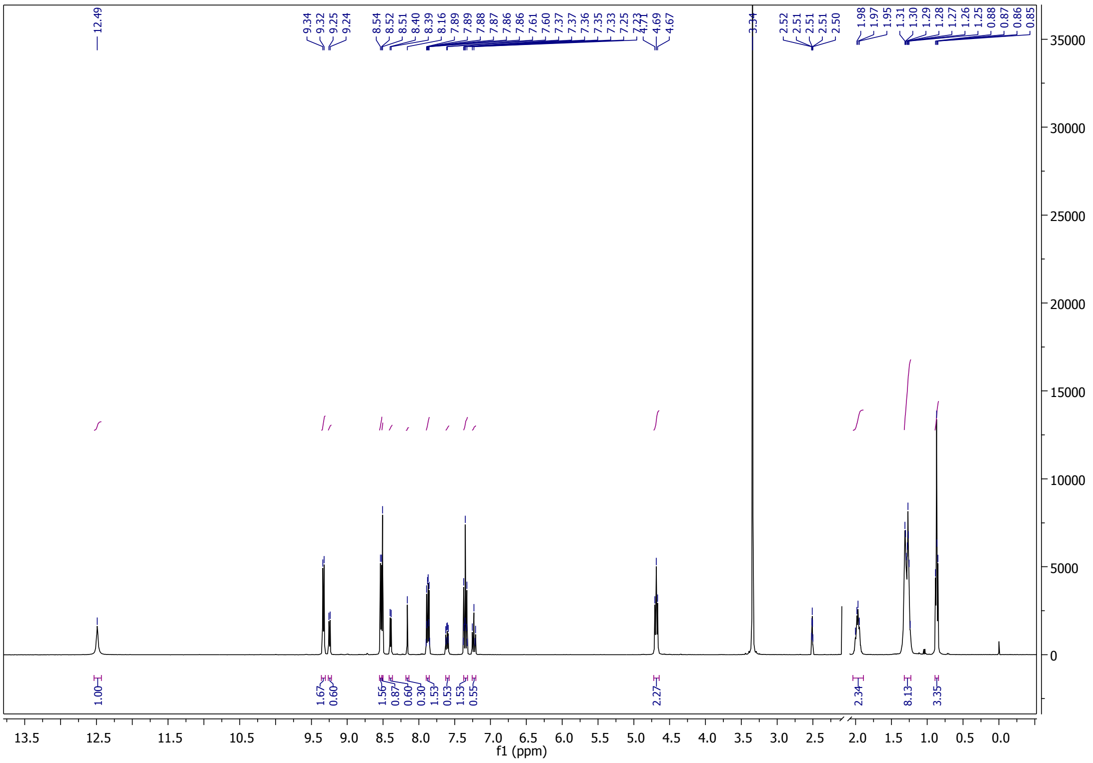

Figure S127. ^{1}H NMR of Compound **36**.

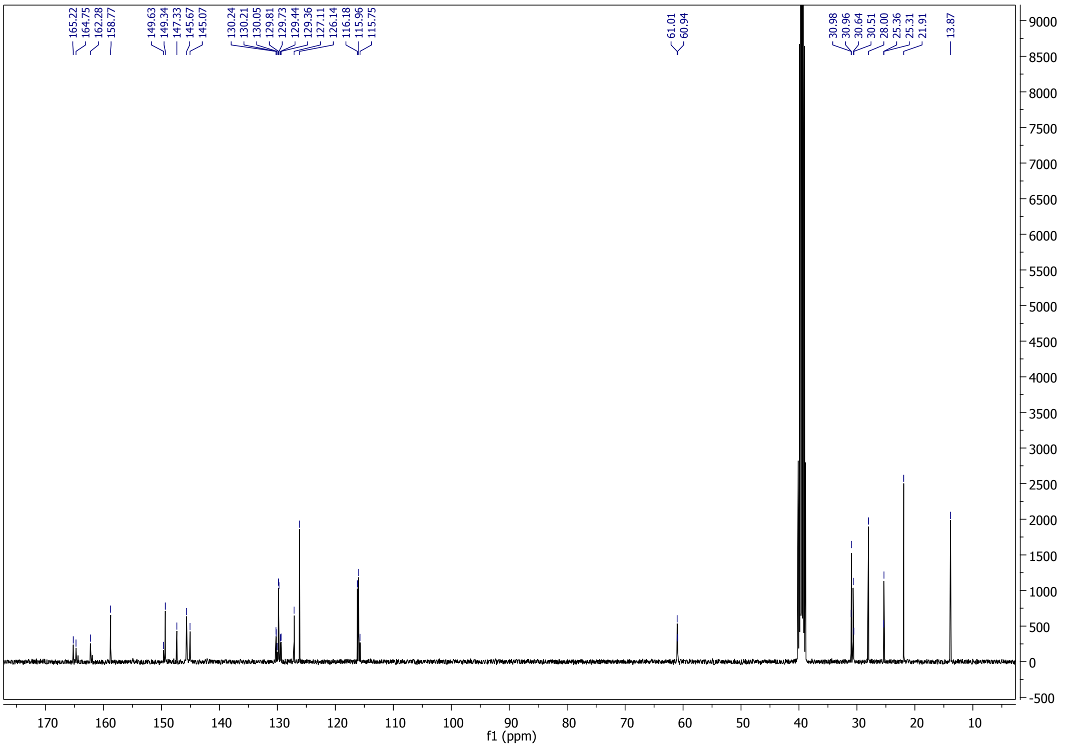

Figure S128. ^{13}C NMR of Compound **36**.

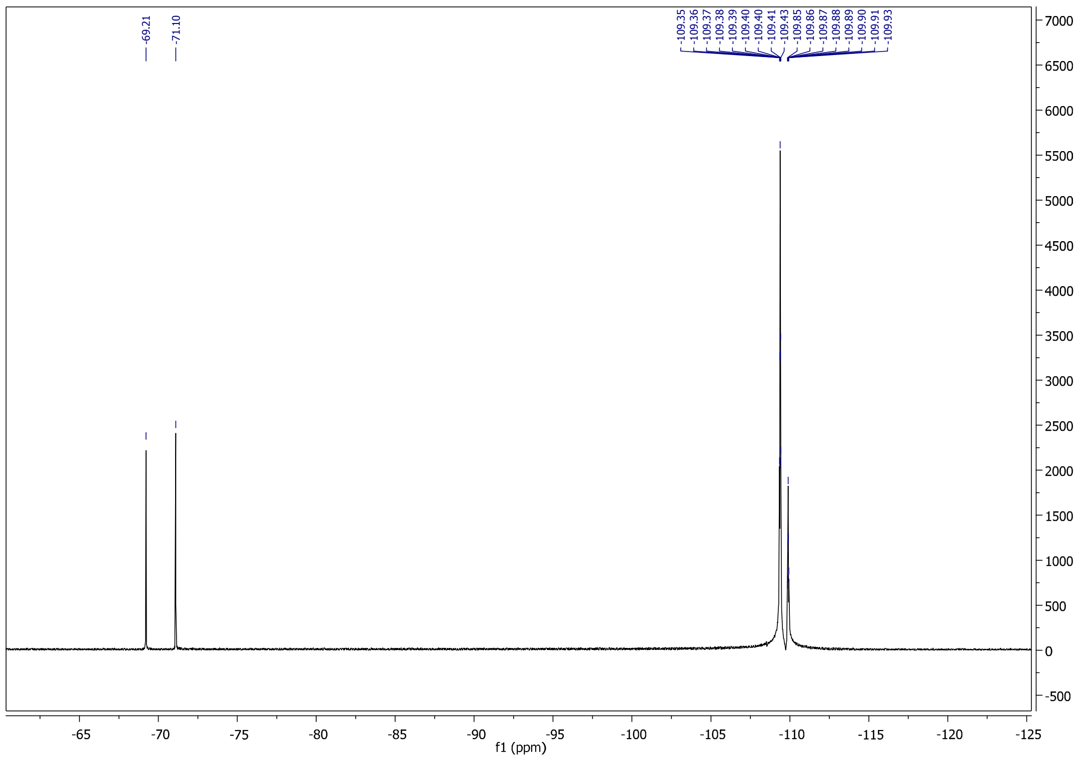

Figure S129. ^{19}F NMR of Compound **36**.

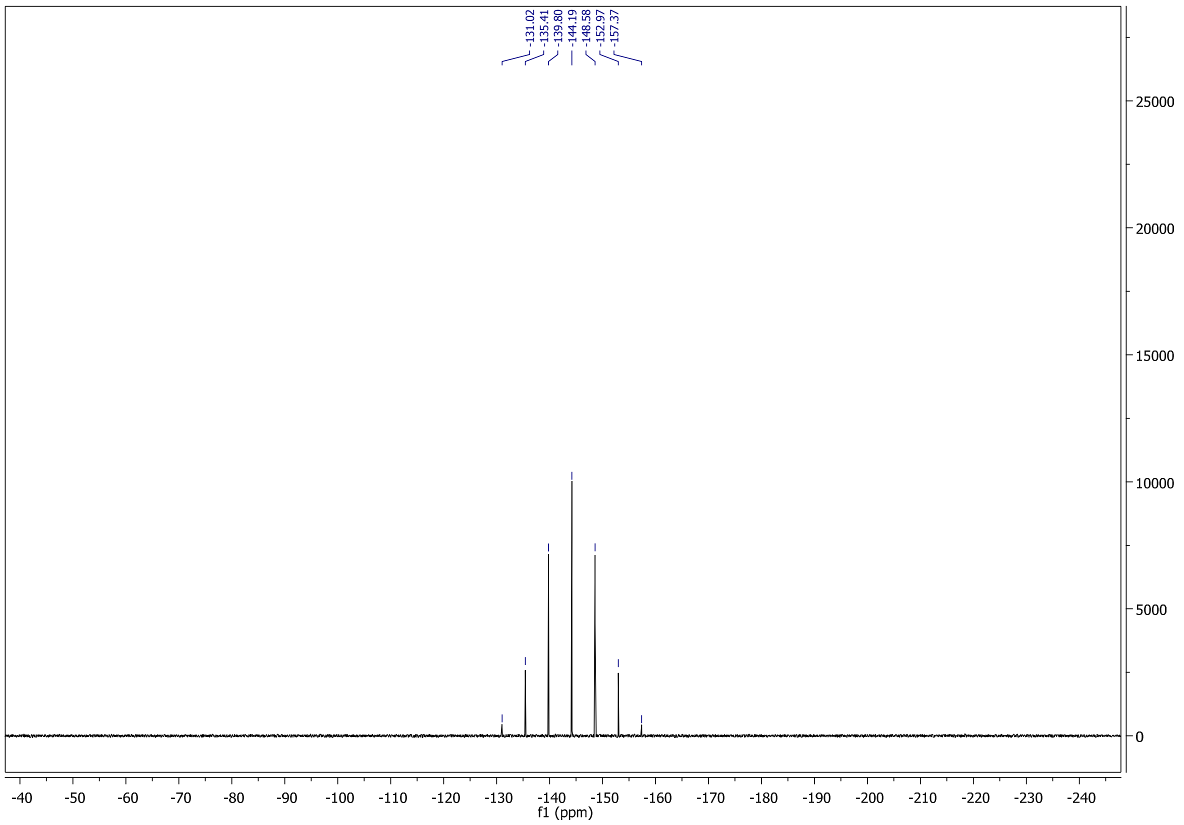

Figure S130. ^{31}P NMR of Compound **36**.

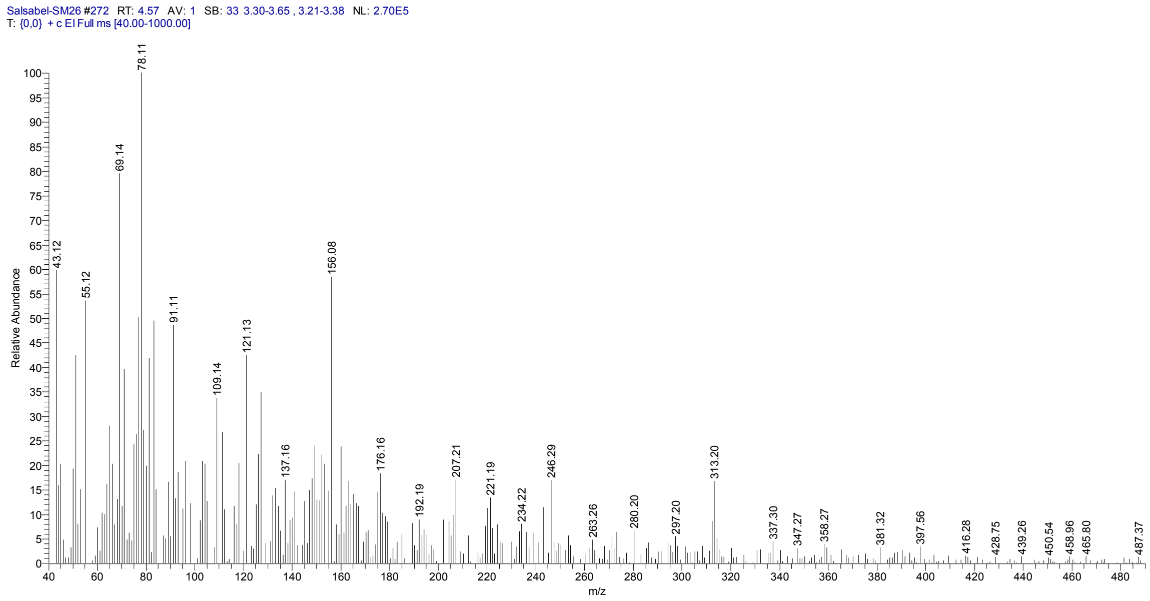

Figure S131. MS (ESI) of Compound **36**.

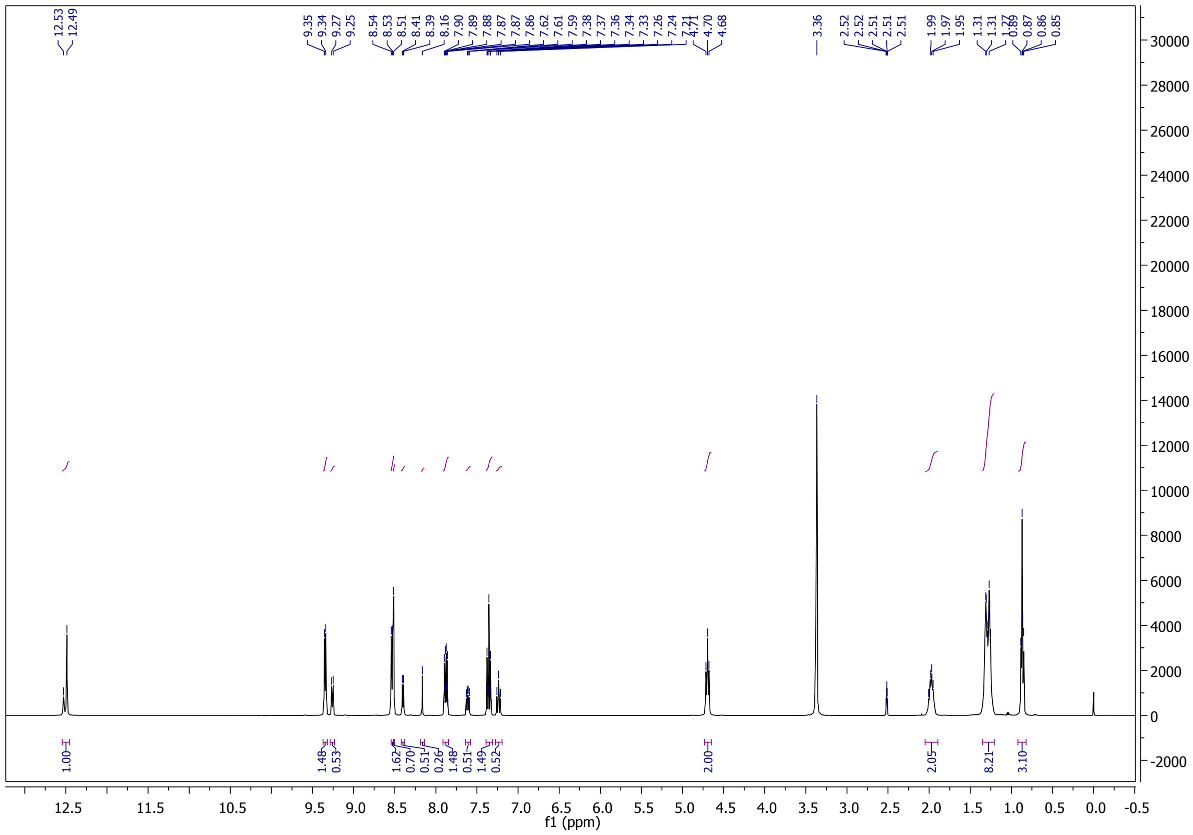

Figure S132. ^{1}H NMR of Compound **37**.

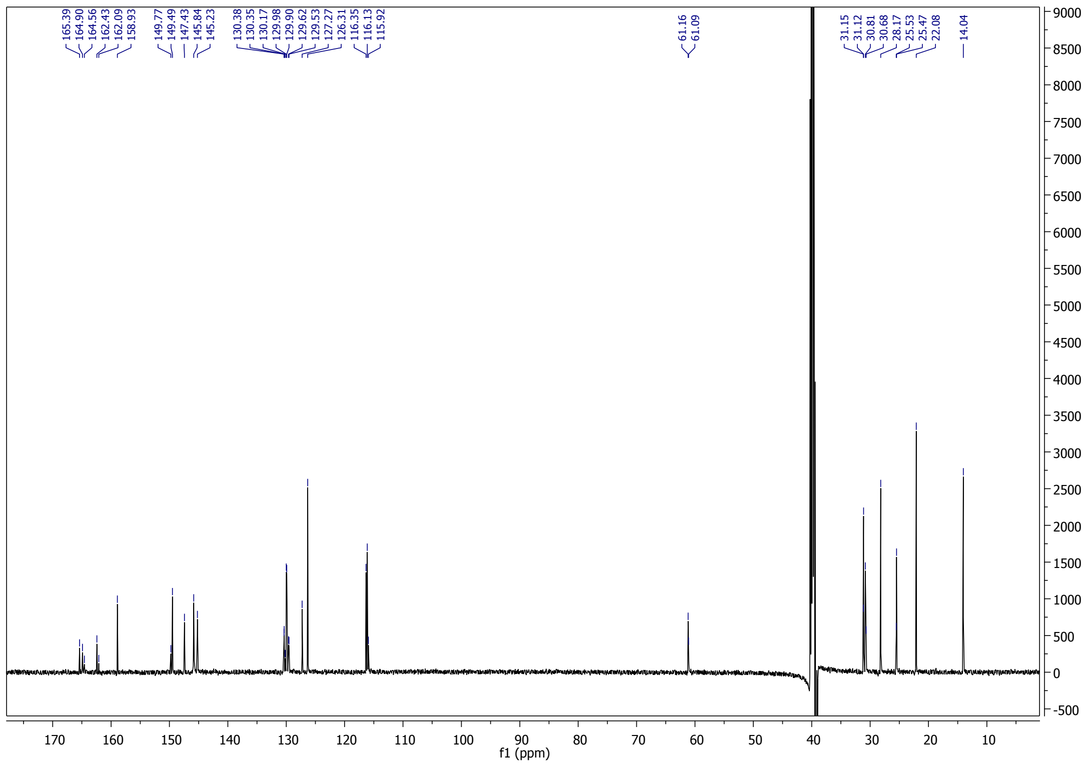

Figure S133. ^{13}C NMR of Compound **37**.

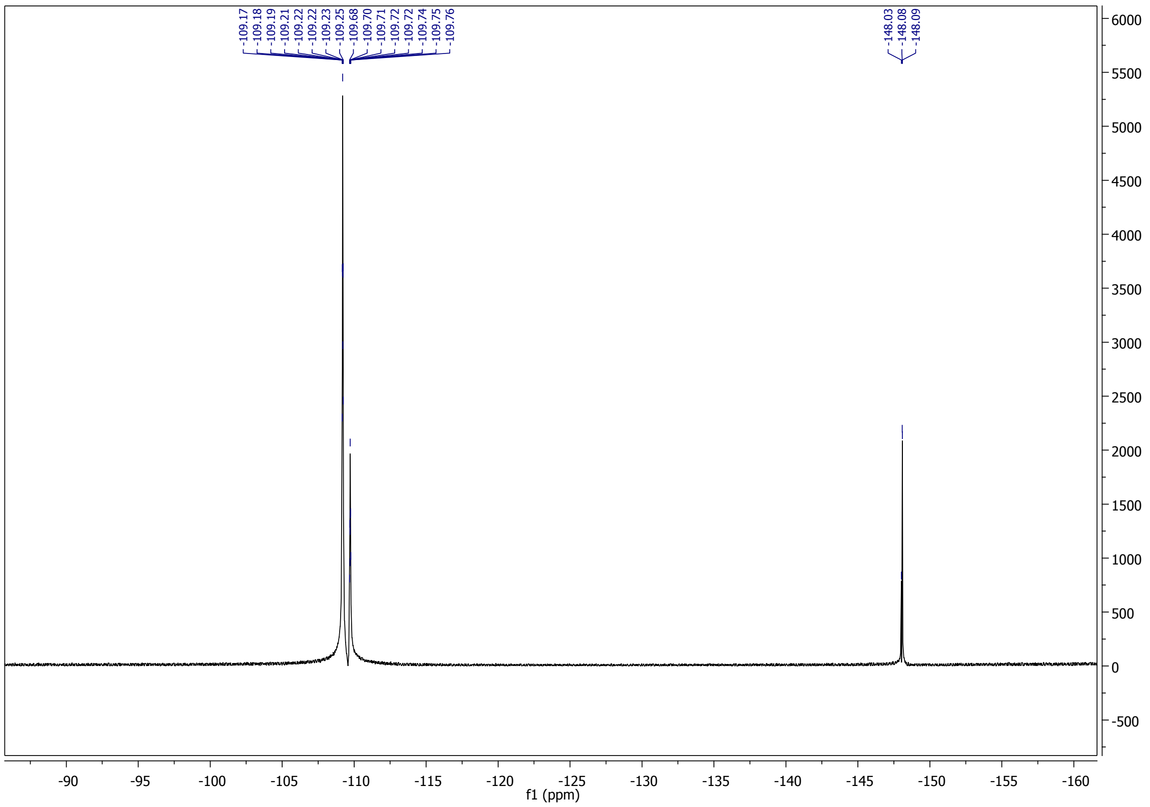

Figure S134. ^{19}F NMR of Compound **37**.

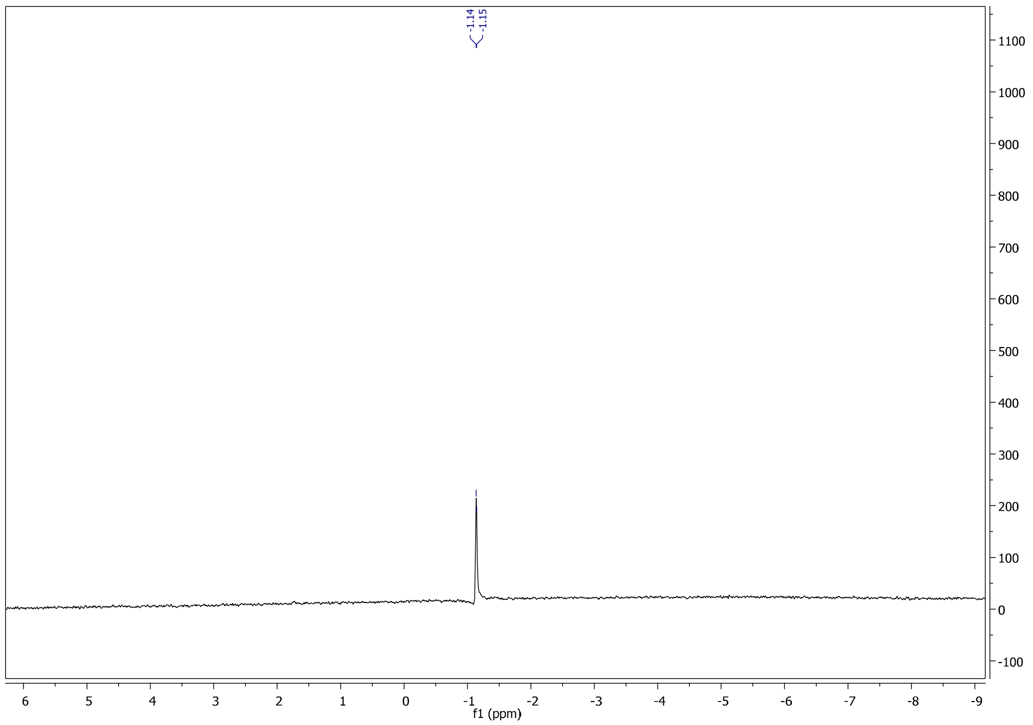

Figure S135. ^{11}B NMR of Compound **37**.

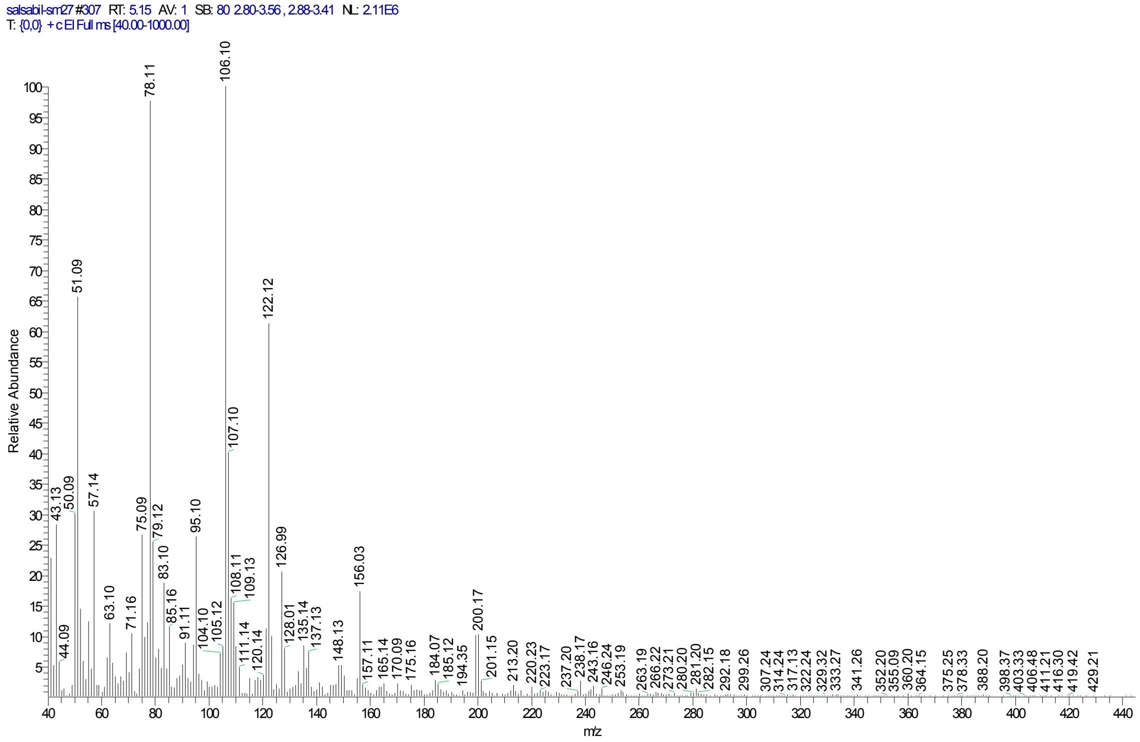

Figure S136. MS (ESI) of Compound **37**.

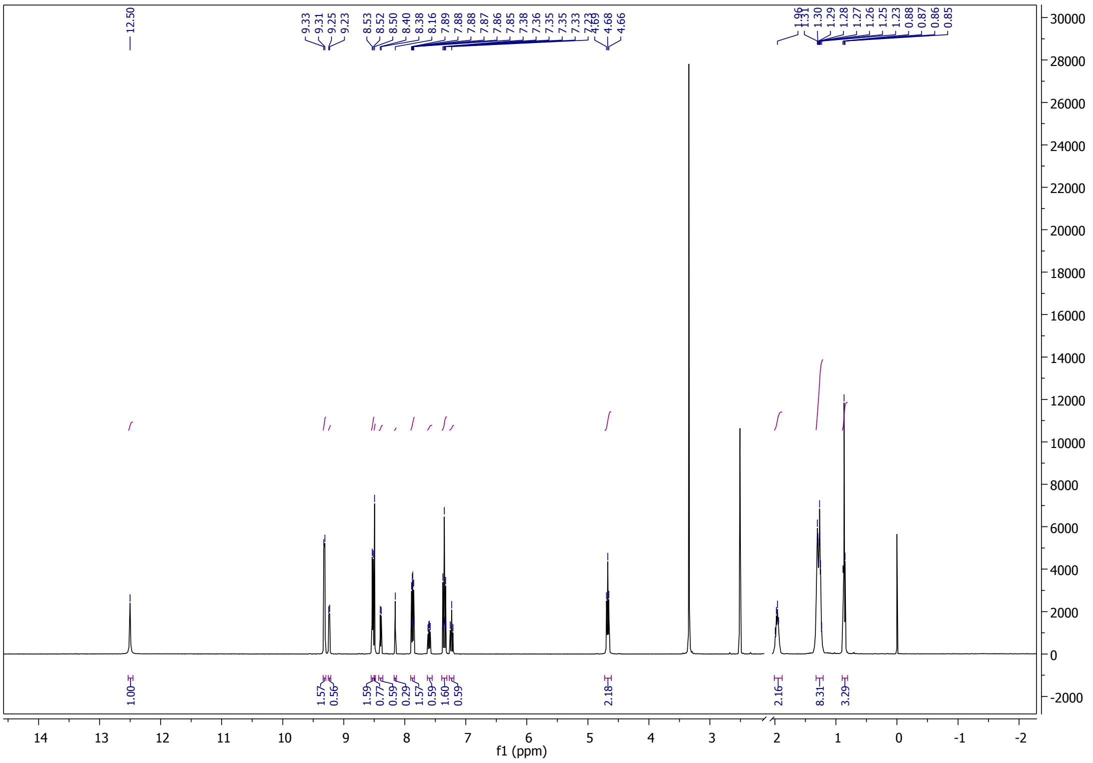

Figure S137. ^{1}H NMR of Compound **38**.

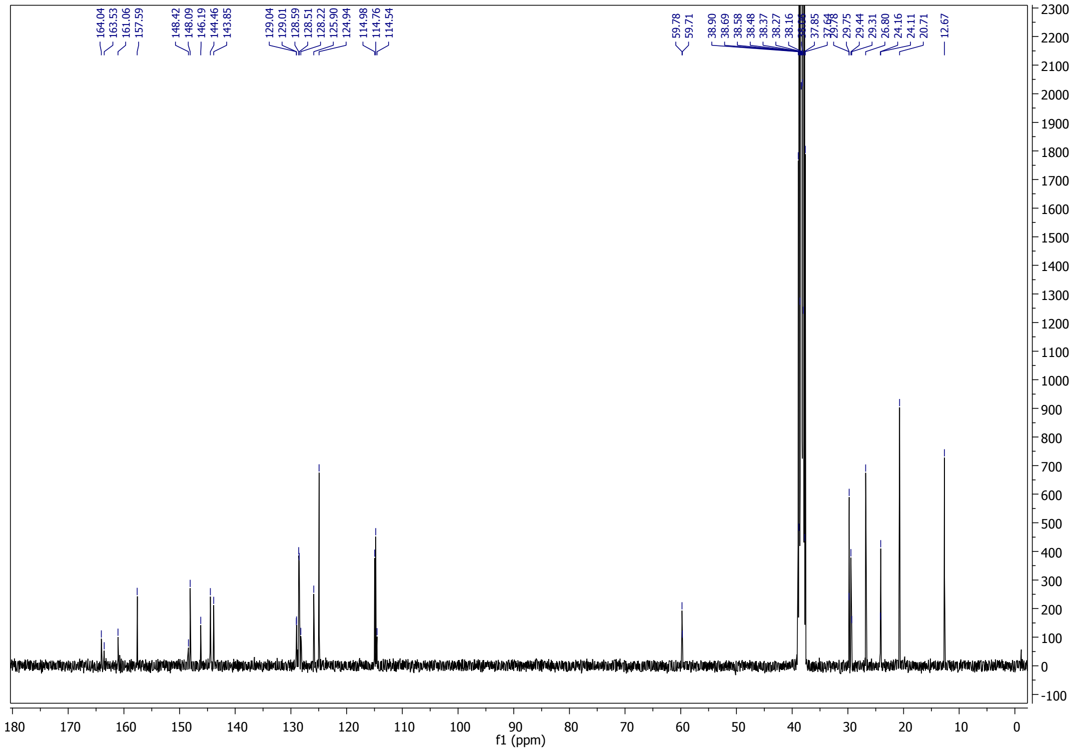

Figure S138. ^{13}C NMR of Compound **38**.

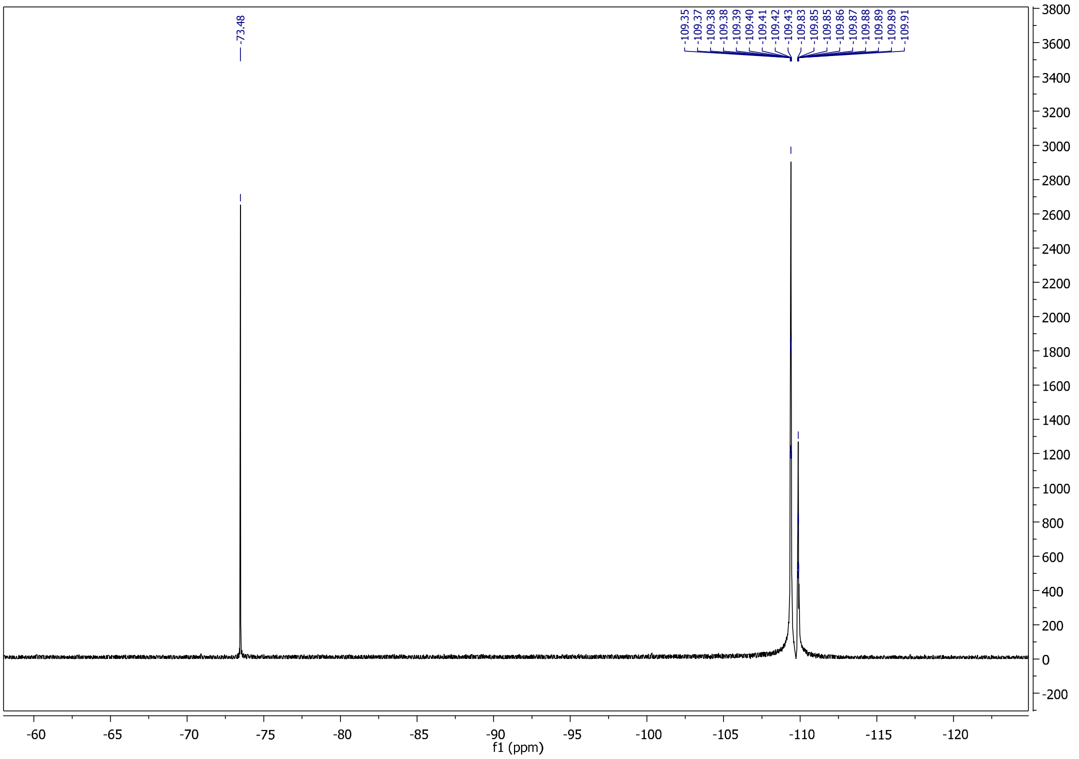

Figure S139. ^{19}F NMR of Compound **38**.

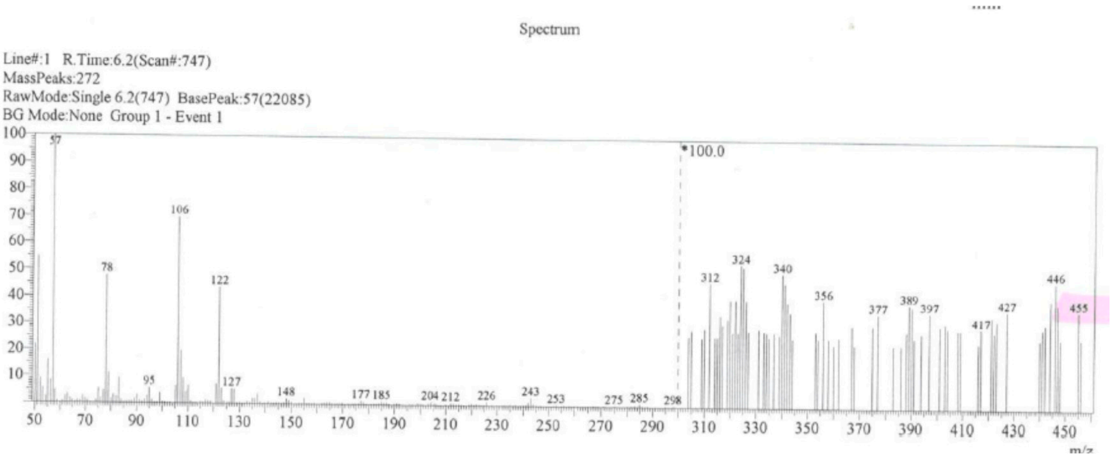

Figure S140. MS (ESI) of Compound **38**.

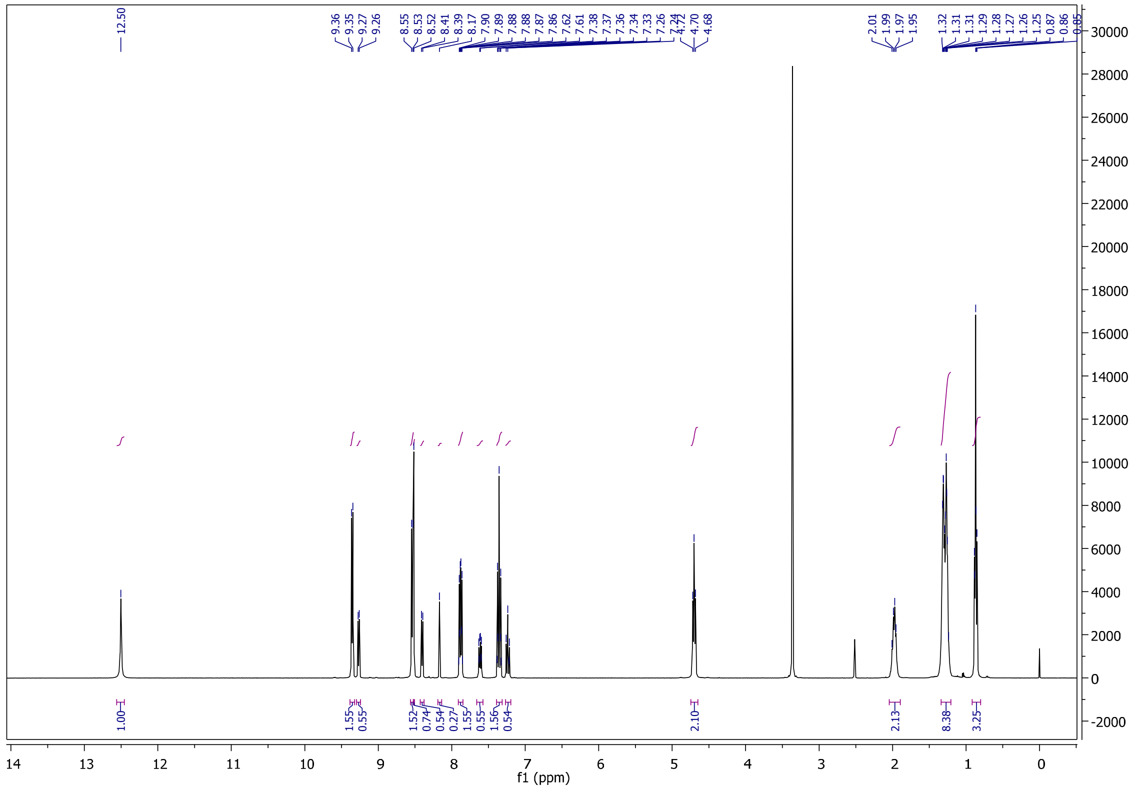

Figure S141. ^{1}H NMR of Compound **39**.

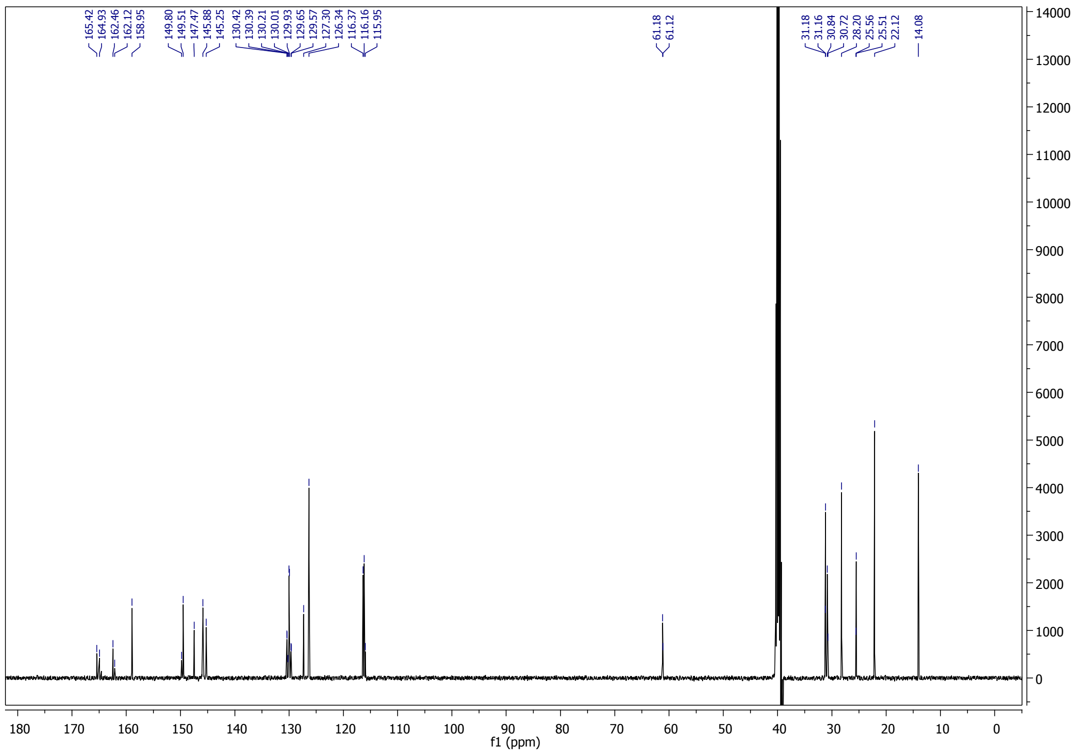

Figure S142. ^{13}C NMR of Compound **39**.

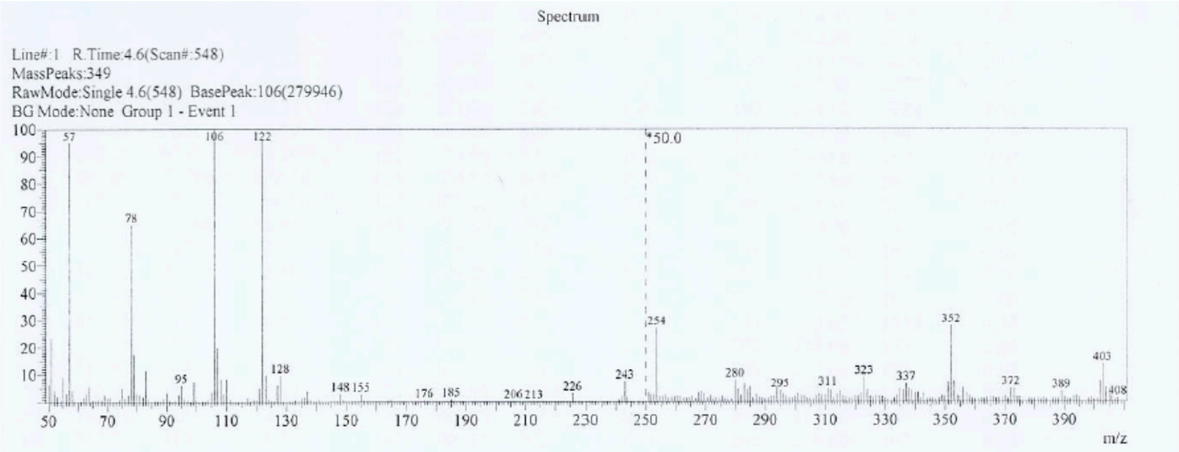

Figure S143. MS (ESI) of Compound **39**.

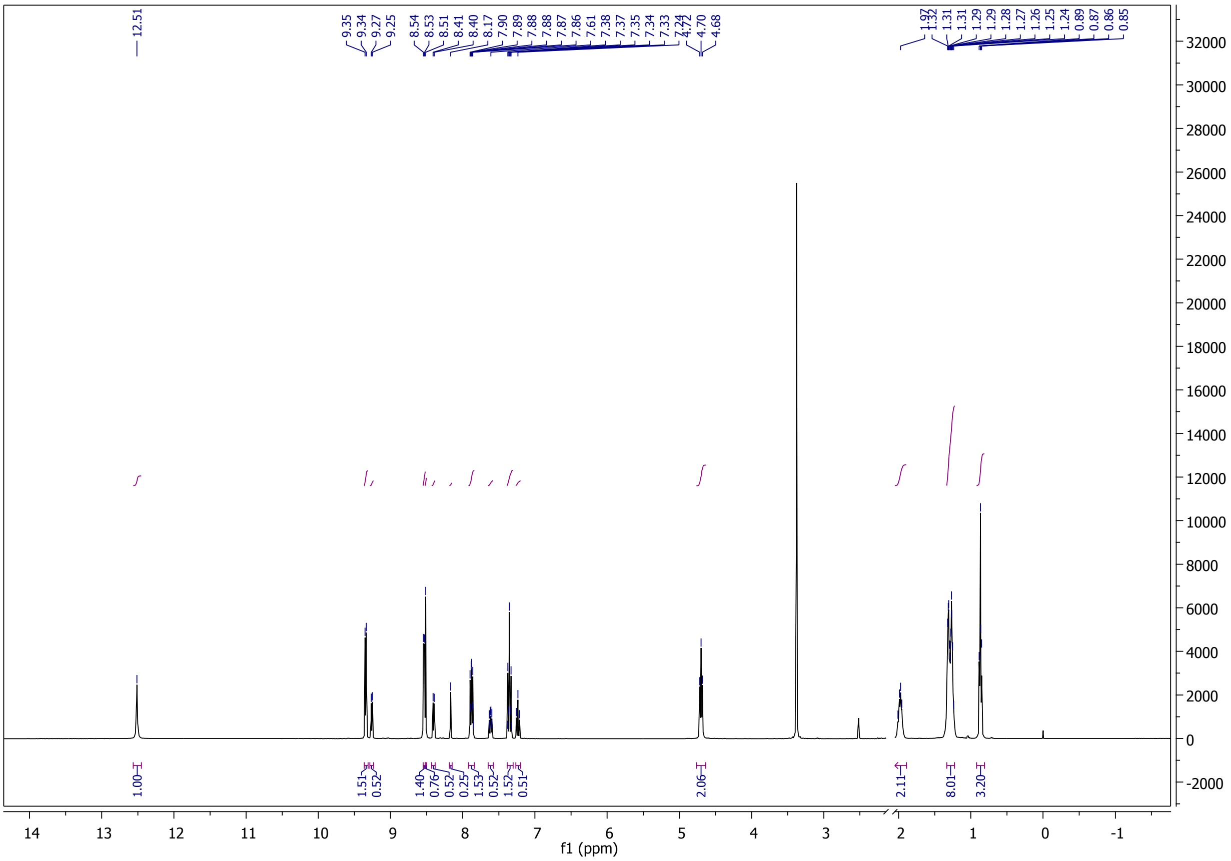

Figure S144. ^{1}H NMR of Compound **40**.

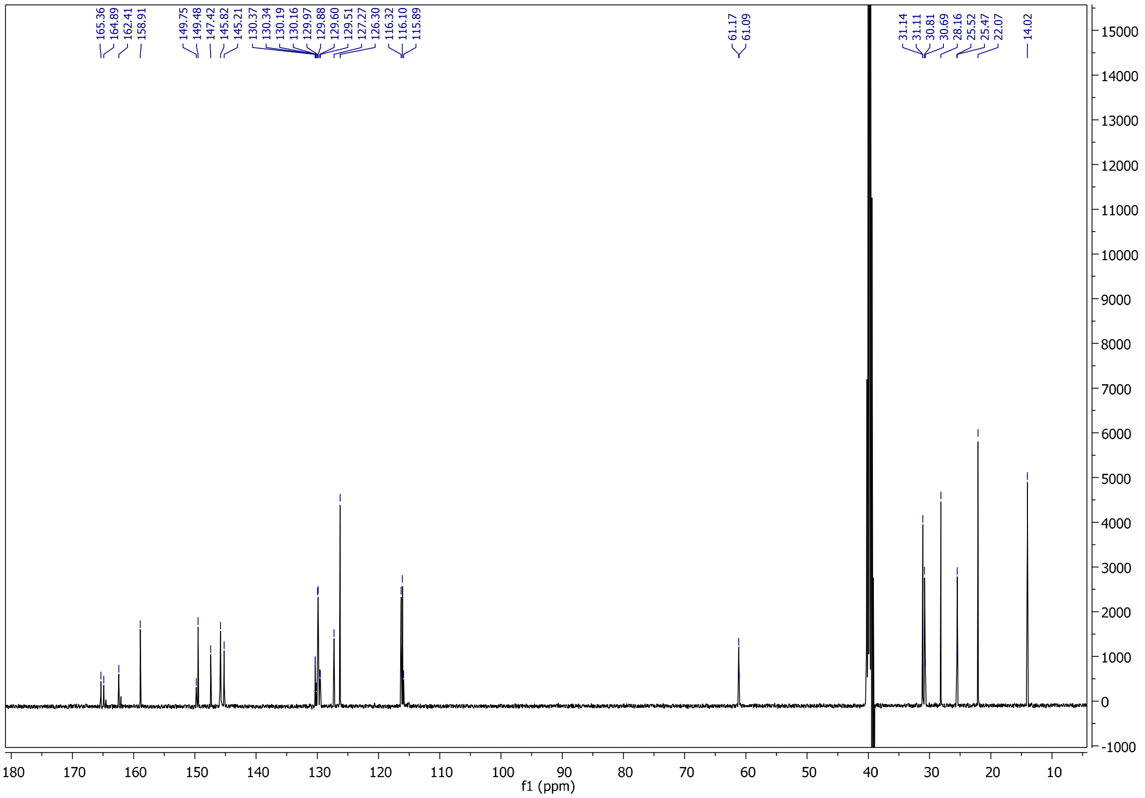

Figure S145. ^{13}C NMR of Compound **40**.

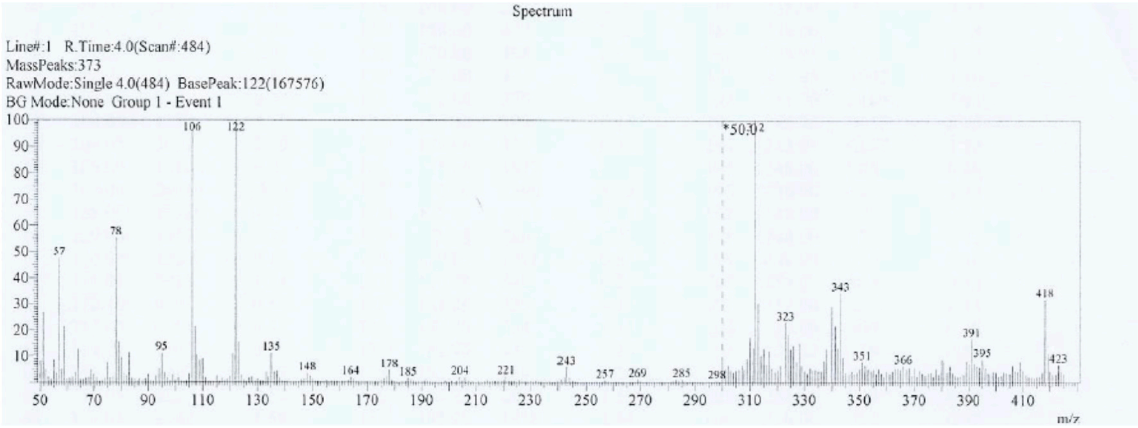

Figure S146. MS (ESI) of Compound **40**.
